# Direct access to chemodivergent Ni(ii) diaryl complexes *via* C–F activation of aryl fluorides

**DOI:** 10.1039/d6dt02433e

**Published:** 2026-10-28

**Authors:** María S. Blankemeyer, Jacob O. Rothbaum, Annalissa Valdez, Camille M. Schubert, Stephen P. DiLuzio, Ryan G. Hadt

**Affiliations:** a Division of Chemistry and Chemical Engineering, Arthur Amos Noyes Laboratory of Chemical Physics, California Institute of Technology Pasadena California 91125 USA rghadt@caltech.edu; b Department of Chemistry and Biochemistry, California State University, Los Angeles Los Angeles CA 90032 USA

## Abstract

The activation of C–F bonds remains a central challenge in organometallic chemistry due to their exceptional bond strength and the limited availability of practical synthetic methods for converting fluorinated feedstocks into well-defined organometallic intermediates. Here, we report a general and practical strategy for C–F activation of polyfluoroarenes using a commercially available Ni(0) precursor, ligands, and aryl fluorides, providing direct access to a diverse family of air- and moisture-stable Ni(ii) diaryl complexes. This method exhibits high regioselectivity across substituted polyfluoroarenes, favoring activation of the C–F bond *para* to the nonfluorine substituent or the ring heteroatom. It also displays high chemoselectivity, as demonstrated by selective C–F over C–H activation of C_6_F_5_H. Fifteen complexes were characterized by single-crystal X-ray diffraction, enabling direct comparison of structural variation across the substrate and ligand series. We further demonstrate that Ni(cod)_2_ can engage PyF_5_ in the absence of a preinstalled bipyridine, providing a bpy-free, MeCN-ligated Ni(ii) diaryl synthon that undergoes subsequent ligand substitution. This platform for installation of two polyfluoroaryl moieties *via* C–F activation bypasses traditional salt metathesis approaches that rely on prefunctionalized and often hazardous organometallic reagents. Finally, these well-defined complexes exhibit stimulus-dependent chemodivergent reactivity, with thermal and photochemical activation producing distinct distributions of hydrodefluorinated and C(*sp*^*2*^)–C(*sp*^*2*^) homocoupled products under otherwise identical solvent and atmospheric conditions. This ability to switch reaction pathways through external stimuli, rather than ligand or reagent changes, introduces a new dimension of control in Ni-mediated bond activation chemistry.

## Abstract 2

Dual C–F activation of polyfluoroarenes by Ni(cod)_2_ enables direct access to chemodivergent and bench-stable Ni(ii) diaryl complexes.

## Graphical abstract

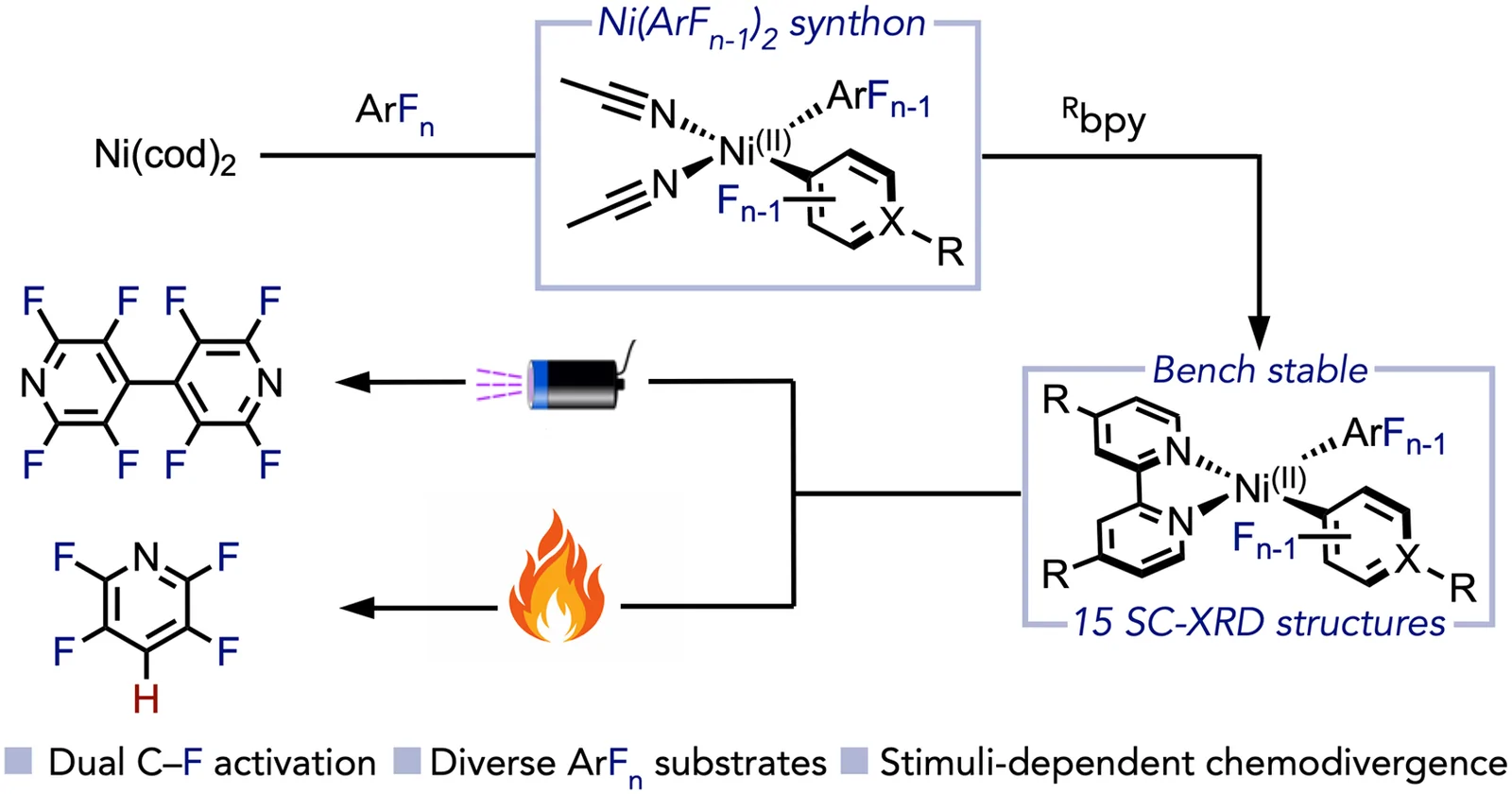

## Introduction

Fluorine-containing molecules play a central role in modern pharmaceutical, agrochemical, and materials chemistry.^1–3^ The physiochemical and metabolic properties imparted by fluorine substitution have driven sustained efforts to develop methods for the precise incorporation and modification of C–F bonds. Significant advances in nucleophilic and electrophilic fluorination strategies have enabled access to partially fluorinated frameworks under increasingly mild and functional-group-tolerant conditions.^4^ Complementary to these approaches, selective C–F activation of inexpensive, polyfluorinated feedstocks offers a promising route to diverse partially fluorinated scaffolds through defluorinative functionalization.^5^ Despite their broad importance, selective manipulation of existing C–F bonds remains a fundamental challenge due to the substantial thermodynamic stability of C–F bonds, although bond strength and reactivity can vary considerably depending on the degree and pattern of fluorination.^6^ In particular, transition-metal (TM) mediated C–F bond activation has emerged as a powerful platform, enabling oxidative addition into C–F bonds followed by downstream bond-forming pathways.

Studies involving Pd,^7,8^ Rh,^9–11^ Ru,^12^ Pt,^13–17^ and Au^18–21^ established the feasibility of TM-mediated C–F bond activation. While these systems provided foundational insights, many compete with C–H activation (BDE_Ph–H_ = 110 kcal mol^−1^),^6^ require strongly fluorophilic reductants (*e.g.*, Al-hydrides) or silane additives to overcome the stability of TM–F products, or are limited to products derived from a single C–F activation event.^4^ Furthermore, Pd, Rh, and Pt systems usually exhibit exclusive *para*-selectivity toward C–F bond activation.^22–24^ To address these challenges, Ni has emerged as a particularly promising platform for C–F activation of polyfluoroarenes. Work by Fahey and Mahan demonstrated that Ni(0)(PEt_3_)_2_ reacts with hexafluorobenzene to afford *trans*-Ni(PEt_3_)_2_(C_6_F_5_)F, albeit in low yield and over extended reaction times.^25^ Subsequent studies by Perutz and McGrady provided crystallographic confirmation of C–F activation and expanded the scope to substrates such as pentafluoropyridine (PyF_5_), which unlike previous precious-metal based systems underwent predominantly *ortho*-activation, but was still not fully selective.^26^ In a catalytic context, Martin and co-workers subsequently demonstrated Ni(cod)_2_/PCy_3_-catalyzed borylation of aryl fluorides, including naphthyl fluoride, providing an important precedent for engaging aryl C–F bonds with a Ni(0)/phosphine system.^27^ More recently, N-heterocyclic carbene (NHC)-stabilized Ni complexes have demonstrated enhanced efficiency in C–F activation and defluorinative functionalization of highly fluorinated arenes (Fig. 1a).^28–30^ However, mechanistic interrogation of both phosphine- and NHC-supported Ni platforms revealed that ligand cooperativity was operative during C–F activation, with certain polyfluoroarene substrates activated even in the presence of phosphine or NHC ligands alone.^31–36^

**Fig. 1:**
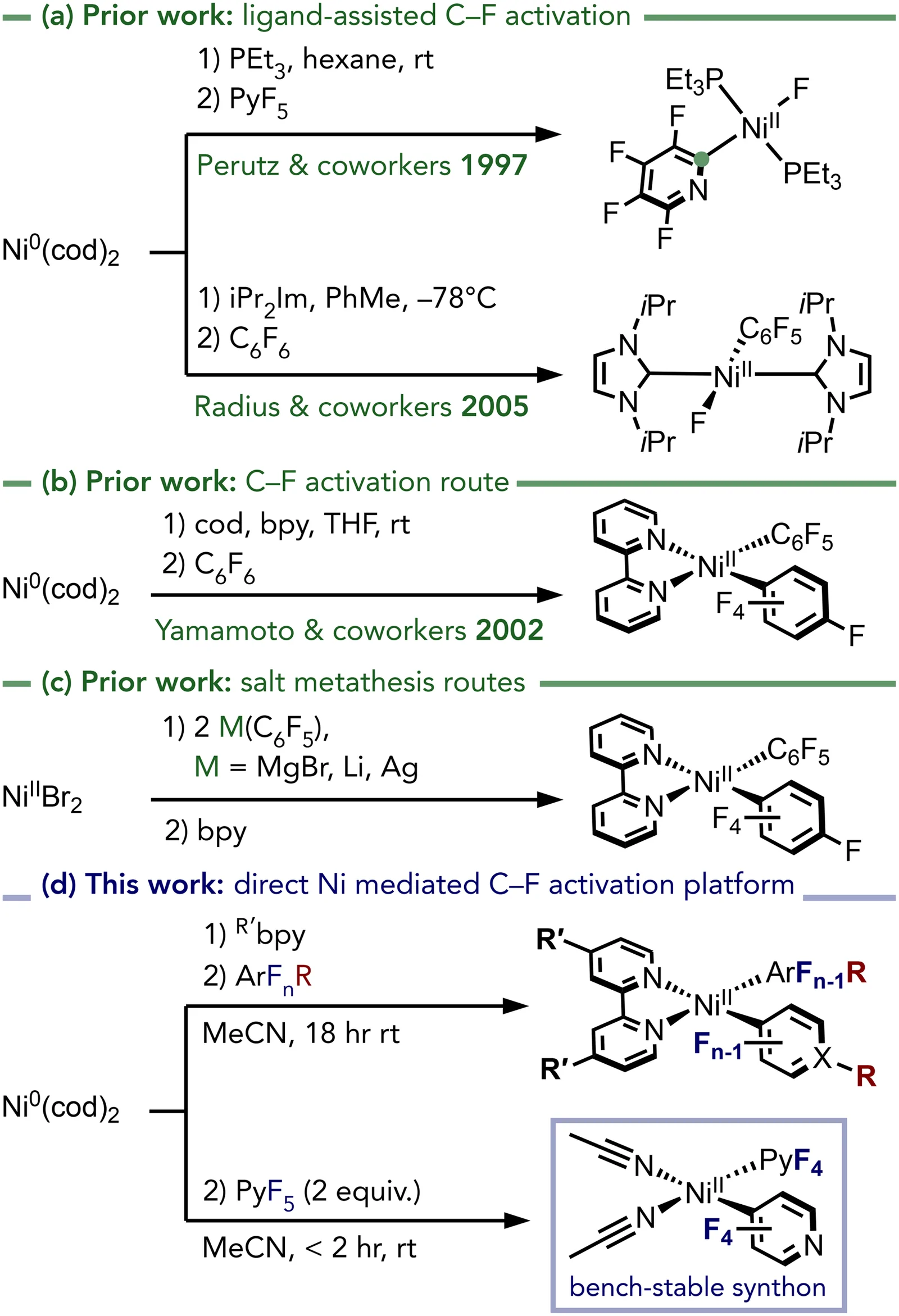
Representative stoichiometric routes to well-defined ArF_*n*_-containing Ni complexes. (a) Previous work with Ni(cod)_2_ (1) mediated C–F activation of pentafluoropyridine (PyF_5_) with phosphine ligands (top)^26^*vs.* activation of hexafluorobenzene (C_6_F_6_) with NHC ligands (bottom).^28^ (b) Yamamoto's preparation of (bpy)Ni(C_6_F_5_)_2_ from 1, bpy, cod, and C_6_F_6_ in THF.^37^ (c) Representative salt-metathesis routes to Ni(ii) perfluoroaryl complexes using preformed organometallic transfer reagents.^38–40^ (d) This work: a general platform to access (^R′^bpy)Ni(ArF_*n*−1_)_2_ (R′ = OMe, *t*-Bu, H, CO_2_Me, and CF_3_) complexes *via* direct C–F activation of the parent aryl or pyridyl fluoride (ArF_*n*_, X = C and N) (top); air- and moisture-stable synthon for facile ligand exchange formed *via* C–F activation (bottom).

Bidentate nitrogen donors such as 2,2′-bipyridine (bpy) provide an electronically distinct coordination environment from the strongly σ-donating phosphine and NHC ligands commonly used in Ni-mediated C–F activation. Bpy-supported nickel complexes are central to modern cross-electrophile coupling because of their stability, modularity, and accessibility from commercially available precursors, yet their ability to generate well-defined organonickel products directly from aryl fluorides remains comparatively underexplored. An early study from the Yamamoto group demonstrated the first example of a stable *cis*-type diaryl nickel(ii) complex, (bpy)Ni(ii)(C_6_F_5_)_2_, prepared from (bpy)Ni(Et)_2_ and hexafluorobenzene.^41^ They subsequently demonstrated that the same complex could be prepared directly from Ni(cod)_2_, bpy, cod, and C_6_F_6_ in THF (Fig. 1b).^37^ These studies established an important precedent for accessing a bpy-supported bis(perfluoroaryl)nickel(ii) complex through C–F activation. However, the investigation was limited to the symmetric C_6_F_6_ substrate and parent bpy ligand and did not address chemo- or regioselectivity across substituted aryl fluorides, establish the air- and moisture-stability of this class of complexes, or examine how ligand substitution and molecular structure influence subsequent thermal and photochemical reactivity.

Recently, Xie and coworkers reported a photoexcited Ni-catalyzed cross-electrophile coupling between polyfluoroarenes and aryl chlorides, wherein a Li salt can be used to steer regioselectivity,^42,43^ and a (^*t*-Bu^bpy)Ni(C_6_F_4_H)_2_ species was identified as a key, catalytically-competent intermediate. Even so, fluorinated Ni complexes have almost exclusively been prepared through salt-metathesis and transmetallation approaches, including studies by Vicic, Klein, and others employing preformed perfluoroaryl reagents to access well-defined Ni–perfluoroaryl and fluorinated alkyl architectures.^44–49^ Although Ni(ii) perfluoroaryl complexes such as (bpy)Ni(C_6_F_5_)_2_ can be obtained through salt metathesis using preformed organometallic reagents (*e.g.*, Ag(C_6_F_5_), Li(C_6_F_5_), and (C_6_F_5_)MgBr), these approaches require preactivated substrates, introduce additional handling considerations, and add an additional synthetic step rather than directly engaging the C–F bond (Fig. 1c).^38–40,50^ Consequently, general and practical strategies for generating well-defined Ni(ii) diaryl platforms directly from simple aryl fluorides *via* C–F activation remain underdeveloped.

Building on this precedent, we report a broadly applicable strategy for installing two ArF_*n*−1_ groups at Ni through C–F activation of parent polyfluoroarenes (ArF_*n*_), affording a family of air- and moisture-stable (^R^bpy)Ni(ii)(ArF_*n*−1_)_2_ complexes across varied aryl fluoride and bipyridine structures (Fig. 1d). The net transformation incorporates two ArF_*n*−1_ groups derived from two equivalents of the parent polyfluoroarene, affording isolable products of Ni-mediated C–F activation. The methodology accommodates a wide range of electronically and sterically diverse substrates, including those bearing electron-donating groups, extended π-systems, and reactive functional groups. High regioselectivity is observed across the scope, generally favoring C–F activation *para* rather than *ortho* to the nonfluorine substituent or the ring heteroatom. The reaction also exhibits high chemoselectivity, including preferential C–F activation in the presence of a potentially reactive C–H bond. The complexes were characterized by multinuclear NMR and UV–vis electronic absorption spectroscopy, and 15 members of the series were additionally characterized by SC-XRD. Additional studies further show that Ni(cod)_2_ (1) alone can promote C–F activation of pentafluoropyridine in acetonitrile to yield a bench-stable *cis*-(MeCN)_2_Ni(ii)(*p*-PyF_4_)_2_ complex, which was isolated and characterized by SC-XRD. This complex, made *via* direct engagement of a C–F bond in two equivalents of the parent ArF_*n*_, has been successfully utilized as a synthon for multiple (^R^bpy)Ni(ii)(PyF_4_)_2_ complexes. Moreover, these complexes exhibit stimulus-dependent chemodivergent reactivity, wherein selectivity between hydrodefluorination and C(*sp*^*2*^)–C(*sp*^*2*^) homocoupling can be modulated through thermal *vs.* photochemical activation, respectively. Importantly, this chemodivergence is observed under identical reaction conditions and does not require any additional reagents, ligands, or manipulations. Collectively, these findings define a robust and modular platform for installation of two ArF_*n*–1_ groups through C–F bond activation of the parent polyfluoroarenes for the preparation of air- and moisture-stable Ni complexes.

## Results and discussion

### Reaction development and substrate scope

Reaction optimization for the formation of 3a from (^*t*-Bu^bpy)Ni(0)(cod), generated *in situ* from Ni(cod)_2_ (1) and 4,4′-di-*tert*-butyl-2,2′-dipyridyl (^*t*-Bu^bpy), and one equivalent of pentafluoropyridine (PyF_5_, 2) revealed that solvent identity strongly influences the observed C–F activation selectivity. Benzene and toluene favor *ortho* activation, whereas THF favors *para* activation and MeCN furnishes exclusively the *para*-activated product, indicating that several solvent-dependent factors—including coordination, cod displacement, intermediate trapping, and solubility—may contribute to the observed product distribution. Lower reaction concentrations suppress competing C(*sp*^*2*^)–C(*sp*^*2*^) homocoupling and maximize the formation of the desired Ni(ii) diaryl product. The concentration dependence is consistent with, but does not establish, competing pathways involving more than one nickel center. With these mild, additive-free conditions (MeCN, rt, see SI section 2 for full optimization), we next investigated the generality and selectivity for incorporation of two polyfluoroaryl groups *via* C–F activation across a range of commercially available polyfluoro(hetero)arenes (Fig. 2). Across the examined scope, the net reaction installs two ArF_*n*−1_ groups at Ni(ii), furnishing (^*t*-Bu^bpy)Ni(ii)(ArF_*n*−1_)_2_ complexes directly from the parent aryl fluoride at room temperature and under additive-free conditions.

**Fig. 2:**
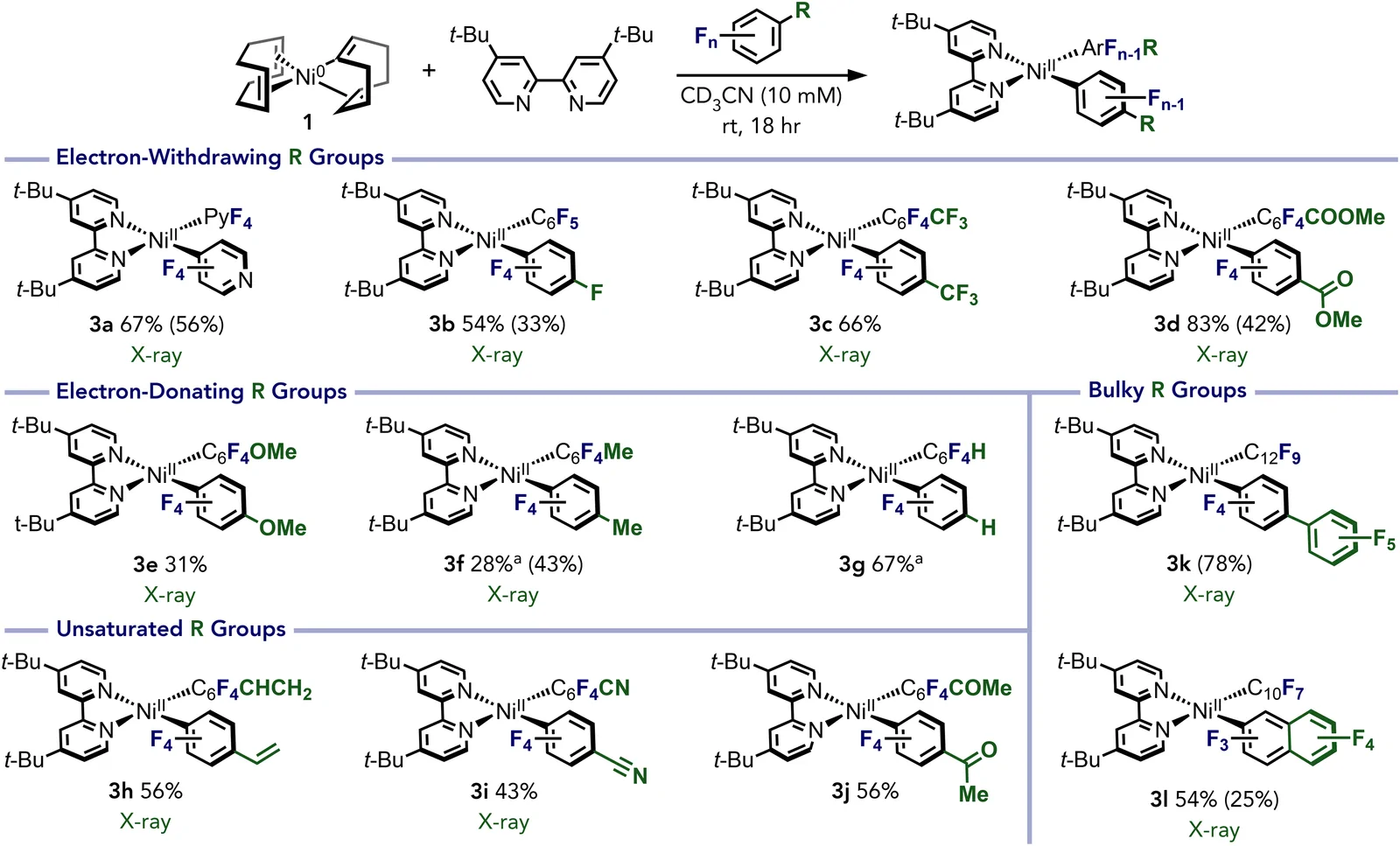
Substrate scope for C–F activation of polyfluoroarenes. Yields for each complex were calculated by quantitative ^19^F NMR spectroscopy employing 1,4-difluorobenzene or trifluorotoluene as an internal standard in CD_3_CN (10 mM). Isolated yield shown in parentheses. ^*a*^ Reaction heated to 60 °C.

The preliminary DFT-validated mechanism predicts initial formation of a LNi(ii)(ArF_*n*−1_)F species followed by redistribution to the observed diaryl complex and a nickel–fluoride-containing coproduct (where L = ^*t*-Bu^bpy). This formulation gives the limiting stoichiometry presented in Scheme 1, where LNi(ii)F_2_ was used only for stoichiometric bookkeeping and was not experimentally observed. Crude-reaction ^1^H NMR and positive-ion ESI-MS measurements support the formation of a paramagnetic nickel–fluoride-containing species but do not identify a unique coproduct (see SI section 2). The limiting stoichiometry also means that no more than 50% of the initial nickel can be incorporated into the desired diaryl product under 1 : 1 Ni/ArF_*n*_ conditions. Preliminary electronic-energy calculations evaluating this and other chemically balanced transformations are provided in the SI (see SI section 6).

**Scheme 1:**
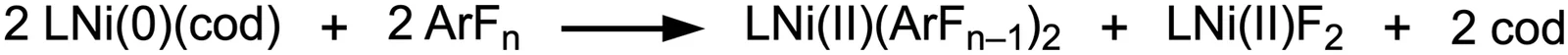
Chemically balanced working model for the formation of the Ni(ii) diaryl product where L = ^*t*-Bu^bpy. The depicted transformation represents thermodynamically evaluated possibilities rather than an established sequence of elementary steps; the Ni–F-containing coproduct has not been structurally assigned.

Across the scope, the *para*-functionalized complex is the major, and in many cases exclusive, regioisomer. Notably, 15 complexes spanning the substrate and ligand scope were characterized by SC-XRD, establishing the structural generality of this Ni(ii) diaryl platform (*vide infra*), and all isolated complexes are air- and moisture-stable in solution and as solids. Electron-deficient substrates, including C_6_F_6_ and CF_3_-substituted and CO_2_Me-substituted polyfluoroarenes, are converted into the corresponding Ni(ii) diaryl complexes (3b, 3c, and 3d, respectively) in good yields, with each structure confirmed by SC-XRD.

Electron-donating substituents were also tolerated (3e and 3f), albeit in more modest yields (Fig. 2) because of increased formation of hydrodefluorinated products (Fig. 2; see SI section S3.1).^51,52^ These substrates are traditionally challenging in C–F activation chemistry, as electron donation strengthens the C–F bond and reduces electrophilicity at the *ipso*-carbon.^53^ Nevertheless, *para*-selectivity is retained for both electron-rich substrates, and the structures of 3e and 3f were confirmed by SC-XRD.

The system further displays high chemoselectivity. With C_6_F_5_H as the substrate (3g), no products arising from C–H activation were detected despite the weaker C–H bond and the established ability of fluorine substituents to direct C–H activation.^54^ Instead, activation occurs selectively at the C–F bond *para* to hydrogen to furnish 3g, although hydrodefluorination was again observed as a competing pathway (Fig. S26). In contrast to phosphine-supported Ni complexes in which C–H activation can compete in the presence of C–F bonds,^55–57^ the present platform selectively engages the C–F bond.

To probe whether unsaturated, potentially π-coordinating functional groups interfere with C–F activation, several such substrates were examined. Pentafluorostyrene underwent exclusive activation of the C–F bond *para* to the vinyl group to afford 3h, despite prior reports of preferential olefin coordination in Ni(0)(PEt_3_)_2_ systems.^58^ To our knowledge, this is the first example of Ni-mediated C–F activation of pentafluorostyrene to furnish a diaryl Ni(ii) complex, whose structure was confirmed by SC-XRD; previous C–F activation of this substrate has been largely limited to Rh complexes.^59^ Nitrile- and ketone-substituted substrates were similarly tolerated, furnishing 3i and 3j, respectively, without detectible modification of these functional groups. The structure of 3i was also confirmed by SC-XRD. Retention of the vinyl group in 3h is particularly notable because styrene derivatives are valuable substrates for radical polymerization.^60,61^

Polyfluoroarenes with extended π-systems and greater steric demands, including decafluorobiphenyl and octafluoronaphthalene, also underwent C–F activation to furnish 3k and 3l, respectively; both structures were confirmed by SC-XRD. Despite the increased steric congestion and nonplanar arene geometries, net installation of two aryl groups occurred at room temperature and produced bench-stable Ni(ii) complexes.^29,62–64^

Collectively, this scope demonstrates the net installation of two ArF_*n*−1_ groups at Ni is compatible with substantial variation in substrate electronics, steric demand, and functional-group reactivity. Direct use of the parent polyfluoroarenes under mild, additive-free conditions provides access to a structurally diverse family of well-defined, bench-stable Ni(ii) diaryl complexes relevant to C–F functionalization and cross-coupling chemistry.

### Ligand effects and synthons

Changing the supporting ligand to other bipyridines afforded complexes 4a (^MeO^bpy) and 4b (bpy), respectively, and their structures were confirmed *via* SC-XRD. In contrast, bipyridines bearing electron-withdrawing substituents (R′ = CF_3_ and CO_2_Me) did not promote C–F activation at Ni(0) under identical conditions, indicating that ligand electronics influence C–F activation at the Ni center. To circumvent the lack of reactivity with electron-withdrawing bpy ligands, the ability of 1 to independently activate C–F bonds was investigated.

Reacting 1 with excess 2 in MeCN furnished complex 5 in 12% isolated yield, which was definitively characterized by SC-XRD. The modest, isolated yield reflects the challenges associated with isolation and purification rather than its utility *in situ* (see SI section 3.2). Indeed, complex 5 serves as a competent synthon for ligand substitution, furnishing complexes 4a, 4c, and 4d in moderate yields (44–55% isolated yields, all confirmed by SC-XRD, Fig. 3). Thus, despite its low isolated yield, 5 provides a practical entry point to a larger class of (*e.g.*, electron-withdrawing ligands) bpy-supported Ni(ii) diaryl architectures. Complexes of the general type L_*n*_Ni(C_6_F_5_)_2_ are commonly prepared *via* salt metathesis using Ag(C_6_F_5_), Li(C_6_F_5_), or (C_6_F_5_)MgBr.^38,39,49,65–79^ In contrast, synthon 5 is accessible from commercially available starting materials through net C–F activation, is air- and moisture-stable, and avoids the use of pyrophoric organolithium or Grignard reagents. Moreover, the formation of 5 in the absence of a preinstalled bipyridine establishes that Ni(cod)_2_ itself is competent to engage PyF_5_. This observation supports the use of 5 as a ligand-independent entry point to bpy-supported Ni(ii) diaryl complexes.

**Fig. 3:**
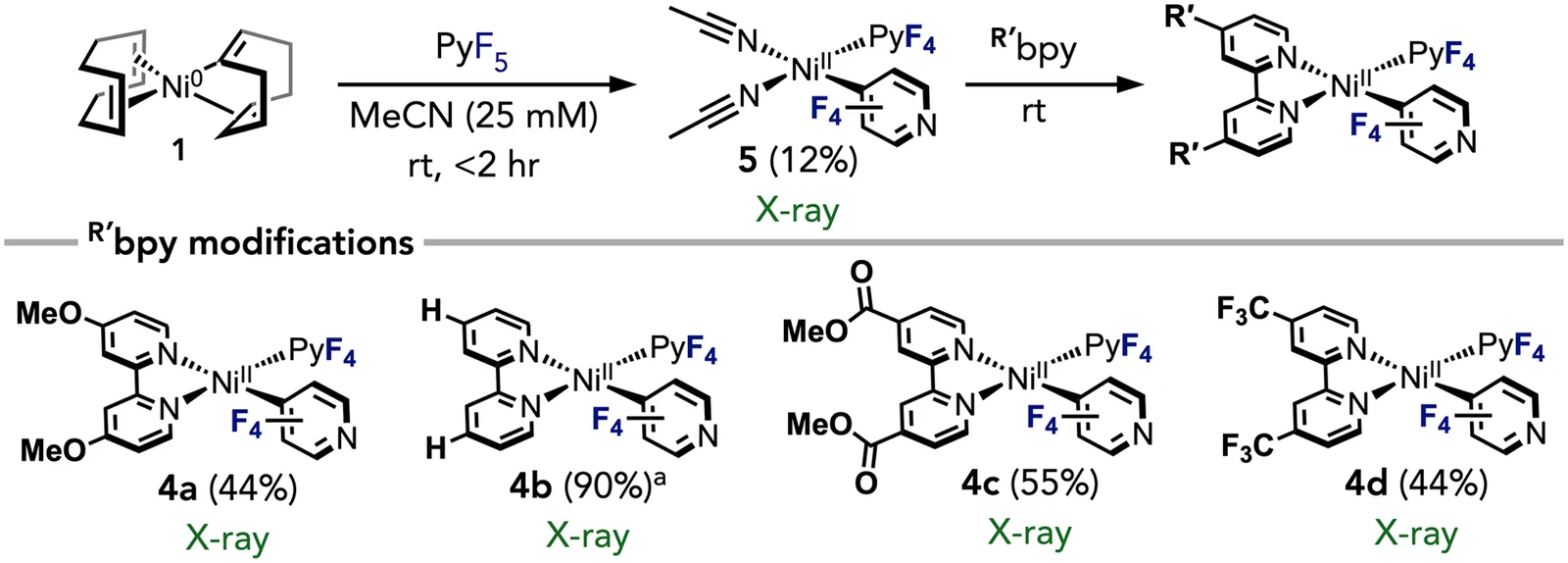
Bipyridine ligand scope and complementary routes to (R′bpy)Ni(PyF_4_)_2_ complexes. ^a^Complex 4b was prepared directly from the corresponding bpy-supported Ni(0) precursor through the route shown in Fig. 2 and is included here to facilitate comparison across the ligand series. Reported yields in parentheses are isolated yields.

Taken together, the direct C–F activation and ligand-substitution pathways substantially expand upon Yamamoto and co-workers’ preparation of (bpy)Ni(C_6_F_5_)_2_ from Ni(cod)_2_, bpy, and C_6_F_6_.^37^ Whereas that study established access to a single complex derived from symmetric C_6_F_6_ and unsubstituted bpy, the present platform encompasses substituted polyfluoroarenes and electronically varied bipyridine ligands, thereby revealing chemo- and regioselectivity, ligand-dependent reactivity, and the stability of the resulting complexes. Again, related L_n_Ni(C_6_F_5_)_2_ complexes have more commonly been accessed through salt metathesis or transmetalation.^42,50^ For several substrates examined here, however, the corresponding monobrominated polyfluoroarene commonly used to prepare such organometallic transfer reagents is not commercially available, making these conventional routes less direct and requiring additional precursor syntheses. Moreover, organolithium and Grignard reagents are incompatible with electrophilic functional groups represented in the present scope, particularly ester and nitrile substituents, because these groups are susceptible to nucleophilic addition. In contrast, the present strategy directly engages the parent polyfluoroarene, while synthon 5 provides a modular route to bpy-supported Ni(ii) diaryl complexes bearing ligands that do not support direct C–F activation from Ni(0).

### SC-XRD

Fifteen complexes from the substrate and ligand scope were characterized by SC-XRD, enabling comparison of structural variation across the series (Fig. 4–6). The bpy-supported complexes adopt slightly distorted square-planar geometries featuring a chelating bipyridine ligand and two ArF_*n*−1_ groups derived from C–F activation, whereas the bpy-free structure of synthon 5 is discussed separately below. Distortion was quantified using two parameters: (1) “planar twist,” defined as the angle between the N1–Ni–N2 plane of bpy and the C1–Ni–C2 plane of ArF_*n*_*ipso*-C (square-planar = 0°), and (2) “ArF_*n*–1_ tilt,” defined as the angle between each ArF_*n*–1_ ring plane and the N1–Ni–N2 donor plane (ideal = 90°).

**Fig. 4:**
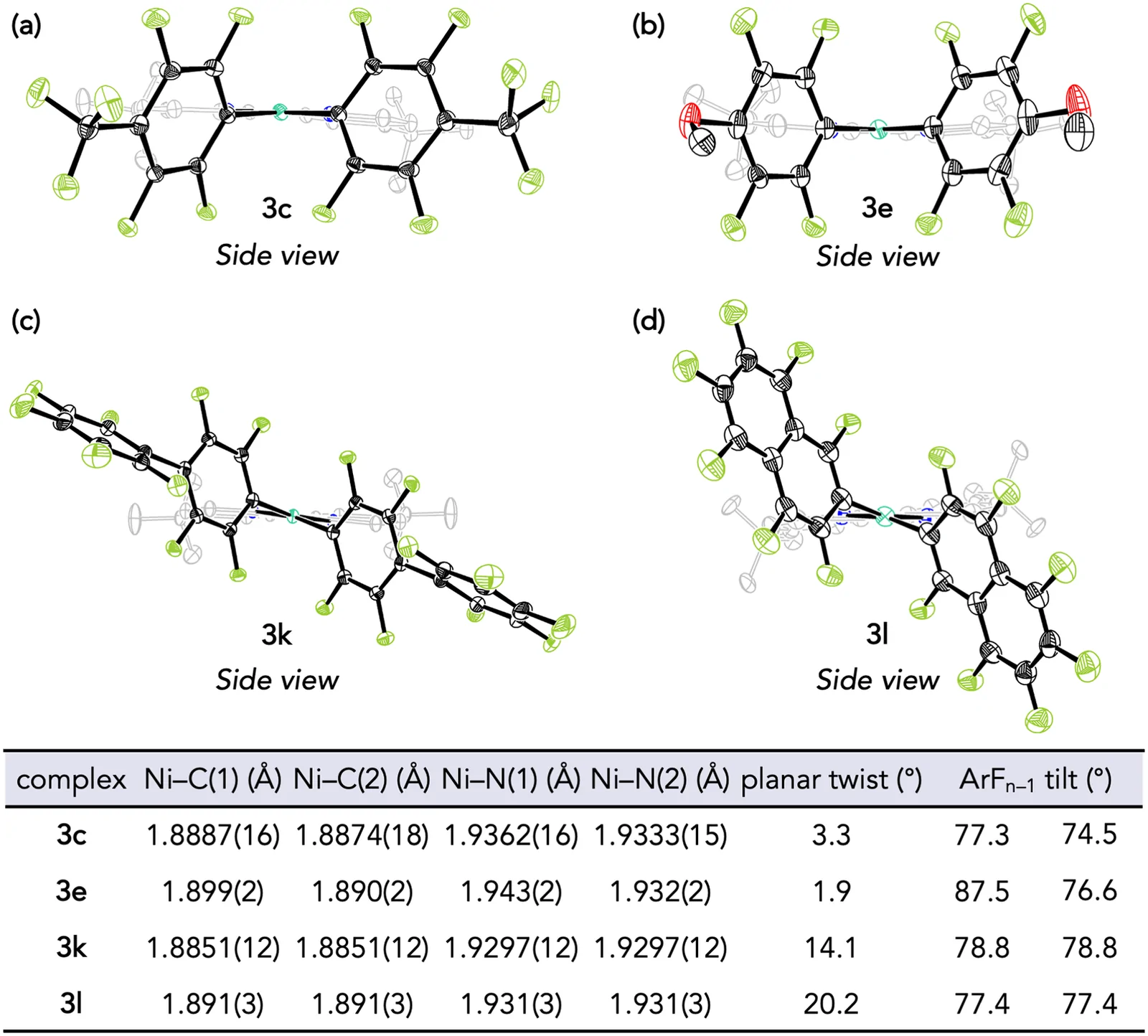
Selected SC-XRD structures of Ni-bpy diaryl complexes with various arenes. (a–d) ORTEP drawings of SC-XRD structures of 3c, 3e, 3k, and 3l, respectively, with ellipsoids drawn at the 50% probability level. H atoms for all, solvent molecules from 3c and 3l, and disordered parts of 3c are omitted for clarity. Selected crystallographic parameters are given at the bottom. Bond distances are given in Å (ESDs in parentheses); least-squares interplanar angles are given in degrees.

**Fig. 5:**
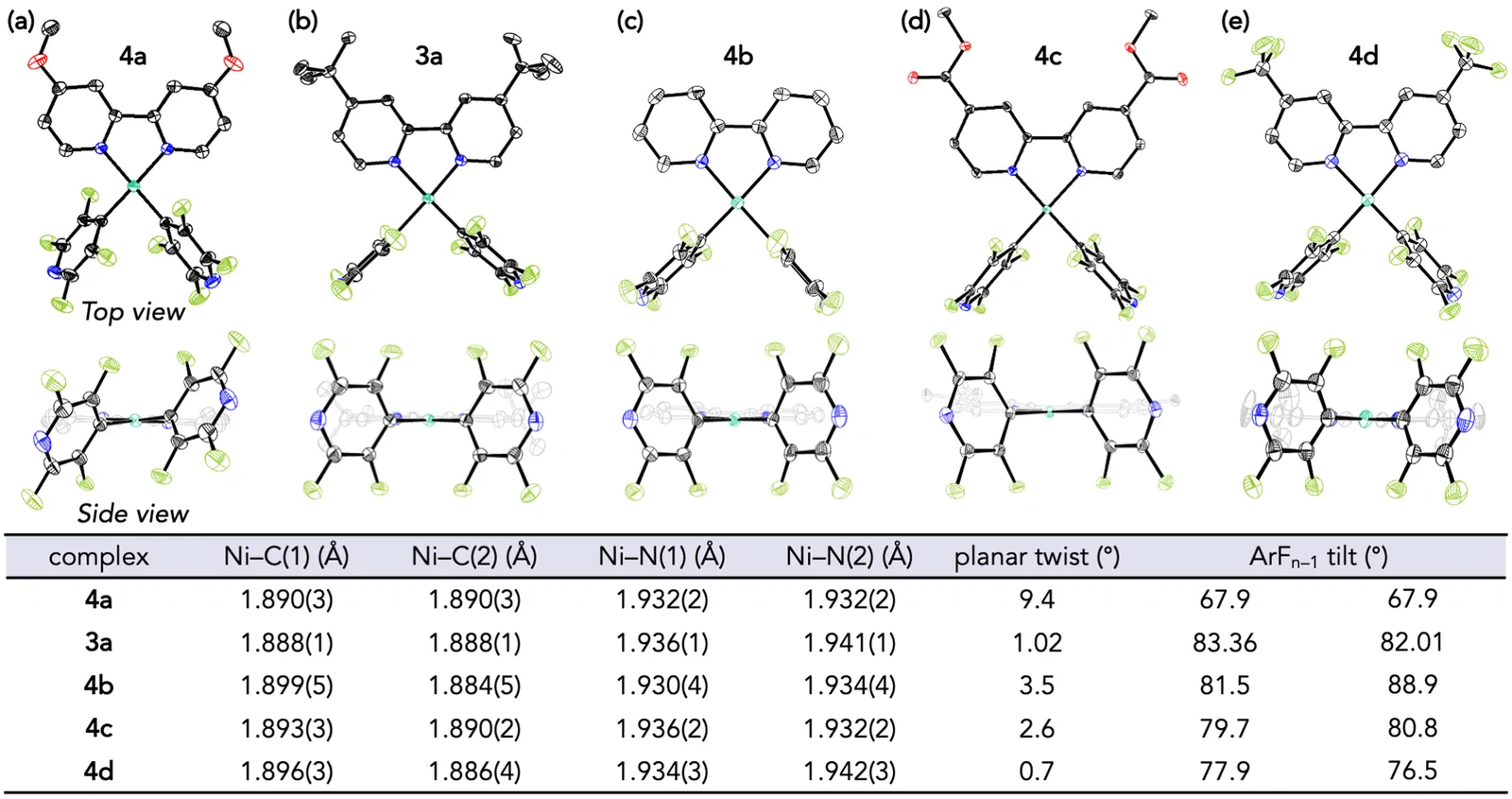
Select SC-XRD structures of Ni-bpy diaryl complexes with various arenes. (a–e) ORTEP drawings of SC-XRD structures of 3c, 3e, 3k, and 3l, respectively, with ellipsoids drawn at the 50% probability level. H atoms for all, solvent molecules from 3c and 3l, and disordered parts of 3c are omitted for clarity. Selected crystallographic parameters are given at the bottom. Bond distances are given in Å (ESDs in parentheses); least-square interplanar angles are given in degrees.

**Fig. 6:**
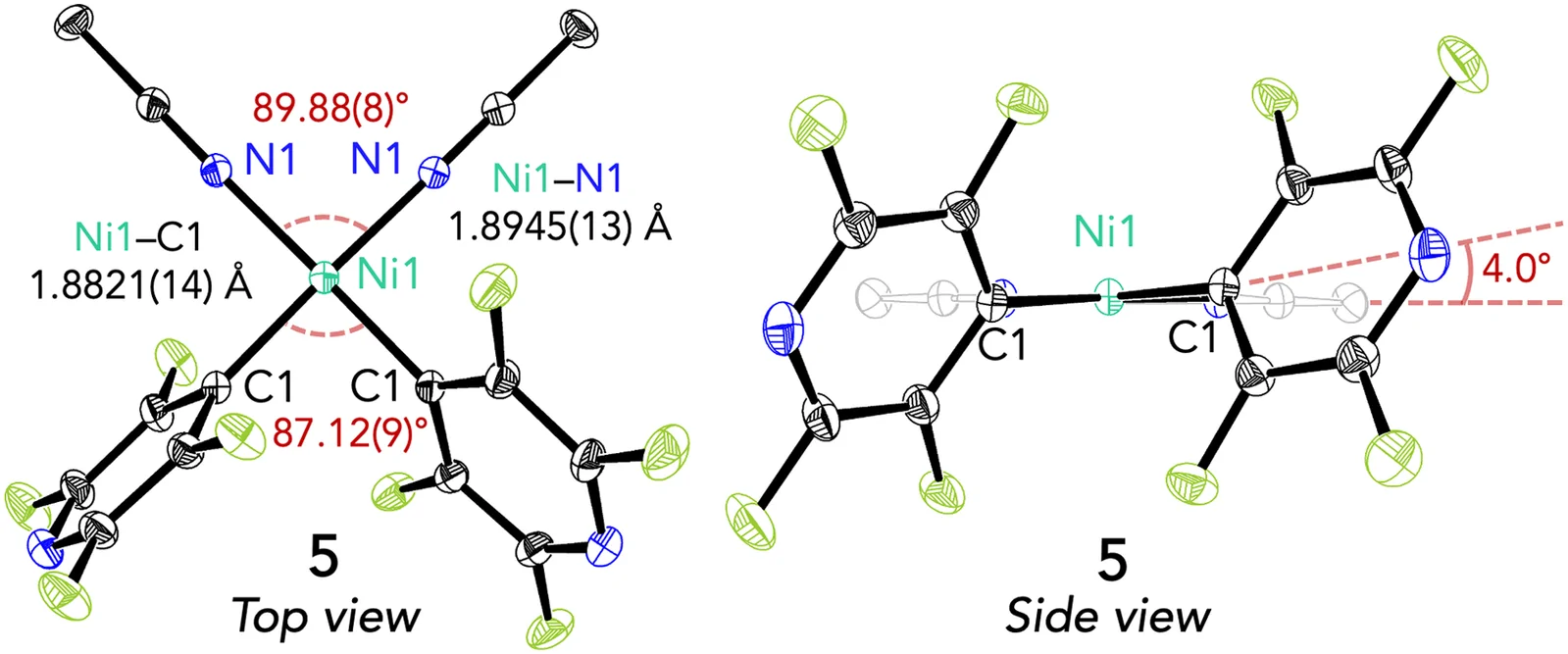
SC-XRD structure of the Ni diaryl synthon. ORTEP drawings of 5 with ellipsoids drawn at the 50% probability level. H atoms are omitted for clarity. Bond distances are given in Å (ESDs in parentheses); least-square interplanar angles are given in degrees.

Comparison of complexes 3c and 3e, which bear substituents with differing inductive character, reveals minimal perturbation of the metal coordination sphere. Ni–C bond lengths span a narrow range of 1.885–1.899 Å, while Ni–N bond lengths fall between 1.930–1.943 Å (Fig. 4a and b). The planar twist angles also remain small (1.9–3.3°), and ArF_*n*_ tilt angles are comparable (Fig. 4a and b). Thus, although substrate electronics influence reactivity, variation in substituent inductive character produces no systematic differences in the measured bond lengths of solid-state geometries of the resulting Ni(ii) diaryl complexes.

Complexes containing larger, extended ArF_*n*–1_ groups exhibit greater deviation from square-planar coordination at Ni. The planar-twist angles of 3k and 3l are 14.1° and 20.2°, respectively, compared with 1.9–3.3° for 3c and 3e, while the Ni–C and Ni–N bond lengths remain comparable across the series (Fig. 4c and d). The increased distortion likely reflects the combined influence of intramolecular steric interactions, conformational constraints imposed by the extended aryl groups, and their solid-state packing environments. Consistent with lower molecular symmetry, the ^19^F NMR spectra of 3k and 3l contain inequivalent fluorine environments in solution (Fig. S39 and S42).

Across the series, Ni–C (1.885–1.899 Å) and Ni–N (1.930–1.943 Å) bond lengths are modestly shorter than those reported for (bpy)Ni(Mes)_2_ (Ni–C = 1.91 Å; Ni–N = 1.97 Å).^80^ These contracted bond metrics may reflect the electronic influence of the perfluoroaryl groups, including polarization of the Ni–C bonds by the electron-withdrawing fluorine substituents. Notably, all isolated complexes are air- and moisture-stable solids, remaining unchanged after weeks of exposure to air as monitored by NMR spectroscopy. Steric shielding of the Ni center by the two ArF_*n*−1_ groups, together with the electronic influence of the perfluoroaryl ligands, may contribute to this stability.

Variation of the 4,4′-substituents on bpy similarly produces no systematic changes in the Ni–C or Ni–N bond lengths (Fig. 5). Across complexes 4a–4d and 3a, these metrics remain within the narrow ranges observed upon variation of the ArF_*n*−1_ groups (Fig. 5). No consistent trends are observed in the planar twist or ArF_*n*−1_ tilt angles across the bpy series. Thus, substitution at the 4,4′-position modulated ligand electronics while largely preserving the solid-state geometry of the square-planar Ni(ii) diaryl core.

Bpy-free complex 5 adopts a distorted square-planar geometry comprising two *cis*-PyF_4_ groups and two N-bound MeCN ligands (Fig. 6). Relative to 3a, the most pronounced structural difference is the greater deviation of the PyF_4_ rings from orthogonality to the N–Ni–N plane, with 5 exhibiting an ArF_*n*−1_ tilt angle of 66.6°, the largest deviation observed in this study (Fig. 6). The *cis* arrangement of the aryl groups is consistent with literature structural precedents for L_n_Ni(C_6_F_5_)_2_ complexes prepared *via* salt metathesis.^79^ Importantly, displacement of the labile MeCN ligands enables installation of alternative bipyridine ligands to furnish 4a, 4c, and 4d. To our knowledge, 5 is the first structurally characterized, bpy-free, (MeCN)_2_Ni(ArF_*n*−1_)_2_ synthon prepared directly from a parent polyfluoroarene through C–F activation, providing a modular entry point to Ni(ii) diaryl organometallic and catalytic chemistry.

### Thermal and photochemical reactivity

With a variety of isolated and characterized (^R′^bpy)Ni(ii)(ArF_*n*−1_)_2_ complexes in hand, their reactivity was examined under thermal and photochemical conditions. Complexes 3a, 3d, and 4d were selected as representative systems to probe the influence of both ArF_*n*–1_ electronics and bpy ligand identity. All experiments were conducted in DMSO-*d*_6_ in air, enabling direct comparison of stimulus-dependent reactivity.

Thermal reactivity varies substantially across the three complexes. Complex 3a demonstrates appreciable formation of the hydrodefluorinated product, 2,3,5,6-tetrafluoropyridine (PyF_4_H), after several days at 100 °C (Fig. 7a).^50^ Complex 3d, in which the PyF_4_ groups are replaced by C_6_F_4_CO_2_Me groups, is more resistant to thermal reaction, exhibiting <20% conversion after several days at 100 °C and forming predominantly the C–C homocoupled product (Fig. 7b). In contrast, replacement of the 4,4′-*tert*-butyl substituents on bpy with CF_3_ groups substantially increases thermal reactivity, and 4d undergoes near-complete conversion to PyF_4_H within 4 hours at 100 °C (Fig. 7c). These results demonstrate that thermal reaction rates and product distributions are sensitive to both the aryl and bipyridine substituents despite the similar solid-state coordination geometries of the complexes.

**Fig. 7:**
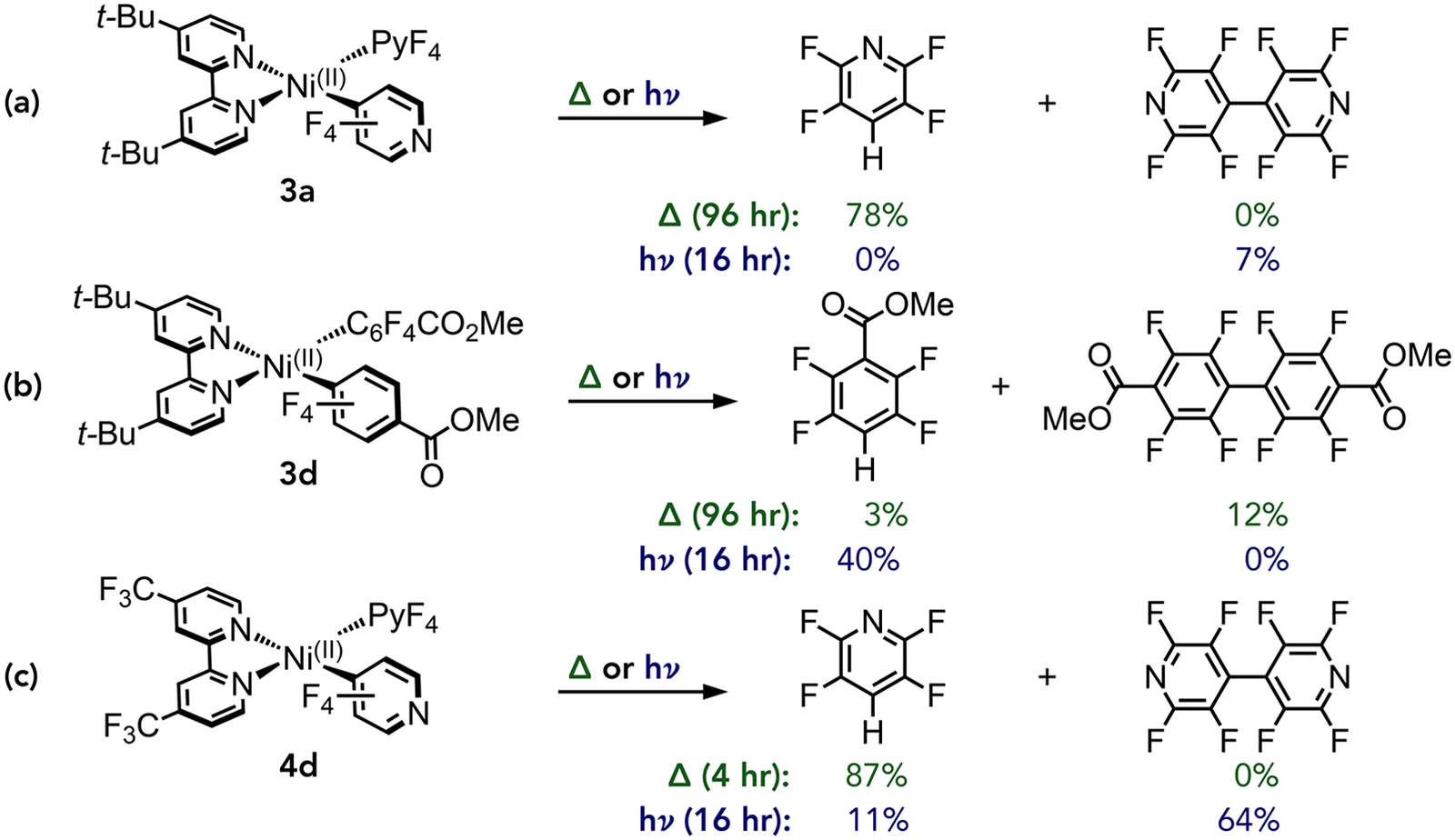
Thermal and photochemical reactivity of Ni-bpy diaryl complexes. Thermally- and photochemically-induced reactivity of representative (^R′^bpy)Ni(ii)(ArF_*n*−1_)_2_ complexes, where R′ = CF_3_ or *t*-Bu and ArF_*n*−1_ = PyF_4_ or C_6_F_4_CO_2_Me. Reactions were monitored by ^19^F NMR with (CF_3_)_3_Ph or 1,4-difluorobenzene as an internal standard. All experiments were conducted in DMSO-*d*_*6*_ in a J-Young NMR tube. Thermal experiments were conducted at 100 °C. Irradiation experiments were performed using a 370 nm Kessil LED at room temperature. Corresponding NMR spectra can be found in the SI (Fig. S57–S63). (a) Reactivity of complex 3a. (b) Reactivity of complex 3d. (c) Reactivity of complex 4d.

The hydrogen source in the hydrodefluorinated products has not been definitively established. Because DMSO-*d*_6_ was used without additional drying, residual water is the plausible proton-source; predominant C–D cleavage of DMSO-*d*_6_ would instead be expected to produce deuterium incorporation. For the reaction of 3a, the diagnostic splitting of the ^19^F NMR resonances is consistent with the formation of PyF_4_H, although this assignment remains tentative because the product was not isolated and independently characterized. Post-reaction ^1^H NMR spectra additionally contain broad paramagnetic resonances, indicating the formation of one or more paramagnetic Ni-containing products whose structures and oxidation states remain unassigned (Fig. S57 and S58).

We next investigated whether photochemical excitation could access distinct reactivity under otherwise identical conditions. Irradiation of 3a (*λ*_max_(3a) = 363 nm, *ε*(363 nm) ∼ 2900 M^−1^ cm^−1^, Fig. S64) with a 370 nm Kessil LED results in minimal conversion, affording only 7% of the homocoupled product after 16 h (Fig. 7a). In contrast, irradiation of 4d (*λ*_max_(4d) = 415 nm, *ε*(415 nm) ∼ 900 M^−1^ cm^−1^, Fig. S64) under the same conditions leads to substantially enhanced reactivity, yielding 64% of the homocoupled product, along with minor hydrodefluorination and an unidentified fluorinated species (Fig. 7c, Fig. S66). The enhanced conversion of 4d under 370 nm irradiation, together with its red-shifted absorption spectrum (Fig. S64), indicates that bpy substitution alters both the charge-transfer absorption profile and the resulting photochemical response.^81^ For the PyF_4_-containing complexes 3a and 4d, the products observed under irradiation are dominated by C–C homocoupling, whereas thermal treatment favors hydrodefluorination, particularly for 4d. Complex 3d exhibits the opposite selectivity; irradiation predominantly affords the hydrodefluorinated product (*λ*_max_(3d) = 375 nm, *ε*(375 nm) ∼ 4900 M^−1^ cm^−1^, Fig. S64), while heating favors C–C homocoupling (Fig. 7b).

Together, these results demonstrate that the mode of activation can strongly bias product distribution under otherwise identical reaction conditions, with the direction and magnitude of this effect depending on both the aryl and bipyridine substituents. While prior studies of *cis*–L_*n*_Ni(ArF_*n*–1_)_2_ complexes have found varying distributions for the formation of homocoupled *vs.* hydrodefluorinated products depending on ligand identity, solvent, and the presence of oxygen,^40,50,79,82^ the present results identify thermal *versus* photochemical excitation as an additional control parameter that alters product selectivity without requiring changes in solvent or additional reagents. Further mechanistic interrogation of these thermal and photochemical pathways is underway.

Based on prior studies of related organonickel complexes, the observed photoreactivity may involve excited-state Ni–C bond homolysis followed by in-cage or out-of-cage radical chemistry.^81,83^ Importantly, all complexes were irradiated at 370 nm, while the energies and intensities of their charge-transfer absorption bands vary with both the bpy ligand and aryl substituents. The different photochemical outcomes of 3a, 3d, and 4d therefore reflect differences in the efficiency of excitation or the specific excited states populated at 370 nm, as well as differences in their subsequent Ni–C bond cleavage, cage recombination, and cage-escape dynamics. Detailed assignment of the relevant excited states and their wavelength-dependent reactivity through time-resolved spectroscopy and computational analysis is ongoing.

## Conclusions

Herein, we report Ni-mediated C–F activation of polyfluoroarenes to furnish air- and moisture-stable (^R′^bpy)Ni(ii)(ArF_*n*−1_)_2_ complexes directly from commercially available starting materials. This strategy bypasses the need for organometallic perfluoroaryl transfer reagents and instead leverages inexpensive aryl fluorides as the aryl source.

A broad substrate scope demonstrates that this transformation is general across electronically diverse polyfluoroarenes, providing consistent access to well-defined Ni(ii) diaryl complexes in moderate to high yield (Fig. 2). Notably, these complexes are bench-stable, and almost all of them were structurally characterized by SC-XRD. Structural analysis across 15 complexes shows that the Ni–C and Ni–N bond lengths and overall square-planar coordination geometries are largely conserved across electronic variation, whereas sterically demanding, extended aryl groups produce greater deviations from planarity (Fig. 4 and 5). This structural robustness underscores the generality of this platform and its tolerance to both ligand and substrate modification. The Ni(ii) diaryl complexes also exhibit stimulus-dependent reactivity, with thermal and photochemical activation producing distinct product distributions under otherwise identical solvent and atmospheric conditions (Fig. 7). The direction and extent of this chemodivergence depend on the identities of both the aryl and bipyridine ligands, identifying activation mode as an additional parameter for controlling Ni-mediated C–F functionalization. Finally, direct access to (MeCN)_2_Ni(ArF_*n*−1_)_2_ (5) from Ni(cod)_2_ and PyF_5_ provides a modular entry point to Ni(ii) diaryl complexes, including those bearing ligands that do not support direct C–F activation from Ni(0). Collectively, these results establish a complementary approach to well-defined Ni(ii) diaryl complexes from parent polyfluoroarenes without requiring preformed perfluoroaryl organometallic reagents.

## Author contributions

M. S. B.: conceptualization, methodology, formal analysis, investigation, validation, data curation, writing – original draft, and visualization. J. O. R.: conceptualization, methodology, formal analysis, investigation, and writing – original draft. A. V.: investigation and validation. C. M. S.: investigation and writing – review & editing. S. P. D.: methodology and writing – review & editing. R. G. H.: conceptualization, writing – original draft, supervision, funding acquisition, and project administration.

## Conflicts of interest

There are no conflicts to declare.

## Supplementary Material

DT-OLF-D6DT02433E-s001

## Data Availability

Data supporting this article have been included as part of the supplementary information (SI). Supplementary information: experimental methods, computational methods, NMR spectra, and UV-vis absorption spectra. See DOI: https://doi.org/10.1039/d6dt02433e. CCDC 2552391 (3a), 2552382 (3b), 2552392 (3c), 2552394 (3d), 2552389 (3e), 2552396 (3f), 2552393 (3h), 2552397 (3i), 2552395 (3k), 2552385 (3l), 2552387 (4a), 2552384 (4b), 2552390 (4c), 2552388 (4d), and 2552386 (5) contain the supplementary crystallographic data for this paper.^84*a*–*o*^ Raw data files are available from the corresponding author upon reasonable request.
