# Supplementary material for: Direct access to chemodivergent Ni(ii) diaryl complexes via C–F activation of aryl fluorides
Source: Dalton Trans. 2026 Oct 28. Online ahead of print. doi: 10.1039/d6dt02433e (PMC13746360; doi:10.1039/d6dt02433e)
Supplement: Supplementary file 1 [file DT-OLF-D6DT02433E-s001.pdf]

Supplementary Information (SI) for Dalton Transactions.
This journal is © The Royal Society of Chemistry

Supporting Information

Direct Access to Chemodivergent Ni(II) Diaryl Complexes via C–F Activation of Aryl Fluorides

María S. Blankemeyer[a], Jacob O. Rothbaum[a], Annalissa Valdez[a,b], Camille M. Schubert[a], Stephen P. DiLuzio[a], and Ryan G. Hadt[a,*]

[a]Division of Chemistry and Chemical Engineering, Arthur Amos Noyes Laboratory of Chemical Physics, California Institute of Technology, Pasadena, CA 91125, United States

[b]Department of Chemistry and Biochemistry, California State University, Los Angeles, Los Angeles, CA 90032, United States.

*Corresponding Author: rghadt@caltech.edu

Table of Contents

1 General methods 3

2 Reaction optimization 4

2.1 Potential byproducts 6

3 Synthesis & characterization of nickel complexes 8

3.1 ($^{t\text{-Bu}}$bpy)Ni(ArF_{n-1})$_2$ complexes 8

3.2 *cis*–$(MeCN)_2Ni(PyF_4)_2$ complex 51

3.3 (^{R}bpy)Ni(PyF_4)$_2$ complexes 55

4 Thermal experiments 66

4.1 Post-reaction analysis of the hydrogen- and nickel-containing products 75

5 Photochemical experiments 76

6 Computational Methods 81

6.1 General Computational Details 81

6.2 Calculated Electronic Energies 82

6.3 Spin-State Analysis 82

6.4 Balanced Reaction-Energy Analysis 83

6.5 Cartesian Coordinates of Optimized Structures 84

7 Single crystal X-ray diffraction 101

8 References 118

1 General methods

All purchased compounds were used as received unless otherwise noted. Bis-(1,5-cyclooctadiene) nickel(0) was purchased from Strem Chemicals. Ligands 4,4′-dimethoxy-2,2′-bipyridine (^{MeO}bpy), 4,4′-di-tert-butyl-2,2′-bipyridine ($^{t\text{-Bu}}$bpy), 2,2′-bipyridine (bpy), dimethyl-2,2′-bipyridine-4,4′-dicarboxylate (^{MeOOC}bpy), and 4,4′-trifluoromethyl-2,2′-bipyridine (^{CF_3}bpy) were purchased from Sigma-Aldrich or Strem Chemicals. Aryl fluoride compounds were obtained from Sigma-Aldrich, CombiBlocks, Ambeed, TCI, or Thermo Fisher Scientific and used without further purification. Solids were dried under vacuum and brought into a nitrogen-atmosphere glove box; liquids were sparged (N_2), degassed via freeze-pump-thaw techniques, brought into the glove box, and stored over activated 3 Å molecular sieves. All solvents were air-free and collected from the solvent purification system (SPS), then stored in the glove box over activated 3 Å molecular sieves. Tetrahydrofuran (THF) was inhibitor-free. All synthesized compounds were made using air-free Schlenk techniques or made in a glove box. UV-vis spectra of the complexes were obtained on an Agilent Cary 5000 UV-Vis-NIR spectrophotometer using Starna Cells 9-SOG or 6-Q 10 mm path length cuvettes. Aside from DMSO-d_6, deuterated solvents were dried and stored over activated 3 Å molecular sieves in a nitrogen-filled glove box. Proton nuclear magnetic resonance (^{1}H NMR) and fluorine nuclear magnetic resonance (^{19}F NMR) spectra were recorded on a 400 MHz Varian Spectrometer with broadband auto-tune OneProbe. ^{19}F NMR spectra were ^{13}C decoupled. All quantitative ^{19}F NMR spectra utilized a 25s delay time, over 7 times the measured T_1 of complex **3a**. All ^{13}C NMR spectra were collected on a Bruker AV-III HD 400 MHz spectrometer. ^{13}C NMR spectra were ^{1}H decoupled. For ^{13}C NMR, some ArF_n carbons on substrates are poorly resolved due to extensive ^{13}C–^{19}F coupling. Chemical shifts are reported in parts per million (δ in ppm, s: singlet, d: doublet, t: triplet, m: multiplet) and are referenced to residual solvent signal (CD_3CN = δ 1.97 ppm, CD_2Cl_2 = δ 5.32 ppm, DMSO-d_6 = 2.50 ppm). Electrospray ionization mass spectrometry (ESI-MS) data were acquired on a Thermo Scientific LTQ linear ion trap mass spectrometer equipped with an Accela LC system and PAL autosampler and operated in positive-ion mode.

2 Reaction optimization

The initial reaction between ($^{t\text{-Bu}}$bpy)Ni(0)(cod) (formed *in situ* from $Ni(cod)_2$ (**1**) and 4,4′-di-tert-butyl-2,2′-dipyridyl ($^{t\text{-Bu}}$bpy)) and one equivalent of pentafluoropyridine (PyF_5, **2**) in THF afforded a translucent yellow-green solution that rapidly became cloudy, with precipitation of the bright-yellow Ni(II) products ($^{t\text{-Bu}}$bpy)Ni(II)(*p*-PyF_4)$_2$ (**3a**) and ($^{t\text{-Bu}}$bpy)Ni(II)(*o*-PyF_4)$_2$ (**3a′**).

Throughout this section, *p*-PyF_4 and *o*-PyF_4 denote PyF_4 groups formed by C–F activation para (C4) and ortho (C2 or C6) to the pyridine nitrogen, respectively. The ^{19}F NMR spectra of the reaction mixture showed peaks at δ = –99.6 and –123.3 ppm, consistent with the *ortho* and *meta* fluorine environments of **3a**, respectively (**Figure S6**). The *ortho* product **3a′** exhibited four characteristic peaks at δ = –86.7, –134.1, –149.4, and –172.1.[1,2] No characteristic Ni–F signal (–300 to –400 ppm) was observed. Complex **3a** was definitively identified by SC-XRD (*vide infra*).

To a tared oven-dried dram vial, $Ni(cod)_2$ (**1**) (0.2 mmol, 1.0 eq.) and $^{t\text{-Bu}}$bpy (0.2 mmol, 1.0 eq.) were added. Enough of the chosen solvent was added to the vial to achieve the desired concentration (**Y** mM in **Table S1**). The sample solution was allowed to stir for at least 30 minutes, after which **X** equivalents of PyF_5 was added. The reaction was allowed to stir for 18 hours at room temperature. The solvent was then removed under vacuum, and the sides of the sample vial were scraped until a fine homogeneous powder was obtained. A precise mass sample of the resulting powder was then weighed in a new vial. To this vial, 660 µL of CD_2Cl_2 was added and the solution was transferred to an NMR tube. To the NMR tube 1 µL of internal standard (1,2-difluorobenzene) was added. Reaction byproducts are discussed in section 2.1. Product quantities were determined by integration relative to the internal standard, with correction for the number of fluorine nuclei represented by each resonance. Yields were calculated relative to the theoretical maximum amount of diaryl product permitted by the initial quantities of nickel and ArF_n, recognizing that formation of one equivalent of $Ni(ArF_{n-1})_2$ requires two equivalents of ArF_n. Accordingly, the theoretical maximum product amount was defined as the smaller of n_0(Ni) and $n_0(ArF_n)/2$. Under the standard 1:1 Ni/ArF_n conditions, a reported 100% yield therefore corresponds to incorporation of 50% of the initial nickel into the diaryl product.

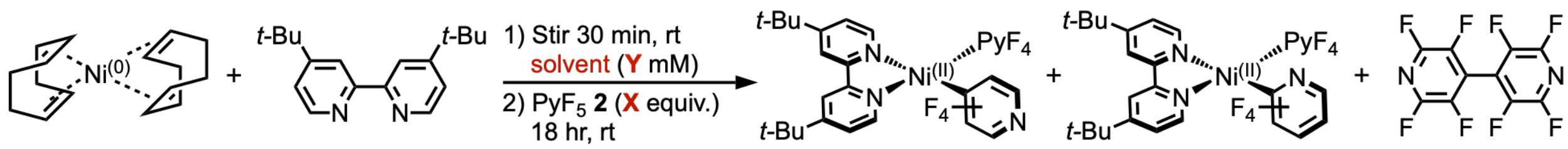

Table S1. Reaction optimization for formation of 3a.

entry	solvent	X (equiv.)	Y (mM)	yield of **3a**/%	yield of **3a′**/%	yield of **2′**/%
1	C_6H_6	1.0	100	18	65	*n.d.*
2	PhMe	1.0	100	27	43	*n.d.*
3	Et_2O	1.0	100	23	64	*n.d.*
4	2-MeTHF	1.0	100	23	43	*n.d.*
5	THF	1.0	100	33	19	*n.d.*
6	MeCN	1.0	100	34	*n.d.*	*n.d.*
7	MeCN	10.0	100	63	*n.d.*	15
8	MeCN	5.0	100	38	*n.d.*	12
9	MeCN	2.0	100	29	*n.d.*	5
10	MeCN	0.6	100	6	*n.d.*	*n.d.*
11	MeCN	0.5	100	*n.d.*	*n.d.*	*n.d.*

12	MeCN	1.0	200	21	*n.d.*	6
13	MeCN	1.0	50	62	*n.d.*	3
14	MeCN	1.0	25	82	*n.d.*	*n.d.*
15	MeCN	1.0	15	94	*n.d.*	*n.d.*
16	MeCN	1.0	5	87	*n.d.*	*n.d.*

Yields were determined by ^{19}F NMR spectroscopy with 1,2-difluorobenzene as an internal standard. *n.d.* = not detected.

To understand the factors governing formation of **3a**, we systematically examined the effects of solvent, concentration, and substrate stoichiometry on the reaction between Ni(0) and **2**, with all reactions performed at room temperature in an N_2-filled glovebox.

Solvent identity exerted a profound influence on both yield and regioselectivity (**Table S1**, **entries 1**–**6**). In non-polar, non-coordinating solvents such as benzene and toluene ($\varepsilon \approx 2.3$–2.4), *ortho* C–F activation predominates to form **3a′**, with the *para* product **3a** comprising only 22% and 39% of the total C–F activated product yield, respectively (**Table S1**, **entries 1**–**2**). Use of the weakly coordinating ethereal solvents Et_2O and 2-MeTHF ($\varepsilon \approx 4$–7) changes the product distribution but does not invert the preference for *ortho* C–F activation (**Table S1, entries 3–4**).The absence of a monotonic trend with dielectric constant indicates that polarity alone does not dictate selectivity. A qualitative shift occurs in THF ($\varepsilon \approx 7.6$), a stronger donor solvent capable of more effectively perturbing Ni(0) speciation. In THF, regioselectivity inverts, favoring *para*-functionalization (**Table S1**, **entry 5**). Most notably, MeCN ($\varepsilon \approx 36$), a strongly coordinating nitrile solvent, delivers exclusive *para*-selective C–F activation (**Table S1**, **entry 6**), representing a major enhancement in *para*-selectivity relative to benzene and a complete reversal of the inherent *ortho* preference historically observed in Ni C–F activation of **2**.

Because dielectric constant alone does not account for the observed trend in regioselectivity, we hypothesize that solvent coordination serves as a potential outer-sphere control element. Coordinating solvents such as THF and MeCN may facilitate cod displacement or stabilize distinct Ni(0)–arene pre-equilibria, thereby biasing the geometry preceding C–F activation.[3,4] These observations suggest that solvent coordination influences the speciation of reactive Ni(0) intermediates prior to C–F activation. This pronounced solvent sensitivity contrasts with σ-donor NHC-supported Ni(0) systems, where regioselectivity is largely invariant across hydrocarbon and ethereal solvents.[5]

The influence of substrate stoichiometry was next evaluated. Increasing equivalents of **2** enhanced formation of **3a** but also led to increased formation of the C–C homocoupled product **2′** (**Table S1, entry 7**). To suppress homocoupling, we evaluated reaction concentration in MeCN. Lowering the reaction concentration improved yields of **3a** while maintaining regioselectivity and minimizing biaryl formation (**Table S1, entries 12–16**). Performing the reaction in MeCN with only 1 equivalent of **2** at reduced concentration afforded **3a** in 94% yield (**Table S1, entry 15**). These findings align with Ni-mediated cross-electrophile coupling studies, where electron-deficient aryl halides perform optimally at controlled substrate concentrations, and excess substrate promotes homocoupled biaryl formation.[6–12]

Collectively, these results show that solvent identity, substrate concentration, and stoichiometry strongly influence regioselectivity and product distribution during formation of **3a**. Solvent coordination may alter cod displacement and Ni(0) speciation, whereas concentration and substrate loading modulate competition between productive C–F activation and biaryl formation. Although the available data do not isolate a single controlling factor, they demonstrate that

reaction conditions can be leveraged to improve *para*-selectivity and yield under additive-free, room-temperature conditions.

2.1 Potential byproducts

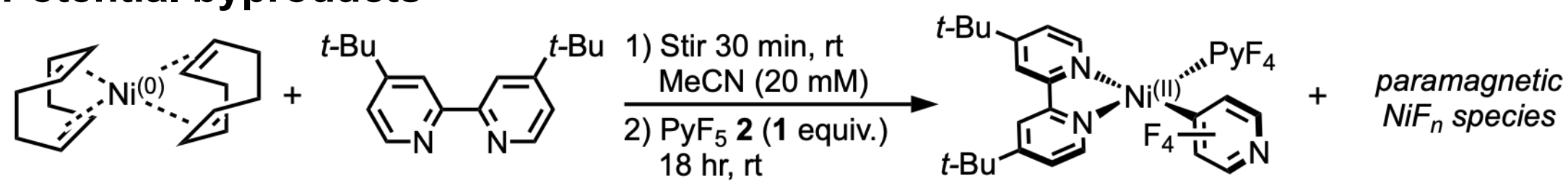

1 (1 equiv.) (1 equiv.) **3a**

NMR analysis of the reaction between ($^{t\text{-Bu}}$bpy)Ni(0)(cod) and pentafluoropyridine (**2**) revealed the formation of a paramagnetic nickel-containing byproduct in addition to the diamagnetic Ni(II) diaryl product **3a** (**Figure S1**). During isolation of **3a**, this byproduct appears as a green-yellow precipitate that can be removed through extensive washing with MeCN (see section 3.1). Definitive characterization of the byproduct proved challenging due to its paramagnetic nature, instability, and limited solubility in common organic solvents. Multiple attempts to isolate and crystallize the material under rigorously air- and moisture-free conditions were unsuccessful due to gradual decomposition.

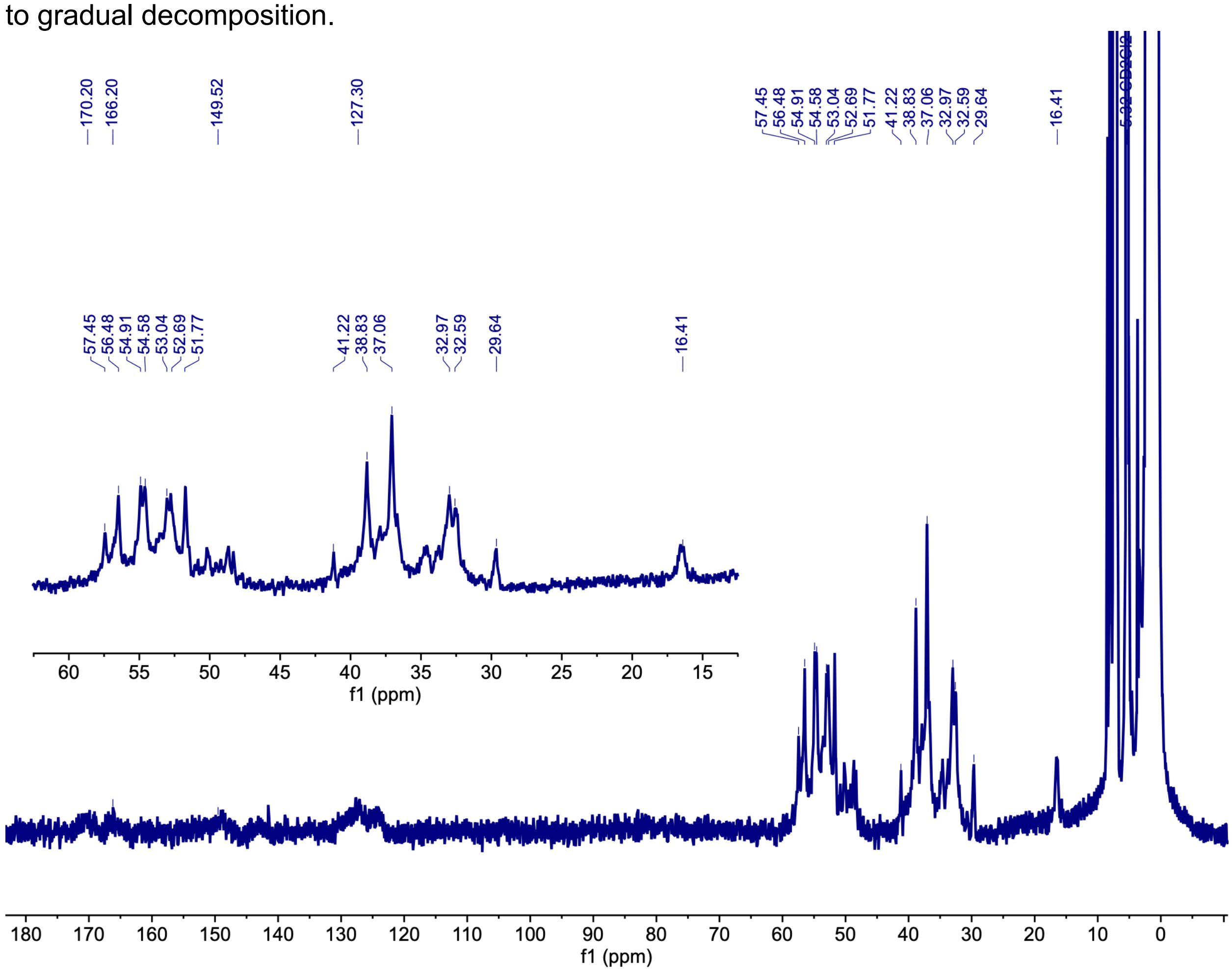

Figure S1. ^{1}H NMR (400 MHz, CD_2Cl_2) spectrum of the crude reaction for the formation of complex **3a**. A short relaxation delay (D1 = 0.3 s) and exponential line broadening (lb = 10 Hz) was applied to enhance visualization of the broad paramagnetic resonances.

The broad ^{1}H resonances observed between δ = 127–170, 51–57, and 29–41 ppm are reminiscent of previously reported paramagnetic bipyridine-supported nickel halide species,

particularly dimeric [($^{t\text{-Bu}}$bpy)Ni(I)X]$_2$ complexes, although *pseudo*-tetrahedral ($^{t\text{-Bu}}$bpy)Ni(II)X_2 species (X = Cl or Br) should also be considered.[13,14] While definitive assignment is not possible from the available spectroscopic data, the observed chemical shift range and line broadening are consistent with the presence of one or more paramagnetic nickel-containing species. Such species are also consistent with pathways proposed for Ni-mediated C–F activation in which Ni(II) aryl fluoride intermediates undergo ligand redistribution to form diaryl Ni(II) products and nickel-fluoride-containing coproducts.

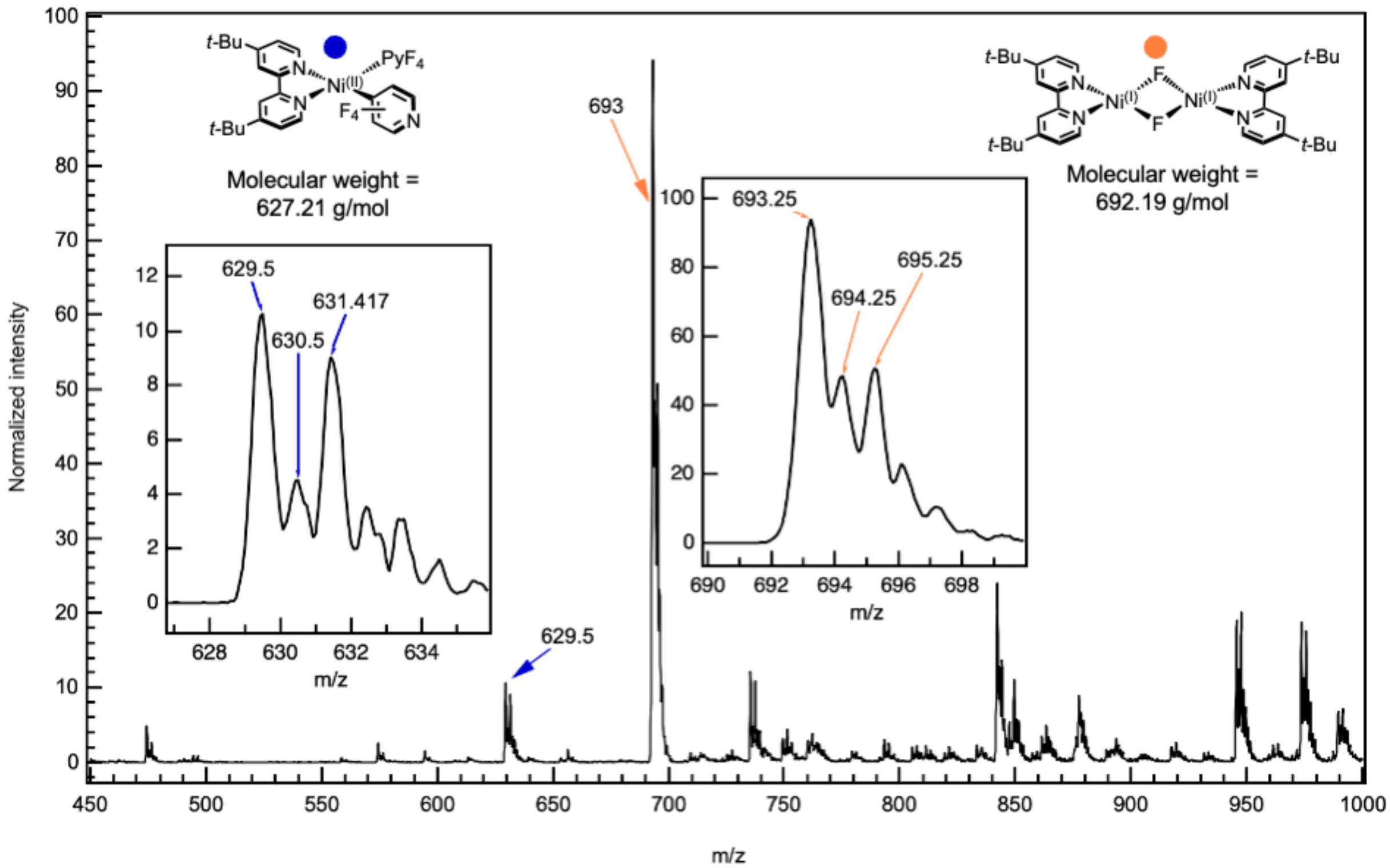

Figure S2. Positive-ion ESI mass spectrum of the crude reaction mixture obtained during formation of **3a** after redissolution in CH_2Cl_2.

To probe the fate of fluoride during C–F activation, the crude reaction mixture was analyzed by positive-ion ESI mass spectrometry. Signals at m/z 629.5 and 693.3 were observed, which are consistent with nickel fluoride-containing species generated during the reaction. The experimentally observed isotope distributions closely match those calculated for the proposed nickel-fluoride-containing ions (**Figure S2**).[15] These results support the formation of nickel-fluoride-containing species and are consistent with fluoride transfer to nickel during C–F activation, although they do not establish a unique coproduct structure.

Alternative fluoride sinks were also considered. In particular, fluorinated cod derivatives and fluorinated solvent products were evaluated as potential byproducts. However, no evidence for such species was observed in the substrate scope experiments performed in sealed J-Young NMR tubes, where crude reaction mixtures were analyzed directly by ^{19}F NMR spectroscopy prior to workup. While these possibilities cannot be completely excluded, the combined NMR, isolation behavior, and mass spectrometric data are most consistent with formation of paramagnetic nickel fluoride-containing byproducts. Although definitive structural characterization remains elusive, these observations provide a plausible destination for nickel and fluoride not incorporated into the isolated Ni(II) diaryl products.

3 Synthesis & characterization of nickel complexes

3.1 ($^{t\text{-Bu}}$bpy)Ni(ArF_{n-1})$_2$ complexes

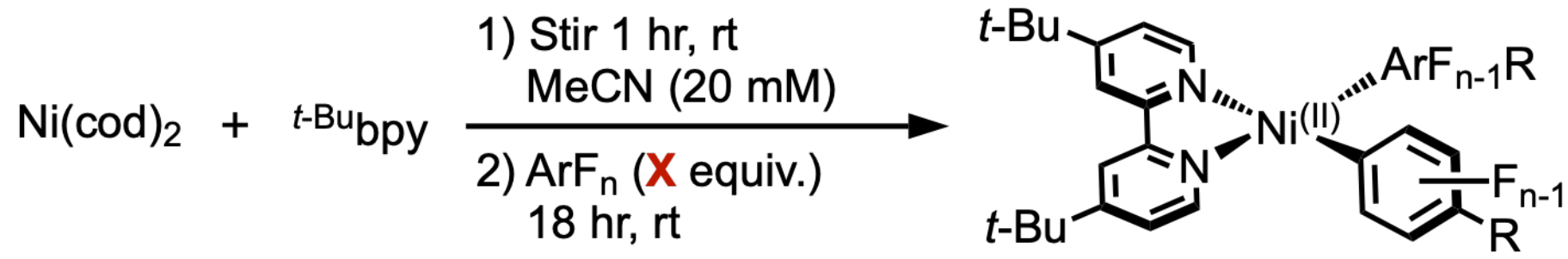

Method A: In a nitrogen-filled glove box, an oven-dried 20 mL scintillation vial was charged with a Teflon-coated stir bar, Ni(cod)$_2$ (0.30 mmol, 1 eq.), and $^{t\text{-Bu}}$bpy (0.30 mmol, 1.0 eq.). To this vial, 15 mL of MeCN was added (20 mM), and the mixture was stirred for one hour affording a deep purple solution. Subsequently, 1 or more eq. of ArF_n was added, affording a color change. More than 1 equiv. of ArF_n was added where reactivity was observed to be slow (**3e**, **3f**, **3h**). The appearance of the reaction mixture varied with substrate. Reactions forming **3a**, **3d**, or **3k** initially produced translucent yellow solutions that gradually became opaque as the products precipitated over several hours, whereas the more sluggish reactions forming **3e** or **3f** remained darker amber-orange solutions.

When a precipitate was present after 18 h (**3a**, **3d**, **3k**, and **3l**), the reaction mixture was concentrated to approximately 2 mL. The solid was collected by filtration through a glass frit, washed sequentially with pentane (3 × 5 mL), MeCN (1 × 1 mL), and pentane (3 × 5 mL), and then dried under vacuum for several hours before weighing.

When the product did not precipitate (**3b** and **3f**), the reaction mixture was removed from the glovebox and concentrated under vacuum to give a dark oil. Pentane (approximately 10 mL) was added, and the mixture was stirred vigorously for 30 min while the oil was periodically scraped with a spatula to induce formation of a light-colored powder. The solid was collected by filtration through a glass frit, washed with pentane (3 × 5 mL), and dried under vacuum for several hours before weighing.

Complexes prepared utilizing method A will be denoted by superscript *a* following the yield. Product yield was calculated relative to the theoretical maximum product amount permitted by the limiting reagent, accounting for incorporation of two ArF_n-derived groups per diaryl complex.

Method B: In a nitrogen-filled glove box, a 20 mL scintillation vial was charged with a Teflon-coated stir bar, Ni(cod)$_2$ (9.6 mg, 0.035 mmol, 1 eq.), and $^{t\text{-Bu}}$bpy (9.4 mg, 0.035 mmol, 1.0 eq.). To this vial, CD_3CN (3.5 mL, 10 mM) was added. After allowing the solution to stir for 1 hour, a 650 µL aliquot of the ($^{t\text{-Bu}}$bpy)Ni(0)(cod) stock solution was transferred to a J-Young NMR tube and the ArF_n (0.0065 mmol, 1.0 eq.) was added directly to the NMR tube. Progress of the reaction was monitored by ^{19}F NMR spectroscopy using 1,4-difluorobenzene (DFB) as an internal standard (1.00 µL, 1.5 eq.) and product yield was determined 24 hrs after adding ArF_n. The product conversion was calculated from the ratio of the integrals of the DFB and the product Ni(ArF_{n-1})$_2$.

For solid substrates, decafluorobiphenyl and octafluoronaphthalene, a 10 mM stock solution was prepared in CH_2Cl_2. An aliquot (650 µL, 0.0065 mmol, 1.0 equiv.) was transferred to a J-Young NMR tube, and the CH_2Cl_2 was removed under vacuum. A 650 µL aliquot of the 10 mM ($^{t\text{-Bu}}$bpy)Ni(0)(cod) stock solution (0.0065 mmol, 1.0 equiv.) was then added to the tube.

Trifluorotoluene (1.5 eq., 1.20 µL) was used as an internal standard for the reaction with pentafluorostyrene, as the product peaks overlap with 1,4-difluorobenzene.

Complexes prepared utilizing method B will be denoted by superscript *b* following the yield. Product yield was calculated relative to the theoretical maximum product amount permitted by the limiting reagent, accounting for incorporation of two ArF_n-derived groups per diaryl complex.

($^{t\text{-Bu}}$bpy)Ni(*p*-PyF$_4$)$_2$ (3a): Yield of 56%[a]. Yield of 67%[b]. Bright yellow powder. ^{1}H NMR (400 MHz, CD_2Cl_2): δ 7.97 (s, 2H), 7.36 (m, 4H), 1.40 (s, 18H). ^{13}C NMR (101 MHz, CD_2Cl_2): δ 165.80, 155.38, 150.46, 147.12 – 144.04 (m), 142.01 – 141.12 (m), 143.34 – 140.62 (m), 124.83, 118.63, 36.02, 30.29. ^{19}F NMR (376 MHz, CD_2Cl_2): δ -99.46 – -99.80 (m, 4F), -123.17 – -123.52 (m, 4F). UV-Vis (THF): λ_{max} = 373 nm, ε(373 nm) = 3700 M^{-1} cm^{-1}.

($^{t\text{-Bu}}$bpy)Ni(*o*-PyF$_4$)$_2$ (3a′):^{19}F NMR (376 MHz, CD_2Cl_2): δ -86.69 (td, *J* = 28.0, 28.0, 16.0 Hz, 1F), -133.99 – -134.23 (m, 1F), -149.38 (dt, *J* = 28.0, 16.0 Hz, 1F), -172.11 (dd, *J* = 28.0, 16.0 Hz, 1F).

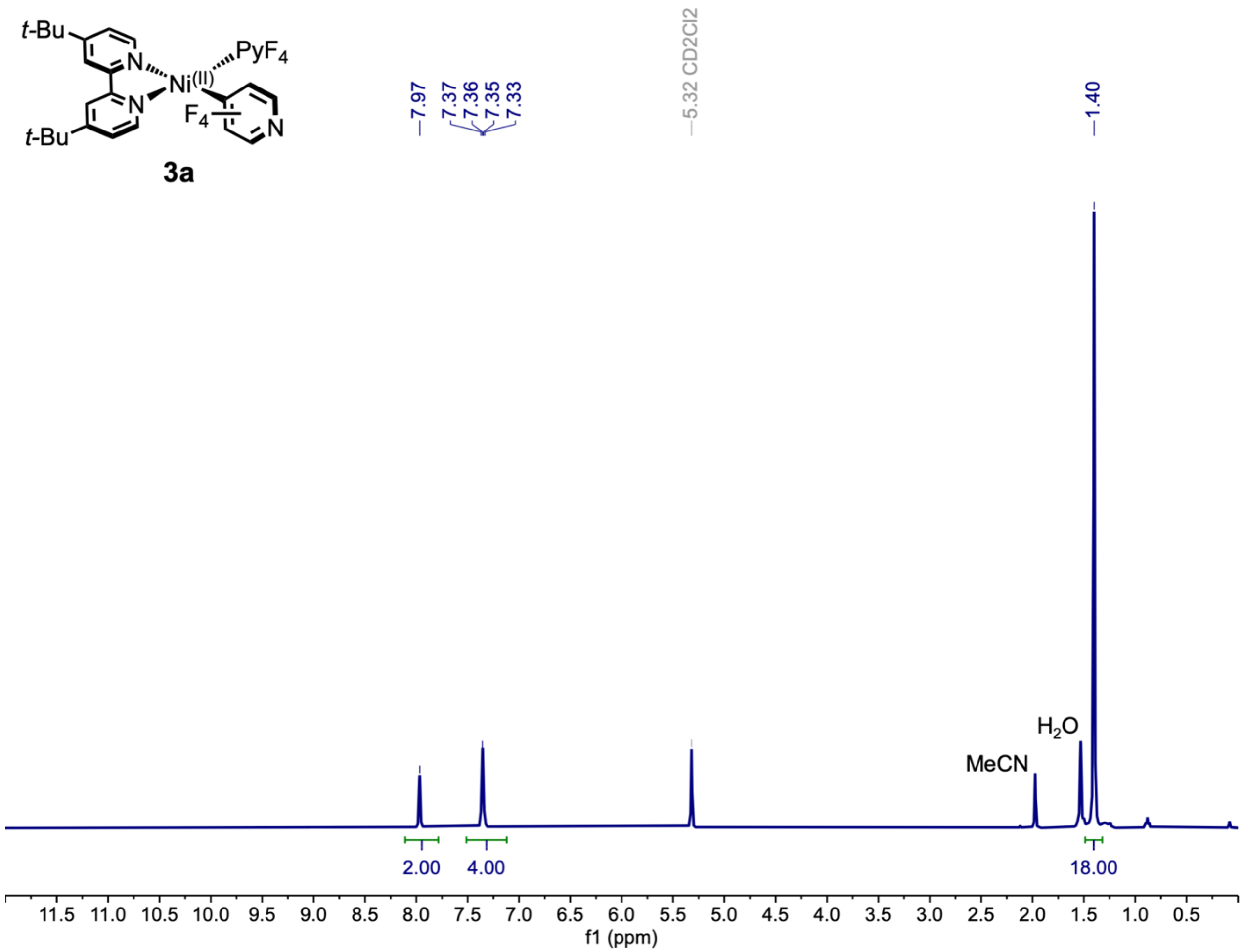

Figure S3. ^{1}H NMR (400 MHz, CD_2Cl_2) spectrum of complex **3a** prepared utilizing method A.

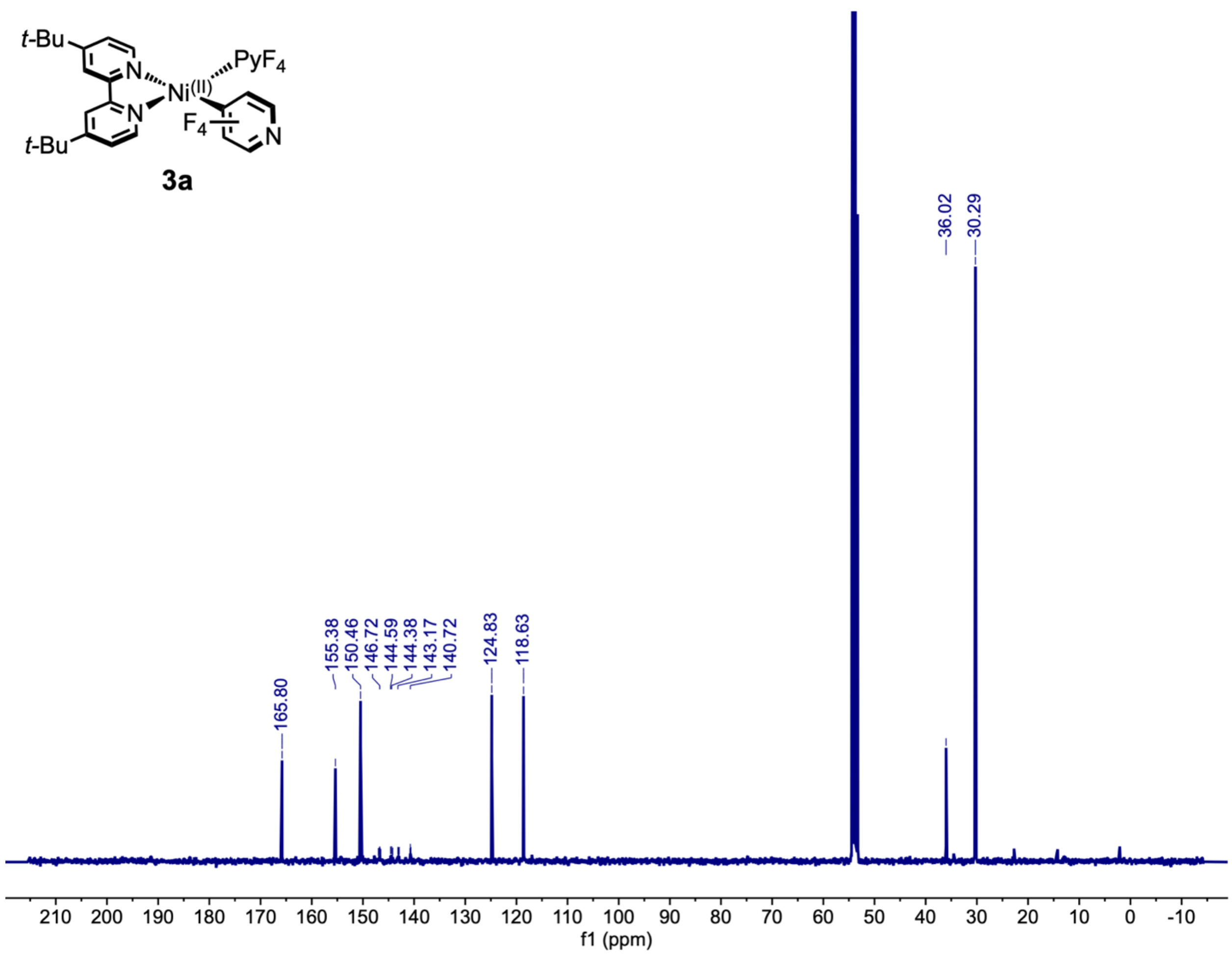

Figure S4. ^{13}C NMR (101 MHz, CD_2Cl_2) spectrum of complex **3a** prepared utilizing method A.

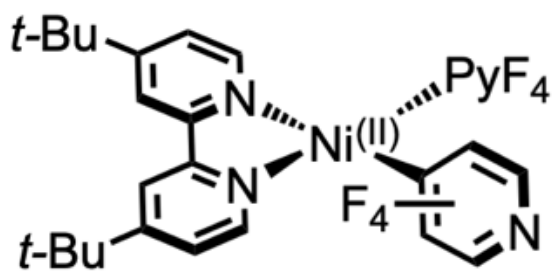

3a

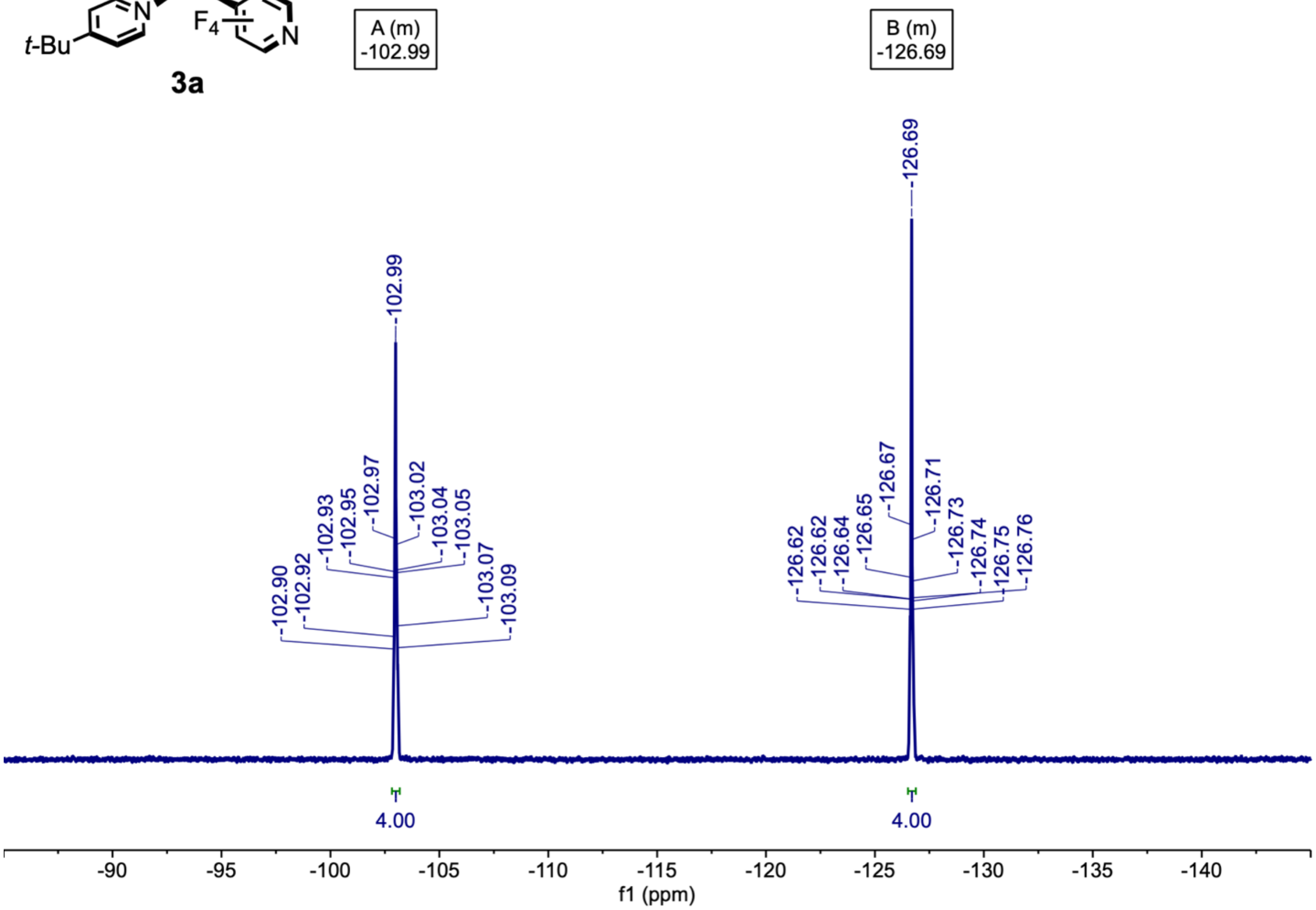

Figure S5. ^{19}F NMR (376 MHz, CD_2Cl_2) spectrum of complex **3a** prepared utilizing method A.

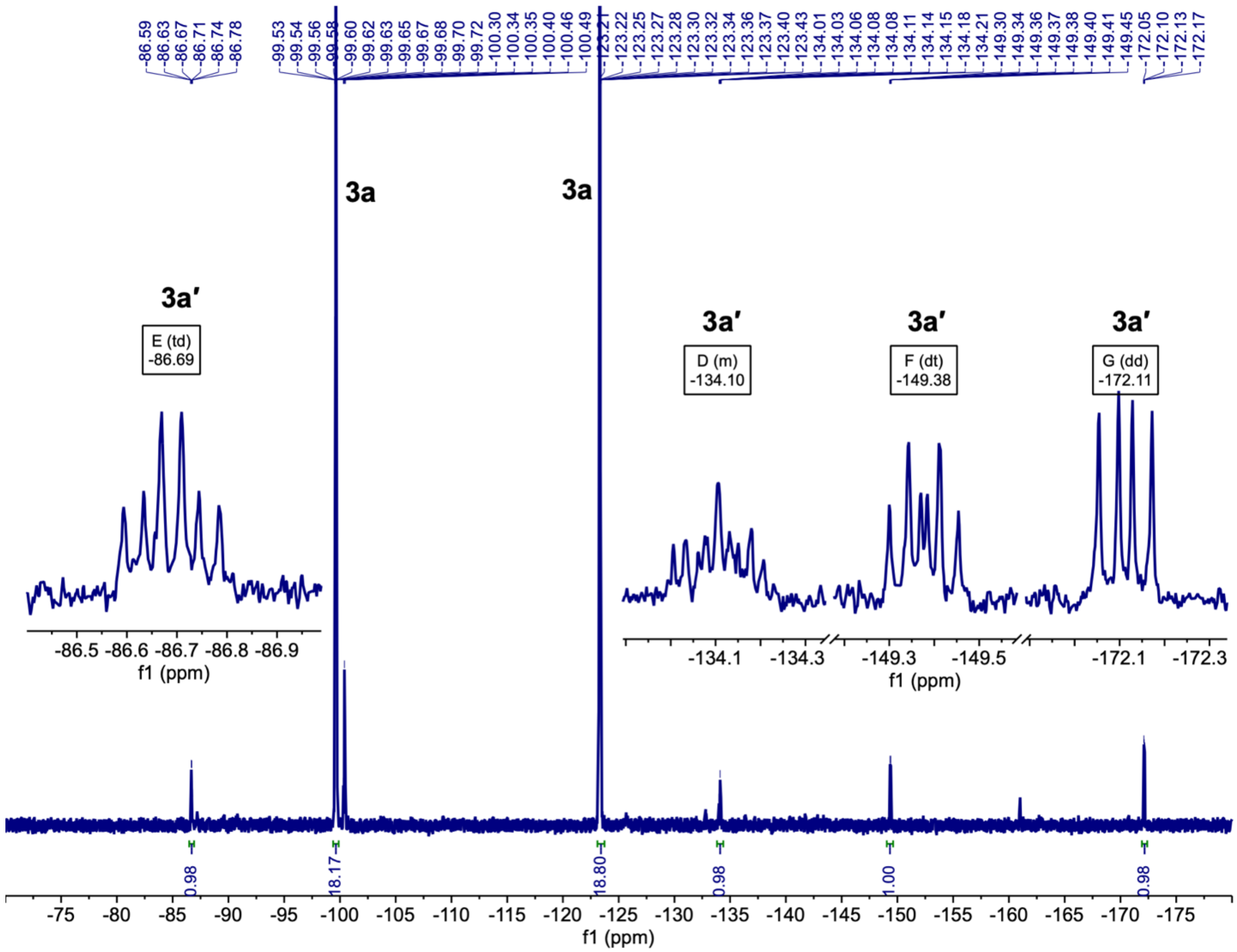

Figure S6. ^{19}F NMR (376 MHz, CD_2Cl_2) spectrum of complexes **3a** and **3a′** prepared utilizing method A in THF.

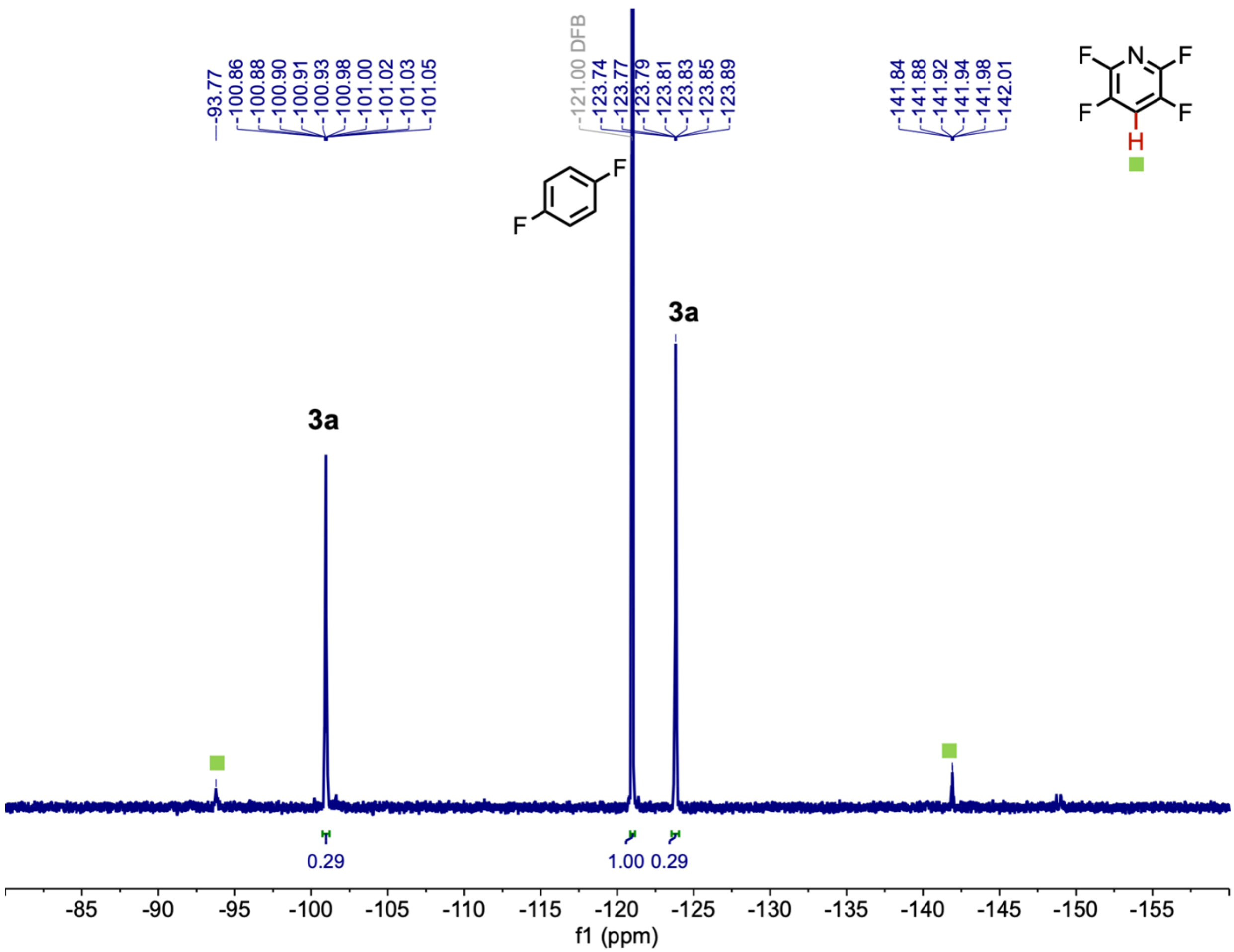

Figure S7. ^{19}F NMR (376 MHz, CD_3CN) spectrum of complex **3a** prepared utilizing method B.

($^{t\text{-Bu}}$bpy)Ni(C_6F_5)$_2$ (3b): Yield of 33%[a]. Yield of 54%[b]. Bright yellow powder. ^{1}H NMR (400 MHz, CD_2Cl_2): δ 7.93 (d, *J* = 2.0 Hz, 2H), 7.39 (d, *J* = 6.0 Hz, 2H), 7.32 (dd, *J* = 6.0, 2.0 Hz, 2H), 1.39 (s, 18H). ^{13}C NMR (101 MHz, CD_2Cl_2): δ 165.07, 155.35, 150.54, 146.95 (m), 137.10 (m), 124.55, 118.29, 35.92, 30.30. ^{19}F NMR (376 MHz, CD_2Cl_2): δ -117.64 (m, 4F), -161.89 (t, *J* = 19.7 Hz, 2F), -164.51 (m, 4F).

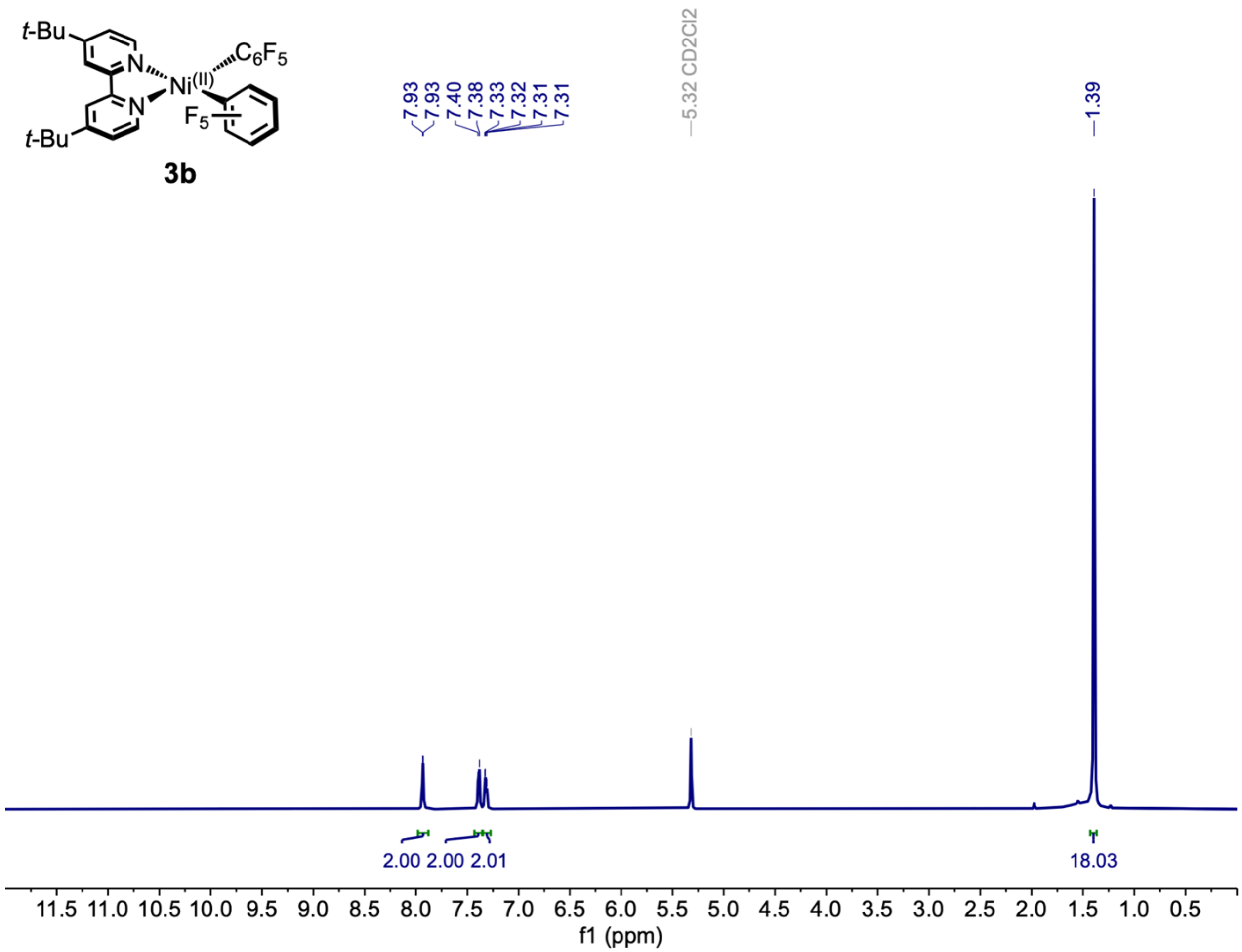

Figure S8. ^{1}H NMR (400 MHz, CD_2Cl_2) spectrum of complex **3b** prepared utilizing method A.

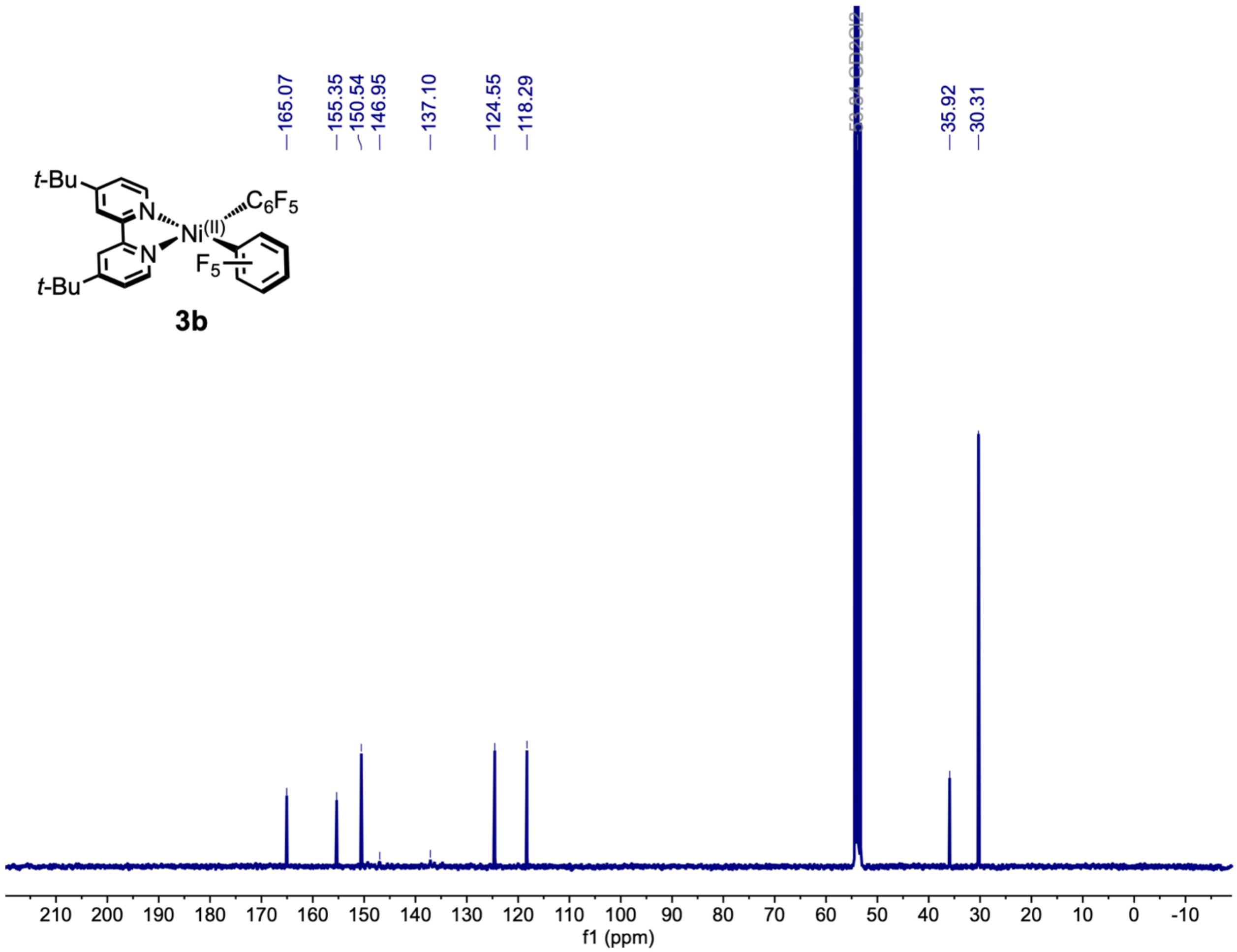

Figure S9. ^{13}C NMR (101 MHz, CD_2Cl_2) spectrum of complex **3b** prepared utilizing method A.

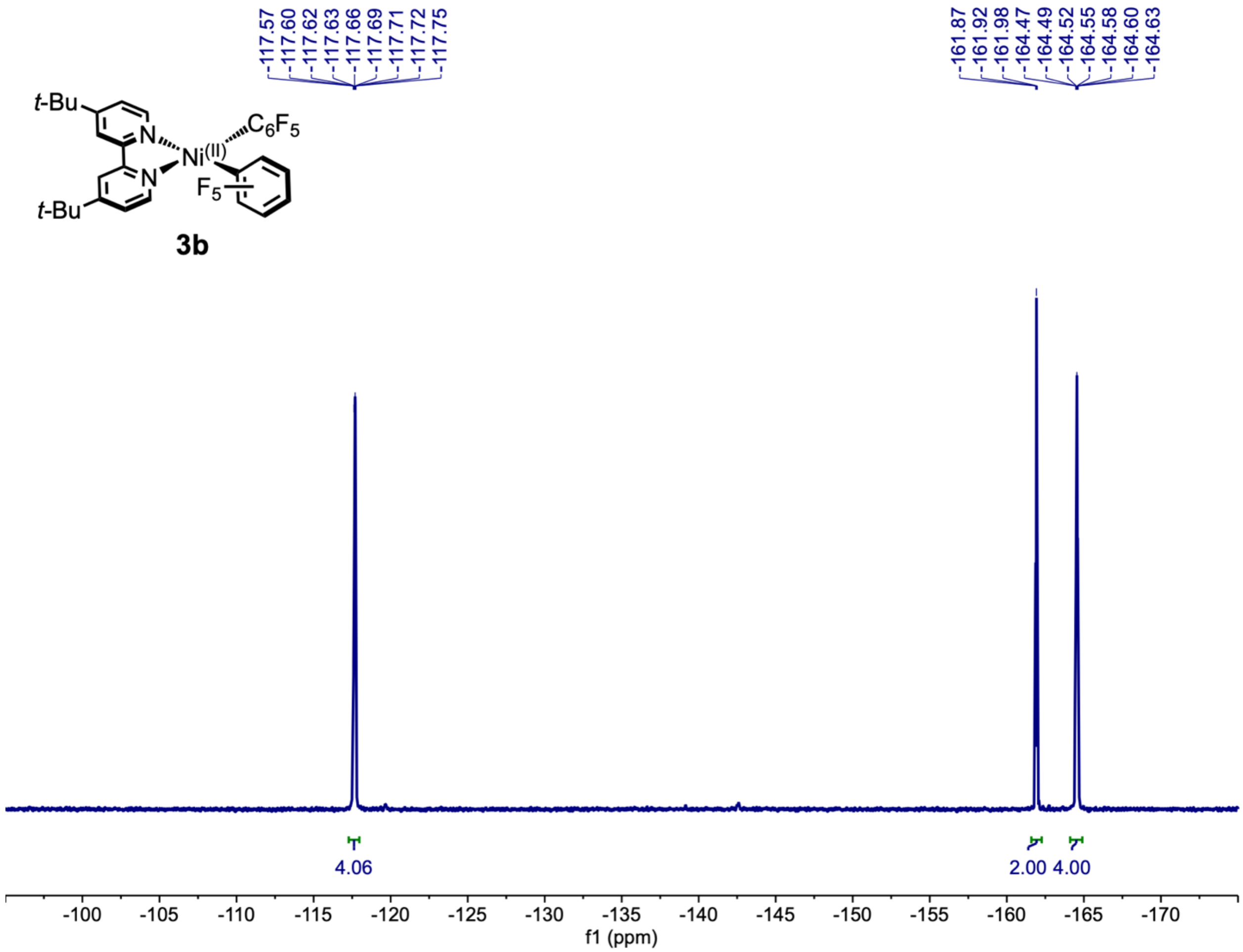

Figure S10. ^{19}F NMR (376 MHz, CD_2Cl_2) spectrum of complex **3b** prepared utilizing method A.

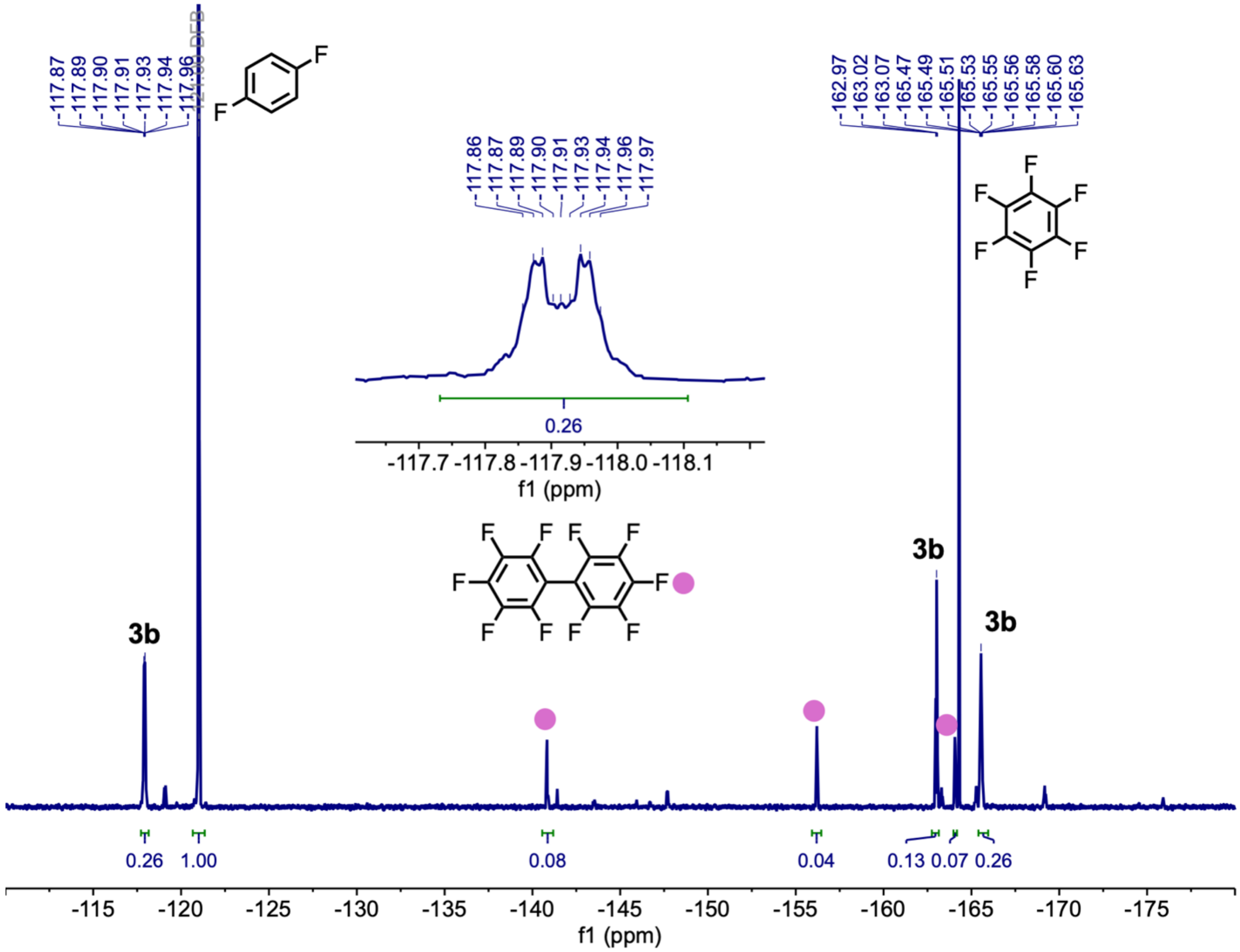

Figure S11. ^{19}F NMR (376 MHz, CD_3CN) spectrum of complex **3b** prepared utilizing method B.[16]

($^{t\text{-Bu}}$bpy)Ni($C_6F_4CF_3$)$_2$ (3c): Yield of 66%[b]. ^{1}H NMR (400 MHz, CD_3CN): δ 8.23 (s, 2H), 7.43 – 7.35 (m, 4H), 1.40 (s, 18H). ^{13}C NMR (101 MHz, CD_2Cl_2): δ 166.55, 155.97, 151.32, 125.15, 36.53, 30.23. ^{19}F NMR (376 MHz, CD_3CN): δ -56.37 (t, *J* = 21.5 Hz, 6F), -117.29 (ddt, *J* = 20.1, 14.8, 6.9 Hz, 4F), -145.93 – -146.17 (m, 4F).

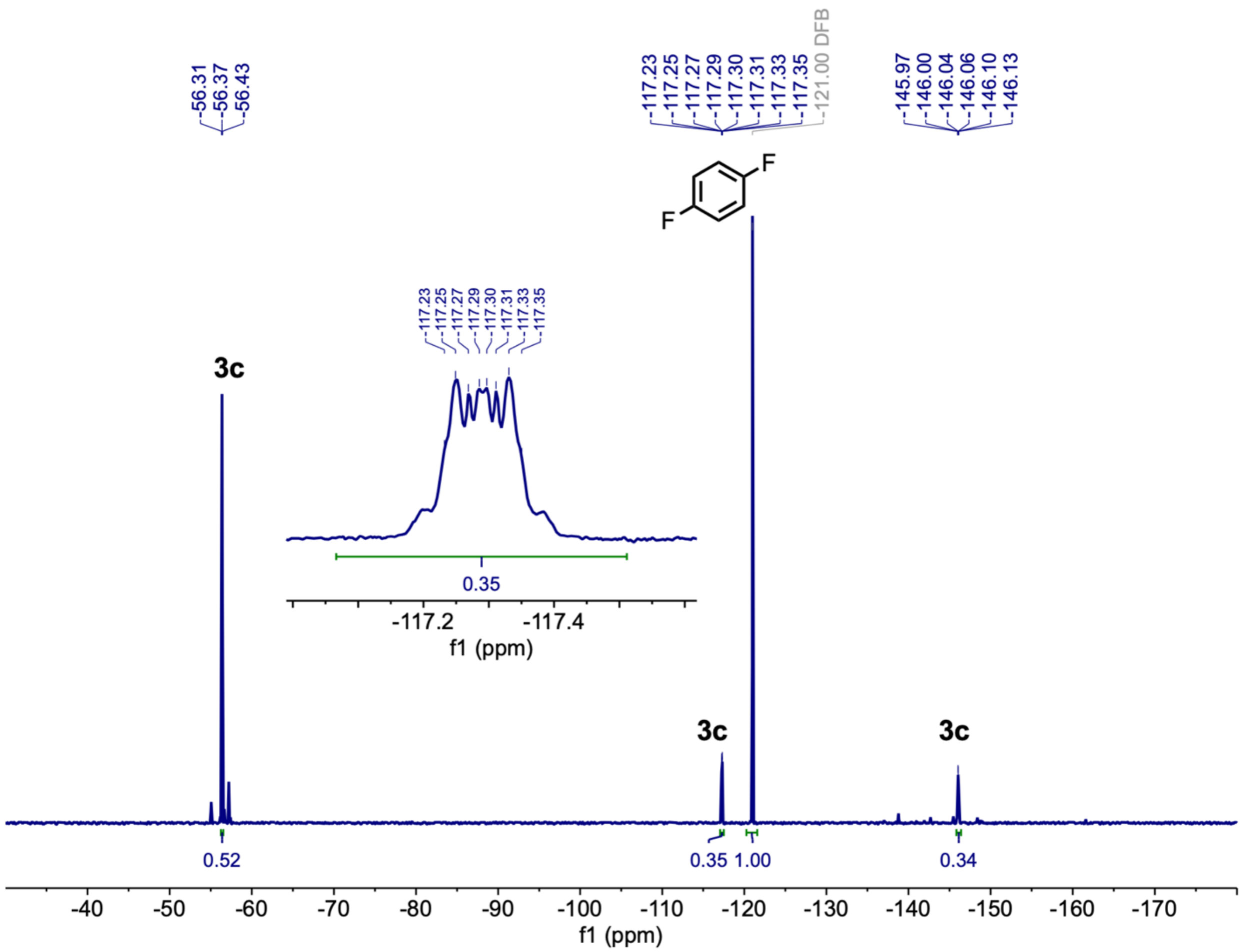

Figure S12. ^{19}F NMR (376 MHz, CD_3CN) spectrum of complex **3c** prepared utilizing method B.

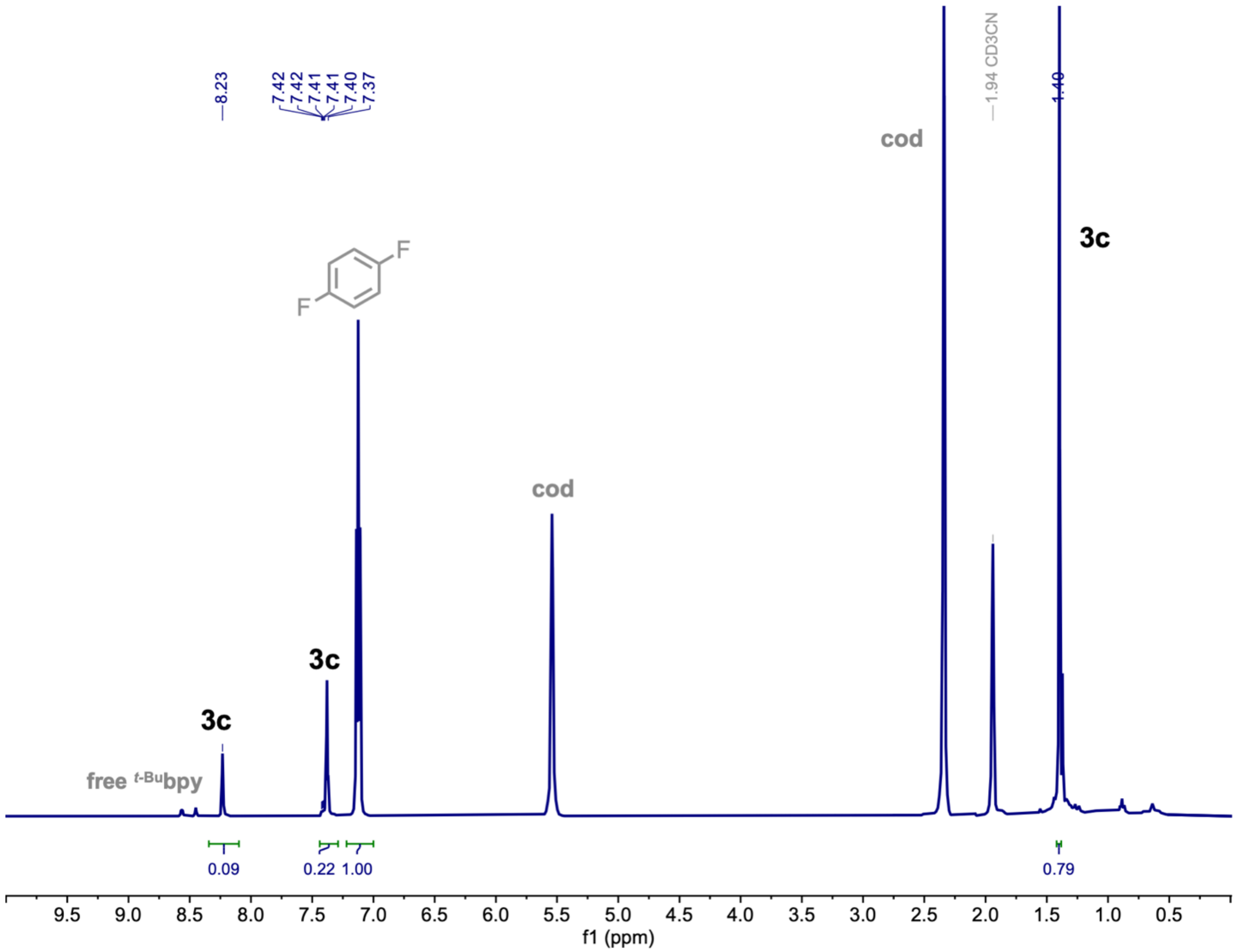

Figure S13. ^{1}H NMR (400 MHz, CD_3CN) spectrum of complex **3c** prepared utilizing method B.

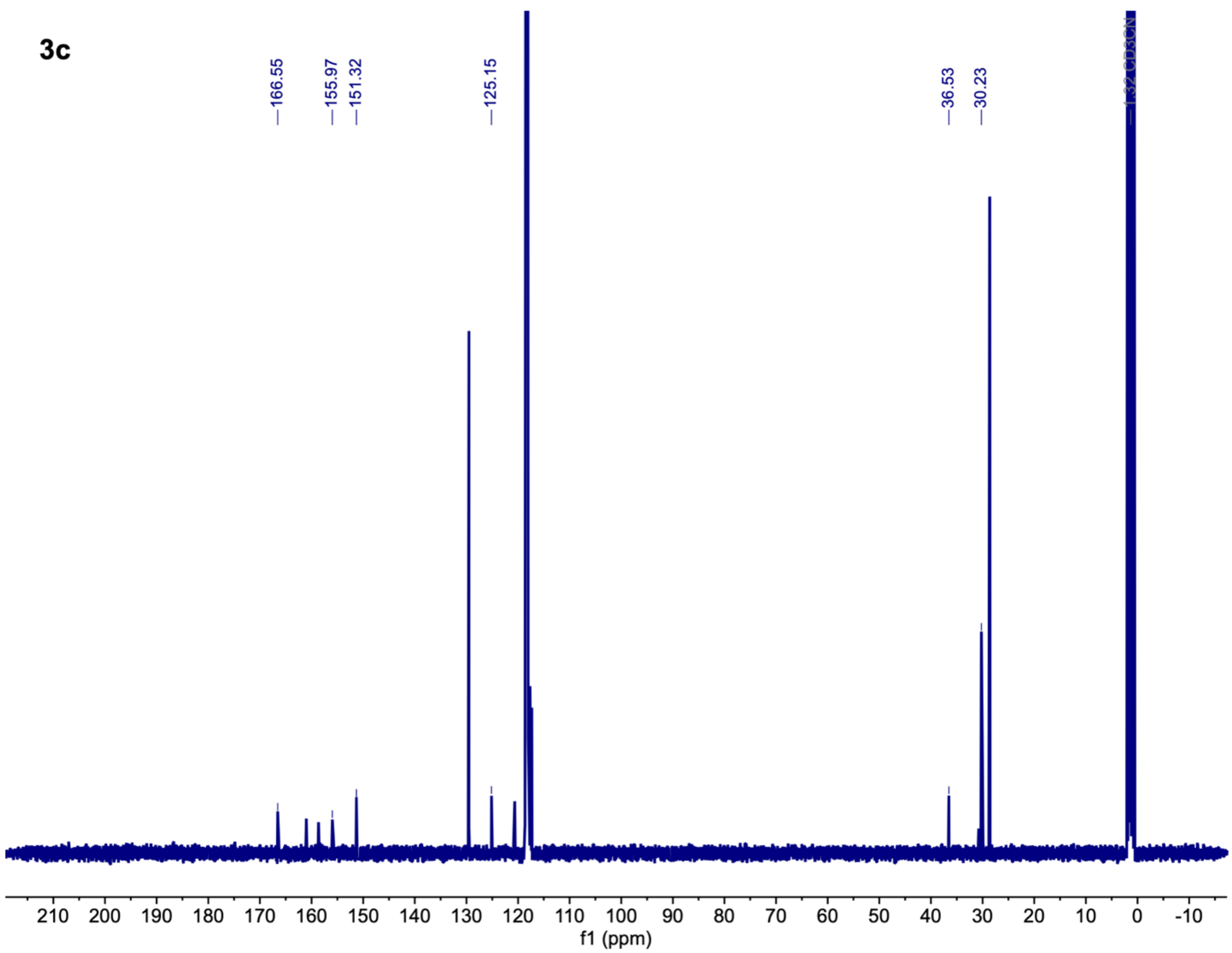

Figure S14. ^{13}C NMR (101 MHz, CD_3CN) spectrum of complex **3c** prepared utilizing method B.

($^{t\text{-Bu}}$bpy)Ni($C_6F_4CO_2Me$)$_2$ (3d): Yield of 42%[a]. Yield of 83%[b]. Bright yellow powder. ^{1}H NMR (400 MHz, CD_2Cl_2): δ 7.94 (d, *J* = 2.1 Hz, 2H), 7.39 (d, *J* = 6.0 Hz, 2H), 7.32 (dd, *J* = 6.0, 2.1 Hz, 2H), 3.89 (s, 6H), 1.39 (s, 18H). ^{13}C NMR (101 MHz, CD_2Cl_2): δ 165.29, 161.94, 155.35, 150.58, 147.96 (m), 144.43 (m), 141.85 (m), 124.67, 118.39, 107.88 (t, *J* = 15.2 Hz), 68.15, 35.94, 30.30. ^{19}F NMR (376 MHz, CD_2Cl_2): δ -117.77 – -118.11 (m, 4F), -143.01 – -143.30 (m, 4F).

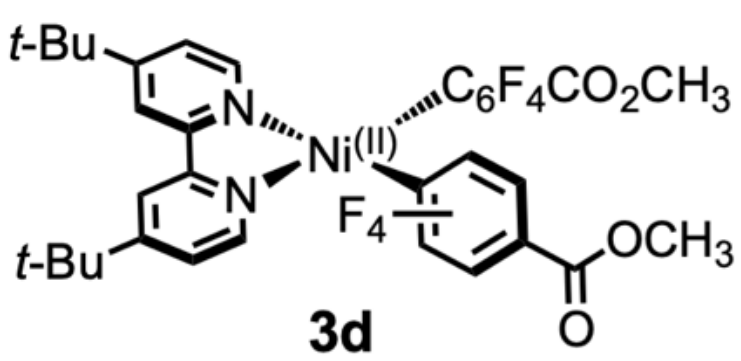

3d

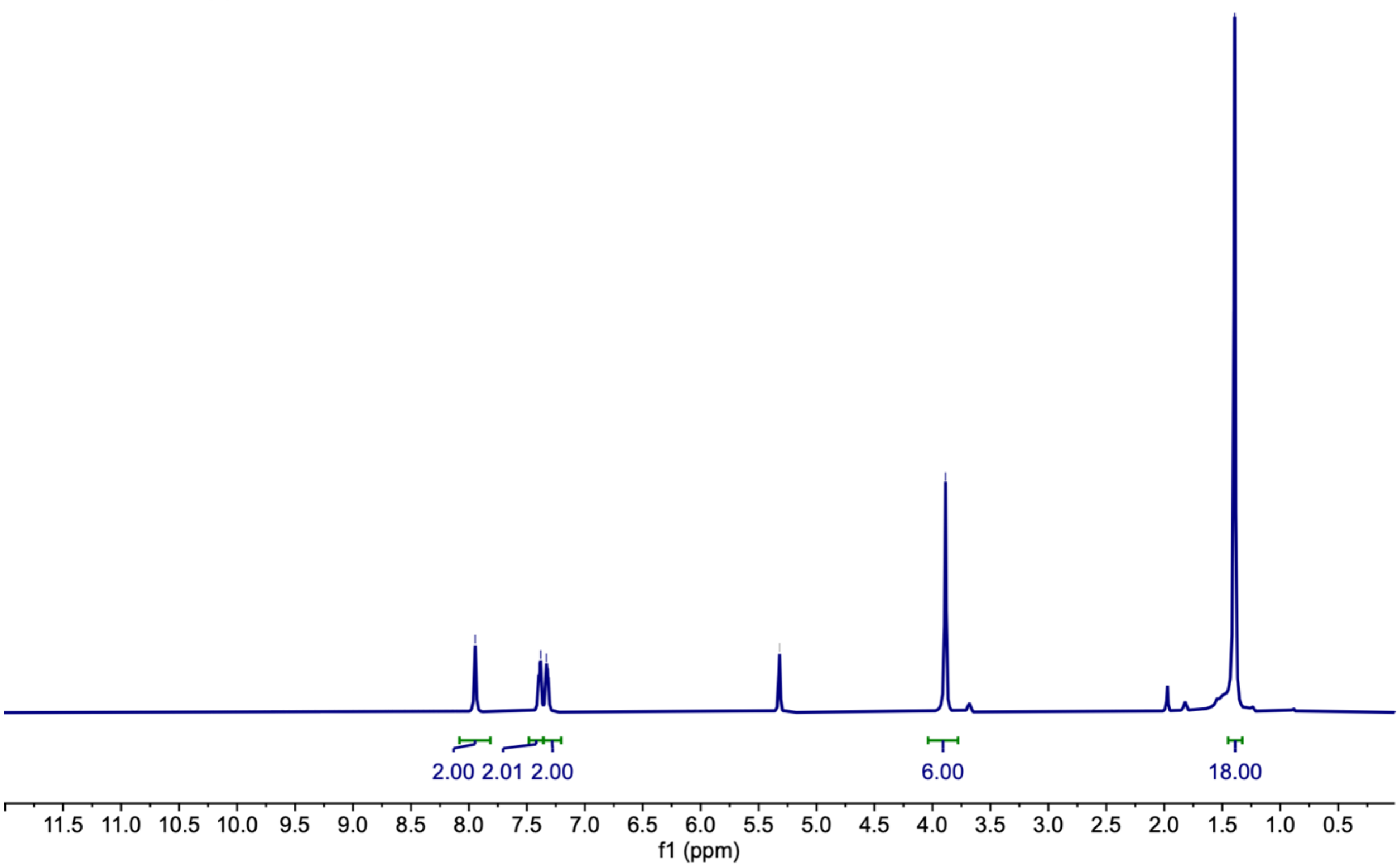

Figure S15. ^{1}H NMR (400 MHz, CD_2Cl_2) spectrum of complex **3d** prepared utilizing method A.

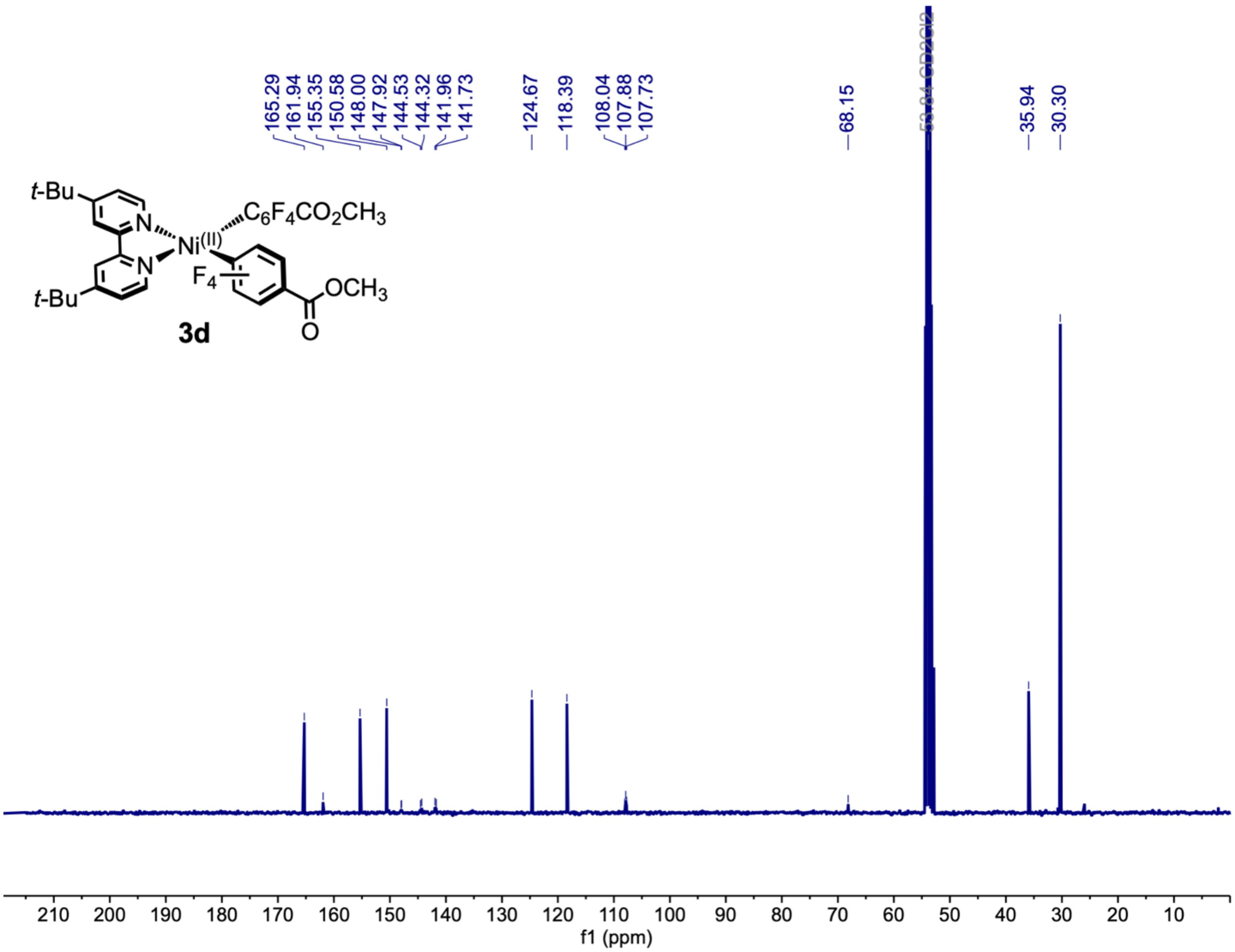

Figure S16. ^{13}C NMR (101 MHz, CD_2Cl_2) spectrum of complex **3d** prepared utilizing method A.

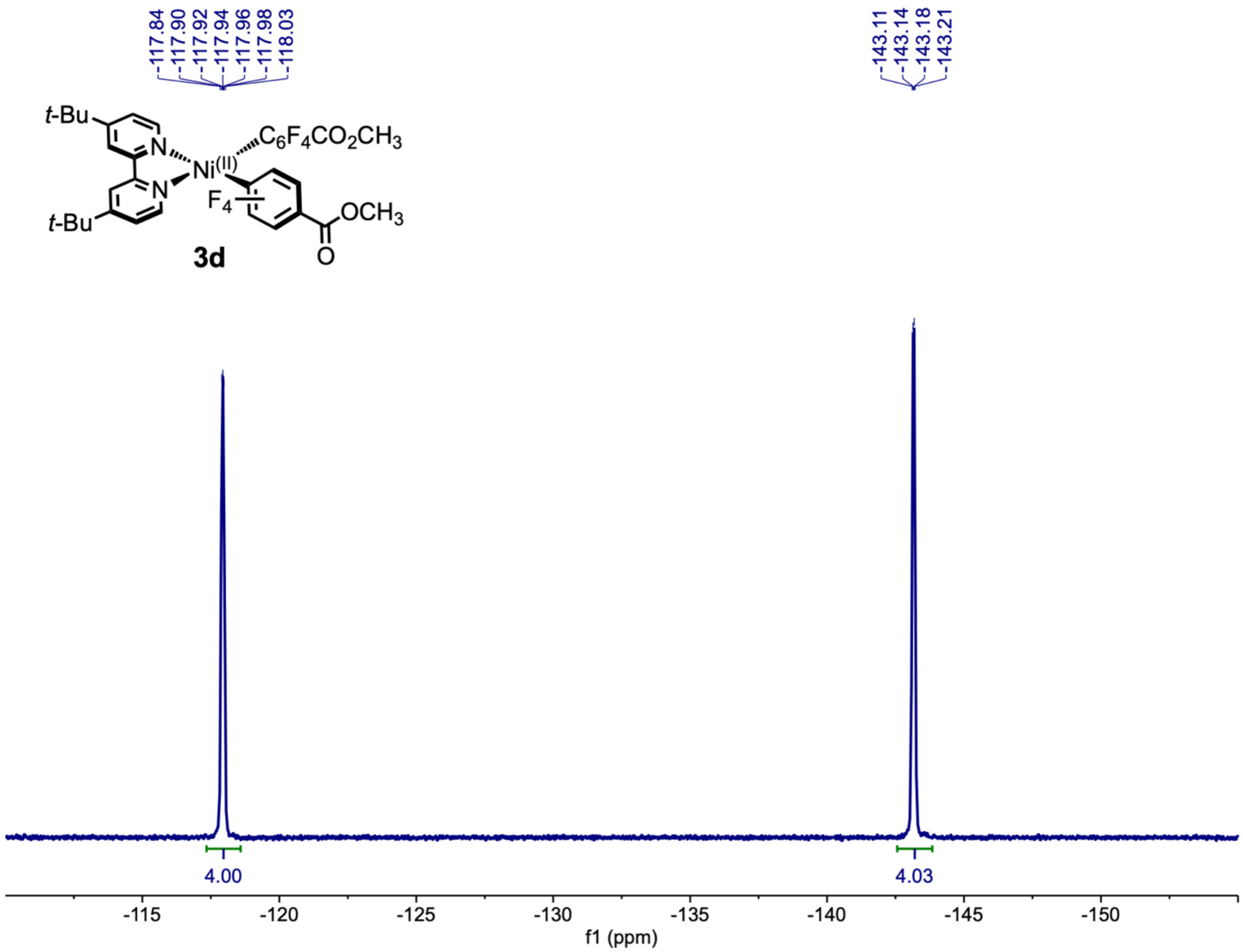

Figure S17. ^{19}F NMR (376 MHz, CD_2Cl_2) spectrum of complex **3d** prepared utilizing method A.

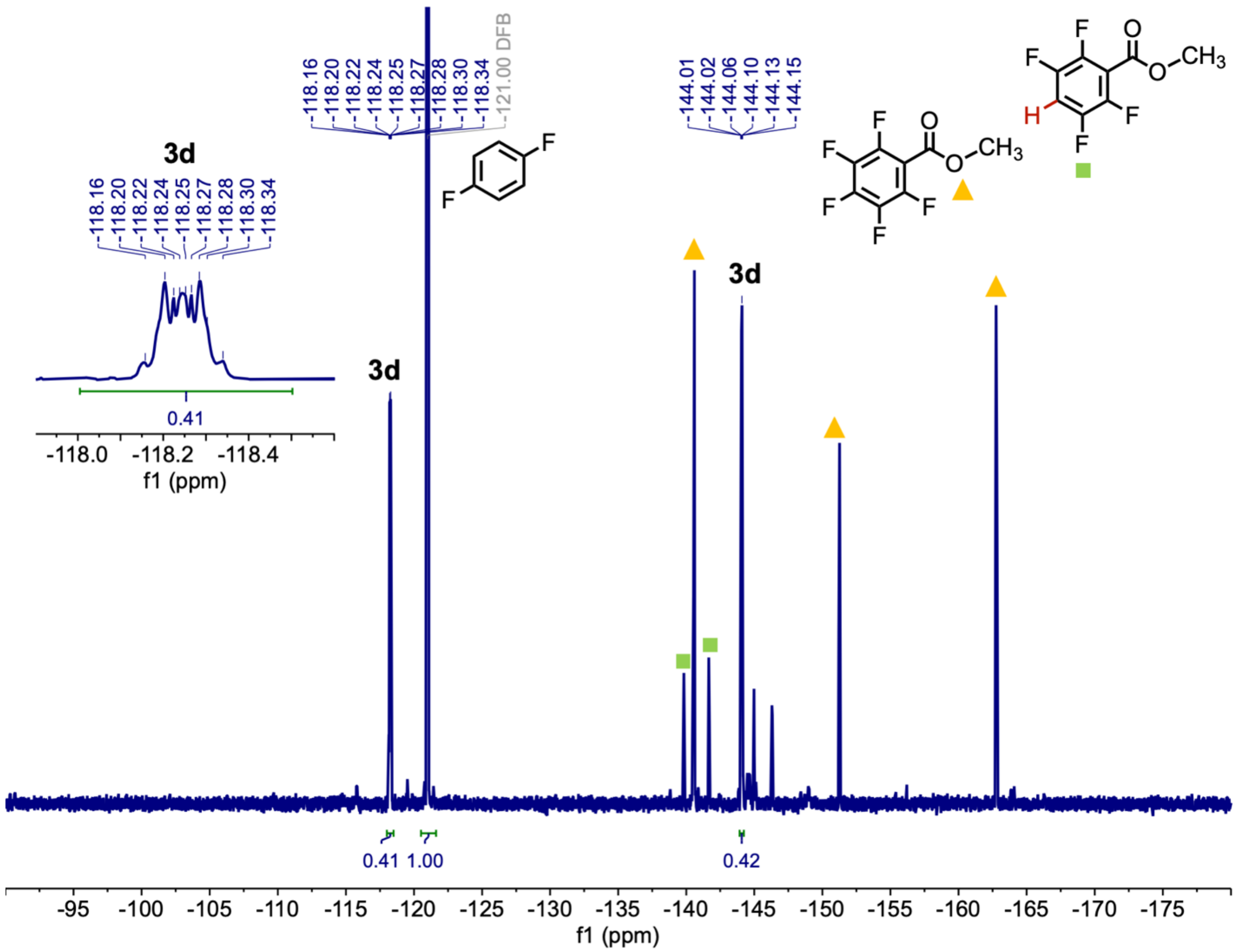

Figure S18. ^{19}F NMR (376 MHz, CD_3CN) spectrum of complex **3d** prepared utilizing method B.[17]

(${}^{t\text{-Bu}}$bpy)Ni(C_6F_4OMe)$_2$ (3e): Yield of 31%[b]. ^{1}H NMR (400 MHz, CD_3CN): δ 8.23 (m, 4H), 7.41 (s, 2H), 3.90 (s, 6H), 1.37 (s, 18H). ^{19}F NMR (376 MHz, CD_3CN): δ -119.32 (dd, *J* = 26.4, 6.5 Hz, 4F), -160.16 (d, *J* = 26.4 Hz, 4F).

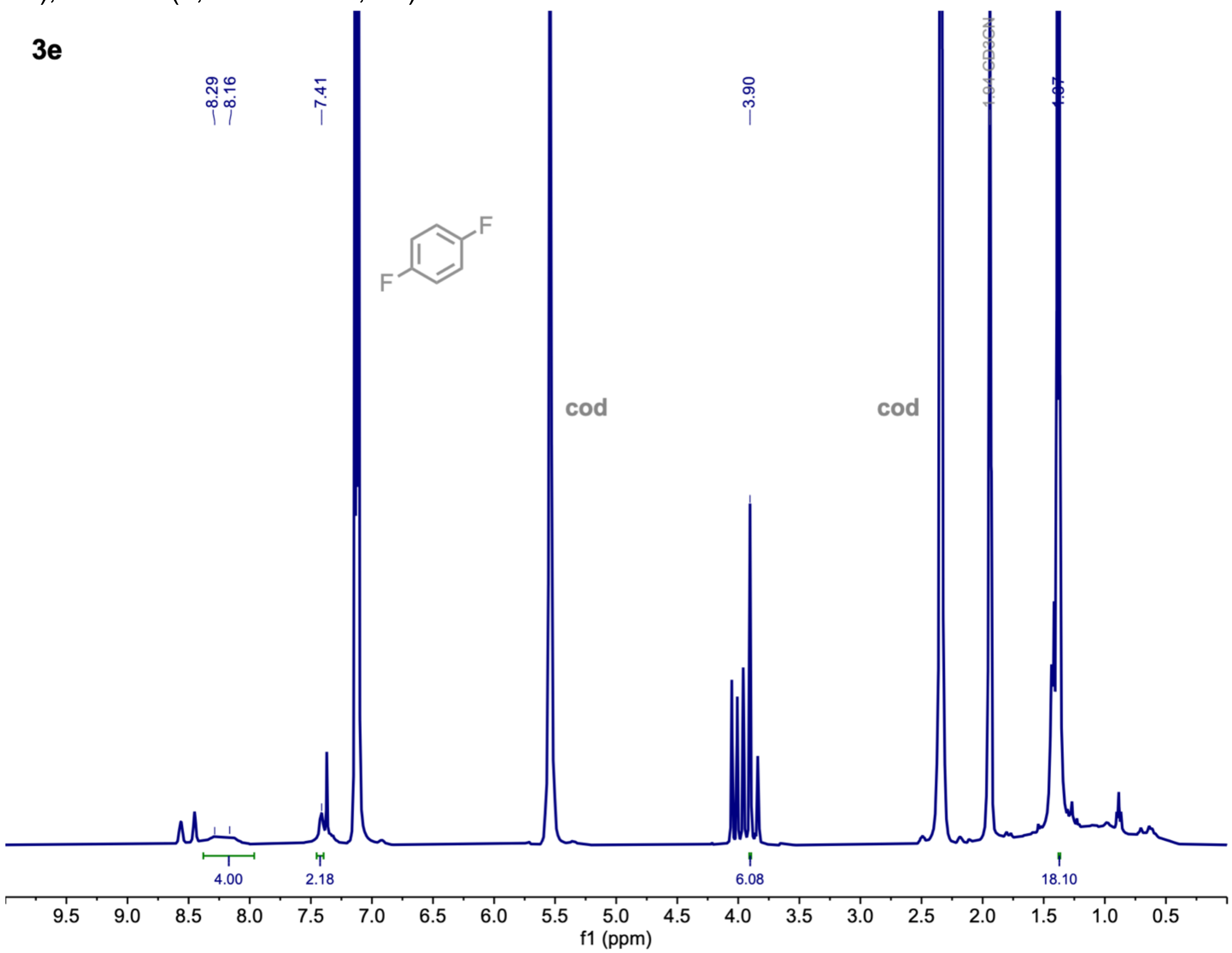

Figure S19. ^{1}H NMR (400 MHz, CD_3CN) spectrum of complex **3e** prepared utilizing method B.

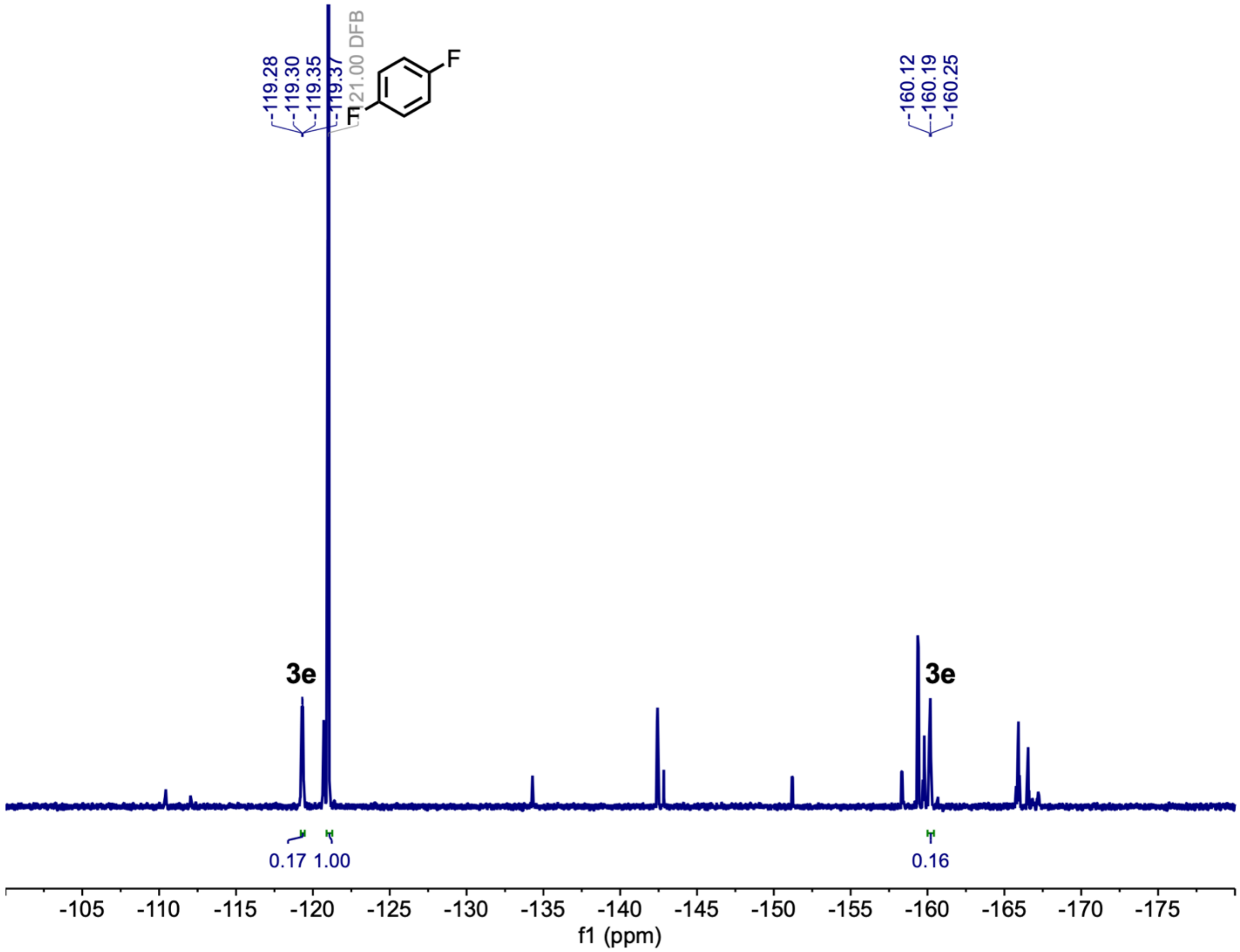

Figure S20. ${}^{19}F$ NMR (376 MHz, CD_3CN) spectrum of complex **3e** prepared utilizing method B.

($^{t\text{-Bu}}$bpy)Ni($C_6F_4CH_3$)$_2$ (3f): Yield of 43%[a]. Yield of 28%[b] (heated to 60 °C). Yellow-orange powder. ^{1}H NMR (400 MHz, CD_2Cl_2): δ 7.92 (d, *J* = 2.1 Hz, 2H), 7.45 (d, *J* = 6.0 Hz, 2H), 7.30 (dd, *J* = 6.0, 2.1 Hz, 2H), 2.14 (t, *J* = 1.9 Hz, 6H), 1.39 (s, 18H). ^{13}C NMR (101 MHz, CD_2Cl_2): δ 164.60, 155.32, 150.59, 124.41, 118.09, 110.54, 35.85, 30.31, 7.69. ^{19}F NMR (376 MHz, CD_2Cl_2): δ -120.28 – -120.58 (m, 4F), -146.92 – -147.17 (m, 4F).

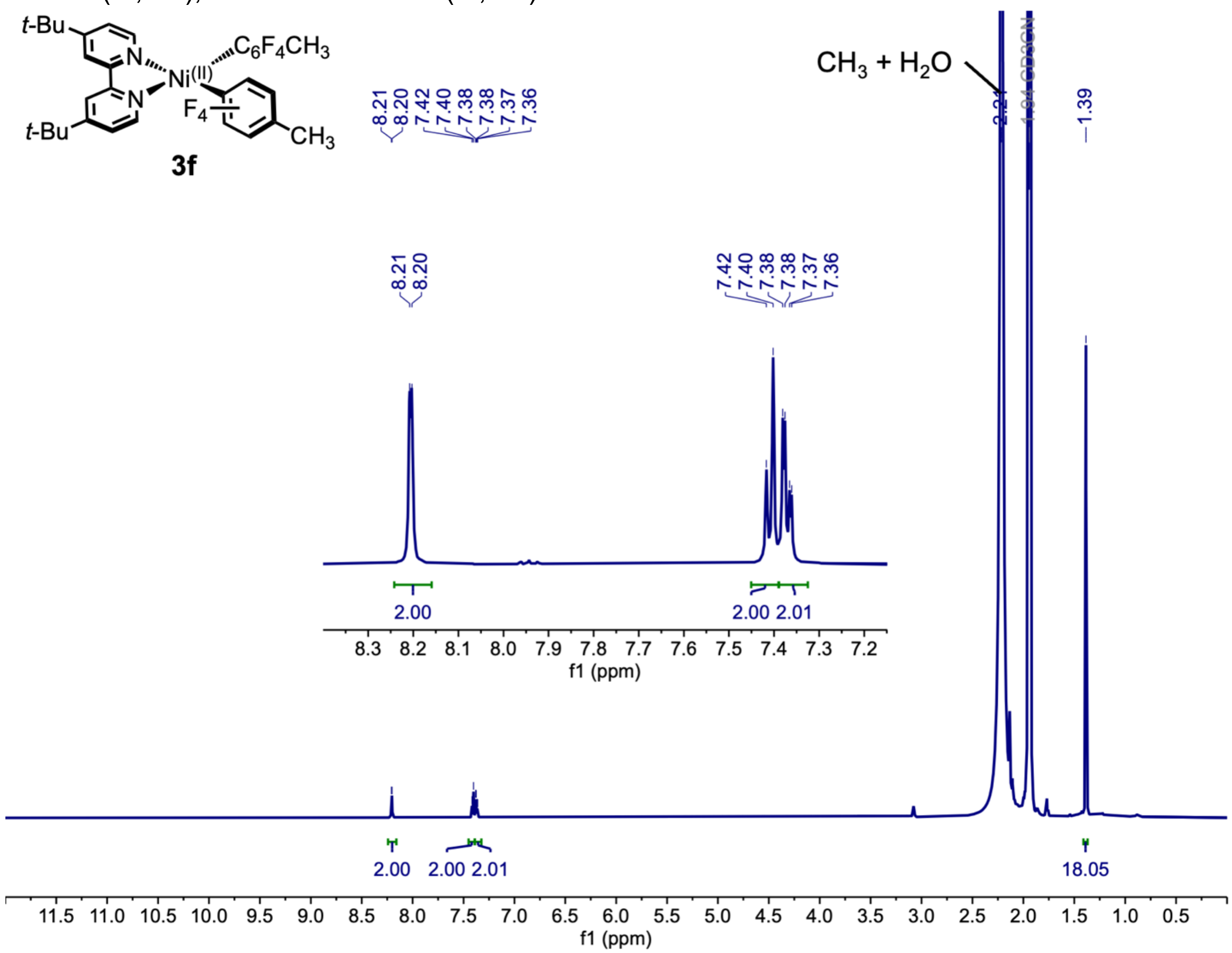

Figure S21. ^{1}H NMR (400 MHz, CD_2Cl_2) spectrum of complex **3f** prepared utilizing method A.

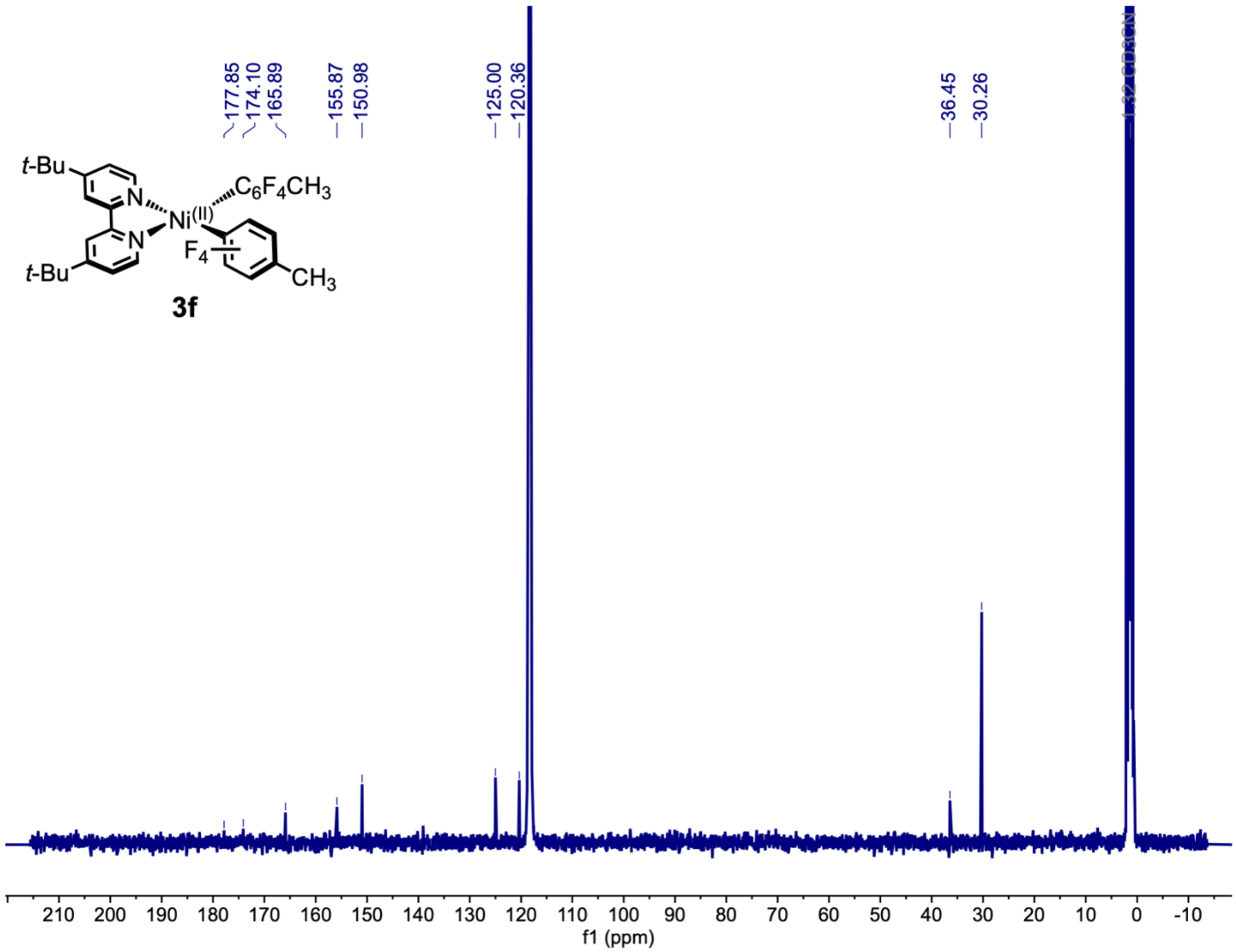

Figure S22. ^{13}C NMR (101 MHz, CD_2Cl_2) spectrum of complex **3f** prepared utilizing method A.

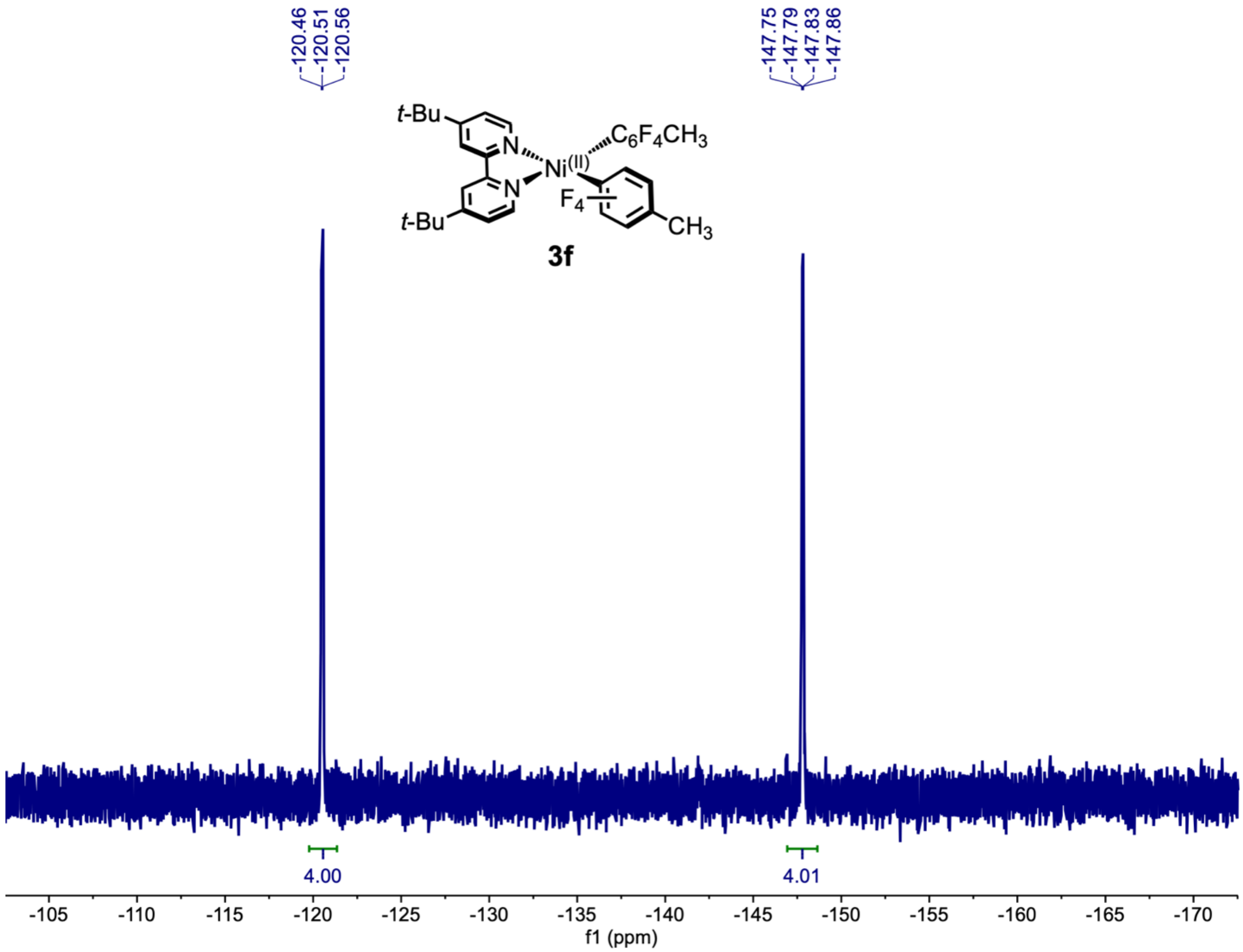

Figure S23. ^{19}F NMR (376 MHz, CD_2Cl_2) spectrum of complex **3f** prepared utilizing method A.

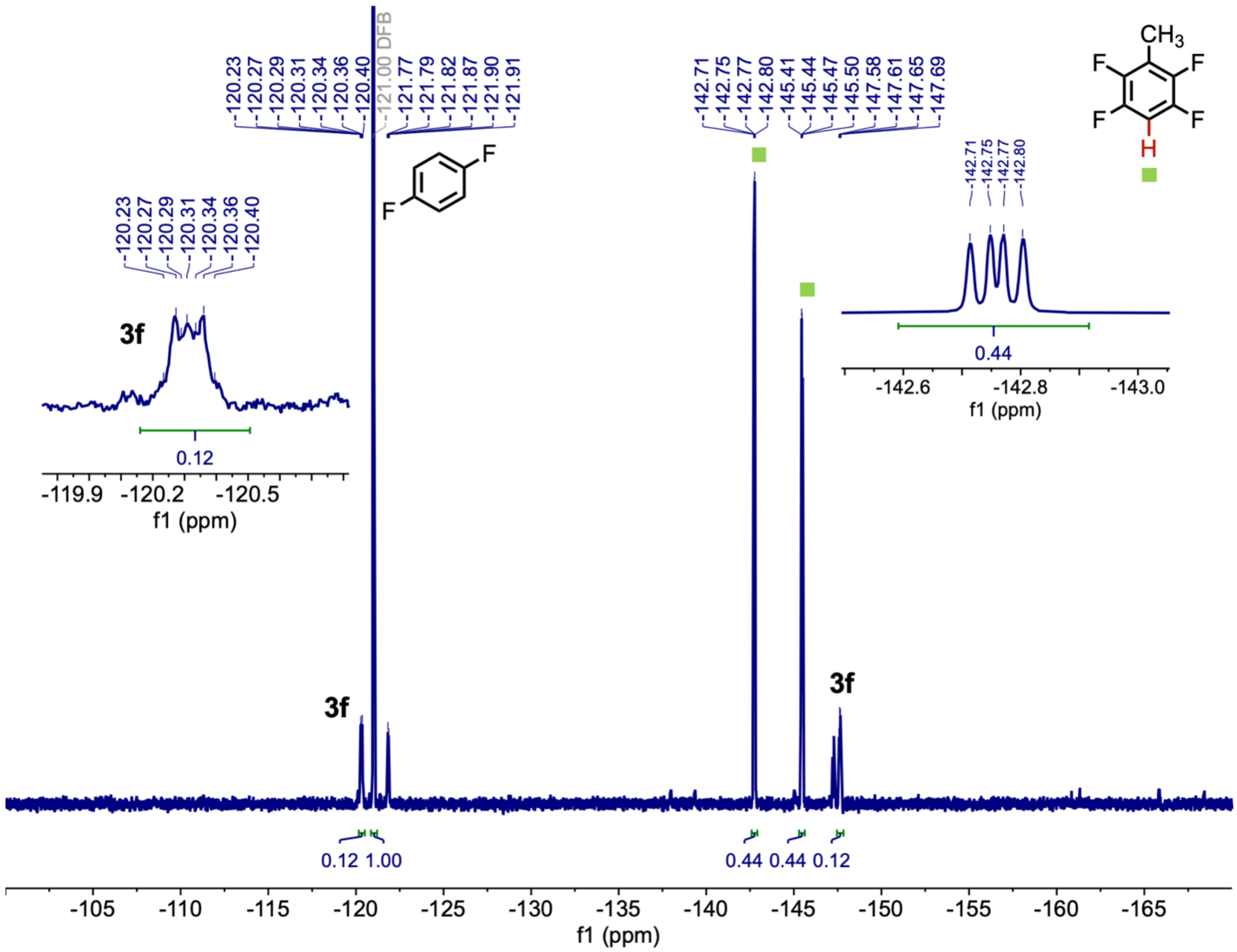

Figure S24. ^{19}F NMR (376 MHz, CD_3CN) spectrum of complex **3f** obtained utilizing method B.[18,19]

($^{t\text{-Bu}}$bpy)Ni(C_6F_4H)$_2$ (3g):[20] Yield of 67%[b] (heated to 60 °C). ^{1}H NMR (400 MHz, CD_3CN): δ 8.21 (d, 2H), 7.40 – 7.31 (m, 6H), 1.39 (s, 18H). ^{13}C NMR (101 MHz, CD_2Cl_2): δ 166.02, 155.88, 151.01, 125.08, 118.42, 36.46, 30.31. ^{19}F NMR (376 MHz, CD_3CN): δ -119.58 (qd, J = 15.2, 10.8 Hz, 4F), -143.59 (q, J = 15.2 Hz, 4F).

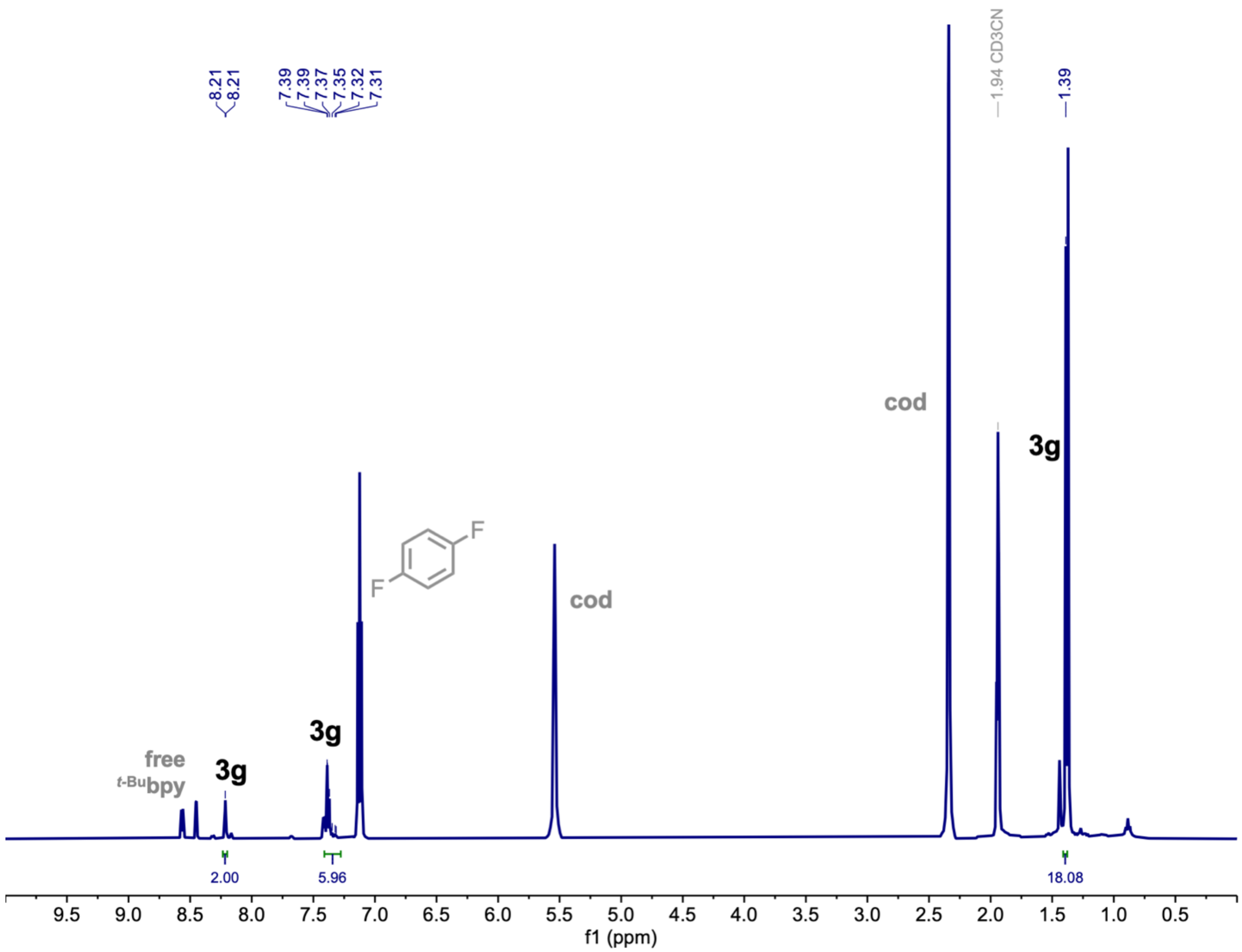

Figure S25. ^{1}H NMR (400 MHz, CD_3CN) spectrum of complex **3g** obtained utilizing method B.

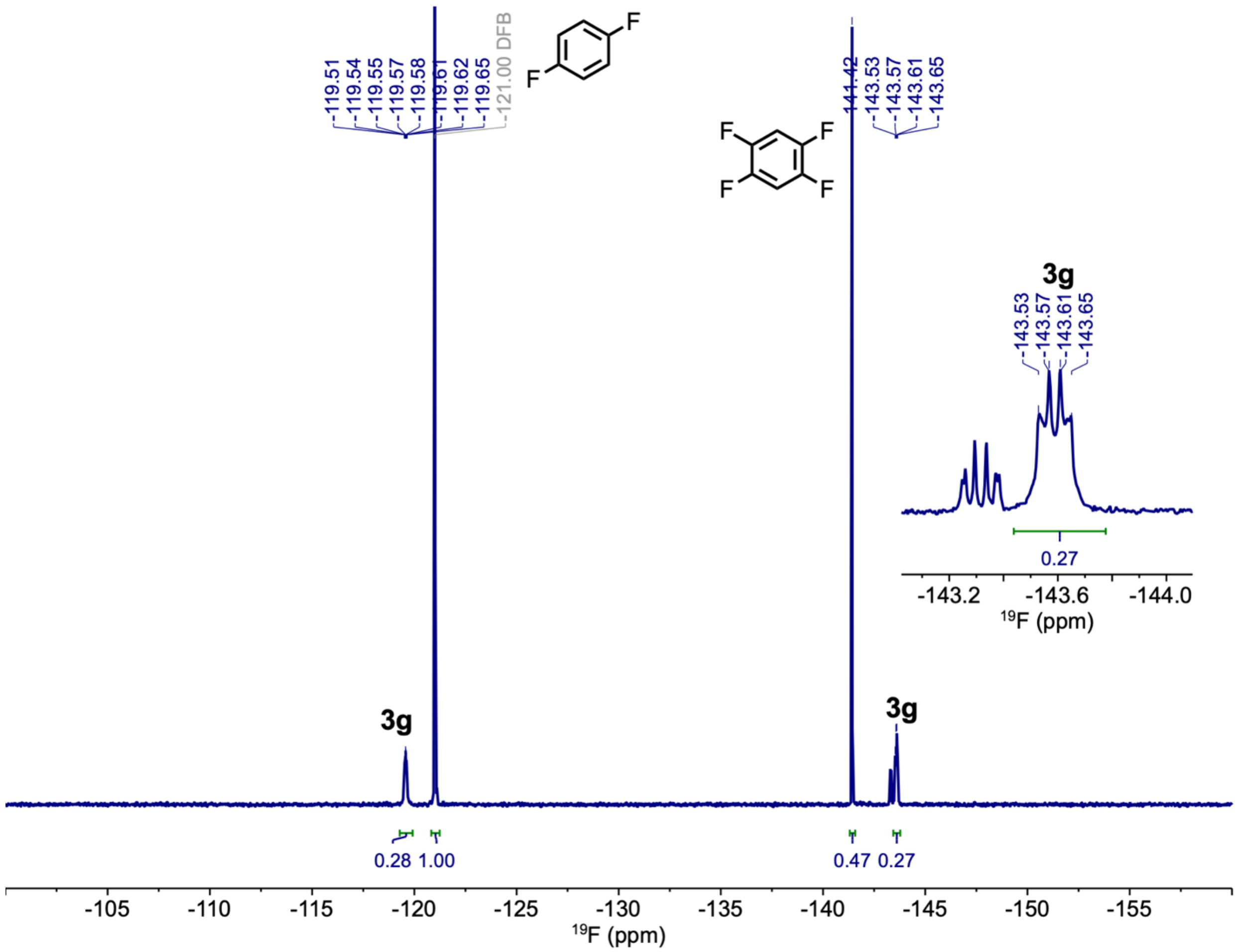

Figure S26. ^{19}F NMR (376 MHz, CD_3CN) spectrum of complex **3g** obtained utilizing method B.

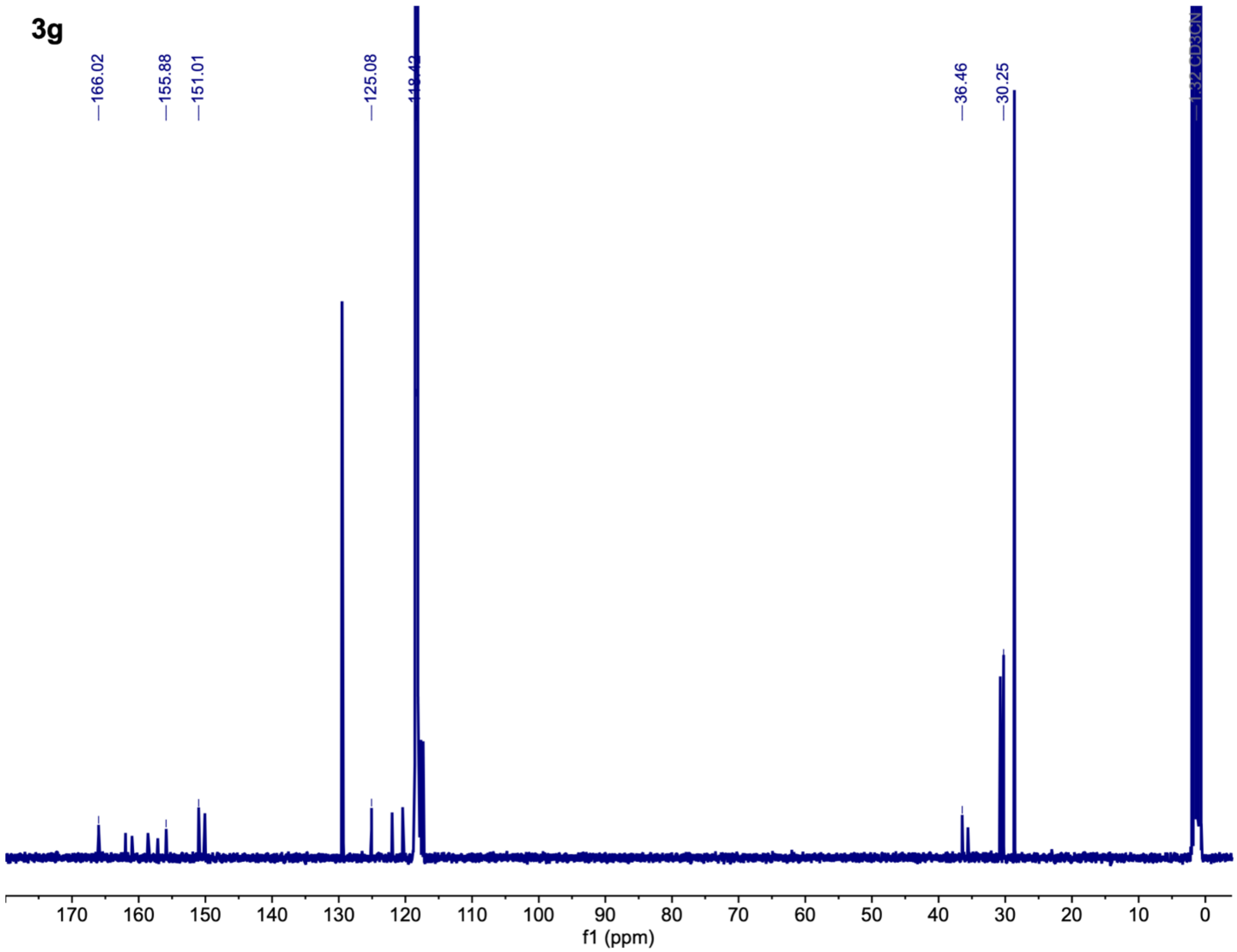

Figure S27. ^{13}C NMR (101 MHz, CD_3CN) spectrum of complex **3g** obtained utilizing method B.

($^{t\text{-Bu}}$bpy)Ni($C_6F_4CHCH_2$)$_2$ (3h): Yield of 56%[b]. ^{1}H NMR (400 MHz, CD_3CN): δ 8.21 (d, *J* = 5.4 Hz, 2H), 7.50 – 7.35 (m, 4H), 6.67 (dd, *J* = 18.0, 11.9 Hz, 2H), 5.90 (d, *J* = 18.0 Hz, 2H), 5.52 (d, *J* = 11.9 Hz, 2H), 1.39 (s, 18H). ^{19}F NMR (376 MHz, CD_3CN): δ -120.03 – -120.38 (m, 4F), -147.90 – -148.14 (m, 4F).

3h

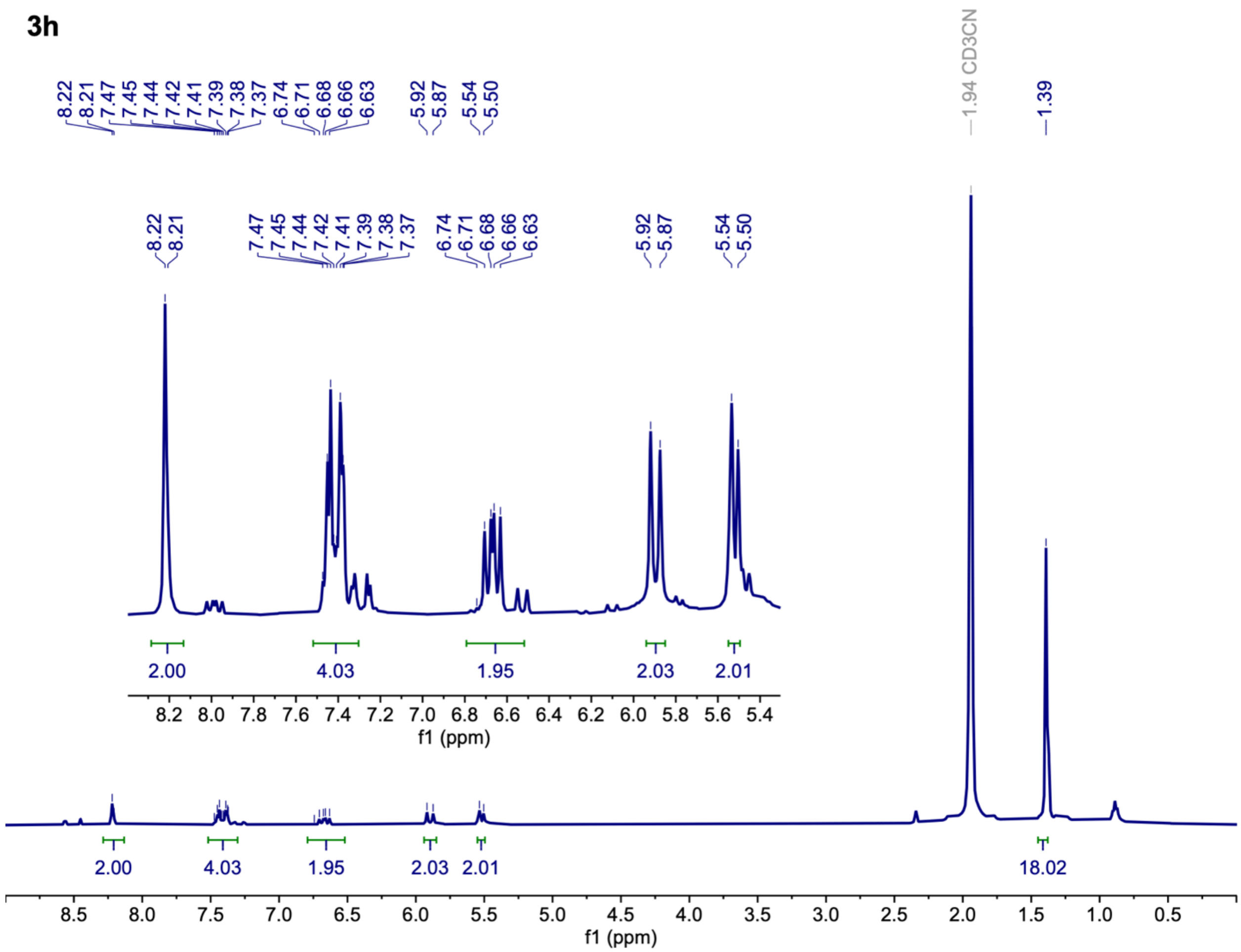

Figure S28. ^{1}H NMR (400 MHz, CD_3CN) spectrum of complex **3h** obtained utilizing method A. Complete isolation of complex **3h** was not achieved.

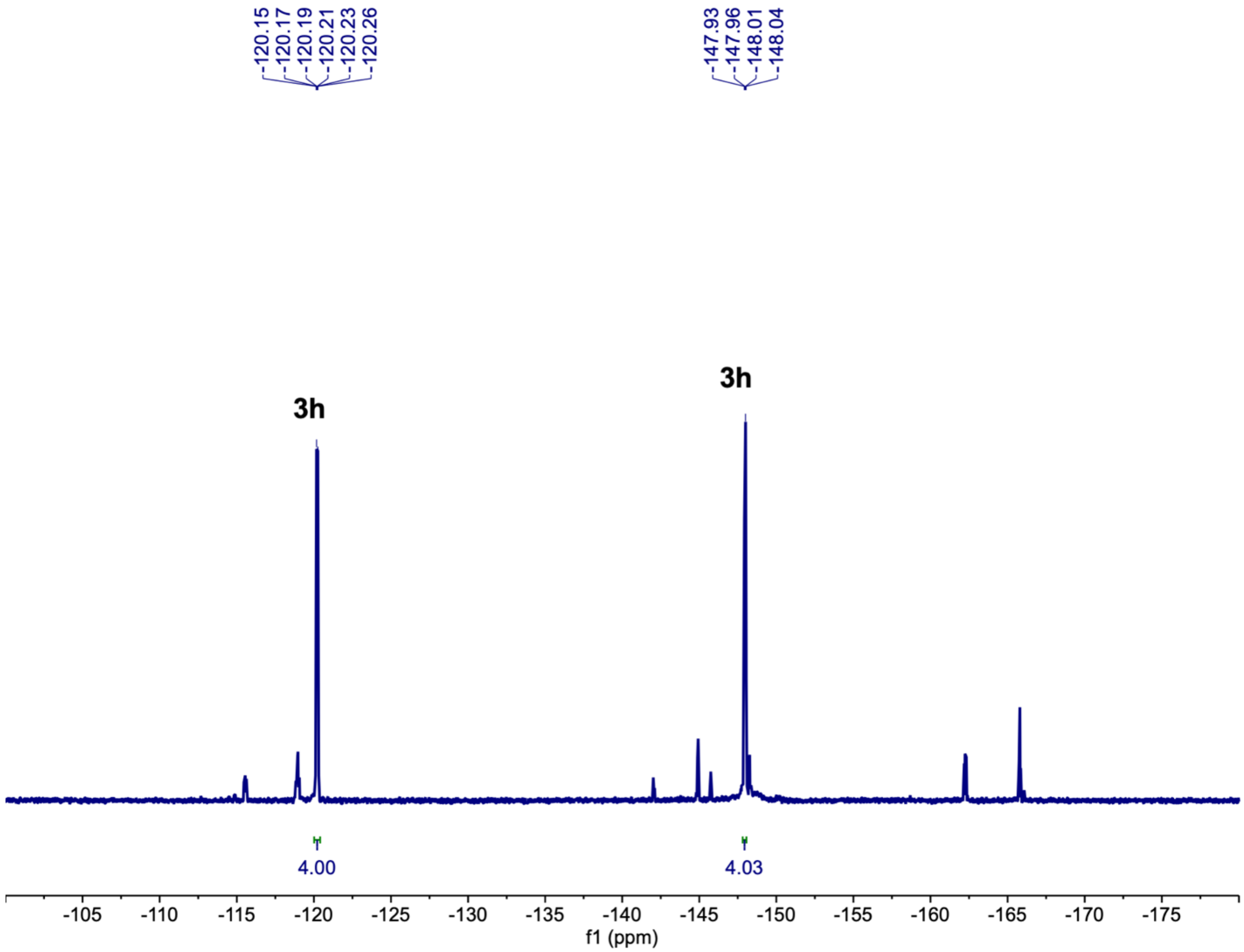

Figure S29. ^{19}F NMR (376 MHz, CD_3CN) spectrum of complex **3h** obtained utilizing method A. Complete isolation of complex **3h** was not achieved.

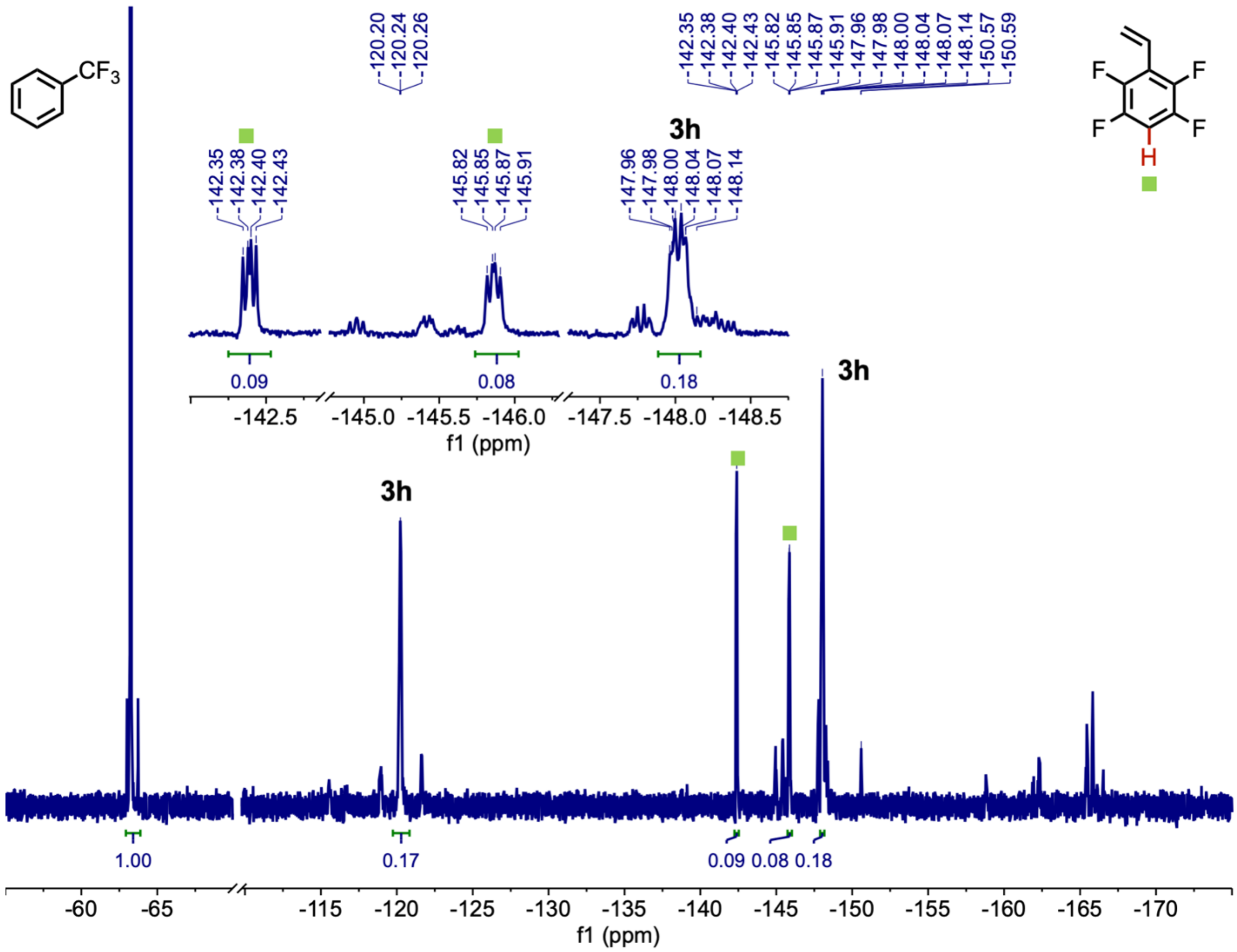

Figure S30. ^{19}F NMR (376 MHz, CD_3CN) spectrum of complex **3h** obtained utilizing method B.**[19]**

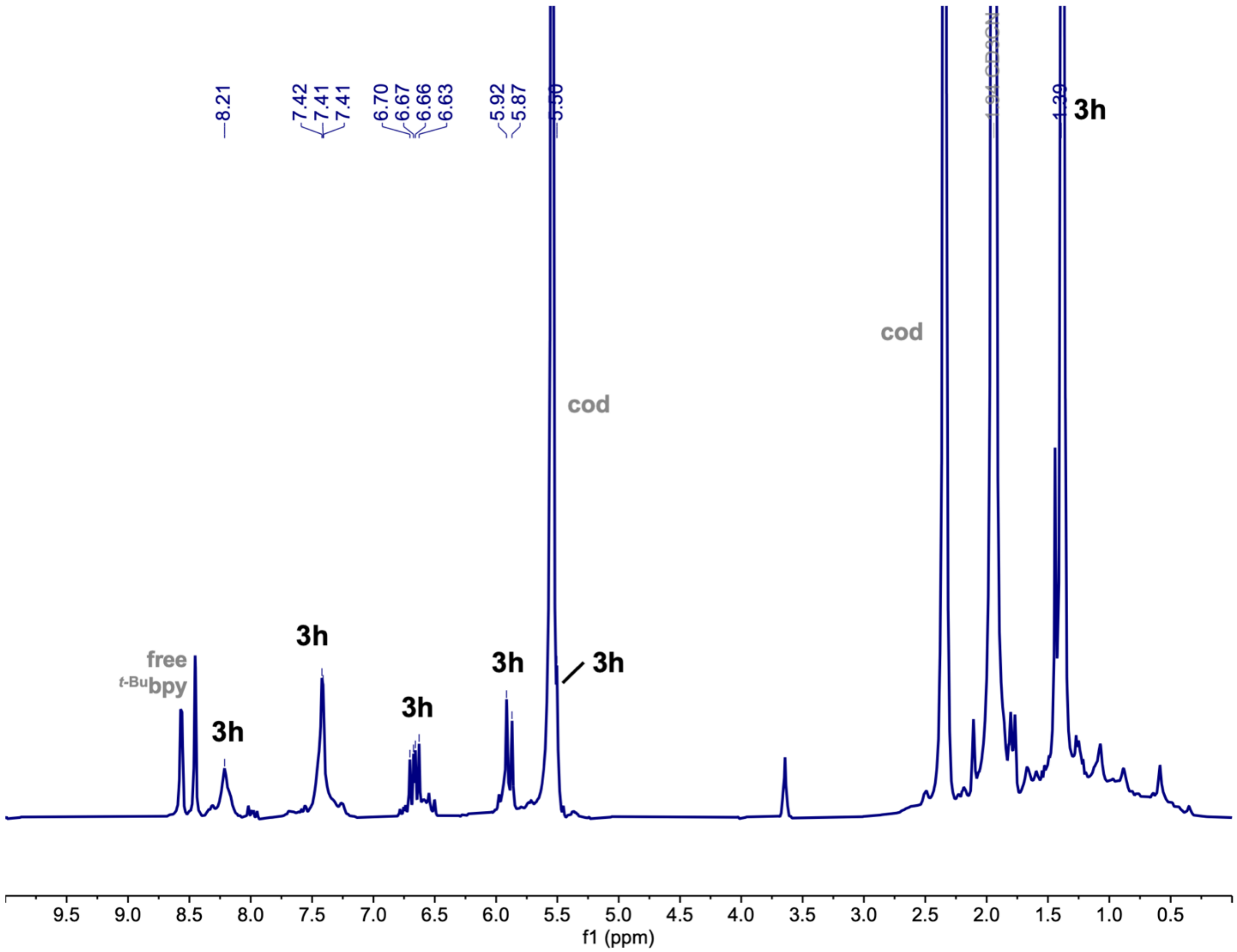

Figure S31. ^{1}H NMR (400 MHz, CD_3CN) spectrum of complex **3h** obtained utilizing method B.[19] No internal standard present.

($^{t\text{-Bu}}$bpy)Ni(C_6F_4CN)$_2$ (3i): Yield of 43%^{b}. ^{1}H NMR (400 MHz, CD_3CN): δ 8.23 (d, 2H), 7.40 – 7.27 (m, 2H), 1.39 (s, 18H). ^{13}C NMR (101 MHz, CD_3CN): δ 166.76, 155.99, 151.24, 125.17, 120.77, 36.55, 30.22. ^{19}F NMR (376 MHz, CD_3CN): δ -116.22 – -116.42 (m, 4F), -138.84 – -139.04 (m, 4F).

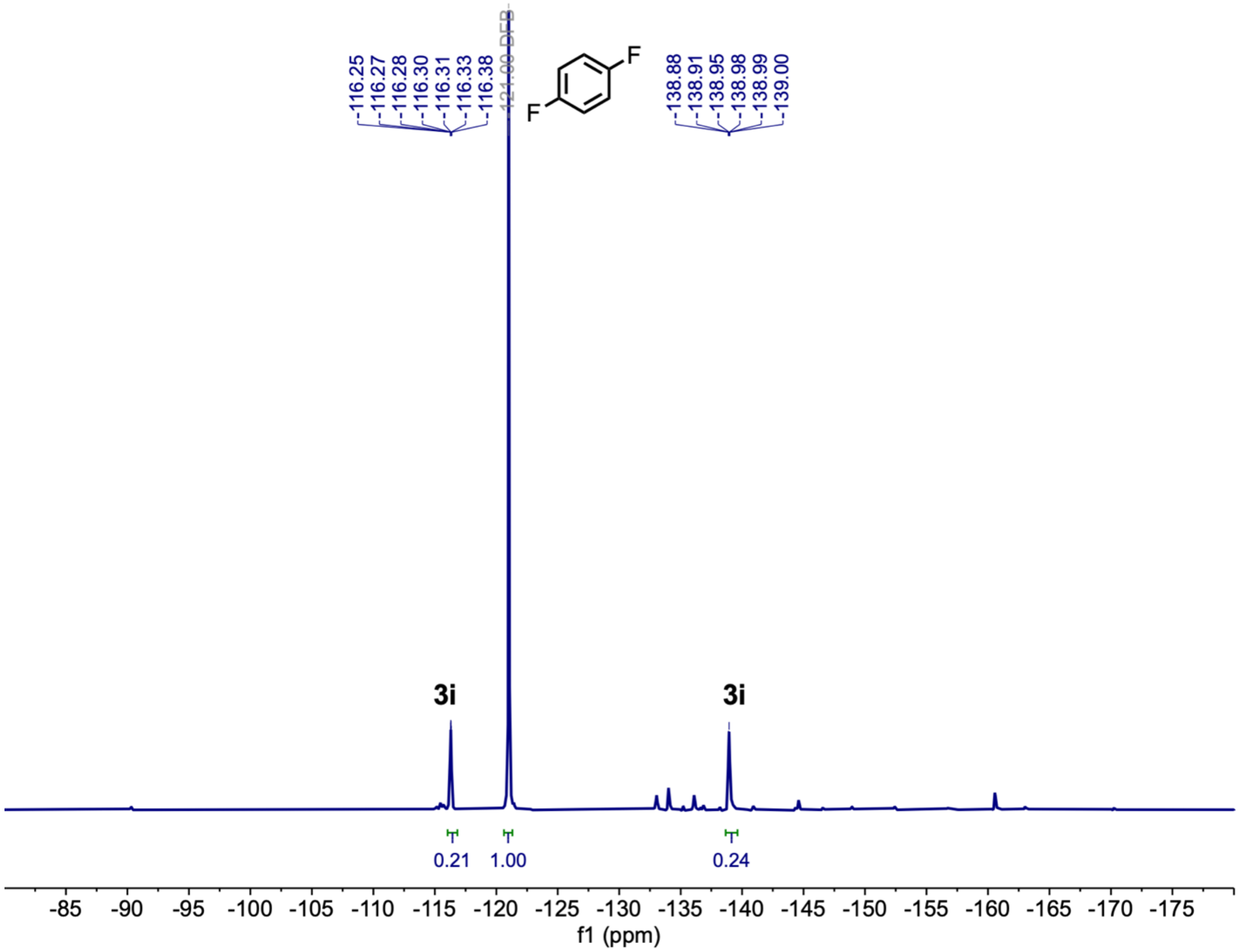

Figure S32. ^{19}F NMR (376 MHz, CD_3CN) spectrum of complex **3i** obtained utilizing method B.

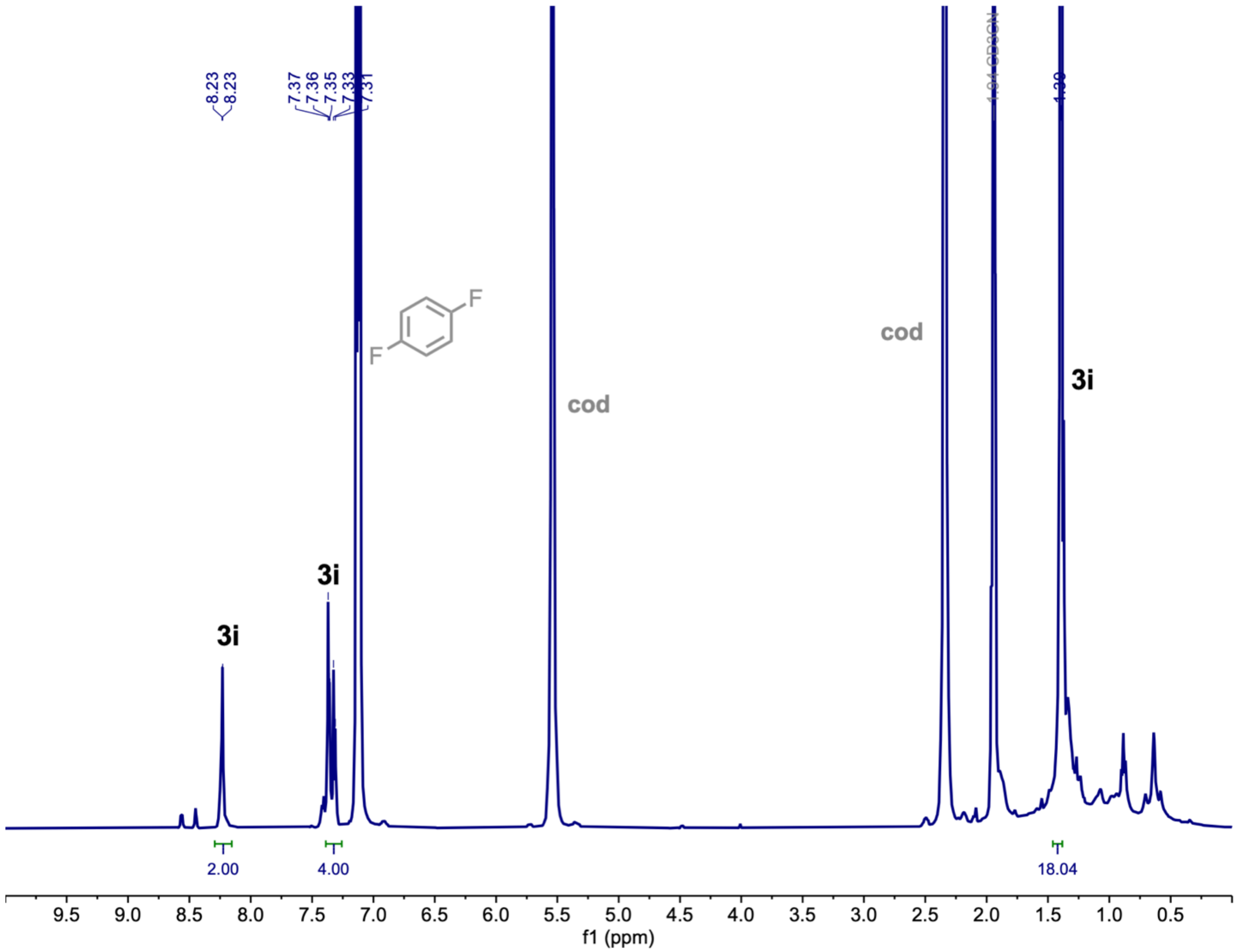

Figure S33. ^{1}H NMR (400 MHz, CD_3CN) spectrum of complex **3i** obtained utilizing method B.

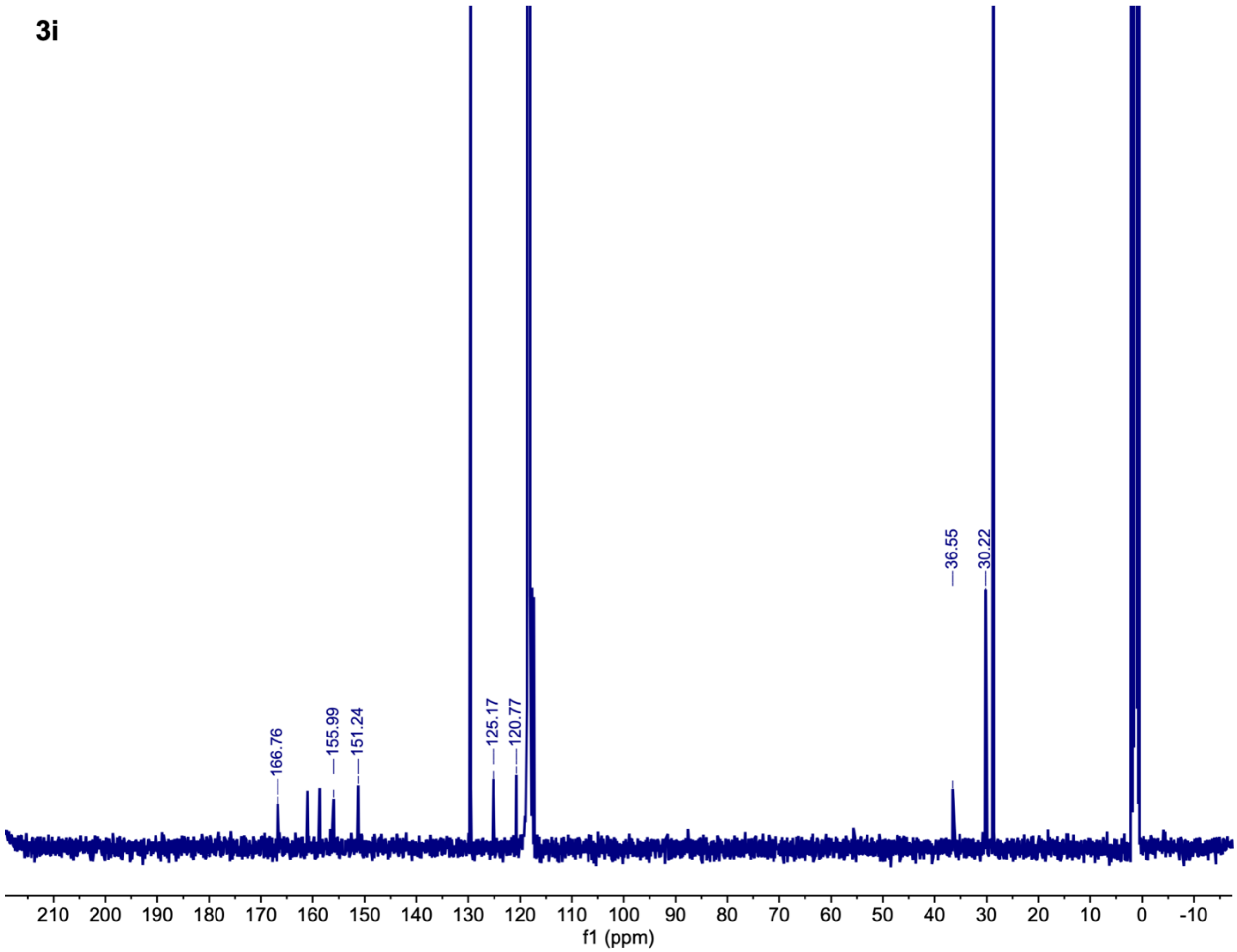

Figure S34. ^{13}C NMR (101 MHz, CD_3CN) spectrum of complex **3i** obtained utilizing method B.

($^{t\text{-Bu}}$bpy)Ni(C_6F_4COMe)$_2$ (3j): Yield of 23%[b]. ^1H NMR (400 MHz, CD_3CN): δ 8.22 (d, *J* = 6.6 Hz, 2H), 7.38 (m, 4H), 2.50 (m, 6H), 1.39 (s, 18H). ^{19}F NMR (376 MHz, CD_3CN): δ -118.65 (ddd, *J* = 21.7, 13.4, 7.4 Hz, 4F), -145.57 – -145.77 (m, 4F).

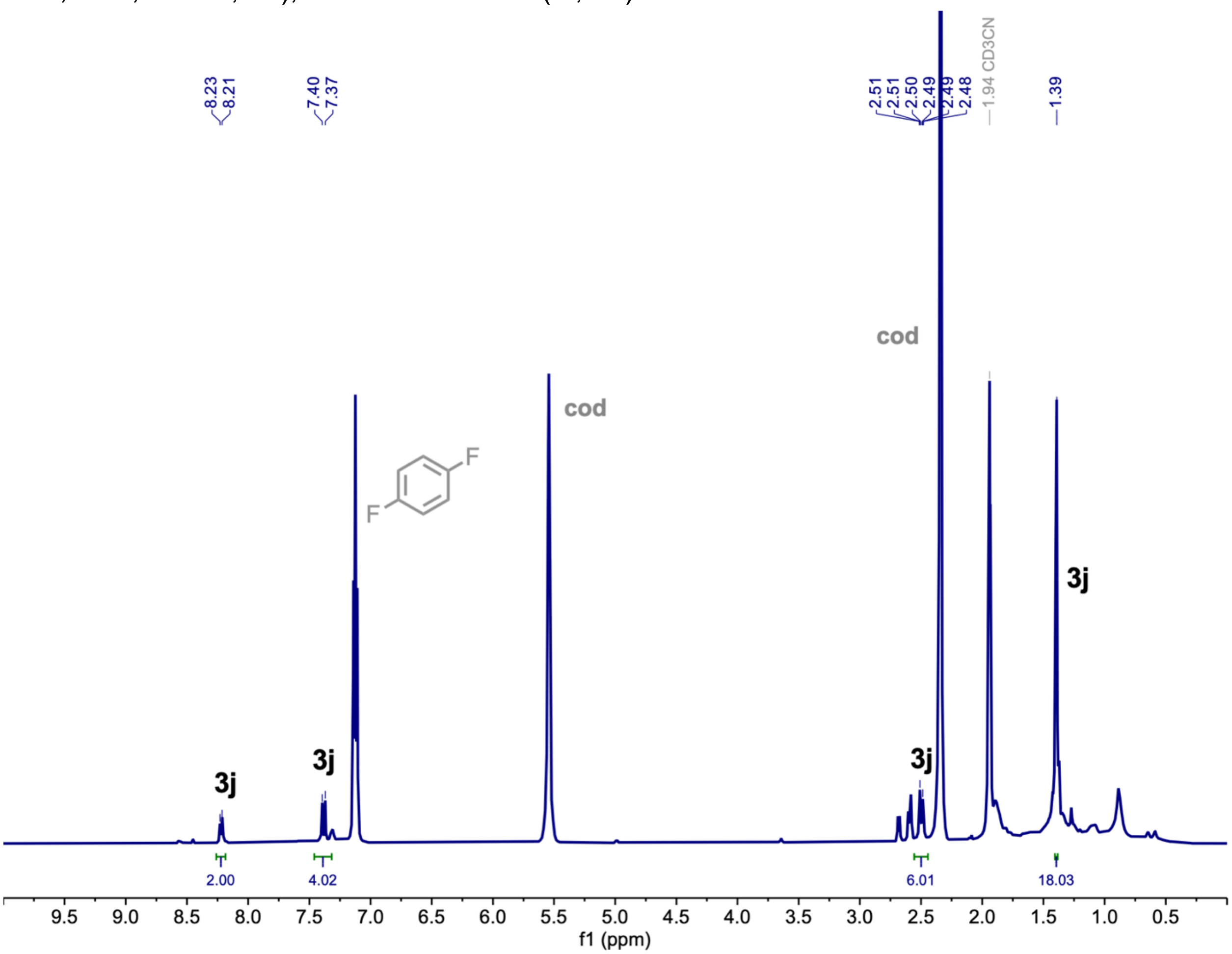

Figure S35. ^1H NMR (400 MHz, CD_3CN) spectrum of complex **3j** obtained utilizing method B.

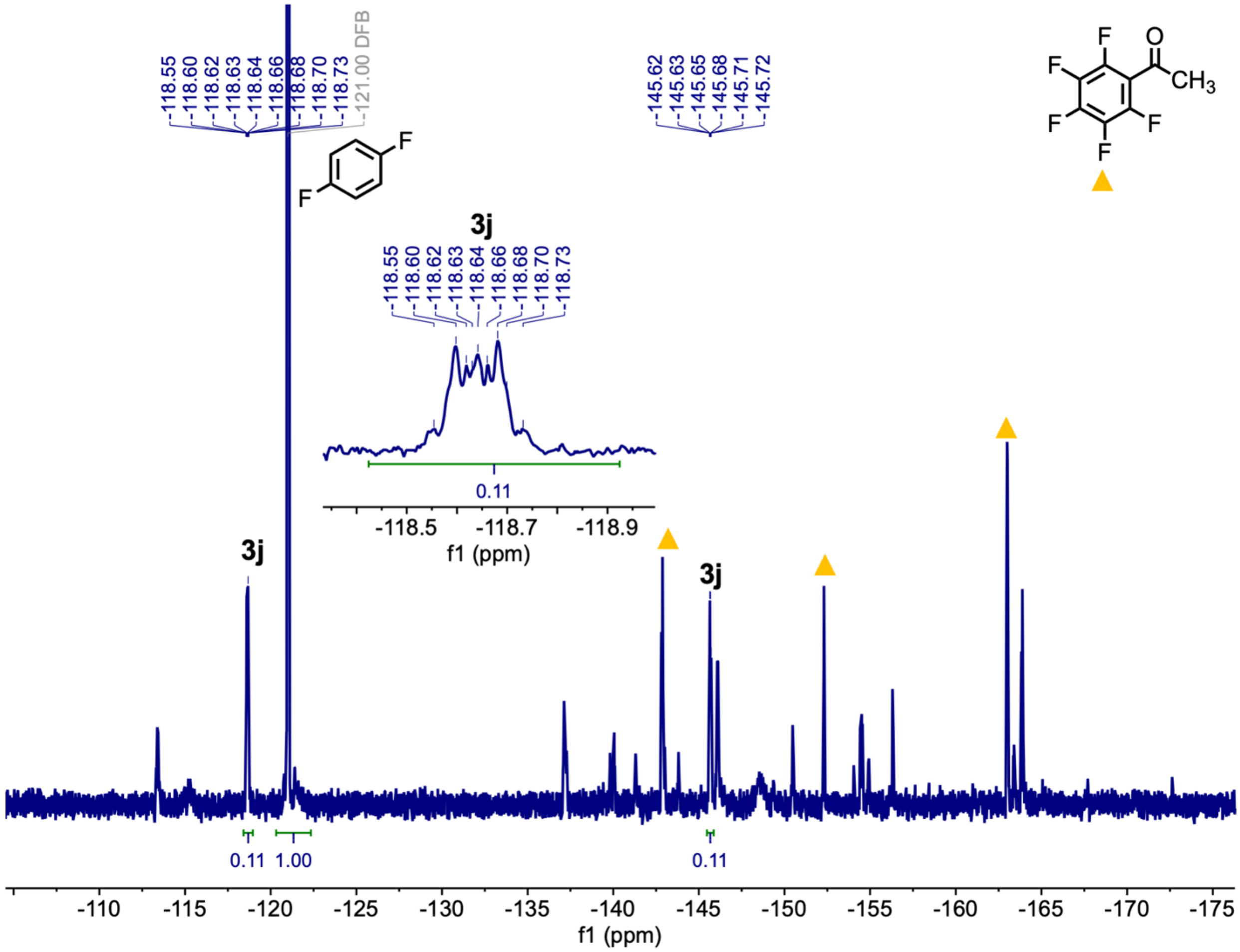

Figure S36. ^{19}F NMR (376 MHz, CD_3CN) spectrum of complex **3j** obtained utilizing method B.

($^{t\text{-Bu}}$bpy)Ni($C_{12}F_9$)$_2$ (3k): Yield of 78%[a]. Bright yellow powder. ^{1}H NMR (400 MHz, CD_2Cl_2): δ 7.97 (d, J = 2.0 Hz, 2H), 7.42 (d, J = 6.0 Hz, 2H), 7.38 (dd, J = 6.0, 2.0 Hz, 2H), 1.41 (s, 18H). ^{13}C NMR (101 MHz, CD_2Cl_2): δ 165.24, 155.41, 150.49, 124.79, 118.40, 35.97, 30.32. ^{19}F NMR (376 MHz, CD_2Cl_2): δ -117.53 – -117.94 (m, 4F), -137.98 (d, J = 12.0 Hz, 2F), -138.80 (d, J = 12.0 Hz, 2F), -142.23 – -142.65 (m, 4F), -153.95 (t, J = 20.8 Hz, 2F), -162.67 (td, J = 21.2, 7.8 Hz, 2F), -163.02 (td, J = 22.1, 8.7 Hz, 2F).

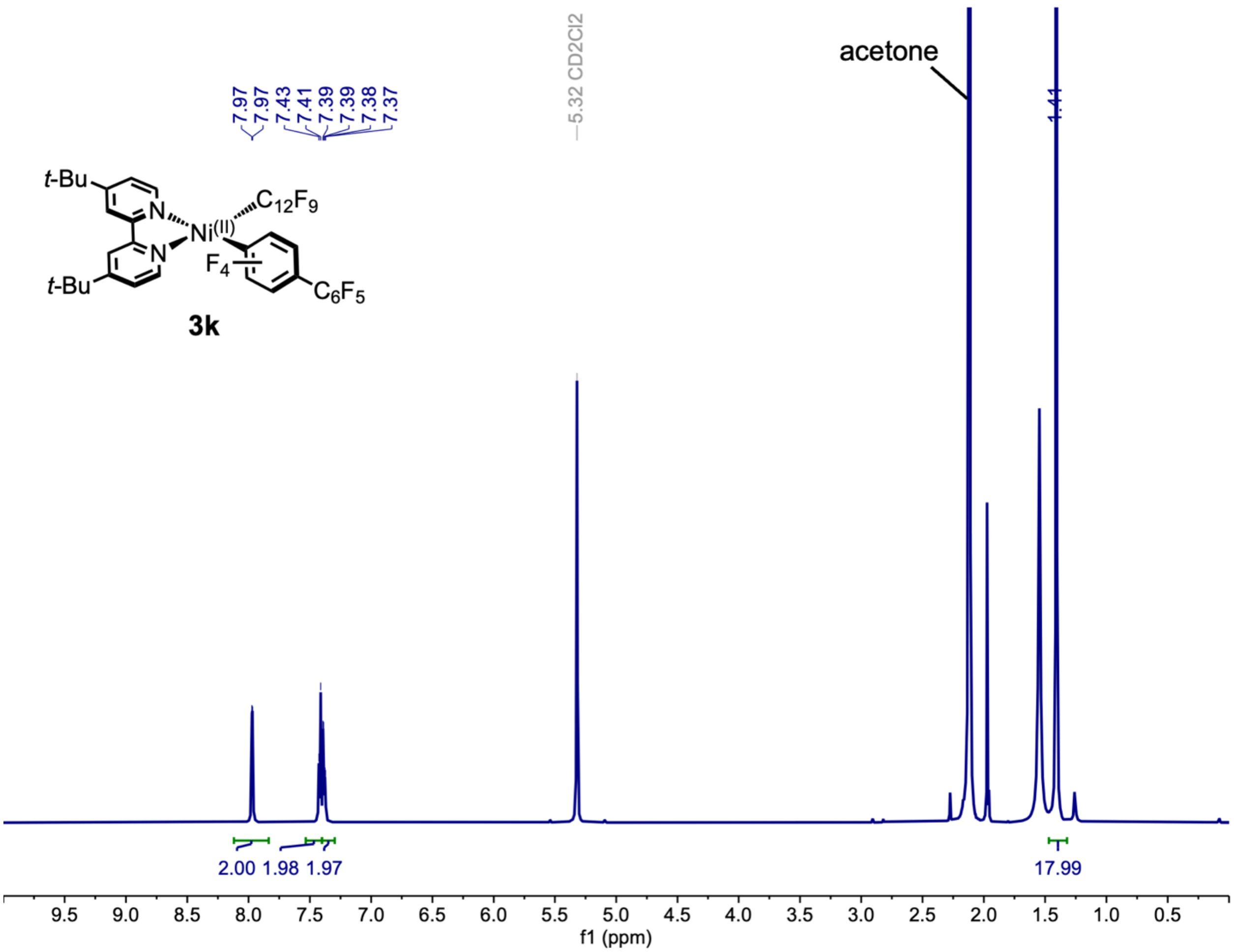

Figure S37. ^{1}H NMR (400 MHz, CD_2Cl_2) spectrum of complex **3k** obtained utilizing method A.

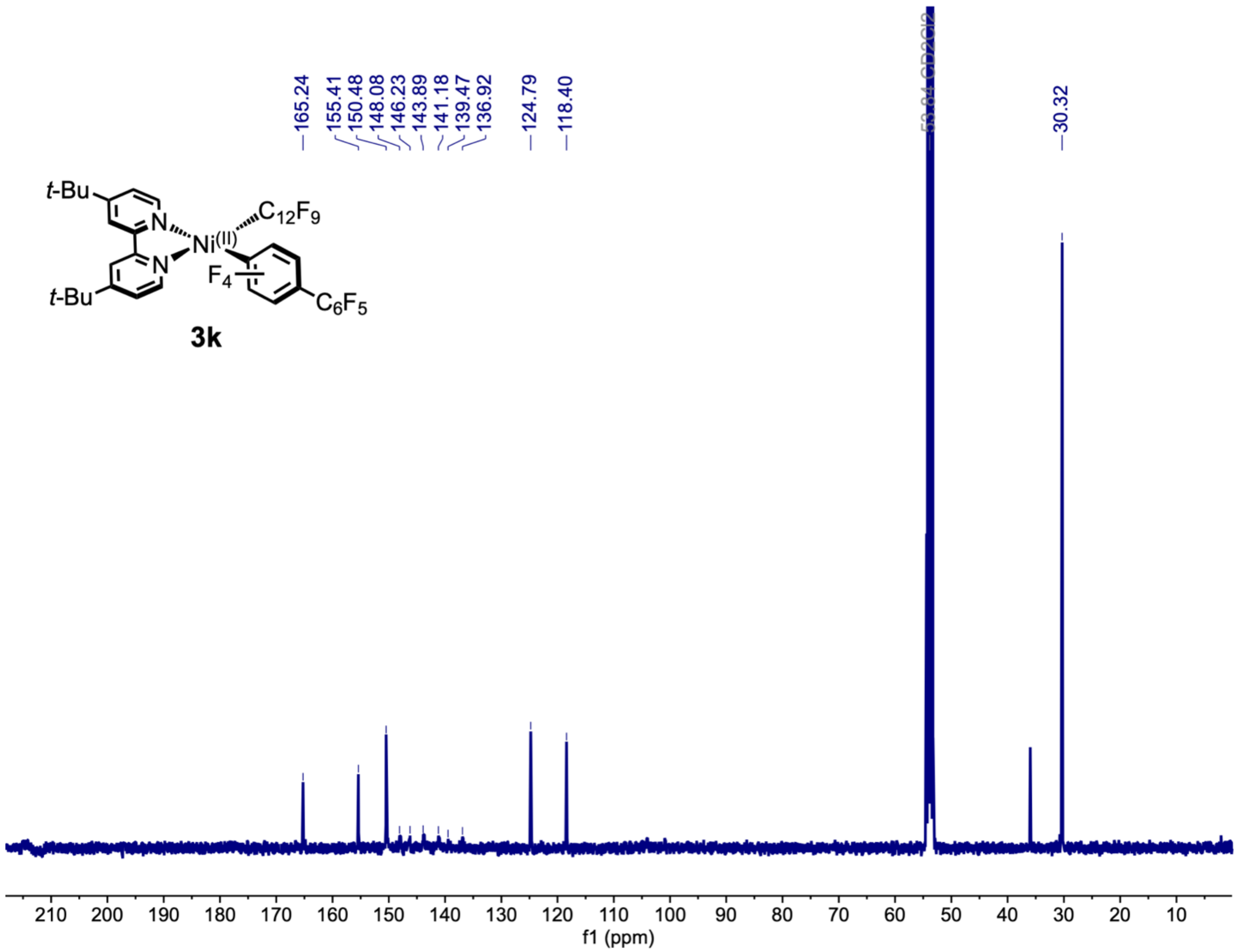

Figure S38. ^{13}C NMR (101 MHz, CD_2Cl_2) spectrum of complex **3k** obtained utilizing method A.

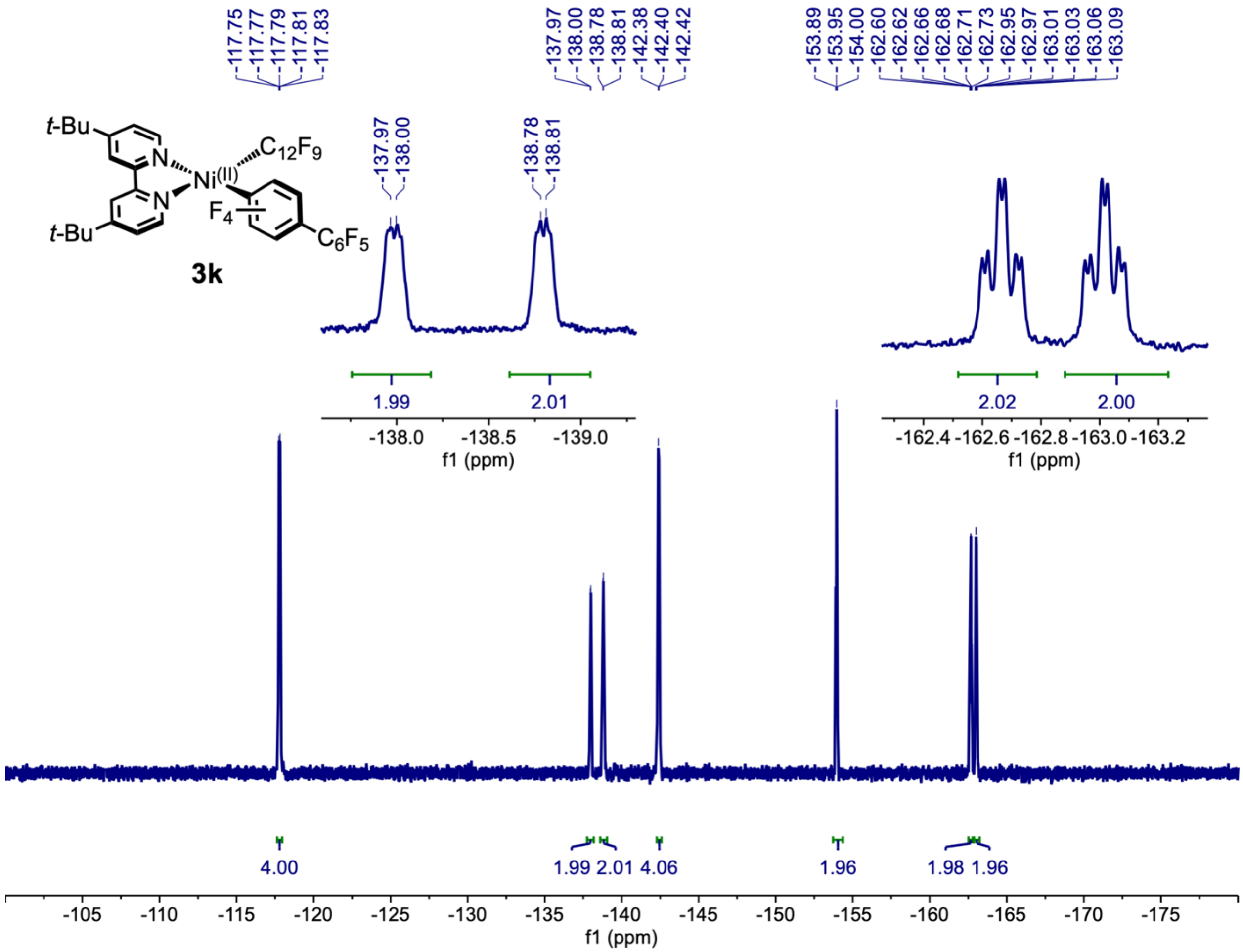

Figure S39. ^{19}F NMR (376 MHz, CD_2Cl_2) spectrum of complex **3k** obtained utilizing method A.

($^{t\text{-Bu}}$bpy)Ni($C_{10}F_7$)$_2$ (3l): Yield of 25%[a]. Yield of 54%[b]. Orange powder. ^{1}H NMR (400 MHz, CD_2Cl_2): δ 7.97 (d, *J* = 2.0 Hz, 2H), 7.38 (d, *J* = 6.0 Hz, 2H), 7.28 (dd, *J* = 6.0, 2.0 Hz, 2H), 1.39 (s, 18H). ^{13}C NMR (101 MHz, CD_2Cl_2): δ 165.12, 155.43, 150.49, 124.58, 118.39, 35.94, 30.31. ^{19}F NMR (376 MHz, CD_2Cl_2): δ -96.11 – -96.29 (m, 1F), -96.38 (m, 1F), -110.75 (m, 1F), -110.96 (m, 1F), -147.44 (m, 2F), -149.75 (m, 2F), -153.83 – -155.17 (m, 2F), -160.96 (m, 2F), -161.39 (m, 2F).

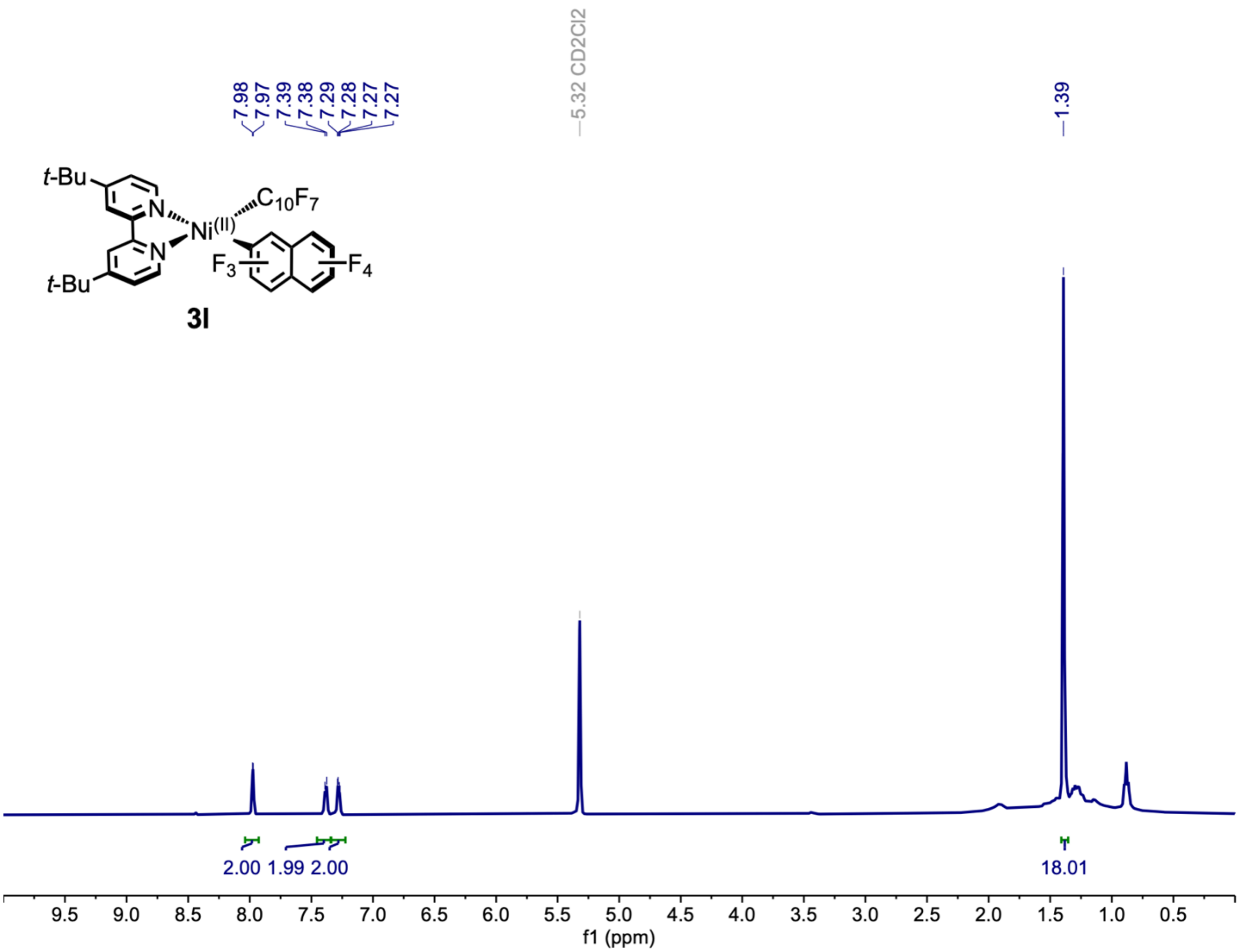

Figure S40. ^{1}H NMR (400 MHz, CD_2Cl_2) spectrum of complex **3l** obtained utilizing method A.

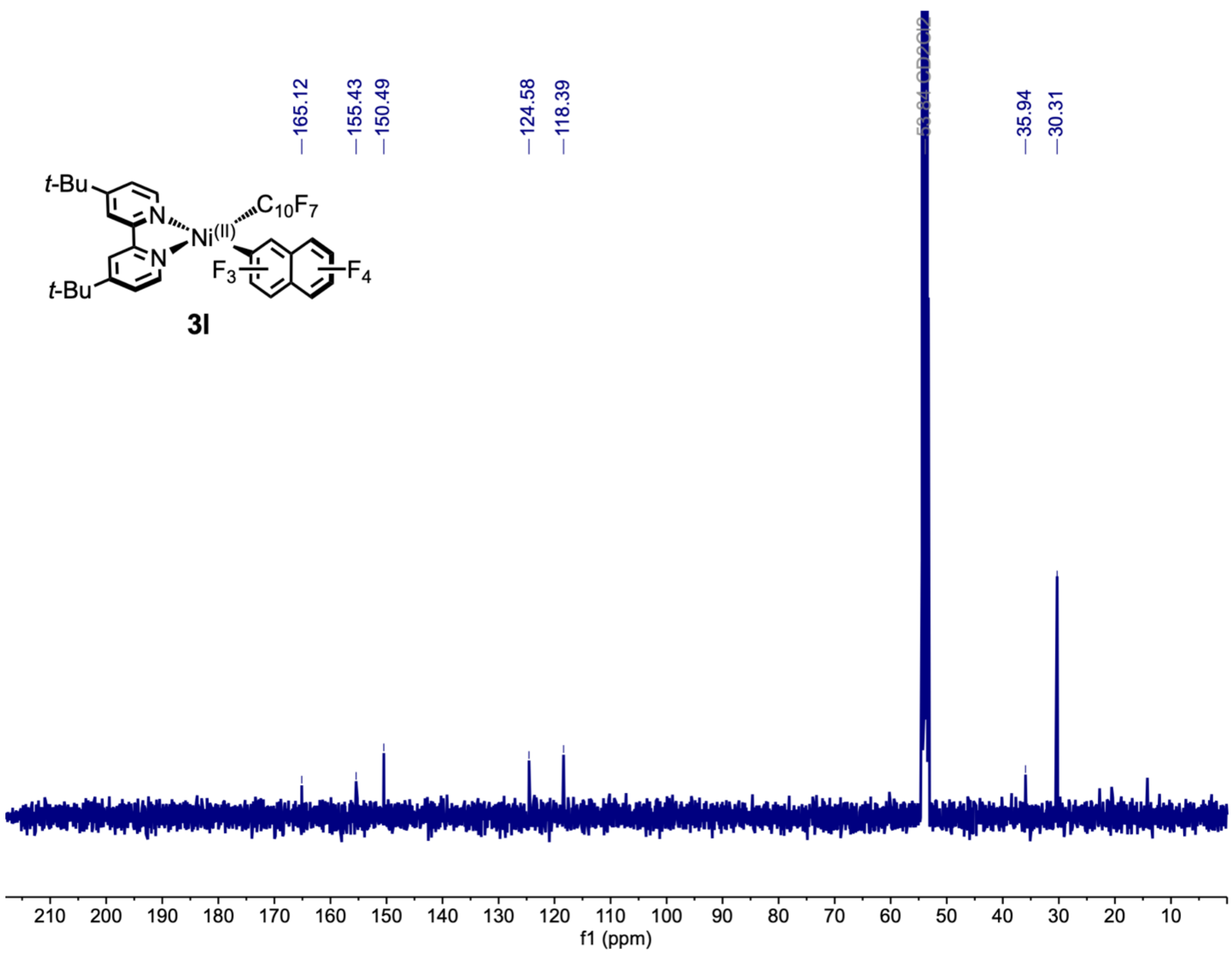

Figure S41. ^{13}C NMR (101 MHz, CD_2Cl_2) spectrum of complex **3l** obtained utilizing method A.

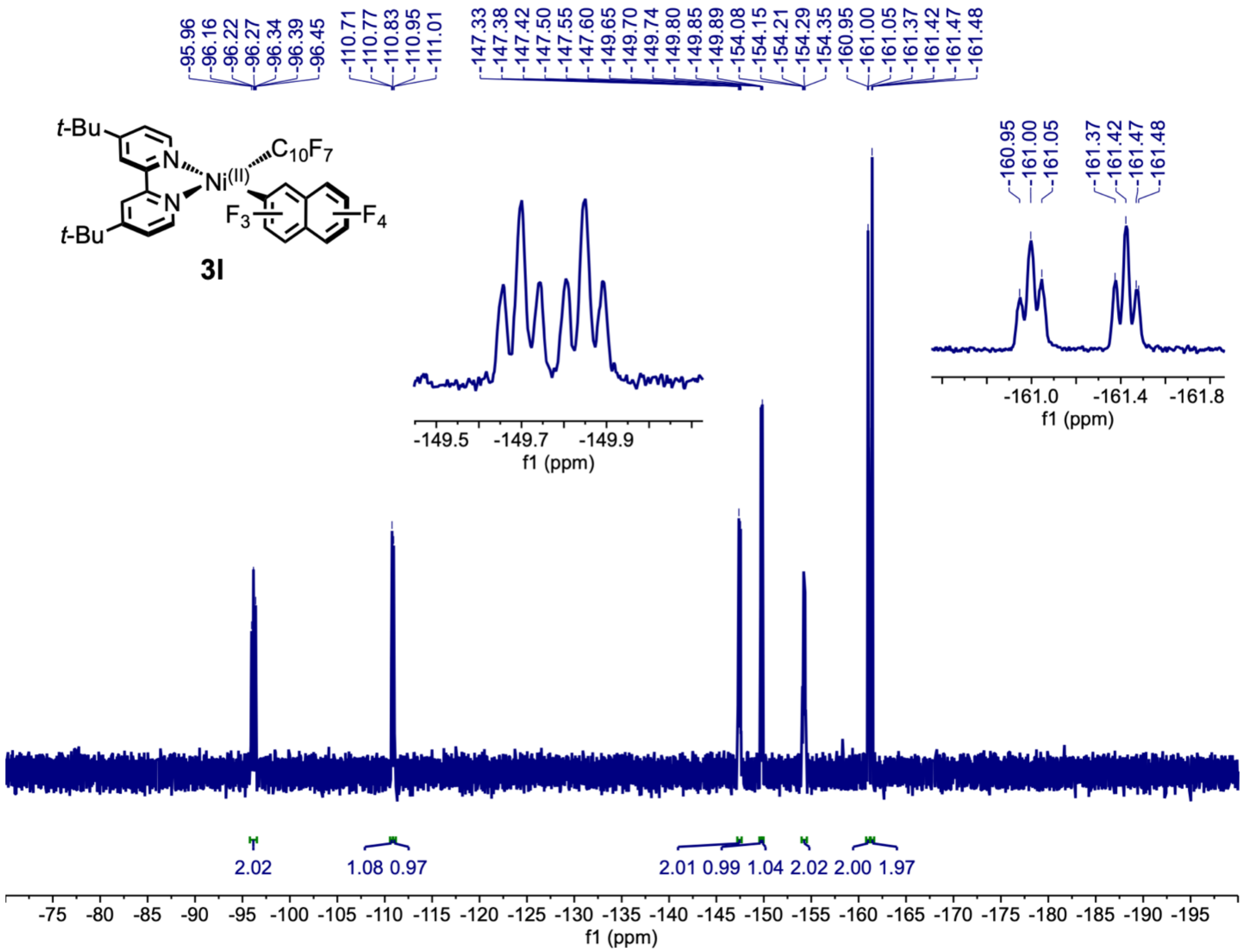

Figure S42. ^{19}F NMR (376 MHz, CD_2Cl_2) spectrum of complex **3l** obtained utilizing method A.

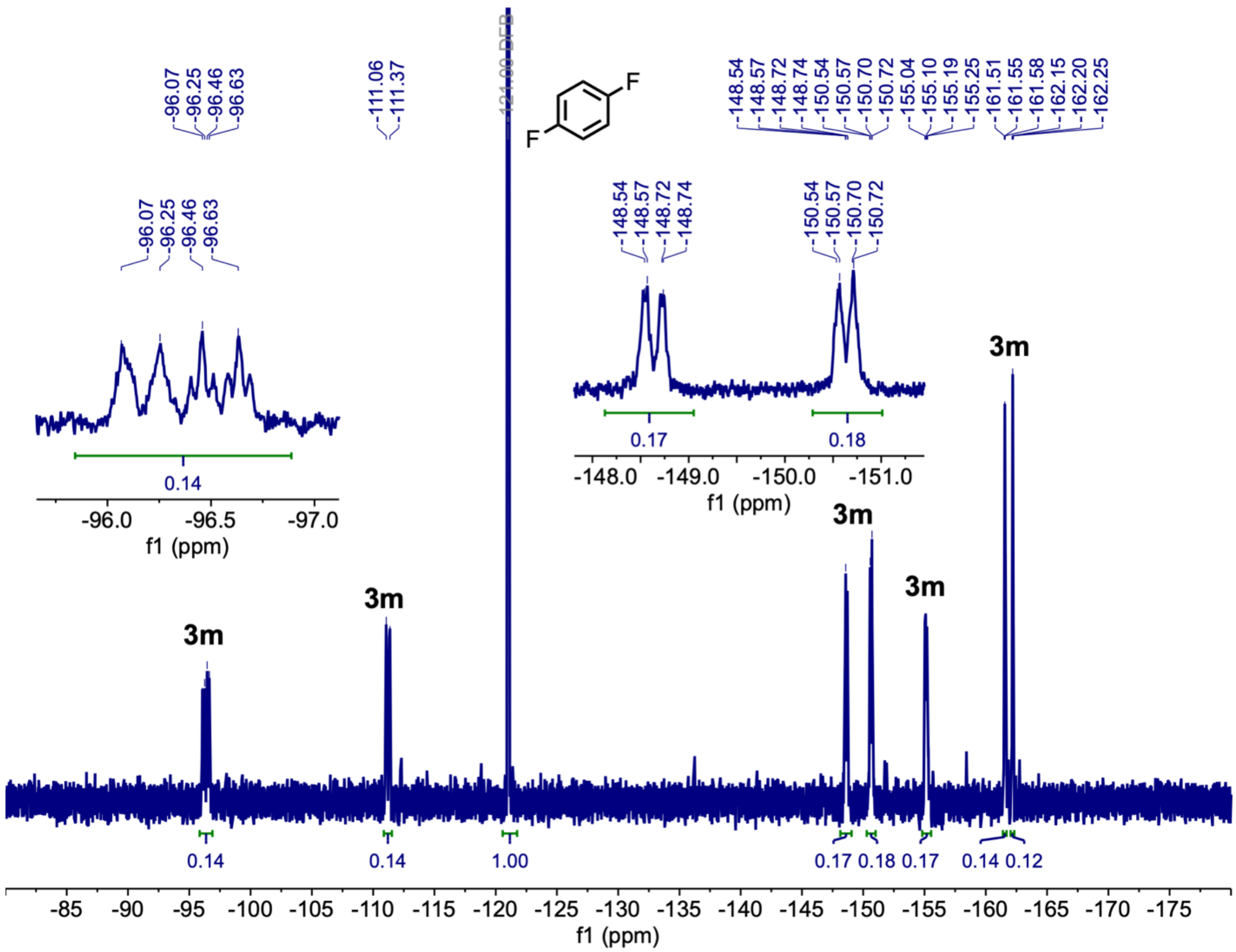

Figure S43. ^{19}F NMR (376 MHz, CD_3CN) spectrum of complex **3l** obtained utilizing method B.

3.2 *cis*–$(MeCN)_2Ni(PyF_4)_2$ complex

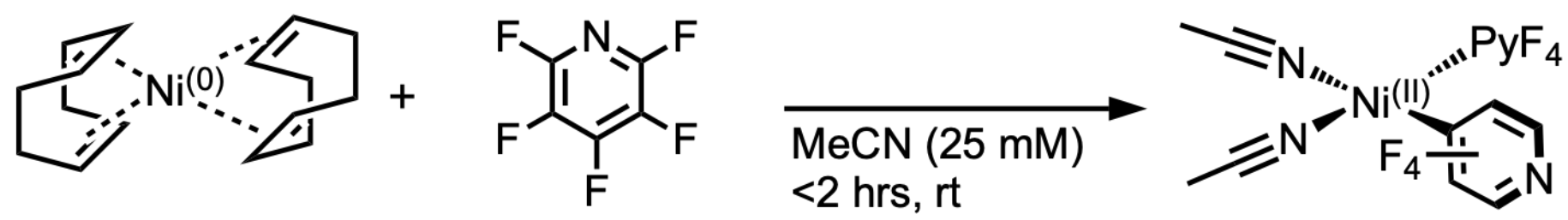

1 (1 equiv.) **2** (2 equiv.) **5**

In a nitrogen-filled glove box, an oven-dried 20 mL scintillation vial was charged with a Teflon-coated stir bar, $Ni(cod)_2$ (137.5 mg, 0.5 mmol, 1 eq.), and pentafluoropyridine (110 µL, 1.0 mmol, 2.0 eq.). MeCN (20 mL) was added to give a nominal nickel concentration of 25 mM, and the mixture was stirred for 1–2 h until homogeneous, affording a translucent yellow-green solution of synthon **5**. This solution was used directly for the ligand-substitution reactions described in Section 3.3. Prolonged standing for more than approximately 5 h resulted in the formation of Ni black. The reaction vial was then removed from the glovebox and the excess solvent was removed under vacuum. To the dry green-gray solid, ~10 mL of pentane was added and the mixture was allowed to stir vigorously for 15 min. The solid was then collected on a glass frit and dried under vacuum. The fine gray powder was collected in 12% yield (24 mg).

^{1}H NMR (400 MHz, DMSO-d_6): δ 2.07 (s, 6H). ^{13}C NMR (101 MHz, DMSO-d_6): δ 147.46 – 144.36 (m), 143.46 (m), 141.36 – 138.23 (m), 118.33, 1.39. ^{19}F NMR (376 MHz, CD_3CN): δ -101.10 – -102.06 (m, 4F), -124.80 – -125.72 (m, 4F). ^{19}F NMR (376 MHz, DMSO-d_6): δ -100.61 – -101.99 (m, 4F), -122.37 – -123.15 (m, 4F).

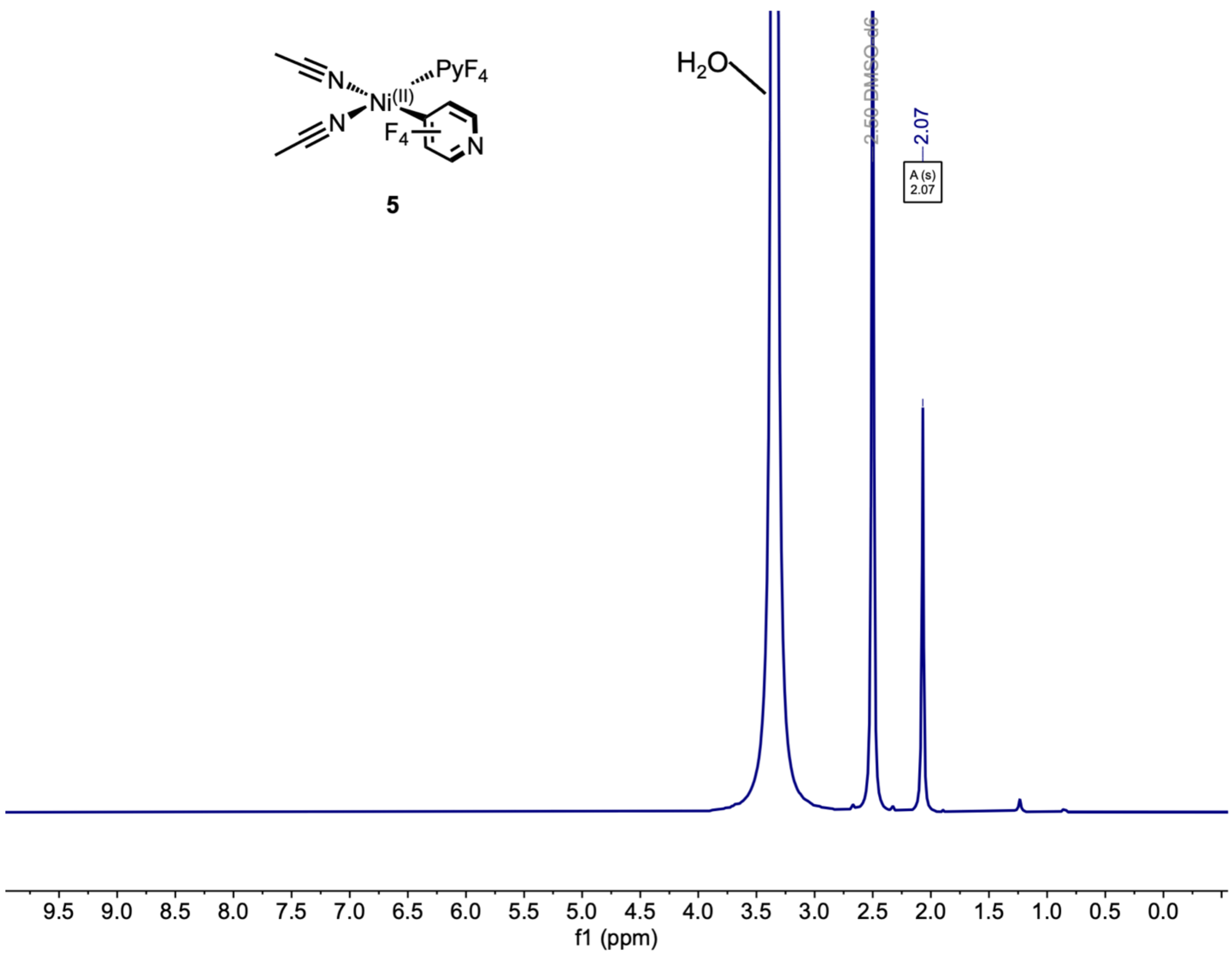

Figure S44. ^{1}H NMR (400 MHz, DMSO-d_6) spectrum of complex **5**.

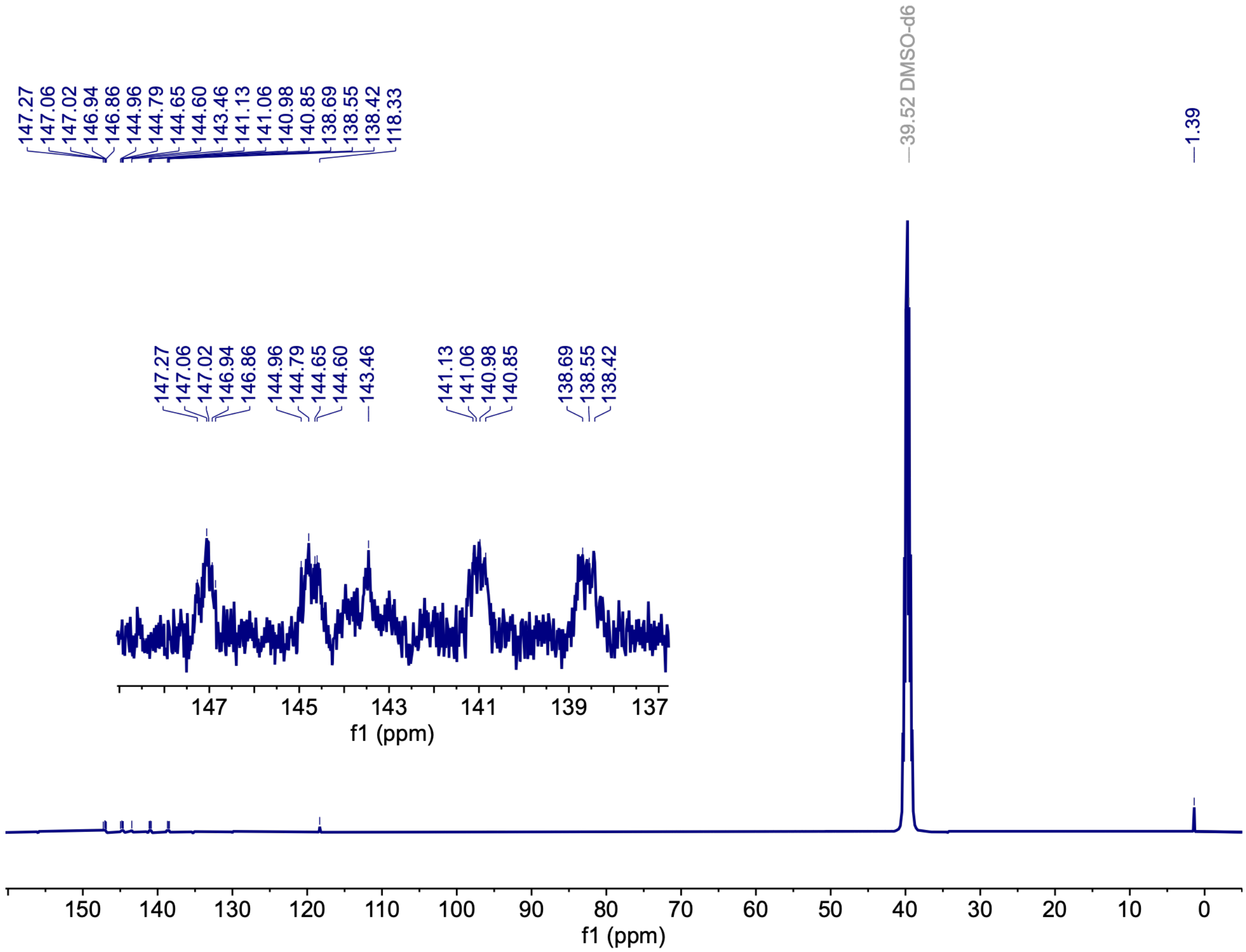

Figure S45. ^{13}C NMR (101 MHz, DMSO-d_6) spectrum of complex **5**.

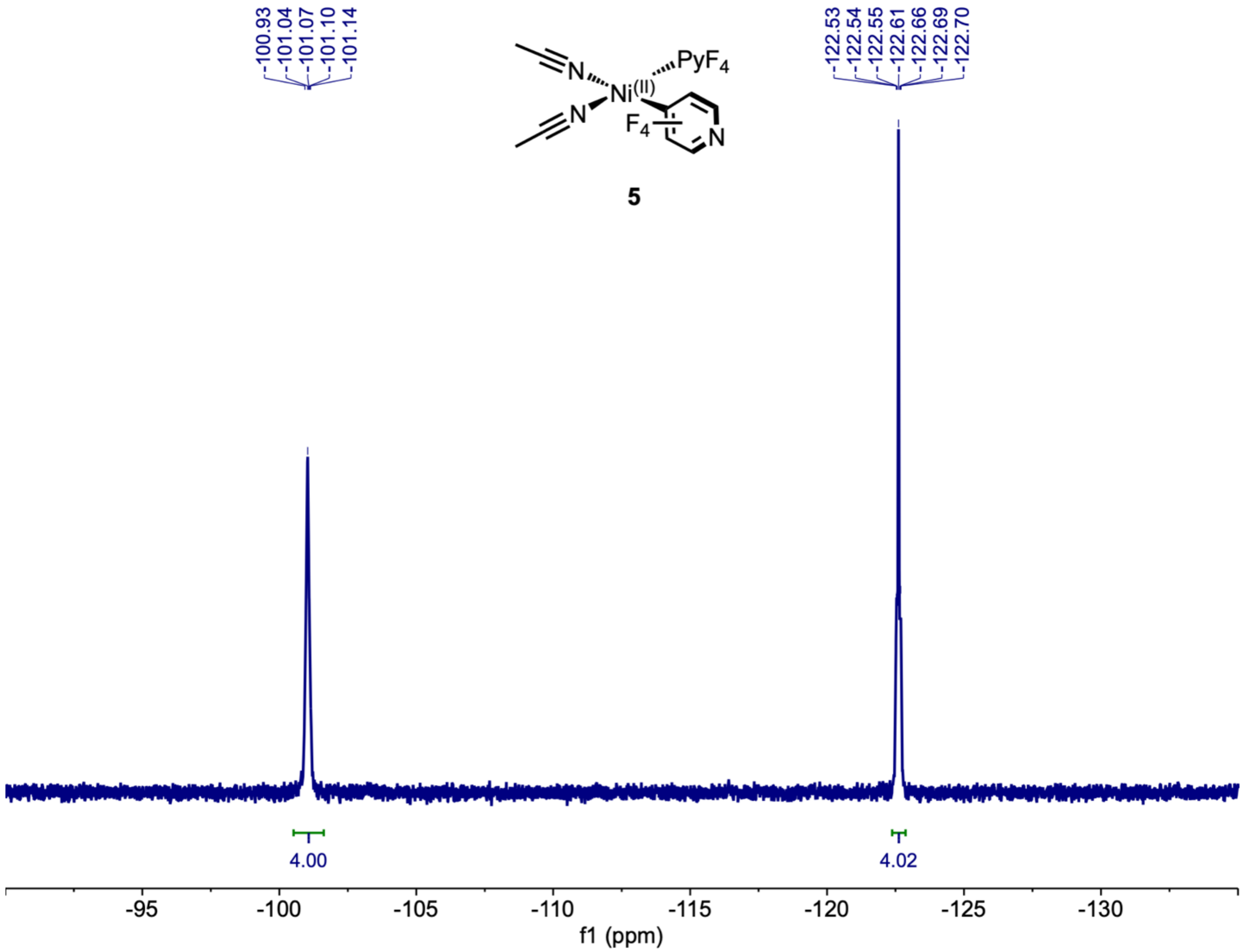

Figure S46. ^{19}F NMR (376 MHz, DMSO-d_6) spectrum of complex **5.**

3.3 (^{R}bpy)Ni(PyF_4)$_2$ complexes

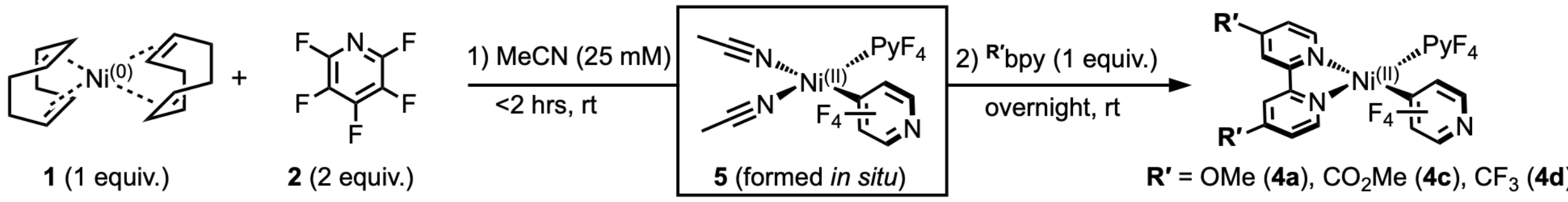

1 (1 equiv.) **2** (2 equiv.) **5** (formed *in situ*) **R′** = OMe (**4a**), CO_2Me (**4c**), CF_3 (**4d**)

Method C: Synthon **5** is formed *in situ* following the procedure described in section 3.2. To the MeCN solution containing synthon **5**, 1 equiv. of $^{R'}$bpy was added (**R′** = OMe, CO_2Me, CF_3), producing a rapid color change. The reaction was allowed to stir overnight to ensure complete ligand substitution. The following morning, the reaction mixture was concentrated to less than 1 mL, and pentane (approximately 10 mL) was added to precipitate and wash the Ni(II) product. The mixture was stirred vigorously for 10 min and allowed to settle, after which the supernatant was decanted. The pentane-washing step was repeated once, and the precipitate was collected by filtration through a glass frit and dried under vacuum before weighing.

Complexes prepared utilizing method C will be denoted by superscript *c* following the yield.

*Note: Synthon **5** and all of the (^{R}bpy)Ni(PyF_4)$_2$ complexes are air- and moisture-stable such that all steps after the formation of **5** can be performed in air.*

(^{OMe}bpy)Ni(PyF$_4$)$_2$ (4a): Yield of 44%^{c}. Bright yellow powder. ^{1}H NMR (400 MHz, CD_2Cl_2): δ 7.44 (d, *J* = 2.7 Hz, 2H), 7.25 (d, *J* = 6.5 Hz, 2H), 6.80 (dd, *J* = 6.5, 2.7 Hz, 2H), 3.97 (s, 6H). ^{13}C NMR (101 MHz, CD_2Cl_2): δ 168.78, 156.76, 152.22, 111.94, 109.04, 57.06, 22.74. ^{19}F NMR (376 MHz, CD_2Cl_2): δ -99.56 – -99.96 (m, 4F), -123.19 (m, 4F).

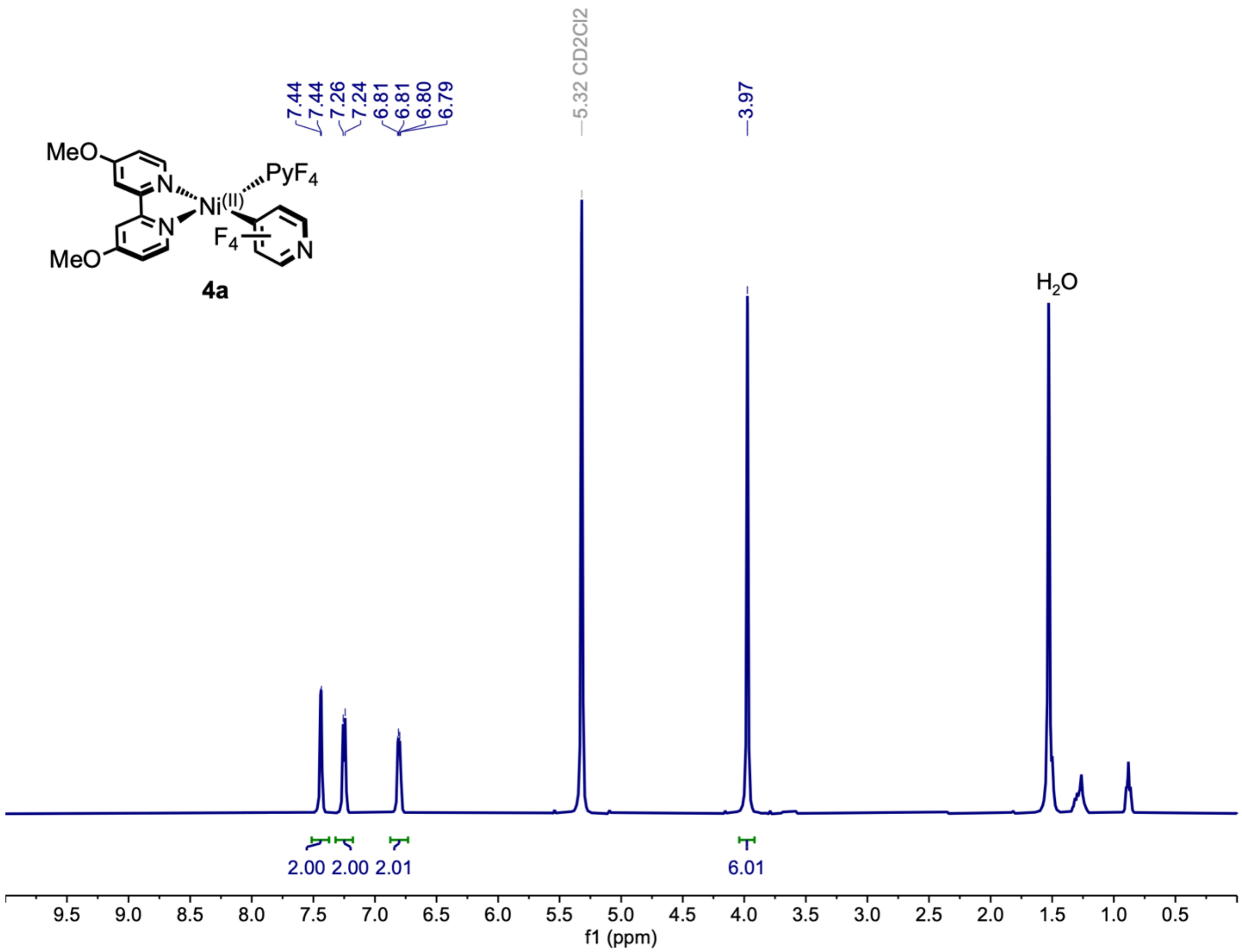

Figure S47. ^{1}H NMR (400 MHz, CD_2Cl_2) spectrum of complex **4a** prepared utilizing method C.

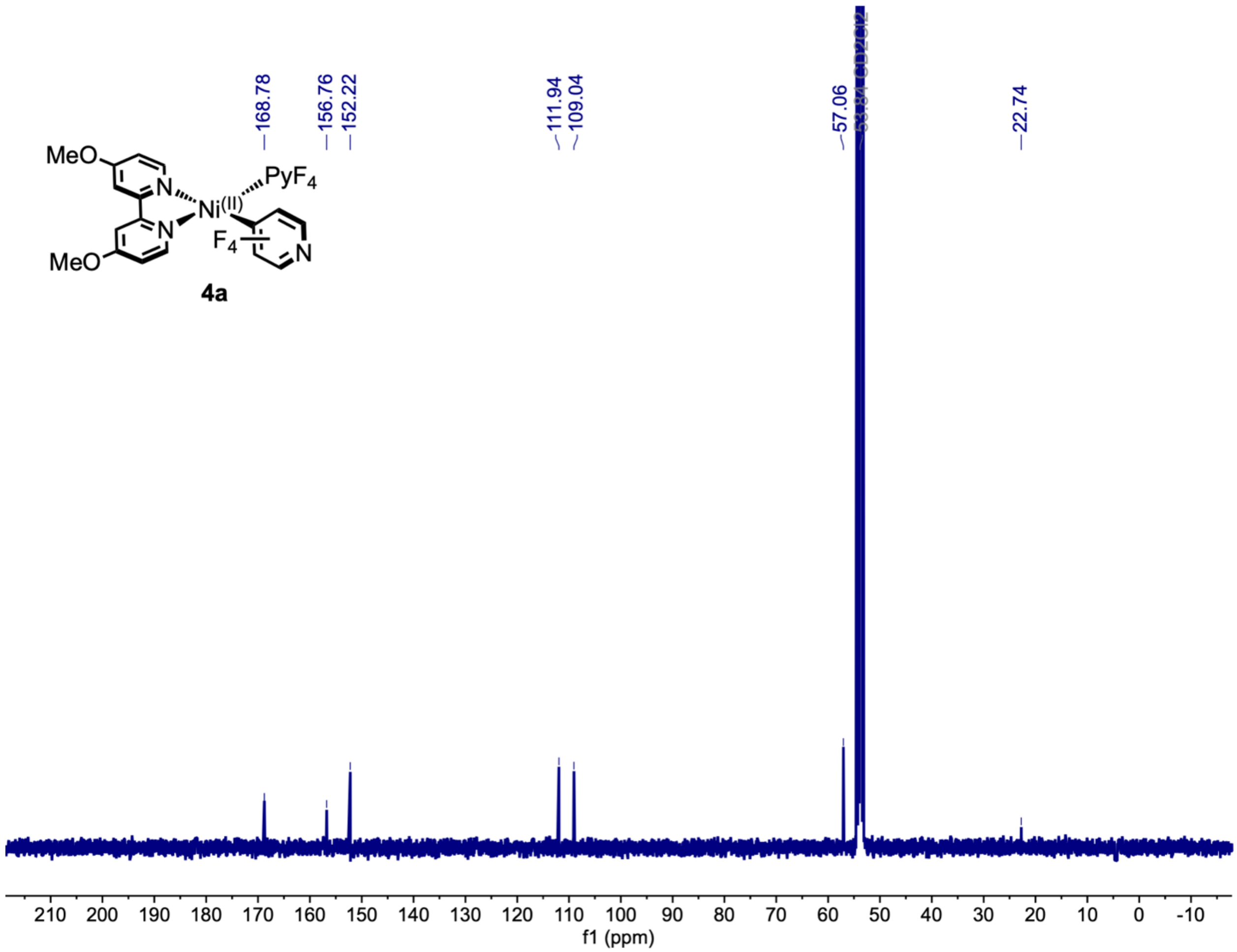

Figure S48. ^{13}C NMR (101 MHz, CD_2Cl_2) spectrum of complex **4a** prepared utilizing method C.

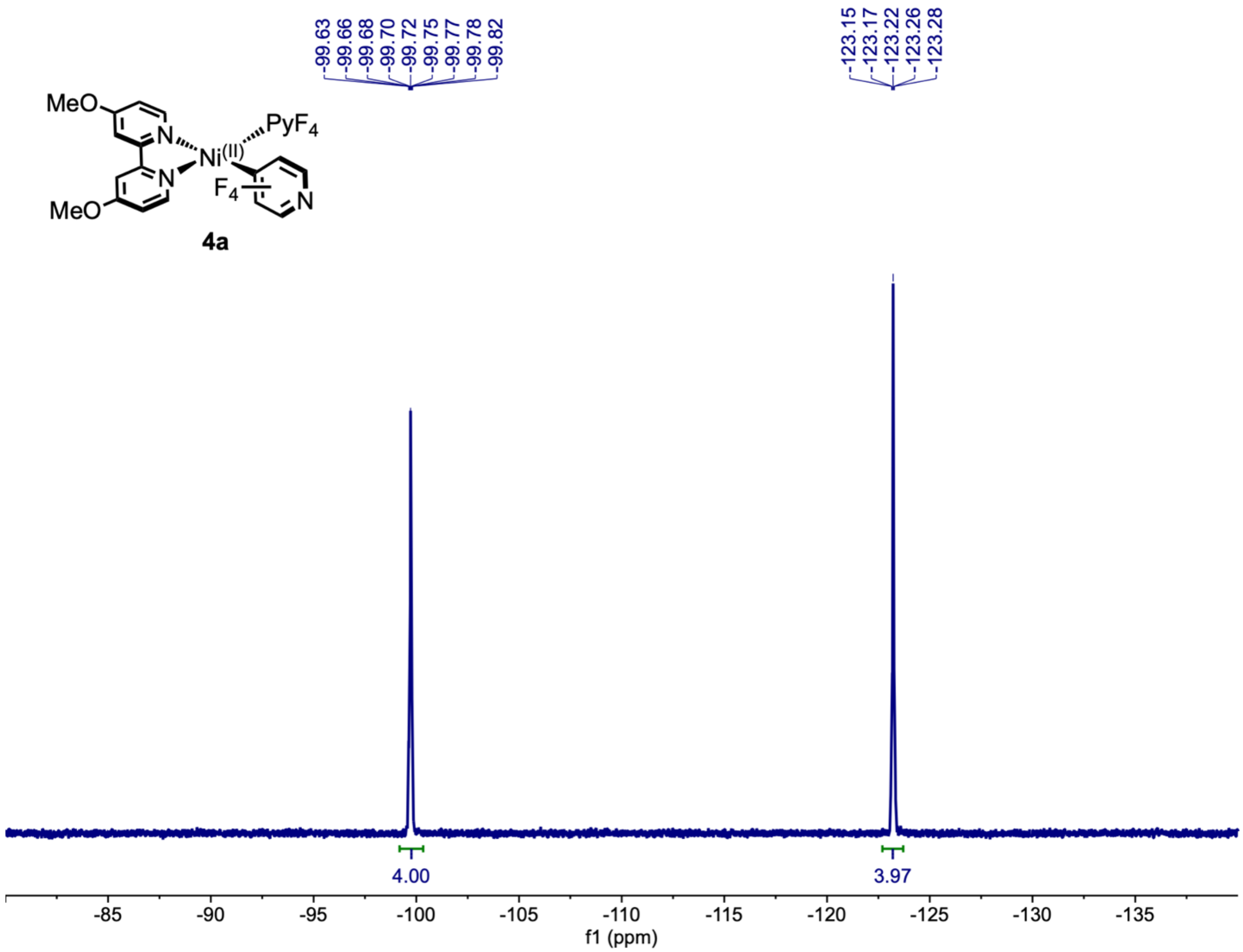

Figure S49. ^{19}F NMR (376 MHz, CD_2Cl_2) spectrum of complex **4a** prepared utilizing method C.

(bpy)Ni(PyF$_4$)$_2$ (4b): Isolated yield of 90%[a]. Bright yellow powder. Poor solubility in DCM and THF. ^{1}H NMR (400 MHz, CD$_2$Cl$_2$): δ 8.15 – 8.00 (m, 4H), 7.51 (m, 2H), 7.40 (m, 2H). ^{1}H NMR (400 MHz, DMSO-*d*$_6$): δ 8.57 (s, 2H), 8.30 (s, 2H), 7.56 (s, 4H). ^{19}F NMR (376 MHz, DMSO): δ -99.18 (m, 4F), -122.61 (m, 4F).

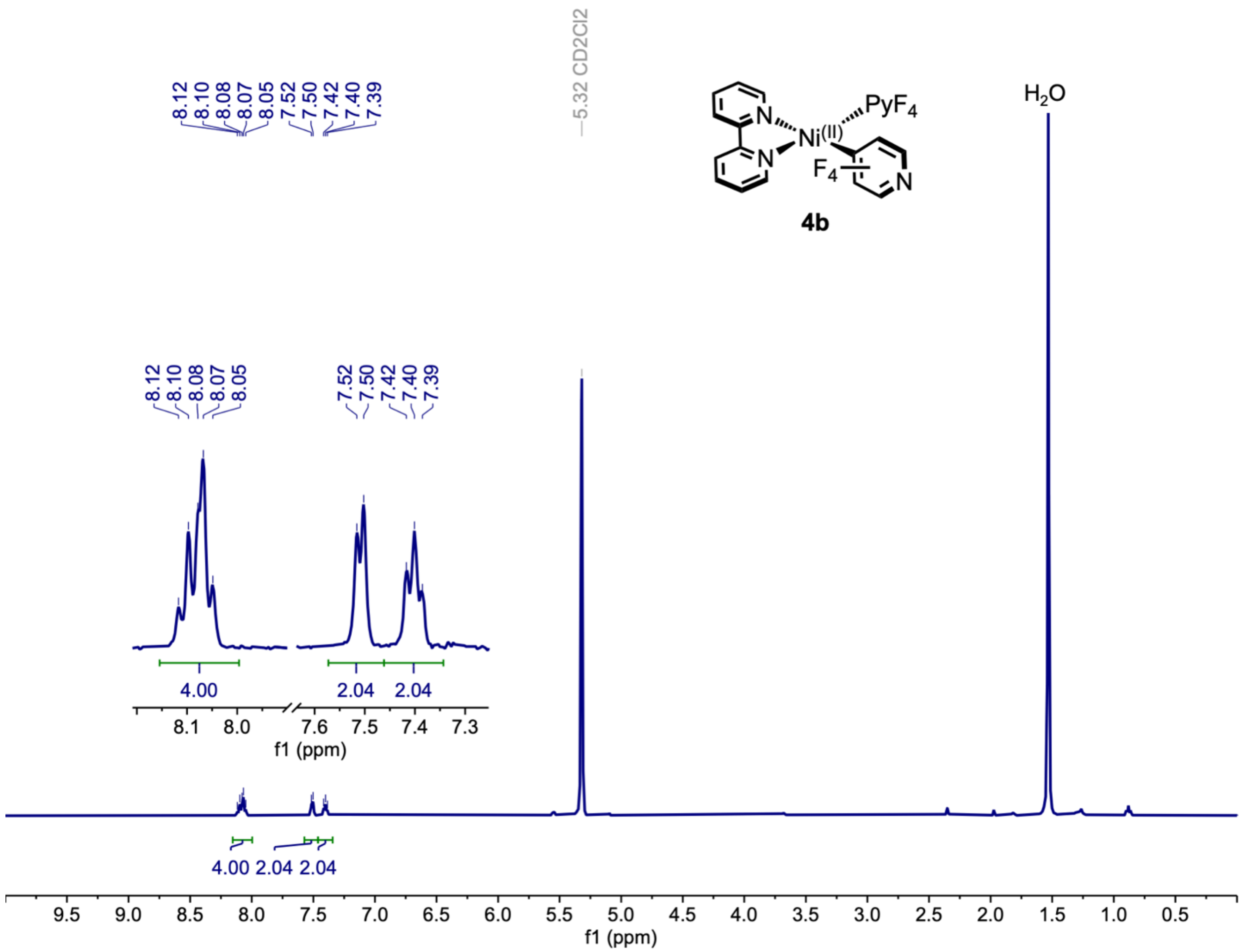

Figure S50. ^{1}H NMR (400 MHz, CD$_2$Cl$_2$) spectrum of complex **4b** prepared utilizing method A.

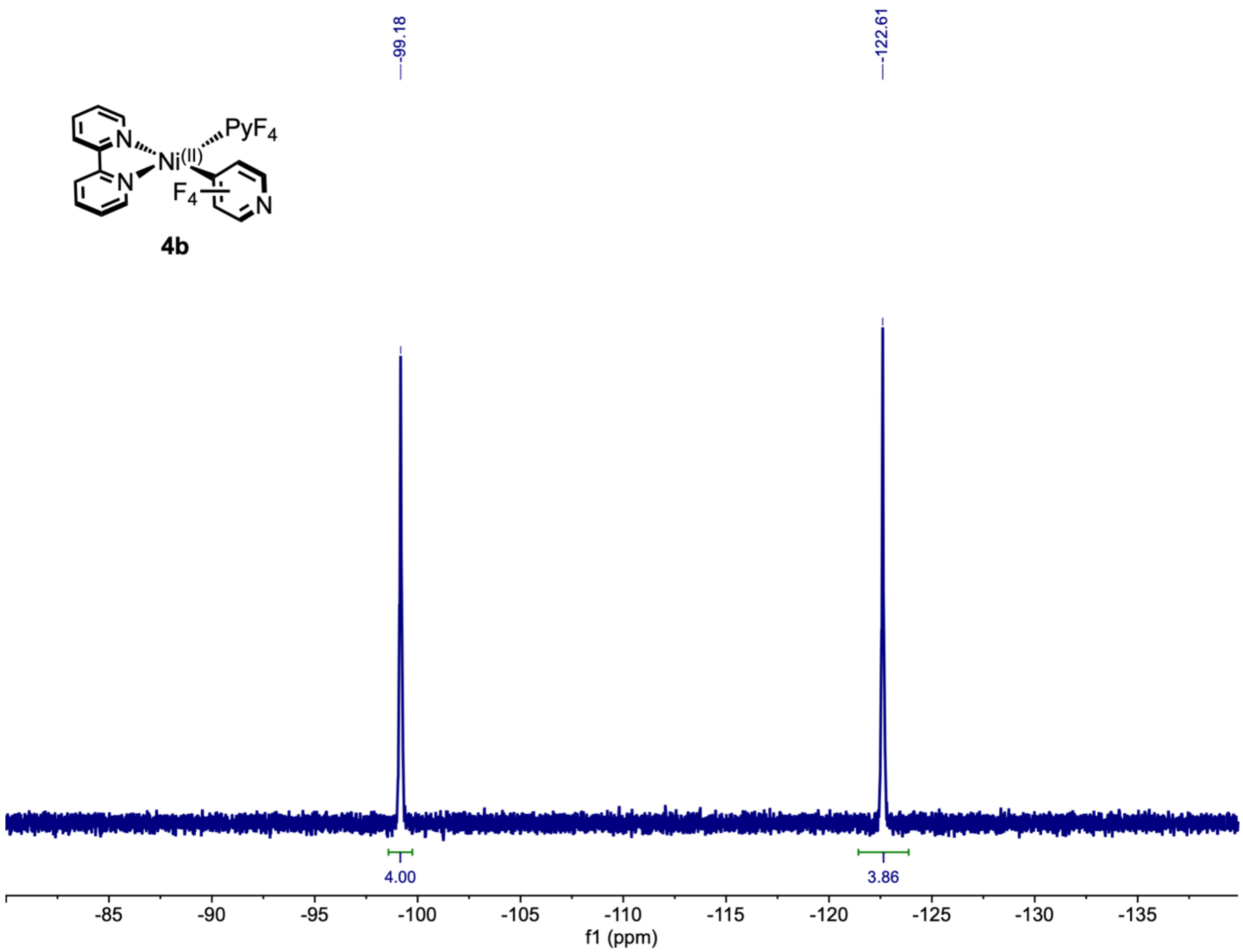

Figure S51. ^{19}F NMR (376 MHz, CD_2Cl_2) spectrum of complex **4b** prepared utilizing method A.

(^{COOMe}bpy)Ni(PyF$_4$)$_2$ (4c): Isolated yield of 55%[c]. Orange powder. ^{1}H NMR (600 MHz, CD_2Cl_2): δ 8.71 (s, 2H), 7.94 (d, *J* = 5.7 Hz, 2H), 7.72 (d, *J* = 5.7 Hz, 2H), 4.04 (s, 6H). ^{13}C NMR (101 MHz, CD_2Cl_2): δ 163.69 – 163.53 (m), 155.60, 151.93, 141.63, 127.44, 121.85. ^{19}F NMR (376 MHz, CD_2Cl_2): δ -98.65 (m, 4F), -123.23 (m, 4F).

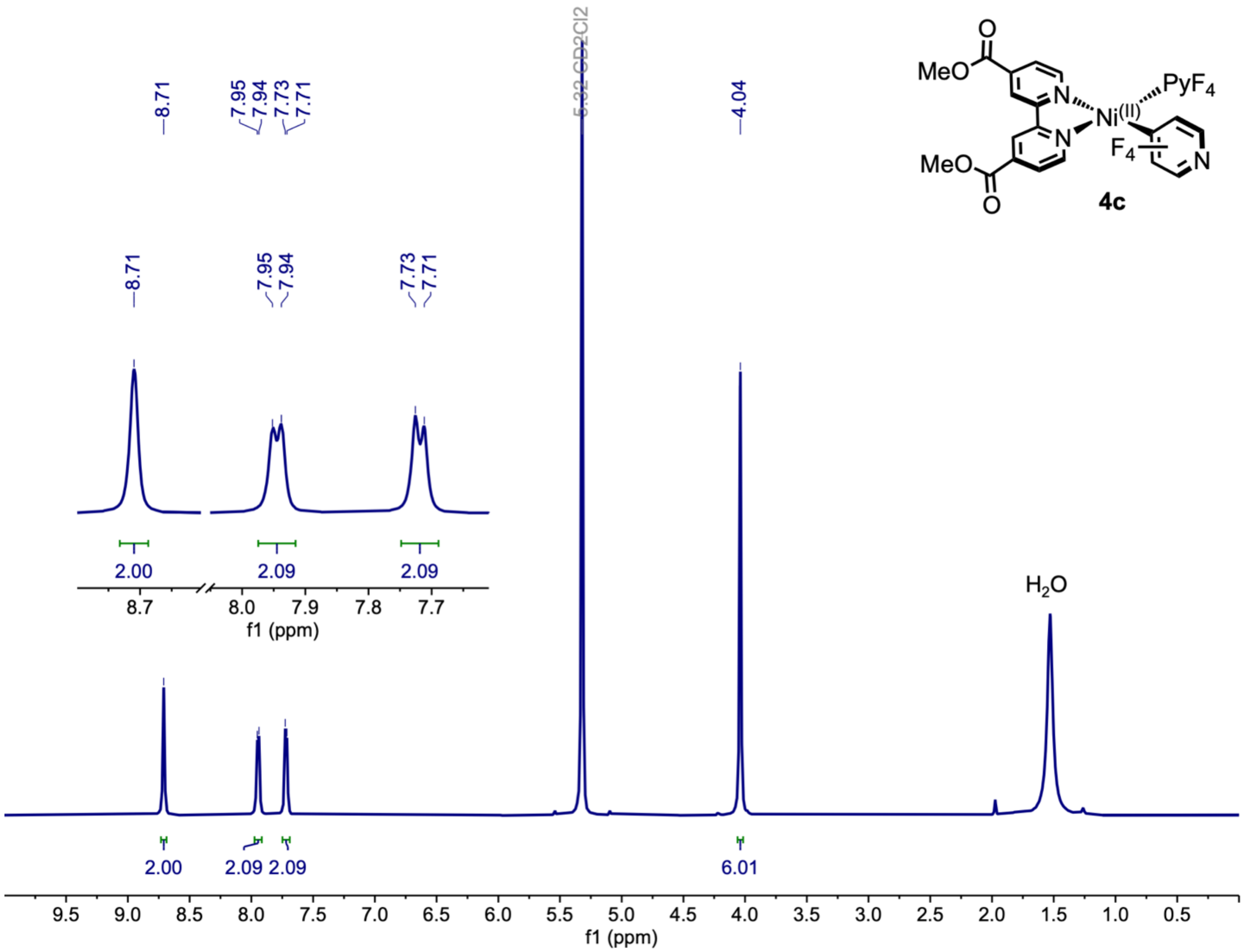

Figure S52. ^{1}H NMR (600 MHz, CD_2Cl_2) spectrum of complex **4c** prepared utilizing method C.

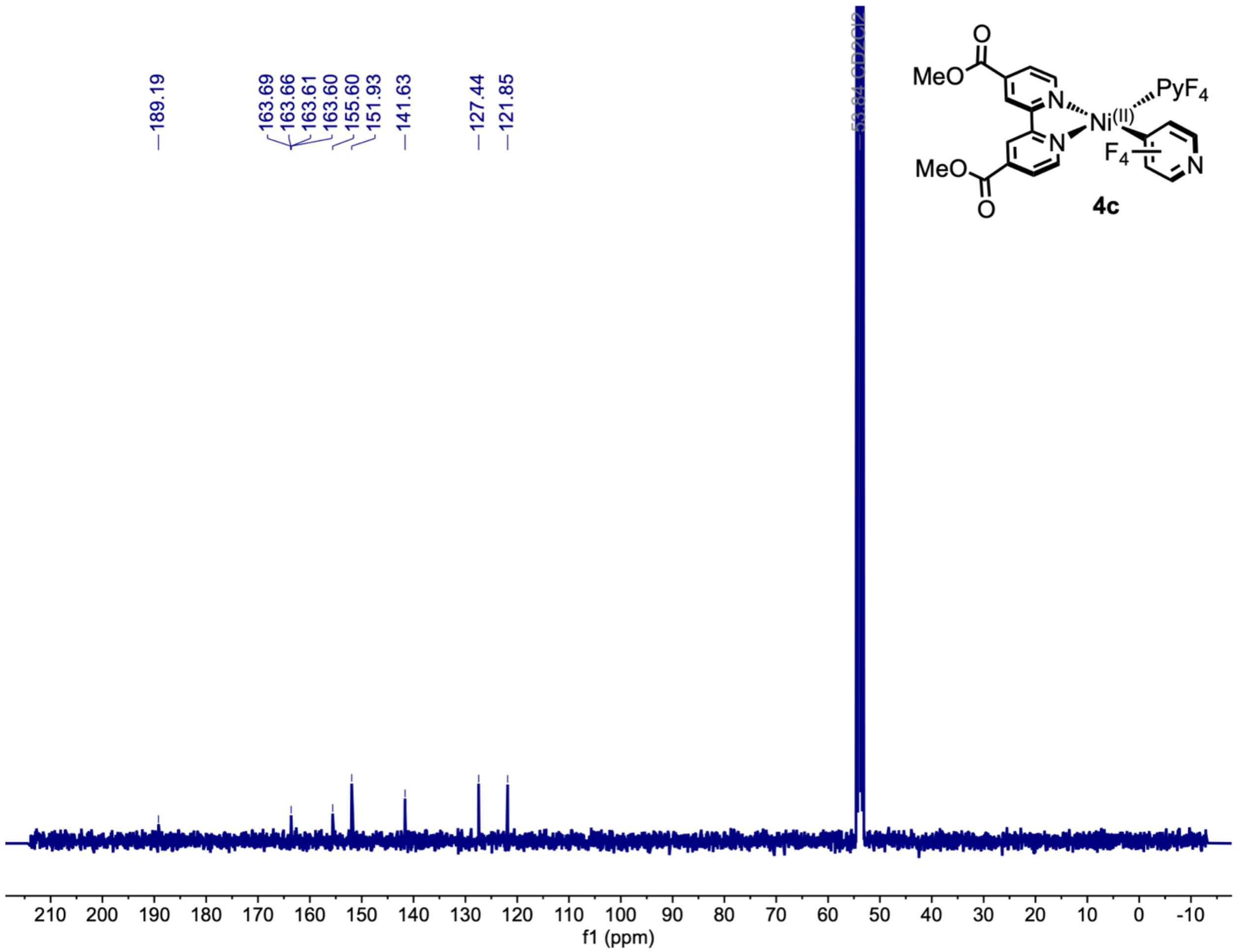

Figure S53. ^{13}C NMR (101 MHz, CD_2Cl_2) spectrum of complex **4c** prepared utilizing method C.

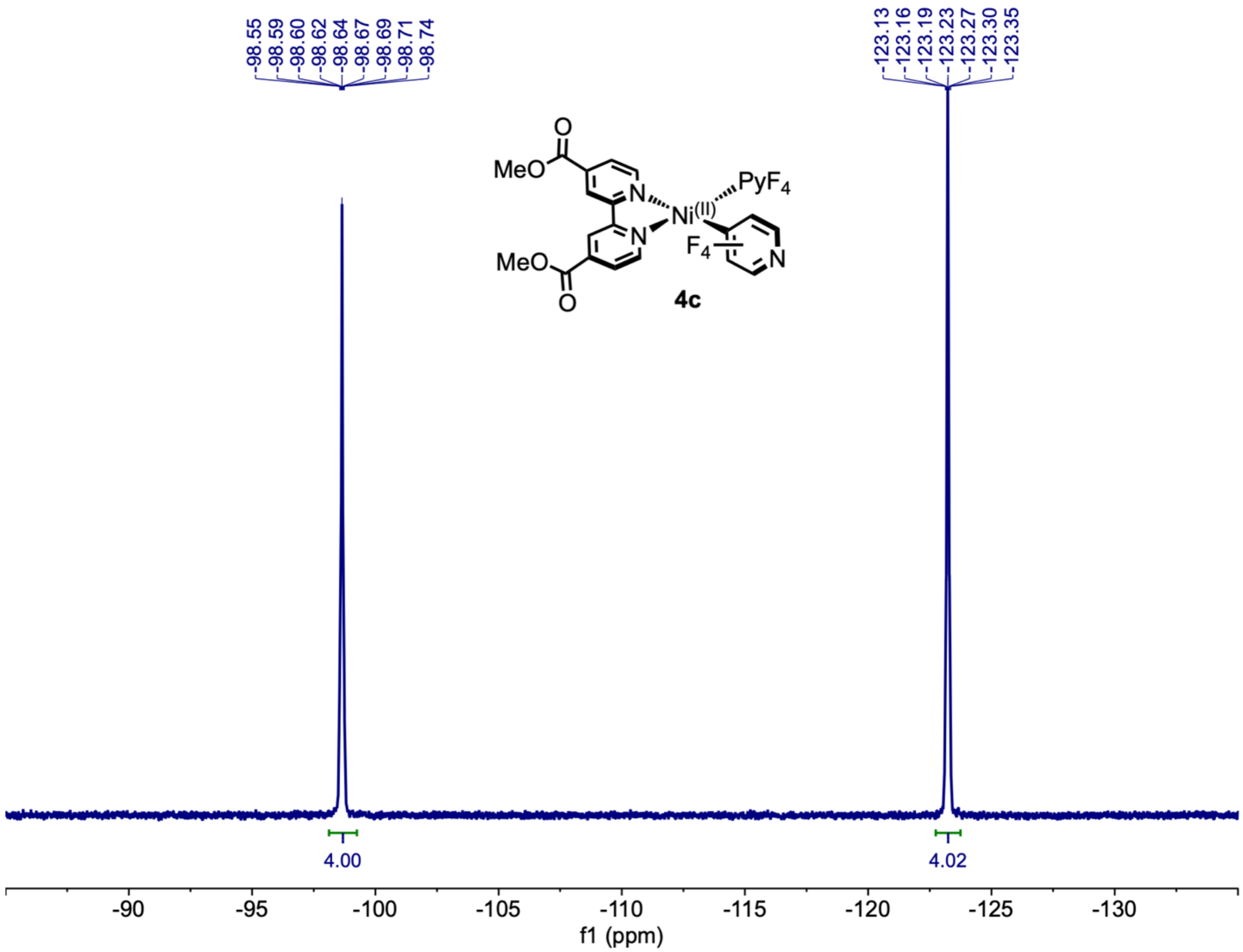

Figure S54. ^{19}F NMR (376 MHz, CD_2Cl_2) spectrum of complex **4c** prepared utilizing method C.

(^{CF_3}bpy)Ni(PyF$_4$)$_2$ (4d): Isolated yield of 44%[c]. Light orange powder. Poor solubility in DCM and THF. ^{1}H NMR (400 MHz, DMSO-d_6)**:** δ 9.29 (d, 2H), 7.95 – 7.83 (m, 4H). ^{19}F NMR (376 MHz, CD_2Cl_2): δ -63.56 (s, 6F), -98.76 (m, 4F), -122.32 – -123.37 (m, 4F).

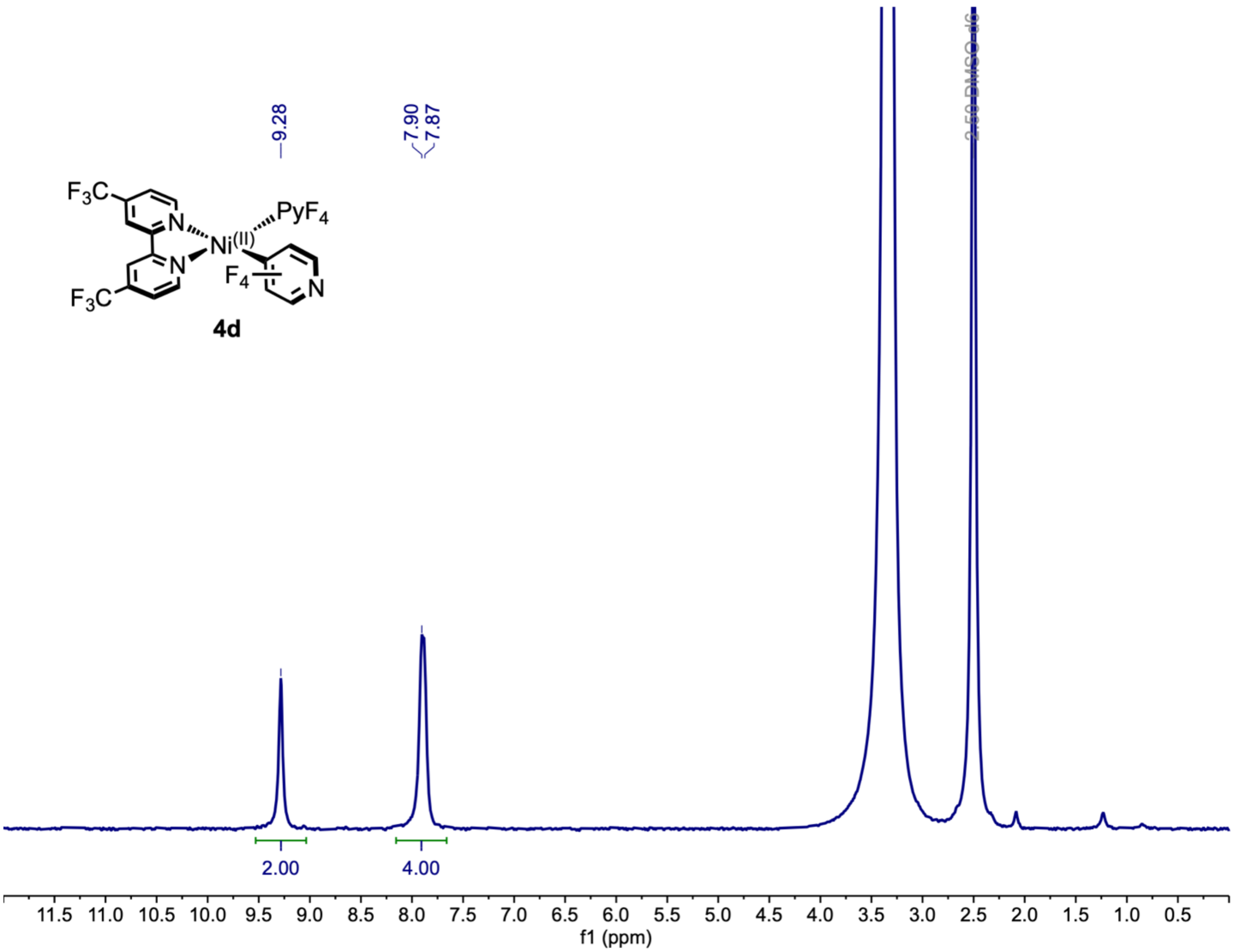

Figure S55. ^{1}H NMR (400 MHz, DMSO-d_6) spectrum of complex **4d** prepared utilizing method C.

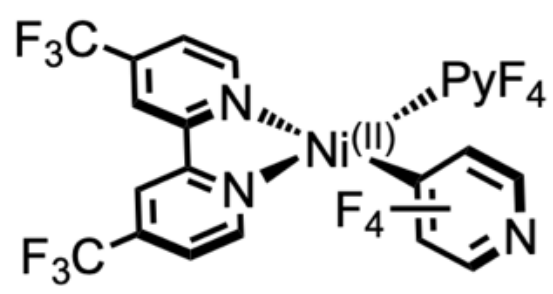

4d

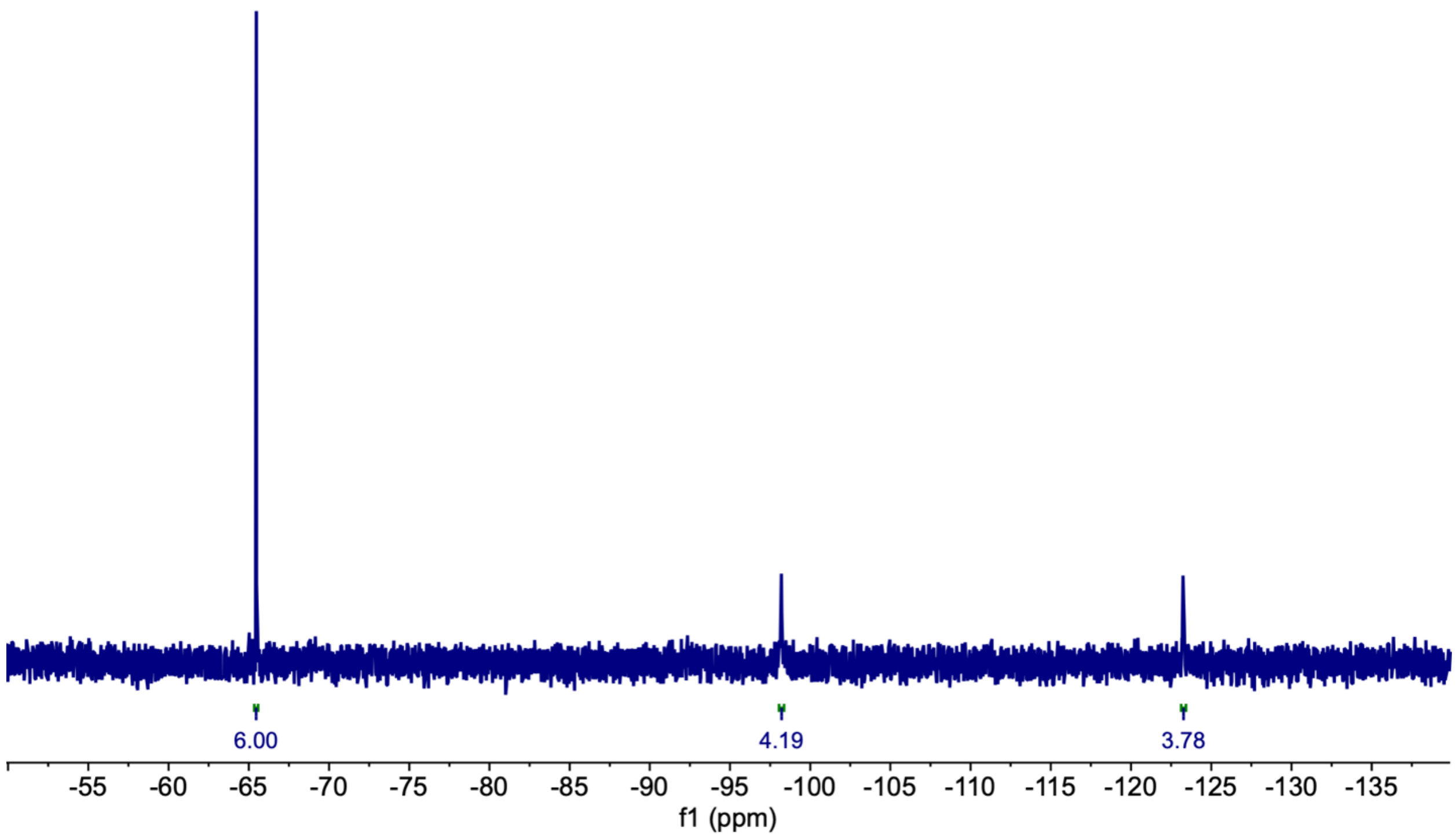

Figure S56. ^{19}F NMR (376 MHz, CD_2Cl_2) spectrum of complex **4d** prepared utilizing method C.

4 Thermal experiments

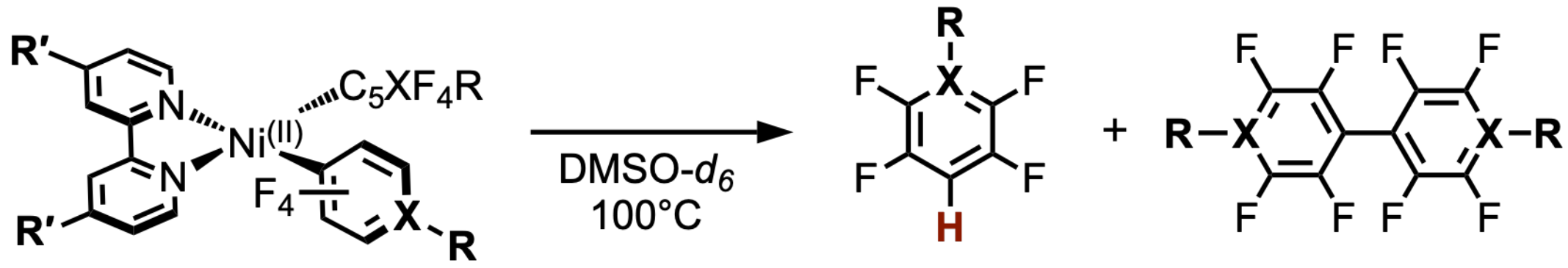

R′ = *t*-Bu (**3a** or **3d**), CF_3 (**4d**)

X = N; **R** = *none* (**3a** or **4d**)
X = C; **R** = CO_2Me (**3d**)

The complex was dissolved in DMSO-d_6 (800 µL) and sonicated for 10 min. A 600 µL portion of the solution was transferred to a J-Young NMR tube, after which 1,3,5-tris(trifluoromethyl)benzene (1.00 µL, 5.4 µmol) was added as an internal standard. Initial $t = 0$ ^{1}H and ^{19}F NMR spectra were collected, and the J-Young tube was then placed in a 100 °C silicone oil bath.

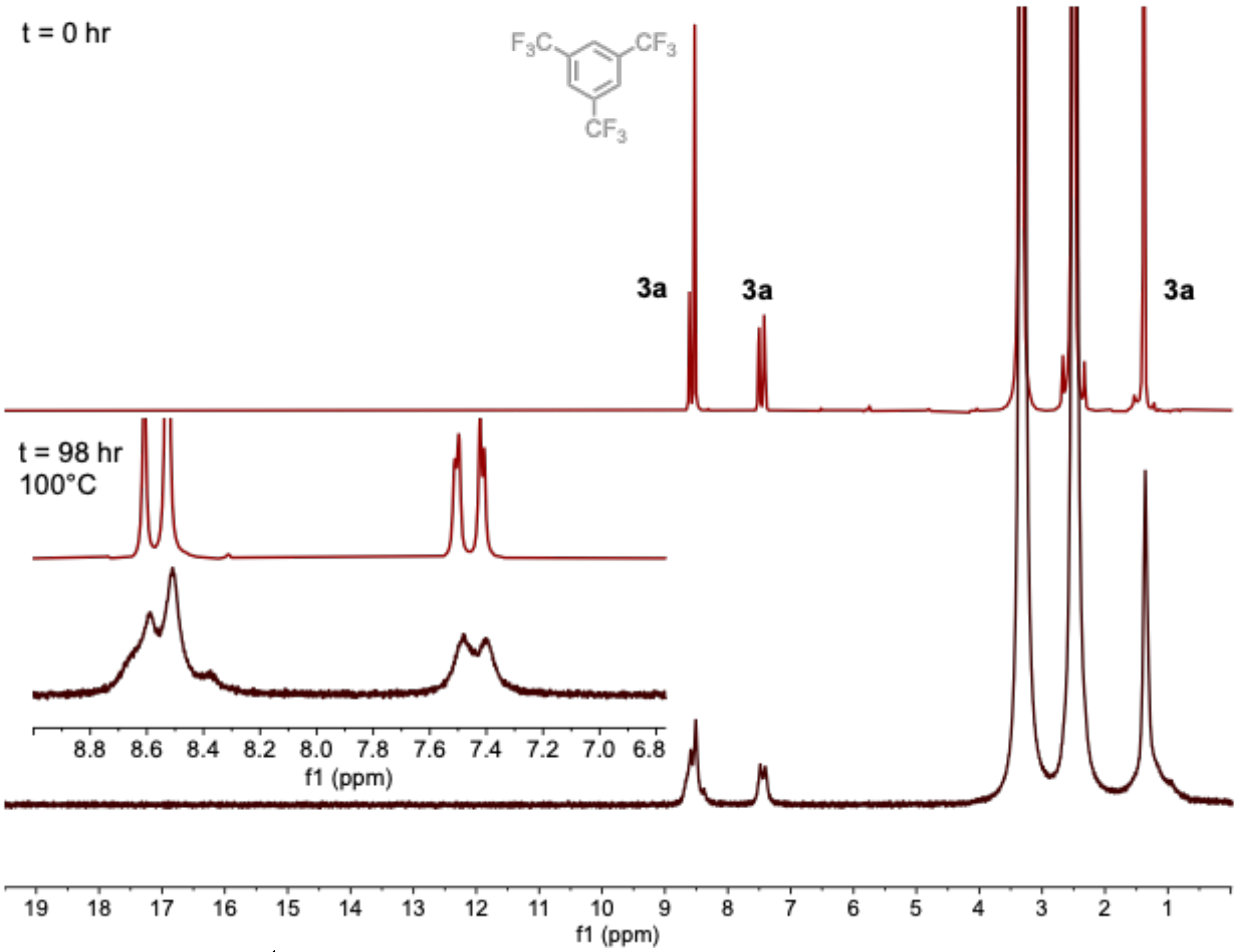

Figure S57. Stacked ^{1}H NMR (400 MHz, DMSO-d_6) spectra of complex **3a** heated to 100 °C in a J-Young NMR tube. Internal standard is tris(trifluoromethyl)benzene.

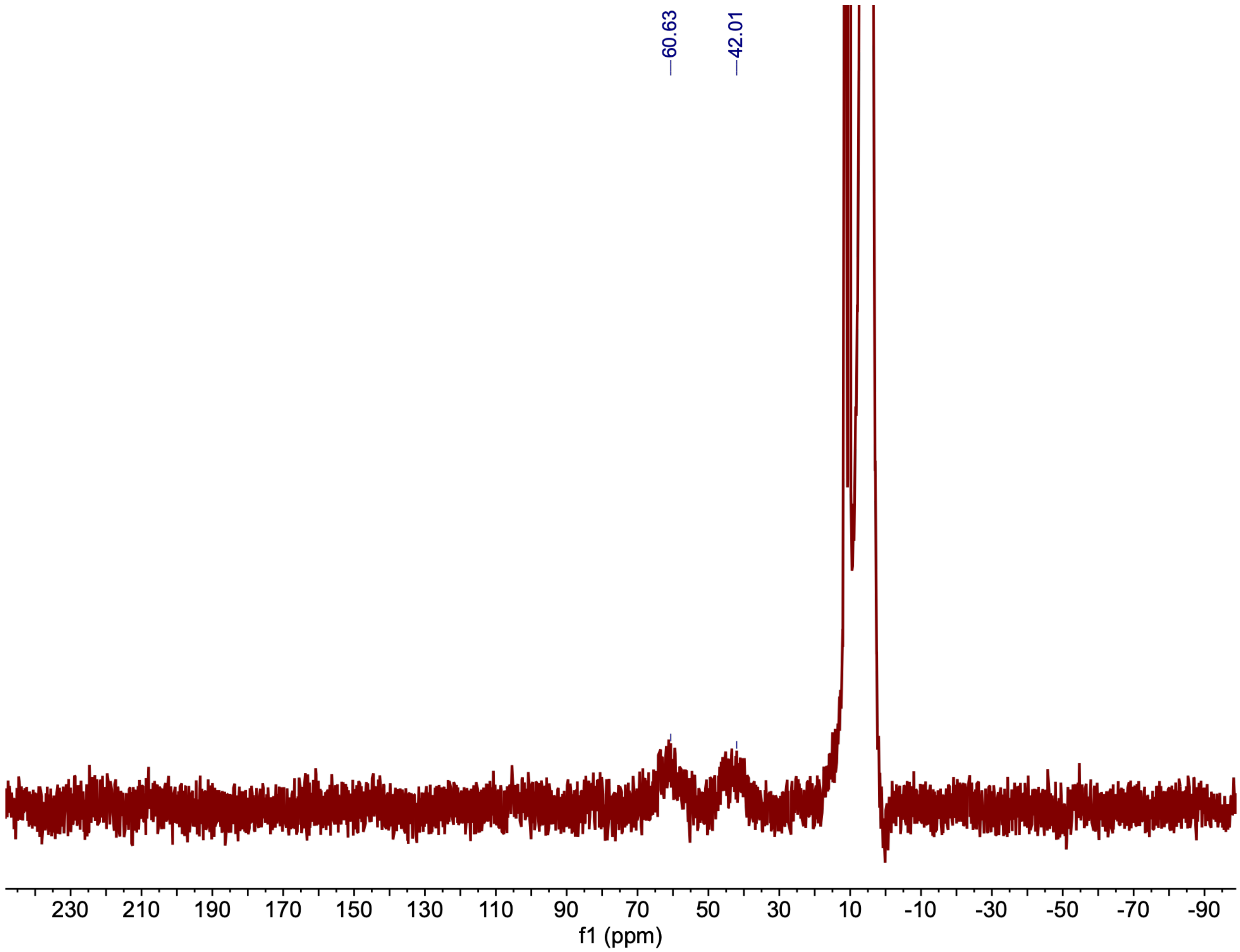

Figure S58. ^{1}H NMR (400 MHz, DMSO-d_6) spectrum of complex **3a** heated to 100 °C in a J-Young NMR tube after 98 hours. Internal standard is tris(trifluoromethyl)benzene.

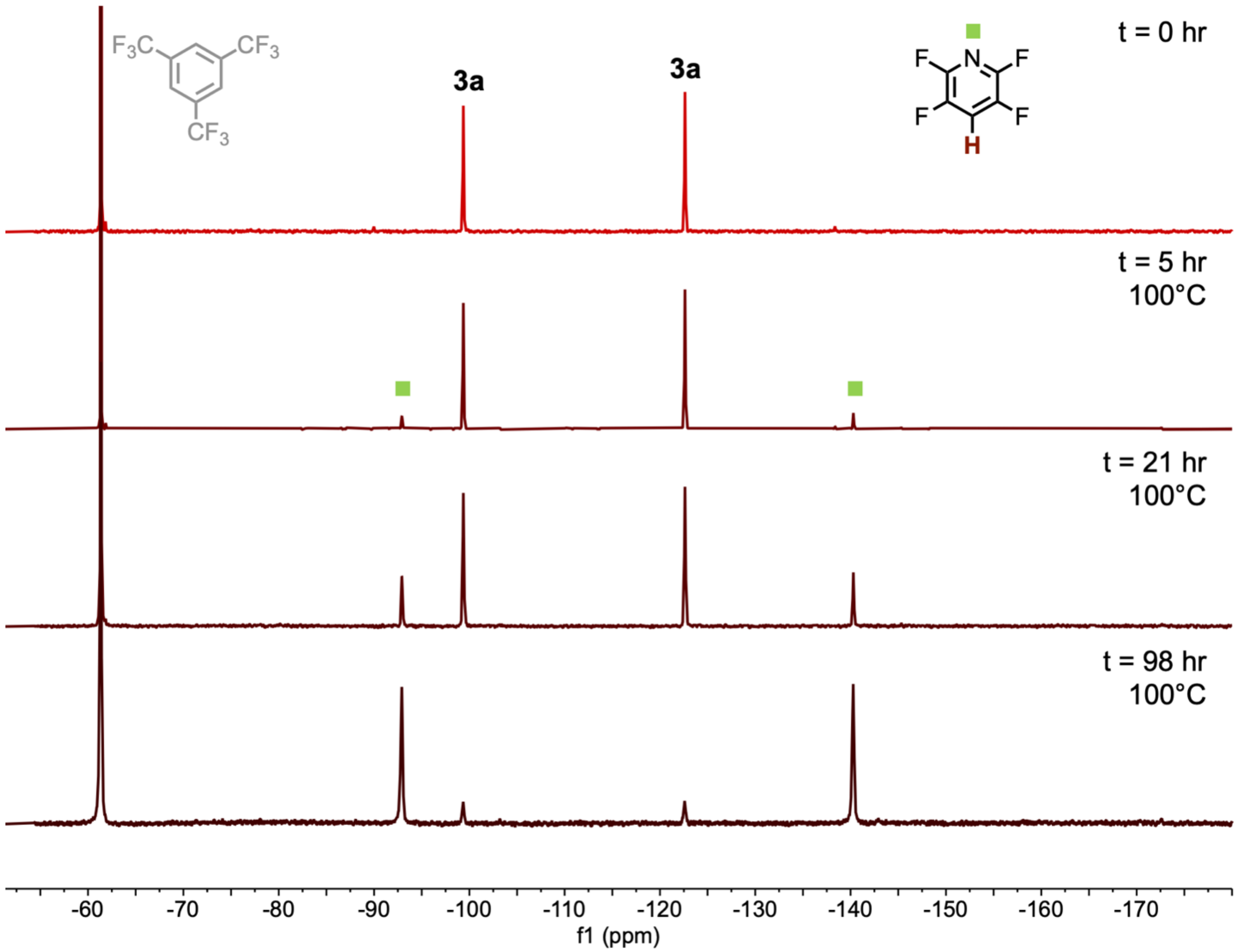

Figure S59. Array of ^{19}F NMR (376 MHz, DMSO-d_6) spectra of complex **3a** heated to 100 °C in a J-Young NMR tube. Internal standard is tris(trifluoromethyl)benzene.

Table S2. ^{19}F NMR-derived integrations, μmol, and yields for 3a and thermal reaction products over time.

Time (hr)	^{19}F NMR integration			μmol			Yield (%)		
	**3a**	HC	HDF	**3a**	HC	HDF	**3a**	HC	HDF
0	0.26	0.03	0.00	3.14	0.36	0.00	45%	5%	0%
0.5	0.47	0.03	0.00	5.68	0.36	0.00	81%	5%	0%
1	0.49	0.02	0.03	5.92	0.24	0.72	84%	3%	5%
2	0.51	0.03	0.04	6.16	0.36	0.97	88%	5%	7%
5	0.45	0.01	0.06	5.43	0.12	1.45	78%	2%	10%
21	0.35	0.01	0.14	4.23	0.12	3.38	60%	2%	24%
27.5	0.33	0.00	0.17	3.98	0.00	4.11	57%	0%	29%
48.5	0.30	0.00	0.22	3.62	0.00	5.31	52%	0%	38%
98	0.08	0.00	0.41	0.97	0.00	9.90	14%	0%	71%
168	0.00	0.00	0.45	0.00	0.00	10.87	0%	0%	78%

Quantification was performed by integration of diagnostic ^{19}F NMR resonances using tris(trifluoromethyl)benzene as an internal standard (integration set to 1.00, 9 F). Integrations correspond to the following chemical shift regions: **3a** (–99.36, –122.61 ppm), HC = PyF_4–PyF_4 (–90.00, –139.37 ppm), and HDF = PyF_4H (–92.92, –140.28 ppm). μmol for each compound were calculated by correcting for the number of fluorine nuclei contributing to each signal. The reaction was initiated with 7.0 μmol of **3a**. The apparent increase in **3a** integration at early time points is attributed to incomplete initial dissolution in DMSO-d_6, resulting in progressive solubilization and more accurate quantification over time.

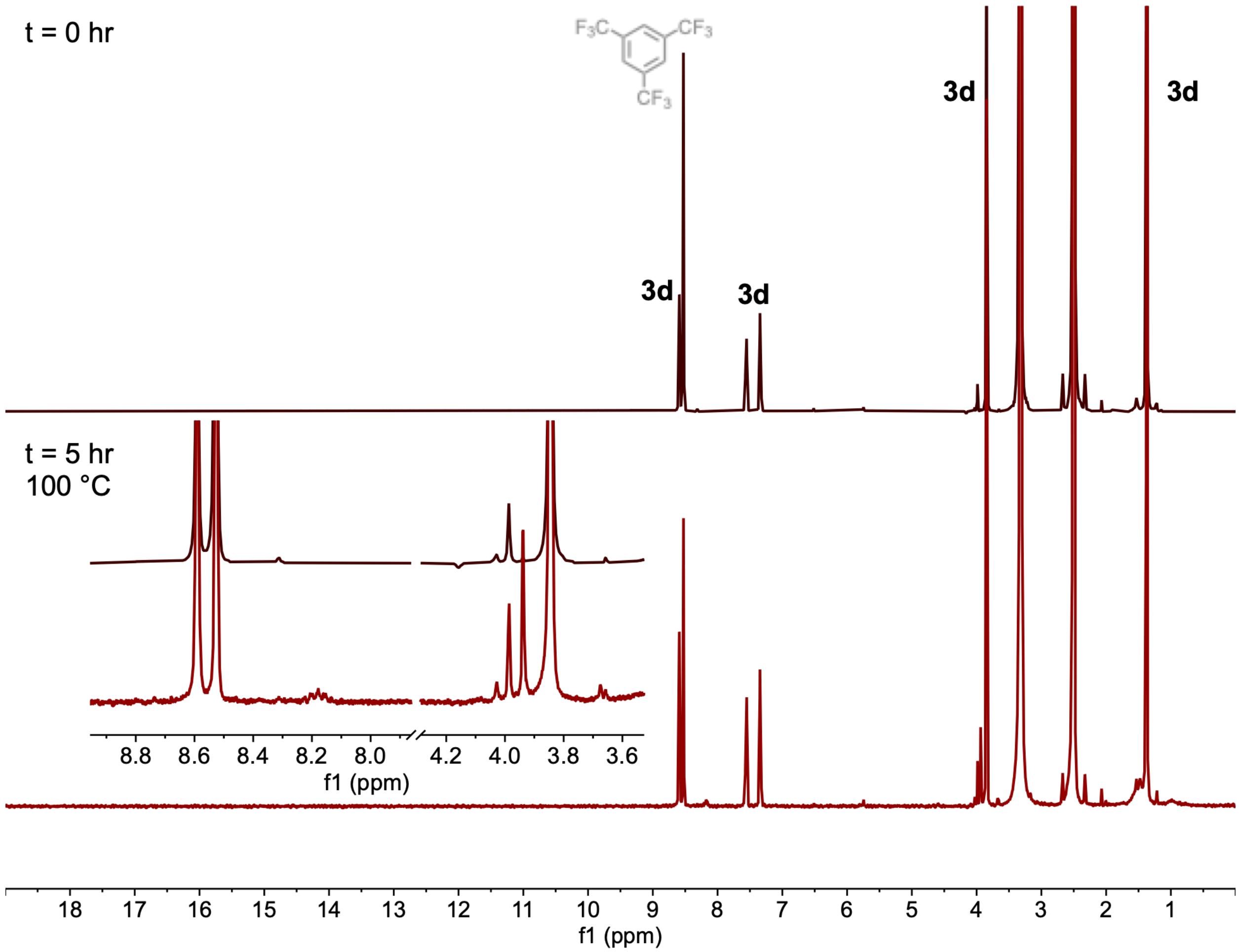

Figure S60. Stacked ^1H NMR (400 MHz, DMSO-d_6) spectra of complex **3d** heated to 100 °C in a J-Young NMR tube. Internal standard is tris(trifluoromethyl)benzene.

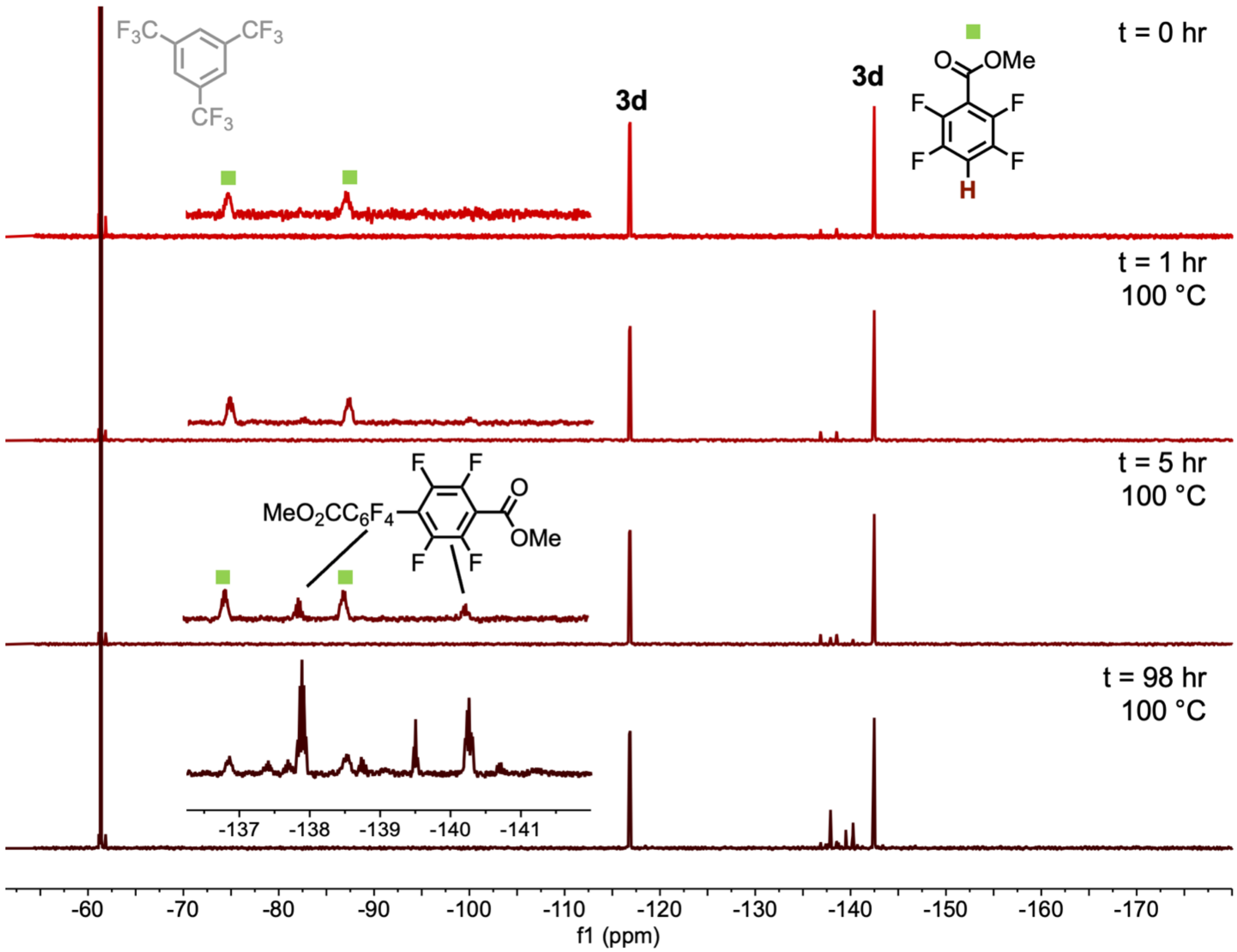

Figure S61. Array of ^{19}F NMR (376 MHz, DMSO-d_6) spectra of complex **3d** heated to 100 °C in a J-Young NMR tube. Internal standard is tris(trifluoromethyl)benzene.

Table S3. ^{19}F NMR-derived integrations, µmol, and yields for 3d and thermal reaction products over time.

	^{19}F NMR integration			µmol			Yield (%)		
	**3d**	HC	HDF	**3d**	HC	HDF	**3d**	HC	HDF
0	0.30	0.00	0.02	3.62	0.00	0.48	52%	0%	3%
0.5	0.57	0.00	0.03	6.88	0.00	0.72	98%	0%	5%
1	0.57	0.01	0.03	6.88	0.12	0.72	98%	2%	5%
2	0.57	0.01	0.03	6.88	0.12	0.72	98%	2%	5%
5	0.57	0.02	0.04	6.88	0.24	0.97	98%	3%	7%
21	0.54	0.03	0.03	6.52	0.36	0.72	93%	5%	5%
27.5	0.54	0.03	0.03	6.52	0.36	0.72	93%	5%	5%
48.5	0.52	0.05	0.02	6.28	0.60	0.48	90%	9%	3%
98	0.49	0.07	0.02	5.92	0.85	0.48	84%	12%	3%

Quantification was performed by integration of diagnostic ^{19}F NMR resonances using tris(trifluoromethyl)benzene as an internal standard (integration set to 1.00, 9 F). Integrations correspond to the following chemical shift regions: **3d** (–116.8, –142.5 ppm), HC = MeO_2CF_4Ph–PhF_4CO_2Me (–136, –138 ppm), and HDF = PhF_4HCO_2Me (–137, –139 ppm). µmol for each compound were calculated by correcting for the number of fluorine nuclei contributing to each signal. The reaction was initiated with 7.4 µmol of **3d**. The apparent increase in **3d** integration at early time points is attributed to incomplete initial dissolution in DMSO-d_6, resulting in progressive solubilization and more accurate quantification over time.

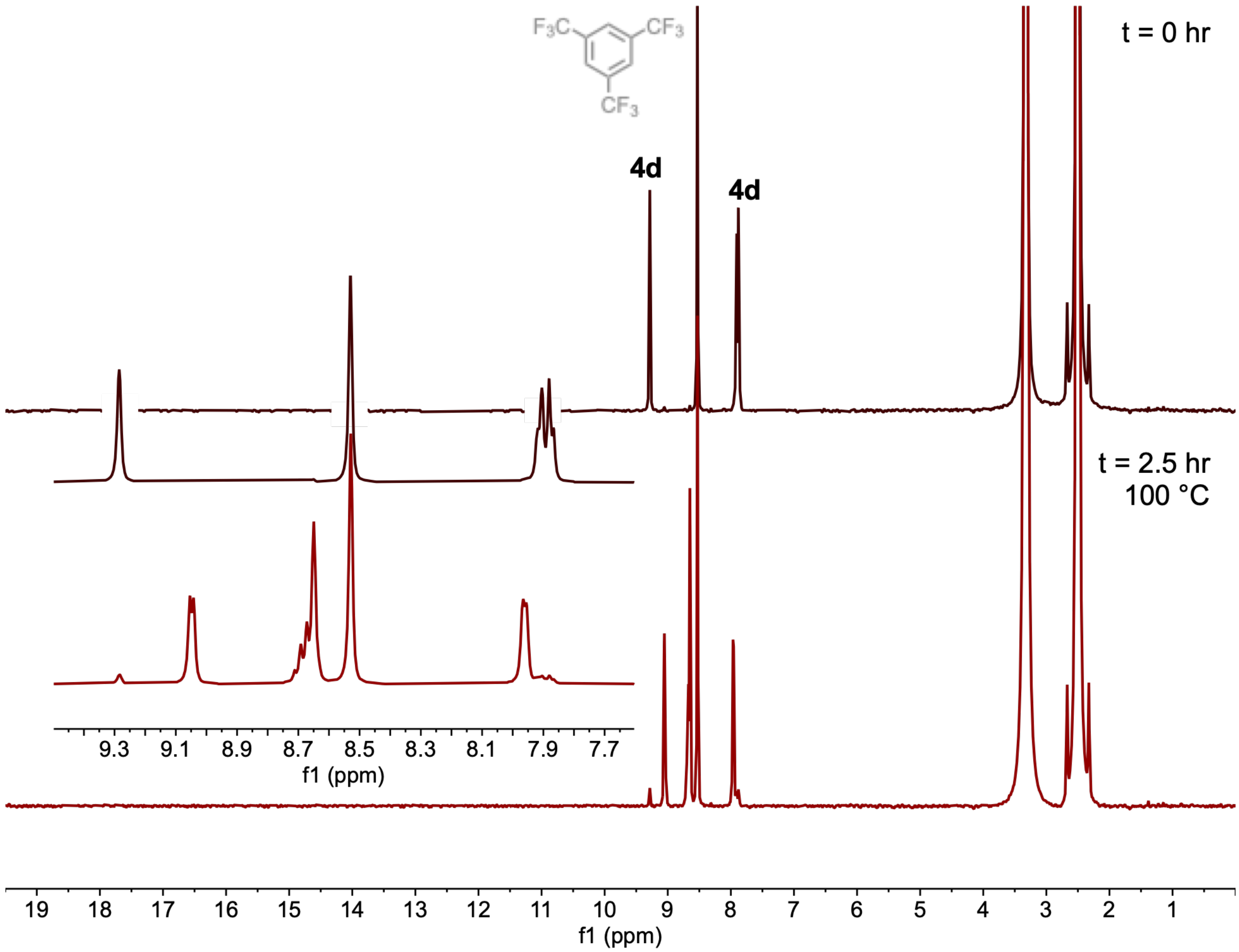

Figure S62. Stacked ^{1}H NMR (400 MHz, DMSO-d_6) spectra of complex **4d** heated to 100 °C in a J-Young NMR tube. Internal standard is tris(trifluoromethyl)benzene.

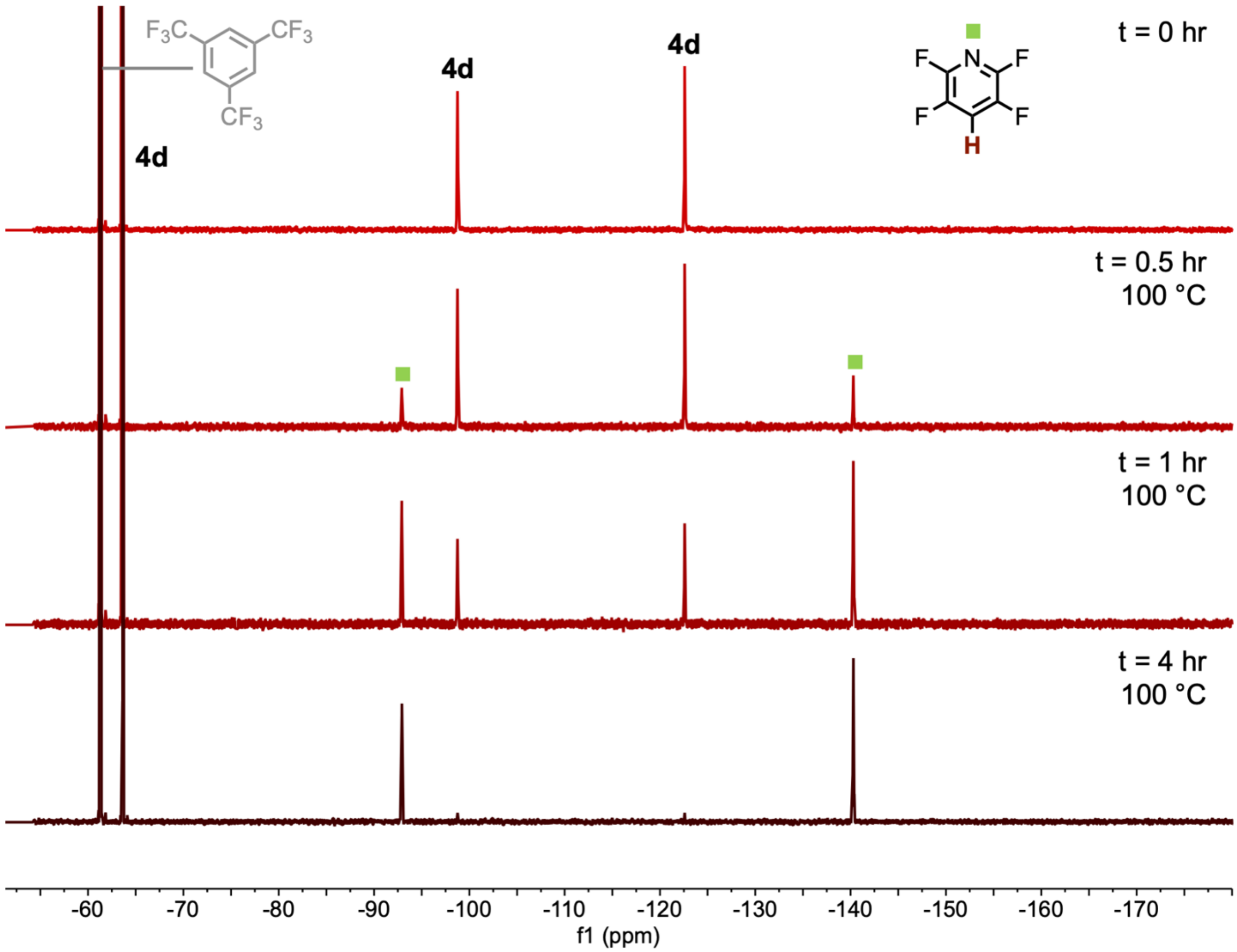

Figure S63. Array of ^{19}F NMR (376 MHz, DMSO-d_6) spectra of complex **4d** heated to 100 °C in a J-Young NMR tube. Internal standard is tris(trifluoromethyl)benzene.

Table S4. ^{19}F NMR-derived integrations, µmol, and yields for 4d and thermal reaction products over time.

Time (hr)	^{19}F NMR integration		µmol		Yield (%)	
	**4d**	HDF	**4d**	HDF	**4d**	HDF
0	0.45	0.02	5.43	0.48	96%	4%
0.5	0.34	0.12	4.11	2.90	72%	26%
1	0.18	0.26	2.17	6.28	38%	55%
2	0.06	0.39	0.72	9.42	13%	83%
2.5	0.04	0.38	0.48	9.18	9%	81%
3	0.04	0.38	0.48	9.18	9%	81%
4	0.03	0.41	0.36	9.90	6%	87%

Quantification was performed by integration of diagnostic ^{19}F NMR resonances using tris(trifluoromethyl)benzene as an internal standard (integration set to 1.00, 9 F). Integrations correspond to the following chemical shift regions: **4d** (–63.56, –99.36, –122.61 ppm), and HDF = PyF_4H (–92.92, –140.28 ppm). µmol for each compound were calculated by correcting for the number of fluorine nuclei contributing to each signal. The reaction was initiated with 5.7 µmol of **4d**.

4.1 Post-reaction analysis of the hydrogen- and nickel-containing products

The products assigned as PyF_4H were identified from their diagnostic ^{19}F NMR chemical shifts and splitting patterns. In particular, the observed splitting is consistent with coupling to hydrogen rather than formation of a deuterated product. Because these products were not isolated and independently characterized, their assignment and the identity of the hydrogen source remain tentative. DMSO-d_6 was not subjected to additional drying, and adventitious water is therefore considered the most plausible proton source under the reaction conditions.

Post-reaction ^1H NMR spectroscopy revealed broad resonances between approximately δ 40 and 60 ppm, consistent with formation of one or more paramagnetic nickel-containing species (**Figure S58**). No visible formation of Ni black was observed. These data indicate that the nickel-containing products remain at least partially soluble and paramagnetic but do not establish their nuclearity, oxidation state, or ligand environment. Formation of a low-valent nickel species following C–C or C–H bond-forming reductive elimination is chemically plausible, although subsequent ligand redistribution, solvent coordination, or redox chemistry cannot be excluded.

5 Photochemical experiments

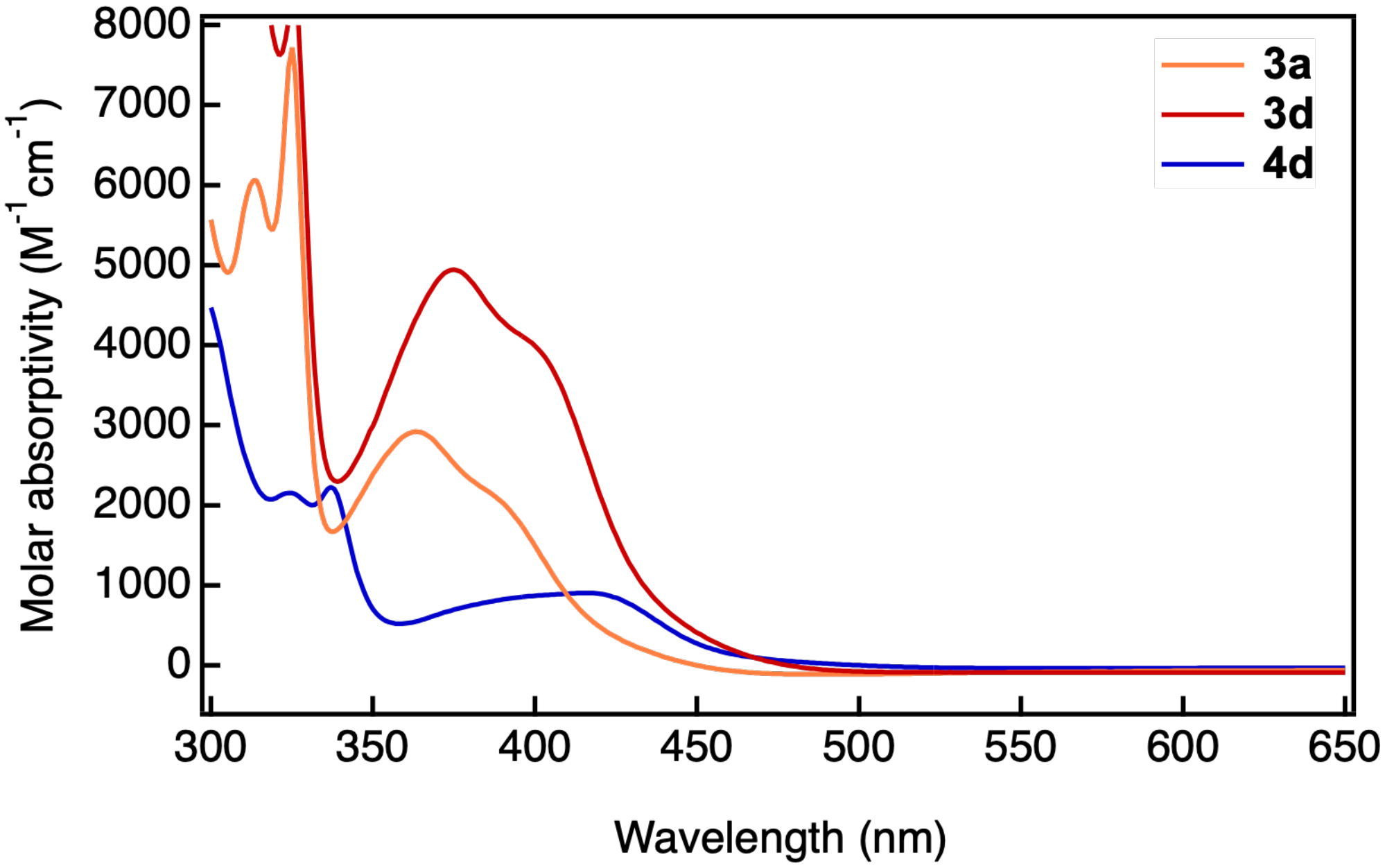

Figure S64. UV-vis absorption spectra for complexes **3a** (orange line), **3d** (red line), and **4d** (blue line) in DMSO.

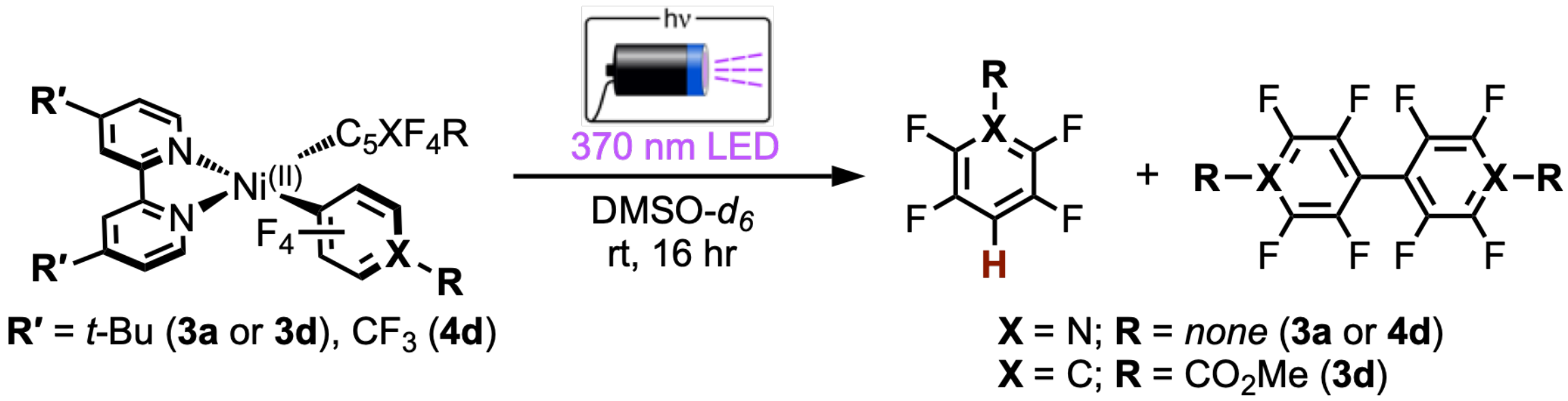

The complex was dissolved in DMSO-d_6 (800 µL) and sonicated for 10 minutes. A 600 µL portion of the solution was transferred to a J-Young NMR tube, after which 1,4-difluorobenzene (DFB; 1.00 µL) was added as an internal standard. Initial t = 0 ^{1}H and ^{19}F NMR spectra were collected, and the J-Young NMR tube was then positioned 5 cm away from a 370 nm Kessil PR160L Gen2 LED operated at maximum power. A cooling fan was directed at the sample during irradiation.

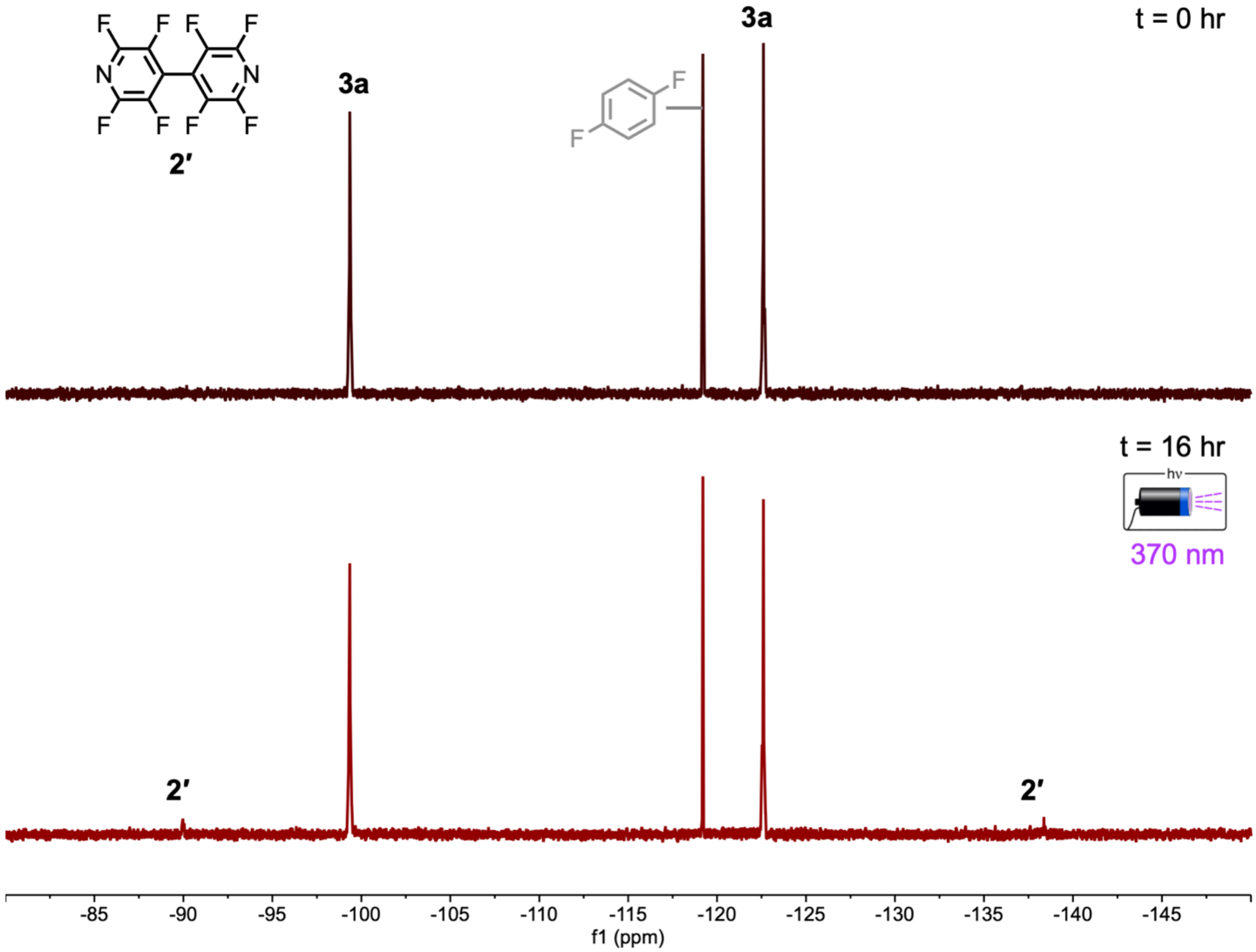

Figure S65. ^{19}F NMR (376 MHz, DMSO-d_6) spectra of complex **3a** irradiated with a 370 nm LED in a J-Young NMR tube. Internal standard is 1,4-difluorobenzene.

Table S5. ^{19}F NMR-derived integrations, µmol, and yields for 3a and photochemical reaction products over time.

Time (hr)	^{19}F NMR integration			µmol			Yield (%)		
	**3a**	HC	HDF	**3a**	HC	HDF	**3a**	HC	HDF
0	2.38	0.00	0.00	11.58	0.00	0.00	100%	0%	0%
16	2.21	0.17	0.00	10.75	0.83	0.00	93%	7%	0%

Quantification was performed by integration of diagnostic ^{19}F NMR resonances using 1,4-difluorobenzene as an internal standard (integration set to 1.00, 2 F). Integrations correspond to the following chemical shift regions: **3a** (–99.36, –122.61 ppm), HC = PyF_4–PyF_4 (–90.00, –139.37 ppm), and HDF = PyF_4H (–92.92, –140.28 ppm). µmol for each compound were calculated by correcting for the number of fluorine nuclei contributing to each signal. The reaction was initiated with 11.6 µmol of **3a**.

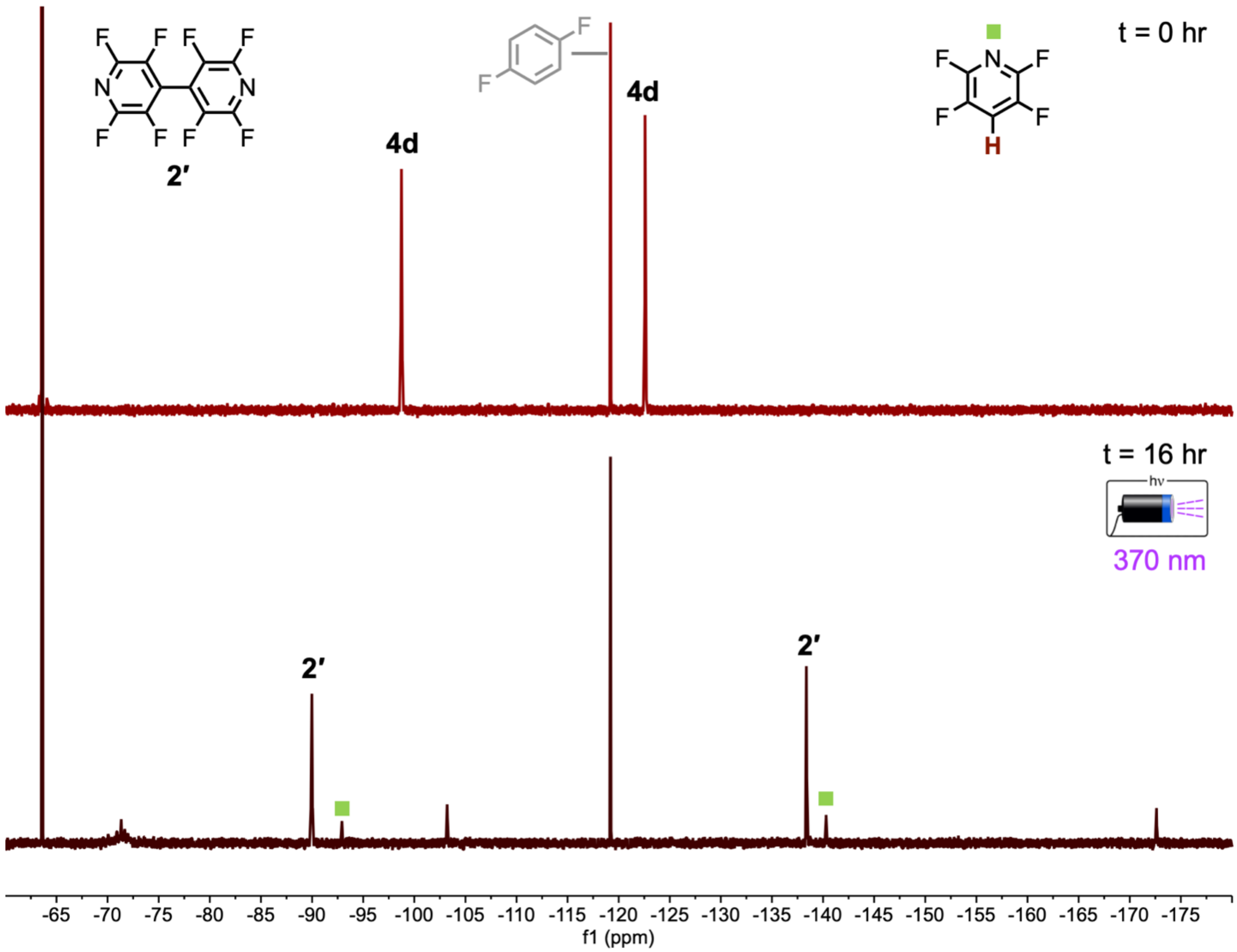

Figure S66. Array of ^{19}F NMR (376 MHz, DMSO-d_6) spectra of complex **4d** irradiated with a 370 nm LED in a J-Young NMR tube. Internal standard is 1,4-difluorobenzene.

Table S6. ^{19}F NMR-derived integrations, μmol, and yields for 4d and photochemical reaction products over time.

Time (hr)	^{19}F NMR integration			μmol			Yield (%)		
	**4d**	HC	HDF	**4d**	HC	HDF	**4d**	HC	HDF
0	1.81	0.00	0.00	8.80	0.00	0.00	100%	0%	0%
16	0.00	1.16	0.2	0.00	5.64	0.97	0%	64%	11%

Quantification was performed by integration of diagnostic ^{19}F NMR resonances using 1,4-difluorobenzene as an internal standard (integration set to 1.00, 2 F). Integrations correspond to the following chemical shift regions: **4d** (–63.56, –99.36, –122.61 ppm), HC = PyF_4–PyF_4 (–90.00, –139.37 ppm), and HDF = PyF_4H (–92.92, –140.28 ppm). μmol for each compound were calculated by correcting for the number of fluorine nuclei contributing to each signal. The reaction was initiated with 8.8 μmol of **4d**.

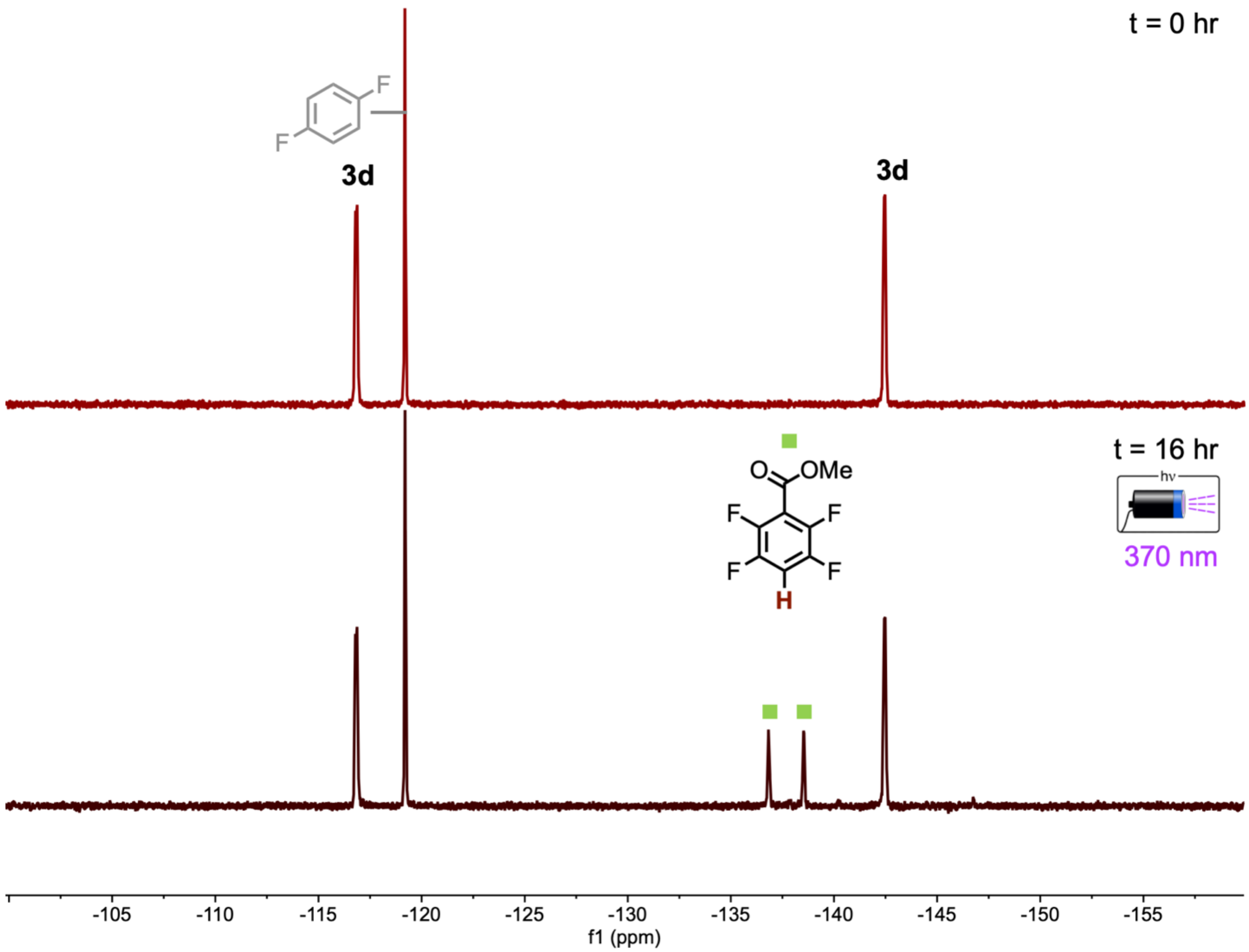

Figure S67. Array of ^{19}F NMR (376 MHz, DMSO-d_6) spectra of complex **3d** irradiated with a 370 nm LED in a J-Young NMR tube. Internal standard is 1,4-difluorobenzene.

Table S7. ^{19}F NMR-derived integrations, µmol, and yields for 3d and photochemical reaction products over time.

Time (hr)	^{19}F NMR integration		µmol		Yield (%)	
	**3d**	HDF	**3d**	HDF	**3d**	HDF
0	1.81	0	4.40	0.00	100%	0%
16	1.13	0.36	2.75	1.75	62%	40%

Quantification was performed by integration of diagnostic ^{19}F NMR resonances using 1,4-difluorobenzene as an internal standard (integration set to 1.00, 2 F). Integrations correspond to the following chemical shift regions: **3d** (–116.8, –142.5 ppm) and HDF = PhF_4HCO_2Me (–137, –139 ppm). µmol for each compound were calculated by correcting for the number of fluorine nuclei contributing to each signal. The reaction was initiated with 4.4 µmol of **3d**.

6 Computational Methods

6.1 General Computational Details

All quantum chemical computations were performed using ORCA version 6.1.0.[21,22] Molecular geometries were optimized and harmonic vibrational-frequency calculations were performed using the hybrid meta-GGA functional TPSSh[23,24] with triple-ζ basis set def2-TZVP[25] on all atoms save Ni, which received the expanded doubly-polarized basis def2-TZVPP. The Weigend auxiliary basis set, def2/J[25] was used, paired with the Becke–Johnson damped atom-pairwise dispersion correction D3(BJ).[26,27] To reduce computational cost, the resolution-of-identity chain-of-spheres approximation, RIJCOSX[28], was utilized throughout as is default for hybrid-DFT in ORCA 6. The conductor-like polarizable continuum solvation model (CPCM)[29] was used for implicit solvation of acetonitrile (MeCN, $\varepsilon = 36.6$).

Restricted Kohn–Sham DFT was used for closed-shell singlet structures identified as RKS in **Table S8**. Unrestricted Kohn–Sham DFT was used for doublet and triplet states, broken-symmetry singlets (BSS), and singlet structures examined for possible open-shell character. UKS singlet calculations with $\langle S^2 \rangle = 0$) converged to spin-unpolarized solutions despite being initiated using an unrestricted formalism. The same functional, basis set, dispersion correction, solvation model, and convergence criteria were used for the RKS and UKS calculations.

Geometry optimizations and harmonic vibrational-frequency calculations were performed using the OPT and FREQ keywords. Tight self-consistent-field convergence criteria (TightSCF) were employed, and SlowConv was used to facilitate SCF convergence. Structures used in the reaction-energy analysis were confirmed as local minima by the absence of imaginary frequencies. XYZ coordinates of optimized geometries are given at the end of this section.

The reported values are electronic energies and do not include zero-point, thermal, entropic, or standard-state corrections. Accordingly, they are denoted (E_{SPE}) and are used only to compare the thermodynamic plausibility of chemically balanced transformations. Reaction energies differences (ΔE_{SPE}) were calculated according to

$$\Delta E_{SPE} = \sum E_{products} - \sum E_{reactants}$$

using a conversion factor of 627.509 kcal mol^{-1} $Hartree^{-1}$. These values are used only to compare the thermodynamic plausibility of chemically balanced transformations and do not represent activation barriers or a complete free-energy profile.

In the following sections, (L) denotes 4,4′-di-tert-butyl-2,2′-bipyridine ($^{t\text{-Bu}}$bpy).

6.2 Calculated Electronic Energies

Table S8. Calculated electronic energies and spin states of species used in the mechanistic analysis

Species	Charge	Multiplicity	Formalism	Electronic energy (E_h)	⟨S^2⟩
cod	0	1	RKS	−312.192953	0.000
PyF_5	0	1	RKS	−744.781872	0.000
$LNi^0(cod)$	0	1	UKS	−2630.963007	0.000
$LNi^{II}(PyF_4)F$	0	1	UKS	−3063.603169	0.000
$LNi^{II}(PyF_4)_2$	0	1	UKS	−3608.622881	0.000
$LNi^{II}F_2$	0	3	UKS	−2518.581121	2.005
$LNi^{I}F$	0	2	UKS	−2418.659413	0.784
$[LNi^{I}F]_2$	0	3	UKS	−4837.350427	2.121
PyF_4–PyF_4	0	1	RKS	−1289.832477	0.000
$LNi^{I}(PyF_4)$	0	2	UKS	−2963.669842	0.763

6.3 Spin-State Analysis

Multiple spin states were evaluated for the principal Ni(II) products and the dinuclear Ni(I) fluoride complex. The singlet states of $LNi^{II}(PyF_4)F$ and $LNi^{II}(PyF_4)_2$ were lower than their corresponding triplet states by 12.7 and 26.9 kcal mol^{-1}, respectively. Both singlet calculations converged to spin-pure solutions with ⟨S^2⟩ = 0.000. In contrast, triplet $LNi^{II}F_2$ was favored over its singlet state by 14.7 kcal mol^{-1}. The calculated singlet $LNi^{II}F_2$ solution had ⟨S^2⟩ = 0.972, indicating substantial open-shell character or spin contamination.

Triplet and broken-symmetry singlet states were optimized for $[LNi^{I}F]_2$. The two states were nearly isoenergetic, with the triplet lower by 0.3 kcal mol^{-1}. The triplet and broken-symmetry singlet solutions had ⟨S^2⟩ values of 2.121 and 1.082, respectively. The nonzero ⟨S^2⟩ value of the broken-symmetry solution is consistent with its intended mixed-spin character. The triplet structure was used in the balanced reaction-energy analysis because it was the lower-energy optimized state. However, the small calculated energy difference does not support an unambiguous assignment of the magnetic ground state.

Table S9. Alternative spin states

Species	State	Formalism	Electronic energy (E_h)	Relative energy (kcal mol^{-1})	⟨S^2⟩
$LNi^{II}(PyF_4)F$	Singlet	UKS	−3063.603169	0.0	0.000
$LNi^{II}(PyF_4)F$	Triplet	UKS	−3063.582946	+12.7	2.006
$LNi^{II}(PyF_4)_2$	Singlet	UKS	−3608.622881	0.0	0.000
$LNi^{II}(PyF_4)_2$	Triplet	UKS	−3608.580080	+26.9	2.006
$LNi^{II}F_2$	Triplet	UKS	−2518.581121	0.0	2.005
$LNi^{II}F_2$	Singlet	UKS	−2518.557775	+14.7	0.972
$[LNi^{I}F]_2$	Triplet	UKS	−4837.350427	0.0	2.121
$[LNi^{I}F]_2$	BSS	UKS-BS	−4837.349942	+0.3	1.082

6.4 Balanced Reaction-Energy Analysis

The calculations were designed to evaluate the thermodynamic plausibility of balanced transformations involving the experimentally relevant PyF_5/PyF_4 system. They were not intended to establish a complete reaction coordinate or a unique sequence of elementary steps. The lowest-energy calculated spin state was used for each species in the balanced reaction-energy analysis.

Table S10. Calculated electronic-energy changes for balanced transformations

No.	Balanced transformation	ΔE(SPE) (kcal mol^{-1})
1	$LNi^{0}(cod) + PyF_5 \rightarrow LNi^{II}(PyF_4)F + cod$	−32.2
2	$2\ LNi^{II}(PyF_4)F \rightarrow LNi^{II}(PyF_4)_2 + LNi^{II}F_2$	+1.5
3a	$LNi^{II}F_2 + LNi^{0}(cod) \rightarrow 2\ LNi^{I}F + cod$	+20.3
3b	$2\ LNi^{I}F \rightarrow [LNi^{I}F]_2$	−19.8
3 overall	$LNi^{II}F_2 + LNi^{0}(cod) \rightarrow [LNi^{I}F]_2 + cod$	+0.5
4	$2\ LNi^{II}(PyF_4)F \rightarrow PyF_4–PyF_4 + [LNi^{I}F]_2$	+14.7
5	$LNi^{II}(PyF_4)_2 + LNi^{0}(cod) \rightarrow 2\ LNi^{I}(PyF_4) + cod$	+33.4

Formation of $LNi^{II}(PyF_4)F$ from $LNi^{0}(cod)$ and PyF_5 is strongly favorable at the electronic-energy level, with a calculated ΔE(SPE) of −32.2 kcal mol^{-1}. This result supports the thermodynamic feasibility of initial C–F oxidative addition. Because no transition state was located, the calculation does not provide an activation barrier or identify the detailed geometry of the C–F cleavage step.

Redistribution of two $LNi^{II}(PyF_4)F$ intermediates to $LNi^{II}(PyF_4)_2$ and triplet $LNi^{II}F_2$ is approximately thermoneutral, with a calculated ΔE(SPE) of +1.5 kcal mol^{-1}. These results support the thermodynamic plausibility of initial C–F oxidative addition at two Ni(0) centers followed by ligand redistribution to the observed diaryl product and a nickel fluoride coproduct. The calculations do not establish whether redistribution occurs through a concerted bimetallic process or a sequence of ligand-transfer steps.

The corresponding balanced limiting stoichiometry is:

$$2\ LNi^{0}(cod) + 2\ PyF_5 \rightarrow LNi^{II}(PyF_4)_2 + LNi^{II}F_2 + 2\ cod$$

In this formulation, $LNi^{II}F_2$ represents a stoichiometrically balanced nickel fluoride coproduct and was not experimentally observed.

Comproportionation of $LNi^{II}F_2$ with $LNi^{0}(cod)$ to generate two monomeric $LNi^{I}F$ species is uphill by 20.3 kcal mol^{-1}. Subsequent dimerization of two $LNi^{I}F$ fragments to form $[LNi^{I}F]_2$ is favorable by 19.8 kcal mol^{-1}. The combined formation of $[LNi^{I}F]_2$ from $LNi^{II}F_2$ and $LNi^{0}(cod)$ is therefore nearly thermoneutral, with a net ΔE(SPE) of +0.5 kcal mol^{-1}. These results indicate that both Ni(II) fluoride and dinuclear Ni(I) fluoride formulations are energetically accessible. However, the calculations do not establish the identity of the experimentally observed nickel–fluoride-containing coproduct or demonstrate that comproportionation and dimerization occur under the reaction conditions.

The balanced transformation converting two $LNi(II)(PyF_4)F$ species to PyF_4–PyF_4 and $[LNi(I)F]_2$ is uphill by 14.7 kcal mol^{-1} in the present ground-state model. This result does not preclude

formation of the experimentally observed biaryl, which may proceed through alternative nickel nuclearities or oxidation states, stepwise radical pathways, and/or substrate-dependent processes promoted by excess PyF_5. Distinguishing among these possibilities will require further mechanistic study.

Finally, comproportionation of $LNi^{II}(PyF_4)_2$ with LNi^0(cod) to form two monomeric $LNi^I(PyF_4)$ species is uphill by 33.4 kcal mol^{-1}. Formation of monomeric Ni(I) aryl species is therefore not thermodynamically favored within the present ground-state model and is not required for formation of the Ni(II) diaryl product.

Overall, the calculations support the thermodynamic plausibility of initial C–F oxidative addition followed by ligand redistribution to the isolated Ni(II) diaryl complex and a nickel fluoride coproduct. They also demonstrate that multiple nickel fluoride formulations are energetically accessible. However, because the analysis is limited to electronic energies of selected stationary structures, it does not define a unique elementary mechanism or provide a definitive structural assignment of the nickel-containing coproduct.

6.5 Cartesian Coordinates of Optimized Structures

Optimized Cartesian coordinates for all structures included in the computational analysis are provided below, reported in ångströms (Å).

$^{t\text{-Bu}}$bpy | multiplicity 1 (UKS)				cod | multiplicity 1 (UKS)			
N	5.51957576	4.34743109	18.45369015	C	3.81507624	7.59677741	2.83447620
C	5.08111622	3.24431501	17.82722553	C	2.77335140	6.82784670	3.66751484
C	5.55790755	2.85419307	16.57188601	C	1.36680061	6.77510655	3.13869752
H	5.15668631	1.95297268	16.13251910	C	0.89747487	7.02090402	1.91166250
C	6.52389882	3.61474340	15.91940547	C	1.65568709	7.43221353	0.67500614
C	6.97063418	4.76308425	16.58367905	C	1.89089552	8.95158692	0.59020947
H	7.72073690	5.40891423	16.14377504	C	2.66744186	9.59490710	1.70596358
C	6.44584055	5.07989978	17.82789256	C	3.45971122	9.04788849	2.63286238
H	6.78554886	5.96867142	18.35188969	H	3.98098030	7.09192311	1.88414039
C	7.08886395	3.24417778	14.55259329	H	4.76444997	7.54737843	3.37520996
C	6.79829465	4.39031391	13.56420828	H	3.13423417	5.80190162	3.82303276
H	5.72164059	4.54602613	13.45820126	H	2.73157122	7.27250037	4.66898571
H	7.25113779	5.32667243	13.89755781	H	0.63058654	6.49297945	3.88981189
H	7.21087159	4.14161438	12.58280818	H	-0.17984865	6.93879352	1.77992412
C	6.47493963	1.95202255	14.00420604	H	2.60251345	6.90087066	0.59265488
H	6.91347541	1.73430818	13.02756812	H	0.91614120	9.45116387	0.53826074
H	6.67442000	1.10236149	14.66228713	H	2.54881172	10.67648034	1.74917515
H	5.39327155	2.04448472	13.87721466	H	3.89662549	9.72995624	3.35969567
N	3.58068794	1.35414260	17.88245372	H	1.06707979	7.14434719	-0.20058496
C	4.05111122	2.43248005	18.52865553	H	2.38391424	9.18065448	-0.36430624
C	3.60890116	2.79499932	19.80475523				
H	4.03561673	3.67628286	20.26025300				

$^{t\text{-Bu}}$bpy | multiplicity 1 (UKS)				cod | multiplicity 1 (UKS)			
C	2.64552831	2.03164855	20.45788463				
C	2.16350755	0.91081196	19.77171580				
H	1.41245032	0.26526597	20.21037790				
C	2.65518552	0.62039582	18.50775053				
C	2.11967527	2.37010443	21.84833325				
C	2.40792647	1.18753556	22.79345639				
H	2.02165086	1.41285028	23.79103439				
H	1.92952906	0.27135957	22.44047726				
H	3.48326178	1.00763575	22.87080241				
C	2.77262657	3.63142313	22.42262212				
H	2.57608597	4.50557393	21.79658105				
H	2.36131693	3.82668958	23.41574676				
H	3.85478525	3.51315480	22.52128539				
C	0.59714794	2.59314687	21.76421636				
H	0.36475539	3.42544982	21.09502125				
H	0.08576743	1.70134080	21.39569653				
H	0.20457869	2.82679460	22.75738550				
C	8.61311227	3.05541541	14.67715333				
H	2.28798288	-0.24674237	17.96644776				
H	9.09884026	3.96860928	15.02748178				
H	8.84652090	2.24962801	15.37776194				
H	9.03217794	2.79786022	13.70087274				

PyF5 | multiplicity 1 (UKS)				MeCN | multiplicity 1 (UKS)			
C	-1.52670778	-1.19214422	-0.28767379	C	-2.76376528	-3.11303862	0.37737660
C	-1.52670491	0.00830137	0.41391673	N	-2.76371288	-3.11303888	-0.77770776
C	-1.52671198	-0.06490688	1.79785274	C	-2.76371388	-3.11303831	1.83067503
N	-1.52671198	-1.19214491	2.46793877	H	-1.73594022	-3.11303951	2.19708262
C	-1.52671091	-2.31938255	1.79785292	H	-3.27766924	-4.00304914	2.19715271
C	-1.52670263	-2.39258882	0.41391968	H	-3.27767094	-2.22302694	2.19715376
F	-1.52669848	-3.44974007	2.50608851				
F	-1.52670049	-3.55705306	-0.23511842				
F	-1.52670904	-1.19215191	-1.61290554				
F	-1.52669935	1.17277057	-0.23511429				
F	-1.52669800	1.06544909	2.50608846				

$Ni(cod)_2$ | multiplicity 1 (UKS)				($^{t\text{-Bu}}$bpy)Ni(cod) | multiplicity 1 (UKS)			
Ni	4.05221077	6.17459975	7.66855427	Ni	1.07572547	3.16834018	8.67267653
C	3.94975612	4.95459850	9.37998497	N	-0.52721478	2.93827258	7.59302711

Ni(cod)$_2$ | multiplicity 1 (UKS)				($^{t\text{-Bu}}$bpy)Ni(cod) | multiplicity 1 (UKS)			
H	4.01885816	5.62569353	10.22939837	N	-0.05441778	4.44414978	9.60798493
C	5.12973753	4.69767304	8.69676076	C	-0.71670646	2.09222696	6.55485346
H	6.03378147	5.16587237	9.07723430	C	-1.31839916	4.56879467	9.10782240
C	5.35631876	3.55896563	7.73065159	C	-0.59462300	6.13394195	11.22788635
H	6.22109277	3.81444039	7.11062970	C	0.27472672	5.23480743	10.65495284
H	5.62541826	2.63735322	8.26775942	C	2.89470916	4.06131375	8.71747724
C	4.14659544	3.30395658	6.81259027	C	2.64504421	3.57995874	7.42540846
H	3.44252476	2.62145401	7.29164124	C	3.17976653	2.27424988	6.87761381
H	4.49067904	2.80020556	5.90495943	C	3.03473716	1.11291855	7.88492418
C	3.43908350	4.58870355	6.41936187	C	1.80828501	1.28046345	8.76854776
H	3.62490935	4.92281692	5.40430025	C	1.82807958	1.85077456	10.04805632
C	2.33088477	5.12774687	7.05481285	C	3.04566774	2.47725225	10.69265946
C	1.67945018	4.57866137	8.30176213	C	3.82211042	3.38623684	9.71597999
C	2.70247765	4.08943918	9.34348071	H	2.70677674	5.11856376	8.89360478
H	2.97607076	3.05092409	9.14977228	H	2.30750432	4.29473560	6.67709850
C	5.87993210	6.99092047	7.03928844	H	4.22852923	2.36907619	6.55752212
H	6.56315115	6.16192982	6.87492386	H	2.60859359	2.03216883	5.97608901
C	4.92208206	7.22149606	6.06230507	H	3.93072515	1.03319796	8.50466904
H	4.91723138	6.53846890	5.21948959	H	2.95935112	0.16945616	7.33619407
C	4.19717282	8.53463257	5.82700515	H	0.97297389	0.62247431	8.53764373
H	4.82422315	9.36855995	6.14696683	H	1.02681090	1.58147398	10.73363758
H	4.04306683	8.66350765	4.75195093	H	2.70043106	3.08137424	11.53738457
C	3.87170012	7.94479664	8.80677160	H	3.71668579	1.71473111	11.11656653
H	3.62693029	7.62753213	9.81476770	H	4.36085019	4.14891067	10.28613158
C	5.13544224	8.77809768	8.68553194	H	4.58243823	2.80993382	9.18424925
H	4.94102214	9.66709184	8.08291060	H	0.14595006	1.50506391	6.26570524
H	5.41430681	9.13888456	9.67955315	H	-0.25172795	6.72464465	12.06852825
C	6.31235074	7.97767513	8.09741753	H	1.28346952	5.11803672	11.03075833
H	6.79127412	7.41290745	8.90301056	C	-1.59593664	3.69236634	7.98342709
H	7.07828903	8.66295583	7.70503812	C	-1.91423182	1.96613416	5.88930292
C	2.84457788	7.89026093	7.87715613	C	-2.24112770	5.47196657	9.65052224
H	1.72236547	5.82323369	6.48310005	C	-1.90463702	6.27676055	10.72669538
H	1.08094817	5.37960676	8.74617143	C	-2.83839577	3.59955048	7.34378006
H	0.97588409	3.77082320	8.05189831	H	-1.97925749	1.26702628	5.06443592
H	2.23561767	4.10337549	10.33233914	C	-3.03050223	2.73394967	6.27940516
C	2.83217091	8.58570531	6.53687439	H	-3.22656936	5.53390624	9.21099664
H	1.87386092	7.55546271	8.23301095	H	-3.65315998	4.21421347	7.69951518
H	2.08946772	8.08533922	5.90804900	C	-2.86617390	7.27580386	11.35768276
H	2.49596040	9.62801432	6.63985789	C	-4.36009571	2.58779851	5.55000070
				C	-4.15907516	2.93762468	4.06259021
				H	-3.41972211	2.28237930	3.59663155

Ni(cod)$_2$ | multiplicity 1 (UKS)				($^{t\text{-Bu}}$bpy)Ni(cod) | multiplicity 1 (UKS)			
				H	-5.10449502	2.82182347	3.52566191
				H	-3.82213172	3.97149767	3.95110191
				C	-4.83894638	1.12752346	5.66585927
				H	-5.78352381	1.00445969	5.12905217
				H	-4.11001241	0.43659034	5.23679277
				H	-4.99711836	0.85537882	6.71261806
				C	-5.44051305	3.50697284	6.12981288
				H	-6.37007345	3.36435503	5.57361687
				H	-5.63764514	3.28055437	7.18101089
				H	-5.15695181	4.55977589	6.04950368
				C	-4.24332587	7.25617161	10.68621514
				H	-4.17804589	7.52595277	9.62883756
				H	-4.71389040	6.27232525	10.76363719
				H	-4.89386197	7.98199649	11.17990345
				C	-3.03805735	6.93488080	12.85082398
				H	-3.45818845	5.93321991	12.97324805
				H	-2.08254096	6.97418690	13.37812484
				H	-3.71555789	7.65461197	13.31822061
				C	-2.27256894	8.69247063	11.22883029
				H	-2.94417095	9.41849834	11.69523093
				H	-1.30066187	8.76021368	11.72252991
				H	-2.14541275	8.96350292	10.17757155

($^{t\text{-Bu}}$bpy)Ni | multiplicity 1 (UKS)				($^{t\text{-Bu}}$bpy)Ni | multiplicity 1 (RKS)			
Ni	-0.00904427	-0.01686505	-0.03236626	Ni	-0.09780148	-0.13158726	-0.08638546
N	0.02713608	-1.90776458	0.19178177	N	-0.07054516	-1.99944943	-0.32144041
C	1.03857965	-2.77674545	0.36681740	C	0.96965603	-2.86540102	-0.21072894
H	2.03235826	-2.34137492	0.38037615	H	1.93975095	-2.47011005	-0.48761019
C	0.86796856	-4.13581548	0.52132535	C	0.82971831	-4.18639991	0.16828734
H	1.74001531	-4.76010205	0.65285236	H	1.71556971	-4.80687261	0.19800096
C	-0.44655453	-4.67782029	0.50523718	C	-0.43929904	-4.71420459	0.45774517
C	-0.72492930	-6.16880350	0.69508962	C	-0.67725512	-6.16625028	0.85674744
C	-1.49413079	-6.37067739	2.01545199	C	-1.28247586	-6.21143161	2.27396443
H	-0.90630564	-6.00862780	2.86318401	H	-0.59653138	-5.76772187	3.00024598
H	-1.70399262	-7.43347627	2.16932870	H	-1.47177203	-7.24866458	2.56471797
H	-2.44422641	-5.83128813	2.00188001	H	-2.22846450	-5.66697047	2.31758405
C	0.56660383	-6.99382821	0.75023716	C	0.61879263	-6.98414067	0.85128937
H	1.15054114	-6.88099023	-0.16699642	H	1.07931917	-6.99696297	-0.14008189
H	0.31359969	-8.05097799	0.86393309	H	0.39435654	-8.01556572	1.13392518
H	1.19460684	-6.70519022	1.59698878	H	1.34626060	-6.58930270	1.56539823

($^{t\text{-}Bu}$bpy)Ni | multiplicity 1 (UKS)				($^{t\text{-}Bu}$bpy)Ni | multiplicity 1 (RKS)			
C	-1.58331304	-6.69157493	-0.47233086	C	-1.66754229	-6.80777589	-0.13477496
H	-1.78529231	-7.75802329	-0.33621763	H	-1.86207243	-7.84509398	0.15208047
H	-1.06289135	-6.55748078	-1.42436263	H	-1.25725398	-6.80003717	-1.14797821
H	-2.54066589	-6.16934688	-0.52794865	H	-2.61995009	-6.27287009	-0.14598999
C	-1.48841841	-3.79092790	0.32952857	C	-1.50606233	-3.81798500	0.38684846
H	-2.50727081	-4.15770133	0.31844827	H	-2.50567868	-4.13857317	0.65547526
C	-1.27464377	-2.40421264	0.16377196	C	-1.31626199	-2.48927936	0.02789761
C	-2.31308340	-1.44162359	-0.03712702	C	-2.37752146	-1.49035010	0.04883763
C	-3.68828052	-1.75206956	-0.11945526	C	-3.71366437	-1.78394025	-0.19438889
H	-3.98901284	-2.78822406	-0.02421573	H	-3.98823177	-2.81841599	-0.36418777
C	-4.64870039	-0.78299443	-0.32273087	C	-4.67889023	-0.77962391	-0.27502409
C	-6.12453932	-1.17009495	-0.41187666	C	-6.13775710	-1.12946655	-0.54646172
H	-6.77945776	0.55371633	-1.58547949	H	-6.73476877	0.80984658	-1.35663401
H	-5.96498470	-2.74834493	1.09372199	H	-6.05272172	-2.99915279	0.58877788
C	-6.32169699	-2.16001505	-1.57627317	C	-6.24519406	-1.83494742	-1.91266786
H	-7.37433868	-2.44975741	-1.64638569	H	-7.28702173	-2.10128687	-2.11266508
H	-5.72700768	-3.06432235	-1.42907949	H	-5.64920262	-2.75025860	-1.93313889
H	-6.02465930	-1.70415312	-2.52452155	H	-5.89570464	-1.17882000	-2.71403928
C	-7.02652236	0.04669985	-0.64900533	C	-7.03176654	0.11537110	-0.56639772
H	-6.94648392	0.77064839	0.16622834	H	-7.00017868	0.64490307	0.38941408
H	-8.06734388	-0.28181835	-0.70752115	H	-8.06532672	-0.18625736	-0.75297002
C	-6.55435745	-1.84853001	0.90284132	C	-6.64522693	-2.08210418	0.55336537
H	-6.42499383	-1.16783333	1.74853046	H	-6.59321006	-1.60177176	1.53397530
H	-7.60881200	-2.13507826	0.84815886	H	-7.68572319	-2.35571816	0.35685710
C	-4.19780228	0.55944882	-0.44496606	C	-4.21270911	0.53636608	-0.12000230
H	-4.88353817	1.37925406	-0.60407184	H	-4.88808137	1.38018161	-0.16806993
C	-2.84777806	0.82616490	-0.36035255	C	-2.87973443	0.78144533	0.14664174
H	-2.48025507	1.84293024	-0.45227193	H	-2.52821775	1.79145802	0.32053362
N	-1.90672660	-0.11471478	-0.16475831	N	-1.94424930	-0.19513438	0.26842582

($^{t\text{-}Bu}$bpy)Ni($MeCN)_2$ | multiplicity 1 (UKS)				($^{t\text{-}Bu}$bpy)Ni($MeCN)_2$ | multiplicity 1 (RKS)			
Ni	0.13648688	0.15619480	0.00573114	Ni	0.12760108	0.14742307	-0.00099973
N	0.02834120	-1.78987033	0.42268143	N	-0.03760527	-1.72294514	0.61798387
C	1.03345700	-2.63349813	0.72064187	C	0.96492679	-2.55011240	1.00121407
H	2.00971313	-2.17217176	0.83072196	H	1.90345889	-2.06480927	1.24421867
C	0.88349717	-3.99263459	0.89579480	C	0.83751227	-3.91793767	1.10713698
H	1.74877226	-4.58597070	1.15661750	H	1.69019905	-4.48940470	1.44911785
C	-0.40222471	-4.57792911	0.73563535	C	-0.38507898	-4.54574665	0.79077495
C	-0.65663648	-6.07658205	0.89448311	C	-0.59252199	-6.05636831	0.85215145
C	-1.71297425	-6.30245106	1.99306313	C	-1.80445338	-6.37446782	1.74813517
H	-1.36142667	-5.91294609	2.95226456	H	-1.63869620	-6.01073323	2.76560000

($^{t\text{-Bu}}$bpy)Ni(MeCN)$_2$ | multiplicity 1 (UKS)				($^{t\text{-Bu}}$bpy)Ni(MeCN)$_2$ | multiplicity 1 (RKS)			
H	-1.91267174	-7.37222725	2.10684863	H	-1.96269544	-7.45590483	1.78906534
H	-2.65293768	-5.80417392	1.74516494	H	-2.71602292	-5.91252753	1.36264423
C	0.61555442	-6.84058529	1.27962598	C	0.63437124	-6.78199767	1.41629608
H	1.38874586	-6.74571942	0.51266562	H	1.51680835	-6.62625179	0.79030060
H	0.37902644	-7.90188969	1.39116957	H	0.43280974	-7.85544005	1.45299165
H	1.02562382	-6.48384677	2.22814593	H	0.86643013	-6.44545809	2.43023935
C	-1.18645370	-6.64630996	-0.43578507	C	-0.86592686	-6.58386130	-0.56999477
H	-1.37880479	-7.71874167	-0.33501407	H	-1.02307429	-7.66612856	-0.54332863
H	-0.45617553	-6.49976185	-1.23613286	H	-0.01916069	-6.37310506	-1.22841331
H	-2.11824261	-6.15700154	-0.72790205	H	-1.75746575	-6.11845608	-0.99719629
C	-1.43559233	-3.72510012	0.40598981	C	-1.41081420	-3.70745493	0.37680373
H	-2.43051238	-4.12372743	0.24616914	H	-2.36576873	-4.12374914	0.07834071
C	-1.23475581	-2.33667597	0.25062985	C	-1.23459403	-2.32300087	0.30092725
C	-2.25488814	-1.39877850	-0.09338952	C	-2.24779001	-1.39653467	-0.14831214
C	-3.63058655	-1.70081033	-0.18248027	C	-3.61797724	-1.67166417	-0.15528465
H	-3.95174775	-2.71405920	0.02876803	H	-3.95077405	-2.64180271	0.19467862
C	-4.56719181	-0.74305585	-0.51258504	C	-4.54447707	-0.72242894	-0.56401429
C	-6.04918690	-1.10599621	-0.59920422	C	-6.03717344	-1.03874074	-0.55199355
H	-6.63019412	0.50017860	-1.96144705	H	-6.61935158	0.39280658	-2.09673889
H	-5.96236839	-2.51907020	1.06719214	H	-5.92048025	-2.21507791	1.28729355
C	-6.24580185	-2.20714547	-1.65851762	C	-6.30651297	-2.26524126	-1.44466797
H	-7.30312247	-2.48163094	-1.72179764	H	-7.37326775	-2.50650369	-1.43514187
H	-5.67461224	-3.10366931	-1.40702480	H	-5.75608258	-3.13989027	-1.09080967
H	-5.92031714	-1.85850651	-2.64235595	H	-6.00649778	-2.06471176	-2.47654236
C	-6.91513519	0.09919873	-0.98523974	C	-6.87806736	0.13516697	-1.06626449
H	-6.83706946	0.90149869	-0.24690165	H	-6.74701683	1.02486056	-0.44481976
H	-7.96211365	-0.21019350	-1.03887372	H	-7.93511937	-0.14118854	-1.04210551
C	-6.52690098	-1.63317146	0.76754292	C	-6.47353770	-1.36073278	0.89071929
H	-6.40297080	-0.86969823	1.54029602	H	-6.29816223	-0.50385959	1.54647565
H	-7.58591760	-1.90339909	0.71551284	H	-7.54021068	-1.60207231	0.91355025
C	-4.08165576	0.57283053	-0.74451445	C	-4.01938490	0.52715583	-0.95328582
H	-4.74445607	1.38433239	-1.01071506	H	-4.66445141	1.32470740	-1.29738350
C	-2.73090710	0.82340304	-0.63181451	C	-2.66065570	0.75253140	-0.91703924
H	-2.34433353	1.82431090	-0.79544104	H	-2.25395888	1.71177103	-1.21662253
N	-1.80660739	-0.10810246	-0.33294343	N	-1.74825987	-0.17419435	-0.53571537
C	3.06819977	0.11605247	-0.93024922	C	2.93388552	0.01389863	-1.11777022
N	1.96813430	0.16991123	-0.57604497	N	1.83524800	0.10135372	-0.74906239
C	4.44704142	0.04327575	-1.37393820	C	4.30946253	-0.09506971	-1.56918357
H	4.49107516	0.11168023	-2.46282931	H	4.35861627	-0.02432695	-2.65829145
H	4.88432099	-0.90516307	-1.05583288	H	4.72582884	-1.05567489	-1.25781573
H	5.01789386	0.86587374	-0.93895186	H	4.90934101	0.70765282	-1.13457400
C	-0.07538179	3.10324830	0.86596927	C	-0.15281862	2.97010350	1.04406036

($^{t\text{-Bu}}$bpy)Ni(MeCN)$_2$ | multiplicity 1 (UKS)				($^{t\text{-Bu}}$bpy)Ni(MeCN)$_2$ | multiplicity 1 (RKS)			
N	0.04298678	2.00143838	0.53344590	N	-0.00987100	1.87032682	0.69669766
C	-0.22818307	4.48267332	1.28709417	C	-0.33437139	4.34405081	1.47675802
H	0.74413483	4.97873193	1.28068680	H	0.62698673	4.86219575	1.47801655
H	-0.64307948	4.51706662	2.29651089	H	-0.75152802	4.36927855	2.48610089
H	-0.90236061	5.00397889	0.60457452	H	-1.01530225	4.86390634	0.79879052

($^{t\text{-Bu}}$bpy)Ni(MeCN)$_2$ | multiplicity 3 (UKS)				($^{t\text{-Bu}}$bpy)Ni(PyF$_4$)F | multiplicity 1 (UKS)			
Ni	0.12981166	0.14468519	0.01819593	Ni	-0.00690979	-0.09289272	-0.18012386
N	0.03526263	-1.84632014	0.25122500	N	-0.06161186	-1.95852835	0.08644774
C	1.04162903	-2.70234839	0.49063174	C	0.98199145	-2.78712732	0.23660934
H	2.03272787	-2.26047425	0.51056732	H	1.96325397	-2.33844875	0.19683793
C	0.88335870	-4.05769490	0.69721324	C	0.83551150	-4.15083251	0.43251823
H	1.75815648	-4.66726907	0.87477728	H	1.73043225	-4.74711315	0.53942494
C	-0.42558932	-4.61516494	0.66928937	C	-0.43435806	-4.72308458	0.48962334
C	-0.69509889	-6.09765963	0.92794620	C	-0.67505959	-6.20856934	0.71359481
C	-1.43204615	-6.24316956	2.27419085	C	-1.44206270	-6.38454594	2.03986049
H	-0.82606453	-5.84173393	3.09093578	H	-0.85724530	-5.99930243	2.87862980
H	-1.63495793	-7.29848659	2.48114442	H	-1.63470211	-7.44686250	2.20918078
H	-2.38367068	-5.70639140	2.25785291	H	-2.40099515	-5.86191241	2.01724931
C	0.60016421	-6.91698744	0.98828512	C	0.63534502	-6.99923129	0.78447974
H	1.16736015	-6.83272714	0.05748531	H	1.20954341	-6.90201758	-0.14042150
H	0.35340185	-7.97066395	1.14219615	H	0.40505425	-8.05661827	0.93111401
H	1.24252523	-6.59886640	1.81328916	H	1.26041782	-6.67138265	1.61900999
C	-1.57853516	-6.67738346	-0.19238396	C	-1.52671982	-6.76088127	-0.44667532
H	-1.77302342	-7.73672753	-0.00073987	H	-1.70540147	-7.82693306	-0.28693702
H	-1.08120913	-6.58785732	-1.16191209	H	-1.00957140	-6.63455937	-1.40092192
H	-2.53976688	-6.16282246	-0.25149262	H	-2.49474498	-6.25949466	-0.50769770
C	-1.46718073	-3.74451596	0.42882111	C	-1.51575245	-3.85161214	0.33354415
H	-2.48465239	-4.11573982	0.41122003	H	-2.52909968	-4.22801473	0.36790229
C	-1.25245498	-2.36335832	0.21222121	C	-1.31278562	-2.49833128	0.12835852
C	-2.28019998	-1.41290284	-0.05336453	C	-2.38188041	-1.52029691	-0.07664537
C	-3.64877135	-1.72855361	-0.21690475	C	-3.73697716	-1.80181135	-0.10675237
H	-3.95373389	-2.76525577	-0.13552187	H	-4.07184477	-2.81925509	0.04178931
C	-4.59311455	-0.76073502	-0.48601944	C	-4.66464980	-0.77991173	-0.33524719
C	-6.06264386	-1.14268149	-0.66352663	C	-6.15207661	-1.10146467	-0.37508446
H	-6.63612241	0.57776447	-1.88162600	H	-6.74147087	0.56434104	-1.65713357
H	-6.00315108	-2.71481415	0.85620622	H	-6.01561165	-2.57293400	1.24272824
C	-6.19612614	-2.13534019	-1.83427726	C	-6.40491931	-2.16330859	-1.46389713
H	-7.24429150	-2.42026485	-1.96637507	H	-7.47025551	-2.40503561	-1.49213409
H	-5.61536789	-3.04197351	-1.65056212	H	-5.85241839	-3.08367692	-1.26369212
H	-5.84162088	-1.68364191	-2.76471513	H	-6.10815394	-1.78796096	-2.44631700

($^{t\text{-Bu}}$bpy)Ni(MeCN)$_2$ | multiplicity 3 (UKS)			
C	-6.94271170	0.07770935	-0.95923792
H	-6.90520169	0.80517867	-0.14417597
H	-7.98048912	-0.24450353	-1.07766811
C	-6.57520918	-1.81306495	0.62529972
H	-6.49270650	-1.12882544	1.47403924
H	-7.62627332	-2.09459201	0.50970691
C	-4.12839019	0.57937781	-0.59034642
H	-4.79982953	1.40116959	-0.79598981
C	-2.78274283	0.83827176	-0.42336069
H	-2.41021874	1.85496135	-0.49763433
N	-1.85476620	-0.09603039	-0.16145031
C	3.15209018	0.19961224	-0.70027309
N	2.03380938	0.19482919	-0.40820870
C	4.55455415	0.19459911	-1.06879840
H	4.65154867	0.29179231	-2.15190566
H	5.01294241	-0.74303953	-0.74885876
H	5.06533995	1.02931265	-0.58527258
C	-0.02394604	3.17181205	0.69690644
N	0.05239656	2.05483018	0.40965323
C	-0.12908935	4.57121693	1.06234786
H	0.86293594	5.02619803	1.06648583
H	-0.57061671	4.65879570	2.05697309
H	-0.76192344	5.09207868	0.34120706

($^{t\text{-Bu}}$bpy)Ni(PyF$_4$)F | multiplicity 1 (UKS)			
C	-7.00049001	0.13588080	-0.68562334
H	-6.88215732	0.90777555	0.07900704
H	-8.05338395	-0.15296356	-0.71308537
C	-6.57230243	-1.66585676	0.99662279
H	-6.40219250	-0.93006065	1.78637153
H	-7.63655294	-1.91323569	0.97543646
C	-4.15398842	0.50630243	-0.51749033
H	-4.80460646	1.35017254	-0.69781369
C	-2.78477456	0.72633106	-0.47225817
H	-2.34637460	1.70623147	-0.60807185
N	-1.91538933	-0.26361070	-0.25959438
F	-0.10080902	1.69435753	-0.44880917
C	1.88438518	0.06958994	-0.07409198
C	2.55374652	0.26133903	1.12497817
F	1.87359215	0.27537444	2.29850909
C	3.92812103	0.43269096	1.15706348
F	4.55765306	0.61462836	2.33214927
N	4.68332689	0.42899420	0.08194730
C	4.07508953	0.25220947	-1.06922945
F	4.85510297	0.24849253	-2.16538264
C	2.70638615	0.07472120	-1.19057294
F	2.18610806	-0.10405555	-2.43012041

($^{t\text{-Bu}}$bpy)Ni(PyF$_4$)F | multiplicity 3 (UKS)			
Ni	0.10200585	-0.05031278	0.29986707
N	-0.04908315	-2.04088079	0.52623559
C	0.94823431	-2.88095387	0.80939321
H	1.92780921	-2.43837941	0.93290816
C	0.75742728	-4.24818652	0.94212748
H	1.61091946	-4.86856395	1.17562048
C	-0.51604224	-4.79120015	0.77016918
C	-0.81284009	-6.27942245	0.89004790
C	-1.86586878	-6.49009454	1.99625966
H	-1.48983701	-6.13867785	2.96015600
H	-2.09348386	-7.55561891	2.07977608
H	-2.79387641	-5.95977914	1.77250278
C	0.43979730	-7.08985458	1.23811455
H	1.20720979	-6.99599105	0.46556583
H	0.16932935	-8.14482327	1.31898029
H	0.86648299	-6.77543816	2.19391960

($^{t\text{-Bu}}$bpy)Ni(PyF$_4$)$_2$ | multiplicity 1 (UKS)			
Ni	-0.01058491	-0.00143553	0.01776093
N	-0.05437779	-1.92242197	0.18599568
C	0.98143690	-2.75543239	0.34575748
H	1.96490111	-2.30787485	0.34649944
C	0.82904819	-4.12390746	0.50390262
H	1.71839798	-4.72674349	0.62047216
C	-0.44406567	-4.69168618	0.51214814
C	-0.69636888	-6.18192157	0.68797266
C	-1.50168522	-6.39282083	1.98586978
H	-0.94110172	-6.03162665	2.85146957
H	-1.69971614	-7.45909722	2.12050736
H	-2.45936738	-5.86861945	1.95052576
C	0.60850098	-6.98010081	0.77431886
H	1.21076914	-6.85889396	-0.12962878
H	0.37004975	-8.04024098	0.88315082
H	1.20948192	-6.67963209	1.63640792

(${}^{t\text{-Bu}}$bpy)Ni(PyF$_4$)F | multiplicity 3 (UKS)				(${}^{t\text{-Bu}}$bpy)Ni(PyF$_4$)$_2$ | multiplicity 1 (UKS)			
C	-1.37666507	-6.78032094	-0.45462540	C	-1.51606719	-6.69621729	-0.51210563
H	-1.60315361	-7.84658952	-0.37744852	H	-1.70459871	-7.76544063	-0.38891356
H	-0.64881798	-6.63681001	-1.25690494	H	-0.97029399	-6.54527494	-1.44662665
H	-2.29569905	-6.25461741	-0.72254550	H	-2.47915209	-6.18721273	-0.58649624
C	-1.54828086	-3.89659156	0.46996865	C	-1.51919480	-3.81222743	0.35537652
H	-2.55444474	-4.26349842	0.32269368	H	-2.53290204	-4.18826505	0.36143372
C	-1.29527420	-2.53828920	0.35761842	C	-1.30408727	-2.45419441	0.19083702
C	-2.33045293	-1.53138694	0.05221083	C	-2.37041283	-1.46729146	0.00155339
C	-3.67411592	-1.83015930	-0.11313840	C	-3.71511195	-1.78249825	-0.09232492
H	-4.00802735	-2.85396513	-0.01722044	H	-4.02155962	-2.81618536	-0.01052951
C	-4.59771012	-0.81861474	-0.39541836	C	-4.67037797	-0.78378897	-0.30144120
C	-6.07124154	-1.16155698	-0.56562733	C	-6.14333269	-1.14926422	-0.40856365
H	-6.59350131	0.54661666	-1.82082617	H	-6.76067520	0.58445285	-1.58532127
H	-6.03545469	-2.69971400	0.99527274	H	-6.00232585	-2.73352270	1.09827643
C	-6.21927418	-2.17960453	-1.71351438	C	-6.32584051	-2.13631799	-1.57905985
H	-7.27421308	-2.43844308	-1.83277915	H	-7.38020896	-2.41215621	-1.65813526
H	-5.66385547	-3.09739036	-1.50950756	H	-5.74498810	-3.04834594	-1.42633780
H	-5.85713912	-1.75713154	-2.65395121	H	-6.01396193	-1.68029792	-2.52177310
C	-6.91538418	0.07563330	-0.88859205	C	-7.02404098	0.08012972	-0.65205653
H	-6.86386745	0.81696924	-0.08717845	H	-6.94461448	0.79956098	0.16697558
H	-7.95879050	-0.22549050	-1.00374116	H	-8.06663625	-0.23727337	-0.72246952
C	-6.58497491	-1.78938976	0.74544455	C	-6.58097292	-1.82756621	0.90469910
H	-6.48454773	-1.08601604	1.57563530	H	-6.45494554	-1.14862896	1.75153849
H	-7.64112288	-2.04712541	0.63435737	H	-7.63586794	-2.10417558	0.83521722
C	-4.09845798	0.48023535	-0.49701114	C	-4.19202795	0.52132106	-0.40595257
H	-4.74458664	1.31918030	-0.71260513	H	-4.85742969	1.35656382	-0.57196496
C	-2.74317084	0.71373901	-0.31639453	C	-2.83347055	0.77650796	-0.29940986
H	-2.33309702	1.71422311	-0.38322196	H	-2.45404723	1.78508758	-0.38129759
N	-1.87432559	-0.26387180	-0.04996631	N	-1.92677757	-0.18736067	-0.09609918
F	-0.11487889	0.76529109	1.97979321	C	-0.00822299	1.89172190	-0.00380651
C	2.02128326	0.32134372	-0.15451722	C	0.30757287	2.68552345	-1.09872076
C	3.10200737	0.18982513	0.70200170	F	0.64745060	2.12394627	-2.28255581
F	2.92819067	-0.23890002	1.97918569	C	0.27745358	4.06764674	-1.01324602
C	4.39466372	0.47468249	0.28991017	F	0.58477235	4.81257591	-2.09039312
F	5.42442817	0.33423210	1.14597131	N	-0.04060622	4.72239312	0.08032370
N	4.69382853	0.88551530	-0.92170955	C	-0.34796740	3.99824091	1.13272815
C	3.69322934	1.02096554	-1.76233616	F	-0.67886400	4.67245443	2.24871818
F	4.00419921	1.44018424	-3.00339314	C	-0.34755981	2.61373986	1.13502331
C	2.37724558	0.75370137	-1.42158972	F	-0.70861340	1.97257362	2.27280366
F	1.42202723	0.92701419	-2.37677985	C	1.87761169	0.13256983	0.01053378
				C	2.65722874	0.50527017	1.09767462

($^{t\text{-Bu}}$bpy)Ni(PyF$_4$)F | multiplicity 3 (UKS)				($^{t\text{-Bu}}$bpy)Ni(PyF$_4$)$_2$ | multiplicity 1 (UKS)			
				F	2.08249852	0.80716777	2.28561485
				C	4.03719323	0.57095605	0.99972535
				F	4.76932593	0.92872543	2.06996473
				N	4.70208062	0.29763489	-0.09992703
				C	3.99145888	-0.06041121	-1.14544082
				F	4.67656678	-0.34566603	-2.26735012
				C	2.61025088	-0.15697201	-1.13492658
				F	1.98551555	-0.56519903	-2.26574448

($^{t\text{-Bu}}$bpy)Ni(PyF$_4$)$_2$ | multiplicity 3 (UKS)				($^{t\text{-Bu}}$bpy)Ni(PyF$_4$) | multiplicity 2 (UKS)			
Ni	0.07109459	0.08532696	0.02339187	Ni	-0.26010249	0.13509942	0.27069279
N	-0.08287039	-1.95312274	0.34063908	N	-0.25308354	-1.79390179	0.44773164
C	0.93426987	-2.77297738	0.61547478	C	0.80963819	-2.57430375	0.68882100
H	1.91211647	-2.31327518	0.68411550	H	1.75672103	-2.06393407	0.81412384
C	0.76994719	-4.13708523	0.80206341	C	0.72686917	-3.95562362	0.77804581
H	1.63954034	-4.73739259	1.02895553	H	1.63041997	-4.51438849	0.97732869
C	-0.49988978	-4.70393606	0.68964911	C	-0.50365002	-4.59229559	0.61256279
C	-0.76988789	-6.19185471	0.86485733	C	-0.68390083	-6.10255318	0.69285079
C	-1.79030990	-6.38240012	2.00473865	C	-1.70118051	-6.42594264	1.80498180
H	-1.39592076	-5.99202710	2.94604719	H	-1.33986194	-6.07338446	2.77418270
H	-1.99704903	-7.44808150	2.13023901	H	-1.84699167	-7.50762735	1.86098612
H	-2.73283612	-5.87562763	1.78723183	H	-2.66956499	-5.96136761	1.60722711
C	0.50381093	-6.97228286	1.20486600	C	0.63198100	-6.82418939	1.00118766
H	1.24869859	-6.89278509	0.40899458	H	1.37933254	-6.64810959	0.22327552
H	0.25177190	-8.02781665	1.32731680	H	0.44618102	-7.89926759	1.05300056
H	0.95135999	-6.62031482	2.13779028	H	1.04719384	-6.50604712	1.96093316
C	-1.36055784	-6.74337632	-0.44826422	C	-1.22817704	-6.60903705	-0.65796170
H	-1.56799585	-7.81006041	-0.33255836	H	-1.37605266	-7.69078824	-0.60833020
H	-0.65596735	-6.61381555	-1.27336776	H	-0.52425418	-6.39108408	-1.46490986
H	-2.29423324	-6.23982858	-0.70801271	H	-2.18637550	-6.14408428	-0.89982217
C	-1.55403967	-3.83358136	0.39550373	C	-1.60557285	-3.76934839	0.36050265
H	-2.55848929	-4.21954300	0.29203727	H	-2.58493559	-4.20652563	0.22085458
C	-1.32413791	-2.47541322	0.23402181	C	-1.46057537	-2.39298759	0.28561202
C	-2.38721758	-1.49585434	-0.06136729	C	-2.57184692	-1.45808108	0.03621842
C	-3.72914181	-1.82774339	-0.17210537	C	-3.89317956	-1.84521352	-0.12662429
H	-4.03800695	-2.85478503	-0.03543927	H	-4.15110895	-2.89473011	-0.08255360
C	-4.68393023	-0.84663402	-0.45584841	C	-4.89244701	-0.89141204	-0.34314118
C	-6.15376867	-1.22614765	-0.56796830	C	-6.33975036	-1.33337383	-0.51588021
H	-6.75574946	0.43619414	-1.84906921	H	-7.02471441	0.41807583	-1.62874754
H	-6.03475582	-2.72344470	1.02802269	H	-6.15948104	-2.98341364	0.91226230
C	-6.31516551	-2.27641431	-1.68460899	C	-6.43117632	-2.26916340	-1.73746317

($^{t\text{-Bu}}$bpy)Ni(PyF_4)$_2$ | multiplicity 3 (UKS)

H	-7.36720455	-2.56151526	-1.76274326
H	-5.73259944	-3.17627498	-1.47633220
H	-5.99321532	-1.87029364	-2.64662448
C	-7.03532954	-0.01608393	-0.89421530
H	-6.97258862	0.74748414	-0.11479742
H	-8.07536116	-0.34162132	-0.96538324
C	-6.61131039	-1.83112632	0.77407619
H	-6.50013666	-1.10519191	1.58322910
H	-7.66460241	-2.11360821	0.70337436
C	-4.21675842	0.45779678	-0.61651726
H	-4.88699453	1.27581076	-0.83921610
C	-2.86255193	0.72495909	-0.48400568
H	-2.47774376	1.73120883	-0.59180786
N	-1.96174249	-0.22289240	-0.21463137
C	-0.03643920	2.09053241	0.29826639
C	0.18302413	3.02452459	-0.69794641
F	0.39824731	2.62115417	-1.97959782
C	0.18613486	4.38388491	-0.43084033
F	0.39615350	5.27181865	-1.42174982
N	-0.01121297	4.88339967	0.76898159
C	-0.22264287	4.02247074	1.73948472
F	-0.42611930	4.54082009	2.96613528
C	-0.24422522	2.65129817	1.54530993
F	-0.48341053	1.85220447	2.62278897
C	2.07041143	0.10639504	-0.31291017
C	3.01482512	0.41583431	0.64895109
F	2.63380658	0.64209538	1.93565071
C	4.36325186	0.49985284	0.34233242
F	5.26167617	0.79664346	1.30108672
N	4.84179778	0.30003614	-0.86549260
C	3.97045537	0.00400024	-1.80407268
F	4.46753399	-0.20168717	-3.03913573
C	2.60950098	-0.10156921	-1.56944685
F	1.79922331	-0.42370575	-2.61638934

($^{t\text{-Bu}}$bpy)Ni(PyF_4) | multiplicity 2 (UKS)

H	-7.46468200	-2.60069618	-1.86558952
H	-5.80227406	-3.15264247	-1.60851757
H	-6.11724511	-1.75056889	-2.64667876
C	-7.28267484	-0.14463509	-0.72780247
H	-7.26404966	0.53864668	0.12521120
H	-8.30408795	-0.51446068	-0.84189725
C	-6.78013878	-2.10020074	0.74687710
H	-6.71436504	-1.46050781	1.63040325
H	-7.81641888	-2.42773703	0.63146176
C	-4.48750549	0.44287665	-0.38630719
H	-5.19620340	1.24309744	-0.54752015
C	-3.14763331	0.76414229	-0.21976630
H	-2.81182360	1.79320421	-0.25026027
N	-2.20092605	-0.15608658	-0.01309121
C	0.19334633	2.01391547	0.13022767
C	-0.08576094	2.80657741	-0.97784986
F	-0.69579693	2.26880283	-2.07214150
C	0.23438821	4.15218755	-1.02287691
F	-0.06013891	4.88556708	-2.11670227
N	0.82870463	4.79084007	-0.03820092
C	1.11636062	4.07529512	1.02741304
F	1.72793680	4.72916408	2.03705545
C	0.82354881	2.72772559	1.14350282
F	1.17622222	2.10400251	2.30449501

($^{t\text{-Bu}}$bpy)NiF | multiplicity 2 (UKS)

Ni	10.07300503	10.45144212	9.40968681
F	9.53201754	10.11944403	11.13481941
N	11.21844056	10.28063523	7.87355601
N	9.06529983	11.63446845	8.20107527
C	12.34120742	9.54209167	7.78267118

($^{t\text{-Bu}}$bpy)NiF_2 | multiplicity 1 (UKS)

Ni	0.00029763	0.00010129	-0.02485446
F	-1.71094834	-0.50377763	-0.53293915
N	-0.37027565	1.26356288	1.48804755
C	-0.21120423	0.70809137	2.70894154
C	-0.43724317	1.44150971	3.86488073

($^{t\text{-}Bu}$bpy)NiF | multiplicity 2 (UKS)				($^{t\text{-}Bu}$bpy)NiF_2 | multiplicity 1 (UKS)			
H	12.61909090	8.99023560	8.67210924	H	-0.30446830	0.96933422	4.82836424
C	13.10711967	9.47801518	6.63536031	C	-0.83459225	2.77986789	3.79404420
H	13.99697050	8.86152277	6.64029610	C	-0.98705163	3.32519068	2.51917034
C	12.73245226	10.20051667	5.49395907	H	-1.29046455	4.35242584	2.37473827
C	11.57088726	10.96094701	5.59504651	C	-0.74761865	2.53999021	1.40081322
H	11.22517912	11.54274456	4.75318918	H	-0.86026989	2.94001172	0.40026445
C	10.83849130	10.98561103	6.77857271	C	-1.08287666	3.56693009	5.07429774
C	9.60356605	11.75846871	6.96674328	C	0.20664065	3.56703397	5.91907540
C	9.01551645	12.55046812	5.98359232	H	0.50656987	2.55279279	6.19126603
H	9.48233253	12.61748430	5.01174049	H	1.02761994	4.03619414	5.37140734
C	7.83839788	13.24571518	6.24781707	C	-2.21278951	2.87853070	5.86534306
C	7.29928531	13.10334005	7.53384912	H	-3.13741287	2.86215163	5.28310739
H	6.38661116	13.61210841	7.81684045	H	-1.94932910	1.85089972	6.12507405
C	7.93141482	12.30309957	8.46640545	C	-1.48845048	5.01668369	4.78917837
H	7.53012407	12.18019162	9.46521516	H	-0.70659567	5.55179997	4.24397987
C	13.57432785	10.13744477	4.22623496	H	-1.65342511	5.53311747	5.73733475
C	12.98946490	10.99894257	3.10197622	H	-2.41428895	5.06815469	4.21056770
H	12.94071104	12.05312001	3.38750818	F	1.71158497	0.50467108	-0.53214786
H	13.62832731	10.91722784	2.21980049	N	0.37045143	-1.26354832	1.48800476
H	11.98548525	10.66781827	2.82345391	C	0.21122539	-0.70813262	2.70892083
C	14.99658062	10.63708130	4.54844467	C	0.43713660	-1.44158997	3.86485470
H	14.97236831	11.67235426	4.89786902	H	0.30439734	-0.96942282	4.82834760
C	13.64569198	8.67460063	3.74627102	C	0.83441991	-2.77996072	3.79403347
H	14.09032269	8.02864629	4.50628068	C	0.98692844	-3.32526809	2.51915655
H	12.64800621	8.29557980	3.51075002	H	1.29029395	-4.35251437	2.37473125
C	7.14483580	14.12309154	5.21387688	C	0.74771946	-2.54001055	1.40079805
C	5.71983452	13.58408256	4.98032862	H	0.86058411	-2.93999926	0.40025872
H	5.13860374	13.58287303	5.90482996	C	1.08275326	-3.56699469	5.07429770
H	5.20477735	14.21730333	4.25336845	C	-0.20669118	-3.56711424	5.91916319
H	5.75197176	12.56367922	4.59048711	H	-0.50663322	-2.55288933	6.19140378
C	7.06165010	15.56301939	5.75748168	H	-1.02769997	-4.03630421	5.37156312
H	8.06119952	15.96635780	5.93776665	C	2.21267739	-2.87851234	5.86524544
H	6.49899537	15.60298151	6.69250930	H	3.13725325	-2.86207148	5.28293781
C	7.89141175	14.14105819	3.87599743	H	1.94915864	-1.85089730	6.12498800
H	7.95522213	13.14106945	3.43890420	C	1.48838852	-5.01674772	4.78921691
H	7.35159240	14.78151327	3.17497542	H	0.70654693	-5.55190705	4.24404121
H	8.90404326	14.53802515	3.98512882	H	1.65339456	-5.53315746	5.73737367
H	15.61392250	10.58921410	3.64762050	H	2.41423283	-5.06817627	4.21061062
H	15.46805996	10.02287771	5.31857610	H	0.03538134	4.13061558	6.83941865
H	14.26109665	8.61544104	2.84482200	H	-2.39531250	3.42903615	6.79164627
H	6.55689514	16.20032672	5.02692532	H	2.39531461	-3.42899901	6.79153414

($^{t\text{-Bu}}$bpy)NiF | multiplicity 2 (UKS)				($^{t\text{-Bu}}$bpy)NiF$_2$ | multiplicity 1 (UKS)			
				H	-0.03532913	-4.13070203	6.83948541

($^{t\text{-Bu}}$bpy)NiF$_2$ | multiplicity 3 (UKS)				[($^{t\text{-Bu}}$bpy)NiF]$_2$ | multiplicity 1 (UKS)			
Ni	0.00083904	0.00025900	-0.00217804	Ni	9.97028628	10.31936516	9.53633059
F	-1.68632848	-0.47392867	-0.66397769	Ni	10.68073304	10.77084894	11.94870884
N	-0.36428162	1.25696443	1.50309751	F	10.67092562	9.02889182	10.92314857
C	-0.21015521	0.70719815	2.72860387	F	8.85295097	10.89721220	11.11223588
C	-0.43921425	1.44500181	3.88033472	N	11.00995667	9.69515342	7.99219853
H	-0.31120133	0.97557956	4.84575242	N	9.47463064	11.74627260	8.28230920
C	-0.83443580	2.78374742	3.80202796	N	12.49818561	10.68355994	12.68466515
C	-0.98160506	3.32363818	2.52409456	N	10.69515145	12.52332994	12.83764683
H	-1.28303457	4.35058229	2.37472417	C	11.78097766	8.59614082	7.92165634
C	-0.73993936	2.53407361	1.40959071	H	11.81902647	7.99570252	8.82211049
H	-0.84893990	2.92987210	0.40710257	C	12.48428675	8.24495504	6.78611623
C	-1.08779476	3.57650689	5.07752032	H	13.08160131	7.34182661	6.80127484
C	0.19473033	3.57319891	5.93253989	C	12.41785098	9.04994735	5.63924261
H	0.49000599	2.55882349	6.20885540	C	11.62061936	10.18808511	5.72267609
H	1.02156102	4.03941320	5.39123278	H	11.52863798	10.85075779	4.87452024
C	-2.22747228	2.89576269	5.86146138	C	10.93046817	10.48669104	6.89621442
H	-3.14695477	2.88047464	5.27127297	C	10.06669057	11.66476345	7.06711635
H	-1.97018267	1.86820894	6.12748962	C	9.86601289	12.63406640	6.08552458
C	-1.48392148	5.02723408	4.78452742	H	10.35986786	12.52511979	5.13083042
H	-0.69623224	5.55604758	4.24180064	C	9.04529693	13.73120423	6.33065842
H	-1.65153688	5.54807561	5.72976906	C	8.44624307	13.79669603	7.59763163
H	-2.40648954	5.08182477	4.20112798	H	7.79661891	14.62036737	7.86618262
F	1.69065746	0.47575283	-0.65677341	C	8.68014672	12.80277036	8.52760422
N	0.36362043	-1.25719143	1.50308371	H	8.23107387	12.82798952	9.51279400
C	0.20968937	-0.70737855	2.72859243	C	13.18386455	8.66440835	4.38009295
C	0.43882188	-1.44517309	3.88031204	C	13.00224441	9.68894895	3.25517625
H	0.31108161	-0.97568685	4.84573018	H	13.36747345	10.67666559	3.54872582
C	0.83381839	-2.78398346	3.80200609	H	13.57125767	9.36429006	2.38110925
C	0.98057122	-3.32399065	2.52406548	H	11.95374562	9.78007646	2.95971672
H	1.28173953	-4.35101043	2.37467677	C	14.68402556	8.55678701	4.71506486
C	0.73893608	-2.53440811	1.40956888	H	15.07410477	9.51839091	5.05790040
H	0.84772608	-2.93026346	0.40707930	C	12.67379190	7.29448305	3.89091202
C	1.08765063	-3.57657916	5.07751212	H	12.81260640	6.52485911	4.65329540
C	-0.19423988	-3.57258426	5.93347611	H	11.61119255	7.34502843	3.64042675
H	-0.48895235	-2.55803858	6.20977071	C	8.79684482	14.82810777	5.30270273
H	-1.02164336	-4.03864738	5.39291401	C	7.28292952	14.93275950	5.03459465
C	2.22814574	-2.89602122	5.86045067	H	6.73171782	15.16519885	5.94820336
H	3.14718843	-2.88116138	5.26956801	H	7.09409281	15.72912100	4.30985715

($^{t\text{-Bu}}$bpy)NiF_2 | multiplicity 3 (UKS)				[($^{t\text{-Bu}}$bpy)NiF]$_2$ | multiplicity 1 (UKS)			
H	1.97136651	-1.86831917	6.12640834	H	6.89700381	13.99481190	4.62729165
C	1.48306339	-5.02753692	4.78463191	C	9.30523870	16.16664826	5.87416367
H	0.69485736	-5.55620632	4.24250831	H	10.37868291	16.11852294	6.07355374
H	1.65105381	-5.54820316	5.72990588	H	8.79276703	16.42052424	6.80470581
H	2.40526881	-5.08261780	4.20070765	C	9.51292473	14.55202721	3.97633690
H	0.01791635	4.13875676	6.85062363	H	9.17256268	13.61582364	3.52598976
H	-2.41580332	3.45073824	6.78391959	H	9.29532044	15.36223546	3.27679376
H	2.41699487	-3.45083345	6.78290167	H	10.59697577	14.50090551	4.10681017
H	-0.01694526	-4.13797168	6.85157631	C	13.38485524	9.68805031	12.54340444
				H	13.02968923	8.82198537	11.99859721
				C	14.67378902	9.74610276	13.04949126
				H	15.33012306	8.90107119	12.89364240
				C	12.89174824	11.79589182	13.35694975
				C	11.85835002	12.83930961	13.45335364
				C	12.03662368	14.05700379	14.10746844
				C	11.01177960	14.99829146	14.14262104
				C	9.81734798	14.65095087	13.49410998
				H	8.97492534	15.33088647	13.47581221
				C	9.70004908	13.42659448	12.86579035
				H	8.79080654	13.13084445	12.35740213
				C	11.14681554	16.34686899	14.83824597
				C	10.92940026	17.46549497	13.80030331
				H	9.94459139	17.38984144	13.33407106
				H	11.00212543	18.43975926	14.29073970
				H	11.68786012	17.41484657	13.01508366
				C	10.06975182	16.45392233	15.93545188
				H	9.06527537	16.36949413	15.51536935
				H	15.23690185	8.25713404	3.82109446
				H	14.86604093	7.81279106	5.49364712
				H	13.22740454	6.99549591	2.99691487
				H	9.12153944	16.96707671	5.15274655
				C	15.10139011	10.88181688	13.74262932
				C	14.17065370	11.91361492	13.88525615
				H	12.98190534	14.26381778	14.58765731
				C	12.52578966	16.53150093	15.48025434
				H	10.19891449	15.66643504	16.68227213
				H	10.15199639	17.42285324	16.43512376
				C	16.50410097	11.03379589	14.31799372
				H	14.44699482	12.81935791	14.40859407

($^{t\text{-Bu}}$bpy)NiF_2 | multiplicity 3 (UKS)				[($^{t\text{-Bu}}$bpy)NiF$]_2$ | multiplicity 1 (UKS)			
				H	12.56808345	17.51172840	15.96071925
				H	12.71877964	15.77270534	16.24315665
				H	13.32435382	16.48582210	14.73514749
				C	17.36273830	9.78771138	14.07616825
				H	16.92746204	8.90385954	14.54982094
				H	17.48484700	9.58498860	13.00903256
				H	18.35454545	9.94749141	14.50505176
				C	16.40471486	11.27891669	15.83620270
				H	15.83441880	12.18354703	16.05903044
				H	15.92100942	10.43453622	16.33357671
				H	17.40805088	11.39877603	16.25330088
				C	17.18543198	12.24443171	13.65014859
				H	18.19056204	12.37341398	14.06015312
				H	17.26804125	12.09328311	12.57084201
				H	16.62328228	13.16393888	13.82740254

[($^{t\text{-Bu}}$bpy)NiF$]_2$ | multiplicity 3 (UKS)				PyF_4–PyF_4 | multiplicity 1 (RKS)			
Ni	9.98756165	10.31594817	9.53740971	N	0.12875268	0.08289283	-3.52118660
Ni	10.70225803	10.76786979	11.93726661	C	-0.02784898	-0.01523105	0.73741180
F	10.69331319	9.02558423	10.91793745	C	1.12827568	0.06726687	1.51029672
F	8.87124633	10.88634380	11.11296396	C	1.02004034	0.02380392	2.89232743
N	11.00730118	9.68291841	7.98412592	N	-0.12395258	-0.08820105	3.52126916
N	9.49251123	11.74660686	8.28750653	C	-1.22174411	-0.16463413	2.81006205
N	12.51965425	10.69129419	12.67348219	C	-1.23436920	-0.13512758	1.42338215
N	10.70351528	12.51906902	12.83136359	C	0.02356313	0.02073622	-0.73742007
C	11.76995816	8.57810527	7.90713399	C	0.56813945	1.10392257	-1.42344662
H	11.80929559	7.97591484	8.80645080	C	0.59326778	1.08041572	-2.81001096
C	12.46398993	8.22378670	6.76721885	C	-0.38683485	-0.94454535	-2.89231219
H	13.05510590	7.31648396	6.77748543	C	-0.46672135	-1.02960503	-1.51019851
C	12.39734005	9.03159153	5.62171006	F	-2.39027390	-0.20041090	0.75863473
C	11.60935045	10.17540677	5.71162951	F	-2.37350288	-0.27330959	3.47296521
H	11.51758900	10.84063712	4.86534360	F	2.12329074	0.09401704	3.63772959
C	10.92783156	10.47728508	6.88961772	F	2.32726535	0.16673290	0.93172363
C	10.07492913	11.66145735	7.06750384	F	1.04213376	2.16060496	-0.75928761
C	9.87416140	12.63339832	6.08831503	F	-0.98741549	-2.11402299	-0.93115058
H	10.35994364	12.52165692	5.12979939	F	-0.84883337	-1.94872089	-3.63792946
C	9.06377842	13.73623516	6.34068696	F	1.10999809	2.11563561	-3.47269144
C	8.47444125	13.80509192	7.61246034				
H	7.83303781	14.63336221	7.88647452				
C	8.70791137	12.80893996	8.53974886				

[($^{t\text{-Bu}}$bpy)NiF]$_2$ | multiplicity 3 (UKS)				PyF$_4$–PyF$_4$ | multiplicity 1 (RKS)
H	8.26677831	12.83669847	9.52845056	
C	13.15580639	8.64371093	4.35873946	
C	12.97128836	9.66871426	3.23470285	
H	13.34117963	10.65538163	3.52610409	
H	13.53451219	9.34225319	2.35749697	
H	11.92139149	9.76289038	2.94493729	
C	14.65728297	8.53266746	4.68722954	
H	15.05032671	9.49307747	5.03035132	
C	12.64063219	7.27497264	3.87154850	
H	12.78106282	6.50446207	4.63276002	
H	11.57705177	7.32792360	3.62558938	
C	8.81478590	14.83553474	5.31555291	
C	7.30014418	14.94434711	5.05346789	
H	6.75330150	15.17610116	5.96992735	
H	7.11035635	15.74270361	4.33121043	
H	6.91035162	14.00821333	4.64574876	
C	9.32846509	16.17201926	5.88694065	
H	10.40248608	16.12094679	6.08276818	
H	8.81958481	16.42641399	6.81925414	
C	9.52547246	14.56004522	3.98609740	
H	9.18126974	13.62564693	3.53490960	
H	9.30742369	15.37233137	3.28906244	
H	10.60991859	14.50602232	4.11286644	
C	13.41395875	9.70293574	12.52902558	
H	13.06758294	8.83880989	11.97543125	
C	14.69970683	9.76556423	13.04222940	
H	15.36278306	8.92654261	12.88243884	
C	12.90206419	11.80120199	13.35684034	
C	11.86313566	12.83885078	13.45213381	
C	12.03394690	14.05701911	14.10772148	
C	11.00565042	14.99460819	14.13797515	
C	9.81507985	14.64307232	13.48419716	
H	8.97059275	15.32037038	13.46162191	
C	9.70479400	13.41847449	12.85543105	
H	8.79889510	13.11965527	12.34277907	
C	11.13340887	16.34507397	14.83124318	
C	10.92440246	17.46030969	13.78786295	
H	9.94383284	17.38214720	13.31325545	
H	10.99212214	18.43620372	14.27574509	

[($^{t\text{-Bu}}$bpy)NiF]$_2$ | multiplicity 3 (UKS)				PyF$_4$–PyF$_4$ | multiplicity 1 (RKS)
H	11.69007605	17.40764370	13.00974171	
C	10.04651613	16.45515243	15.91840748	
H	9.04570285	16.36887926	15.48997381	
H	15.20644088	8.23378421	3.79051264	
H	14.84068646	7.78694777	5.46377209	
H	13.18954747	6.97529566	2.97492413	
H	9.14464999	16.97380177	5.16690719	
C	15.11536117	10.89808422	13.74843449	
C	14.17665790	11.92177926	13.89520319	
H	12.97637249	14.26743307	14.59232816	
C	12.50643126	16.53255384	15.48513896	
H	10.16952017	15.67038374	16.66918395	
H	10.12355273	17.42565529	16.41557710	
C	16.51186192	11.05184126	14.33869445	
H	14.44324171	12.82386601	14.42977255	
H	12.54422235	17.51456975	15.96213950	
H	12.69218012	15.77677148	16.25284577	
H	13.31191572	16.48401124	14.74770579	
C	17.39162433	9.82816371	14.06107100	
H	16.97011580	8.92285152	14.50565392	
H	17.51944101	9.66099373	12.98846722	
H	18.37946559	9.99073287	14.49822441	
C	16.39391571	11.24207933	15.86365218	
H	15.80618487	12.12915067	16.11037921	
H	15.91867112	10.37320266	16.32590199	
H	17.39080975	11.36286352	16.29564144	
C	17.18088097	12.29508393	13.72089835	
H	18.17999814	12.42393301	14.14550542	
H	17.27634693	12.18410262	12.63776939	
H	16.60422212	13.19987604	13.92495614	

7 Single crystal X-ray diffraction

Low-temperature diffraction data (ϕ-and ω-scans) were collected on a Bruker AXS D8 VENTURE KAPPA diffractometer coupled to a PHOTON II CPAD detector with Mo K_α radiation (λ = 0.71073 Å) from an IμS micro-source for the structure of compounds **3a**, **3c**, **3h**, **3k**, **3l**, and **4a**. Low-temperature diffraction data (ϕ-and ω-scans) were also collected on a Bruker AXS D8 VENTURE KAPPA diffractometer coupled to a PHOTON II CPAD detector with Cu K_α radiation (λ = 1.54178 Å) from an IμS micro-source for the structure of compound **3b**, **3d**, **3e**, **3f**, **3i**, **4b**, **4c**, **4d**, and **5**. The structures were solved by direct methods using SHELXS[30] and refined against F^2 on all data by full-matrix least squares with SHELXL-2019[31] using established refinement techniques.[32] All non-hydrogen atoms were refined anisotropically. All hydrogen atoms were included into the model at geometrically calculated positions and refined using a riding model. The isotropic displacement parameters of all hydrogen atoms were fixed to 1.2 times the U value of the atoms they are linked to (1.5 times for methyl groups). All disordered atoms were refined with the help of similarity restraints on the 1,2- and 1,3-distances and displacement parameters as well as enhanced rigid bond restraints for anisotropic displacement parameters.

Suitable crystals of **3a** were grown from a slow evaporation of a DCM solution. The tert-butyl groups were disordered over two positions.

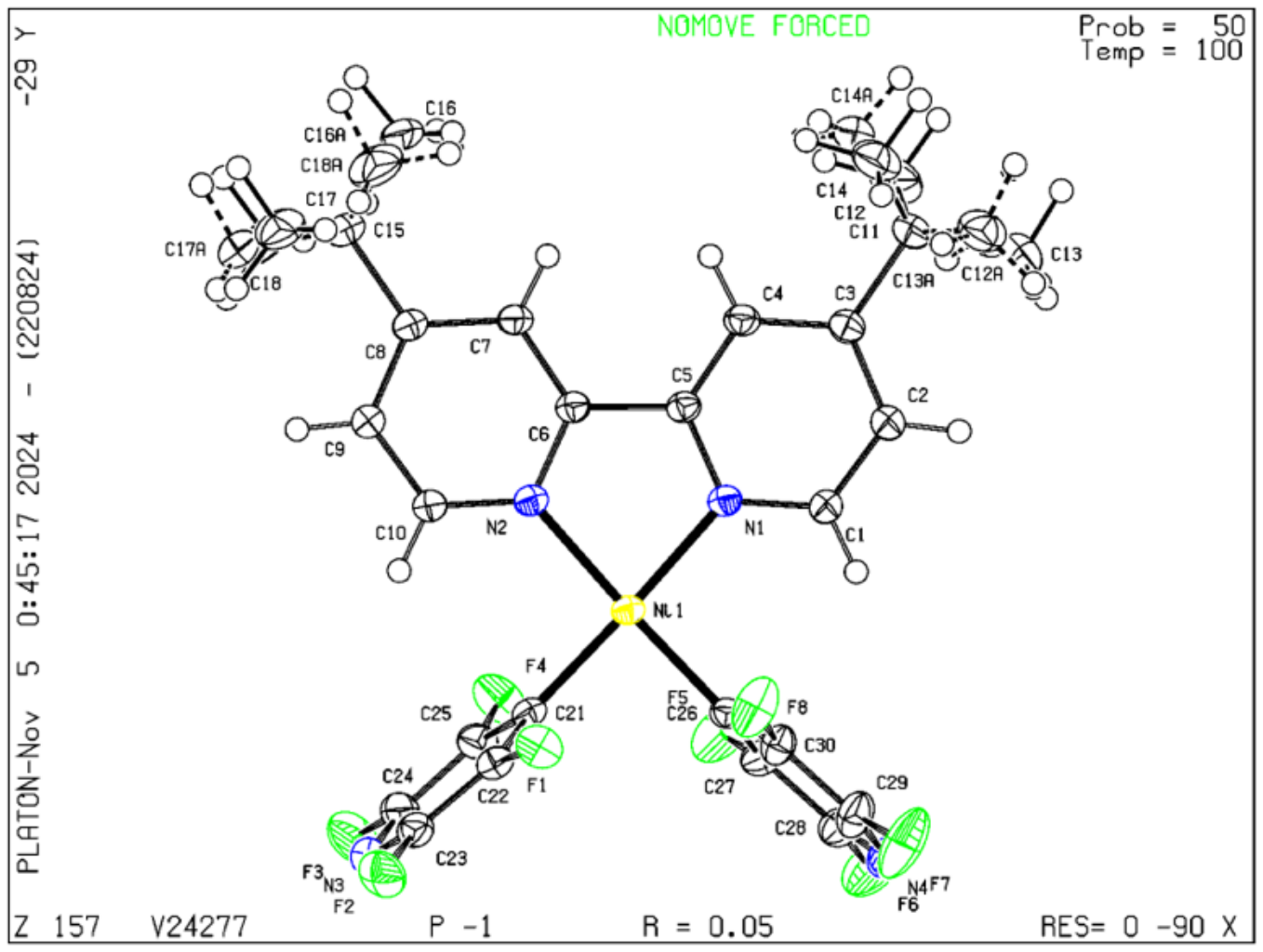

Table S11. Crystal data and structure refinement for 3a (CCDC 2552391)

Empirical formula	$C_{28}H_{24}N_4F_8Ni$
Formula weight	627.22
Temperature/K	100(2)
Crystal system	triclinic
Space group	P-1
a/Å	9.7789(17)
b/Å	11.524(4)
c/Å	11.775(3)
α/°	95.185(11)
β/°	93.165(13)
γ/°	95.429(18)
Volume/Å^3	1312.8(6)
Z	2
ρ_{calc}g/cm^3	1.587
μ/mm^{-1}	0.822
F(000)	640.0
Crystal size/mm^3	0.3 × 0.15 × 0.1
Radiation	MoKα (λ = 0.71073)
2Θ range for data collection/°	4.738 to 72.738
Index ranges	-16 ≤ h ≤ 15, -19 ≤ k ≤ 19, -19 ≤ l ≤ 19
Reflections collected	66562
Independent reflections	12752 [R_{int} = 0.0587, R_{sigma} = 0.0405]
Data/restraints/parameters	12752/469/438
Goodness-of-fit on F^2	1.019
Final R indexes [I>=2σ (I)]	R_1 = 0.0472, wR_2 = 0.1224
Final R indexes [all data]	R_1 = 0.0649, wR_2 = 0.1327
Largest diff. peak/hole / e Å^{-3}	1.48/-1.21

Suitable crystals of **3b** were grown from a slow evaporation of a DCM solution. Compound **3b** crystallizes in the triclinic space group *P*-1 with one molecule in the asymmetric unit.

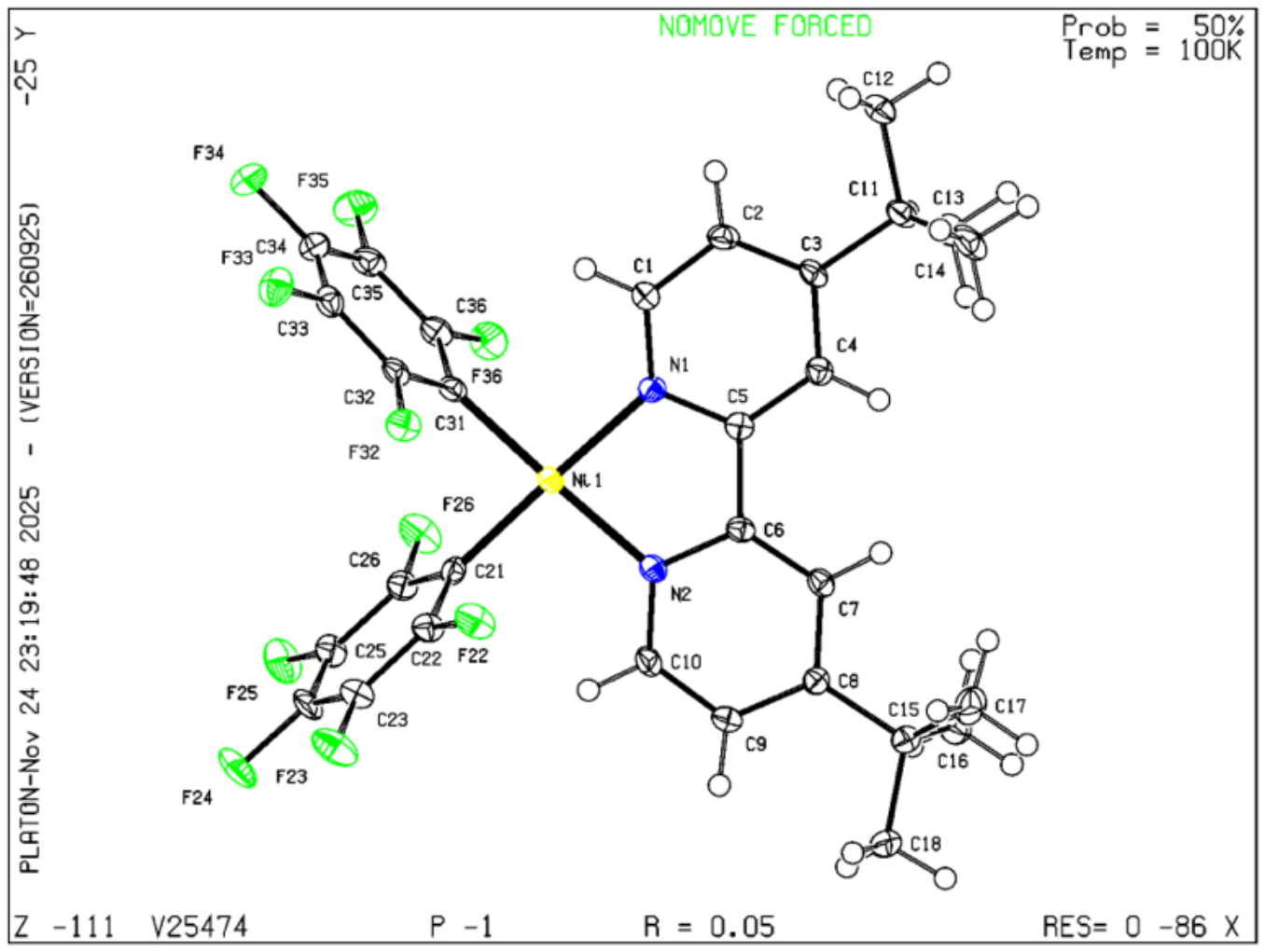

Table S12. Crystal data and structure refinement for 3b (CCDC 2552382)

Empirical formula	$C_{30}H_{24}N_2F_{10}Ni$
Formula weight	661.22
Temperature/K	100(2)
Crystal system	triclinic
Space group	P-1
a/Å	10.3245(3)
b/Å	11.1188(3)
c/Å	12.1405(3)
α/°	97.3400(10)
β/°	91.7261(11)
γ/°	97.0254(11)
Volume/Å^3	1370.40(6)
Z	2
ρ_{calc}g/cm^3	1.602
μ/mm^{-1}	1.868
F(000)	672.0
Crystal size/mm^3	0.3 × 0.15 × 0.1
Radiation	CuKα (λ = 1.54178)
2Θ range for data collection/°	7.35 to 148.92
Index ranges	-12 ≤ h ≤ 12, -13 ≤ k ≤ 13, -15 ≤ l ≤ 15
Reflections collected	25813
Independent reflections	5552 [R_{int} = 0.0535, R_{sigma} = 0.0386]
Data/restraints/parameters	5552/0/394
Goodness-of-fit on F^2	1.204
Final R indexes [I>=2σ (I)]	R_1 = 0.0535, wR_2 = 0.1131
Final R indexes [all data]	R_1 = 0.0667, wR_2 = 0.1254
Largest diff. peak/hole / e Å^{-3}	0.69/-0.47

Suitable crystals of **3c** were grown from a slow evaporation of a DCM solution. Compound **3c** crystallizes in the triclinic space group *P*-1 with one molecule in the asymmetric unit. One of the trifluoromethyl groups was disordered over three positions.

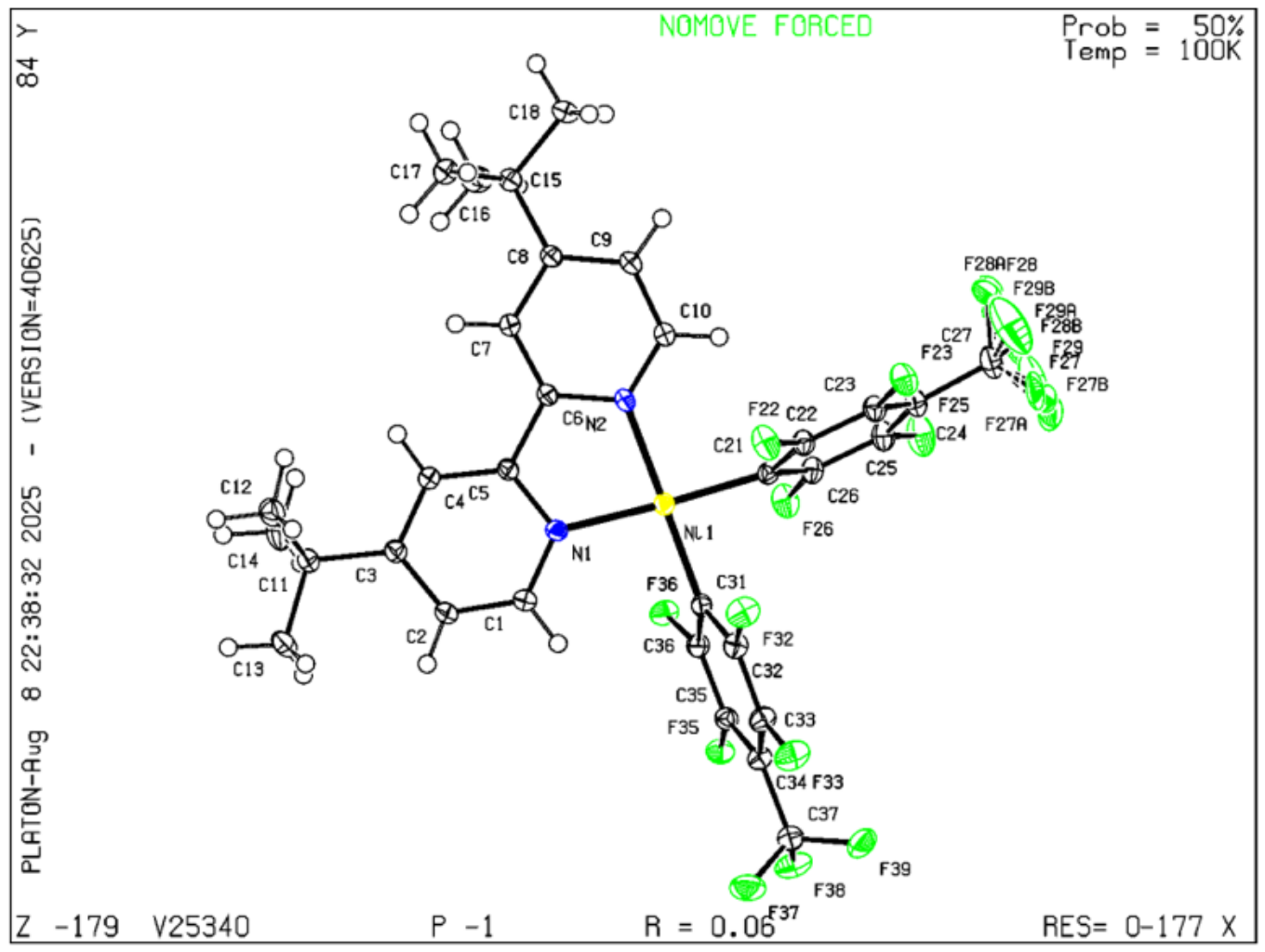

Table S13. Crystal data and structure refinement for 3c (CCDC 2552392)

Empirical formula	$C_{32}H_{24}N_2F_{14}Ni$
Formula weight	761.24
Temperature/K	100(2)
Crystal system	triclinic
Space group	P-1
a/Å	10.584(3)
b/Å	11.487(3)
c/Å	12.779(3)
α/°	88.510(11)
β/°	89.188(9)
γ/°	77.650(14)
Volume/Å^3	1517.1(6)
Z	2
ρ_{calc}g/cm^3	1.666
μ/mm^{-1}	0.753
F(000)	768.0
Crystal size/mm^3	0.2 × 0.2 × 0.2
Radiation	MoKα (λ = 0.71073)
2Θ range for data collection/°	4.754 to 72.684
Index ranges	-17 ≤ h ≤ 17, -19 ≤ k ≤ 19, -21 ≤ l ≤ 21
Reflections collected	71731
Independent reflections	14695 [R_{int} = 0.0483, R_{sigma} = 0.0342]
Data/restraints/parameters	14695/289/505
Goodness-of-fit on F^2	1.124
Final R indexes [I>=2σ (I)]	R_1 = 0.0589, wR_2 = 0.1058
Final R indexes [all data]	R_1 = 0.0910, wR_2 = 0.1294
Largest diff. peak/hole / e Å^{-3}	1.61/-1.31

Suitable crystals of **3d** were grown from a slow evaporation of a DCM solution. Compound **3d** crystallizes in the monoclinic space group *P*2$_1$/*c* with one molecule in the asymmetric unit along with half a molecule of dichloromethane. The dichloromethane was located near a crystallographic inversion center and disordered appropriately.

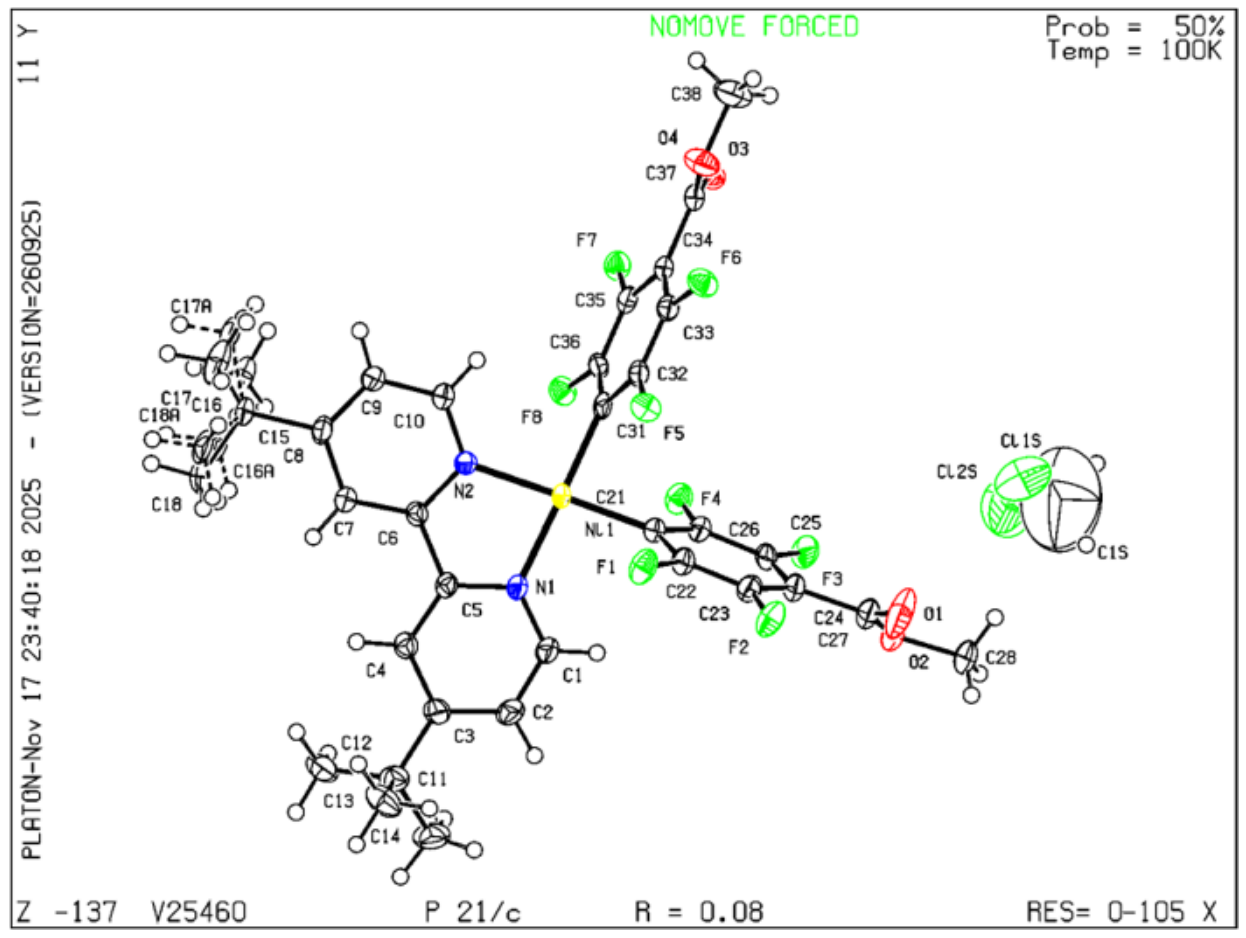

Table S14. Crystal data and structure refinement for 3d (CCDC 2552394)

Empirical formula	$C_{34.5}H_{31}N_2O_4F_8NiCl$
Formula weight	783.77
Temperature/K	100(2)
Crystal system	monoclinic
Space group	P2$_1$/c
a/Å	12.1007(7)
b/Å	22.1669(13)
c/Å	12.2237(7)
α/°	90
β/°	94.433(3)
γ/°	90
Volume/Å^3	3269.0(3)
Z	4
ρ_{calc}g/cm^3	1.593
μ/mm^{-1}	2.407
F(000)	1604.0
Crystal size/mm^3	0.3 × 0.2 × 0.15
Radiation	CuKα (λ = 1.54178)
2Θ range for data collection/°	7.328 to 150.104
Index ranges	-15 ≤ h ≤ 14, -27 ≤ k ≤ 24, -15 ≤ l ≤ 15
Reflections collected	72175
Independent reflections	6680 [R_{int} = 0.0682, R_{sigma} = 0.0300]
Data/restraints/parameters	6680/66/508
Goodness-of-fit on F^2	1.126
Final R indexes [I>=2σ (I)]	R_1 = 0.0814, wR_2 = 0.1969
Final R indexes [all data]	R_1 = 0.0961, wR_2 = 0.2110
Largest diff. peak/hole / e Å^{-3}	0.51/-1.27

Suitable crystals of **3e** were grown from a slow evaporation of a DCM solution. Compound **3e** crystallizes in the monoclinic space group *P*2$_1$/*n* with one molecule in the asymmetric unit.

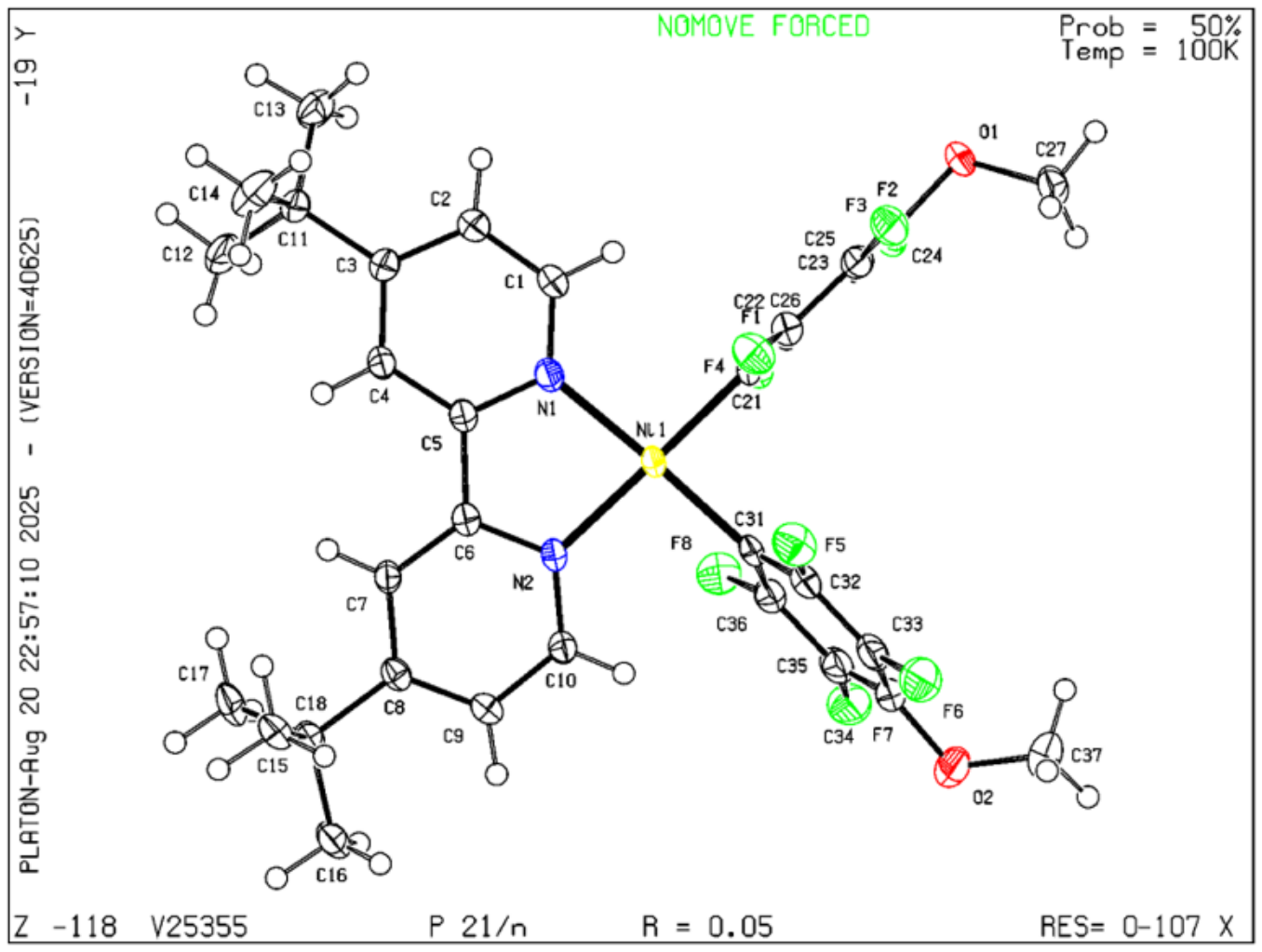

Table S15. Crystal data and structure refinement for 3e (CCDC 2552389)

Empirical formula	$C_{32}H_{30}N_2O_2F_8Ni$
Formula weight	685.29
Temperature/K	100(2)
Crystal system	monoclinic
Space group	P2$_1$/n
a/Å	10.9876(9)
b/Å	23.261(2)
c/Å	11.4659(9)
α/°	90
β/°	93.838(5)
γ/°	90
Volume/Å^3	2923.9(4)
Z	4
ρ_{calc}g/cm^3	1.557
μ/mm^{-1}	1.723
F(000)	1408.0
Crystal size/mm^3	0.25 × 0.2 × 0.15
Radiation	CuKα (λ = 1.54178)
2Θ range for data collection/°	7.6 to 149.208
Index ranges	-13 ≤ h ≤ 13, -28 ≤ k ≤ 29, -14 ≤ l ≤ 14
Reflections collected	43806
Independent reflections	5963 [R_{int} = 0.0520, R_{sigma} = 0.0254]
Data/restraints/parameters	5963/0/414
Goodness-of-fit on F^2	1.173
Final R indexes [I>=2σ (I)]	R_1 = 0.0534, wR_2 = 0.1133
Final R indexes [all data]	R_1 = 0.0658, wR_2 = 0.1225
Largest diff. peak/hole / e Å^{-3}	0.77/-0.44

Suitable crystals of **3f** were grown by vapor diffusion of hexane into a THF solution. Compound **3f** crystallizes in the monoclinic space group *P*2_1/*n* with two molecules in the asymmetric unit.

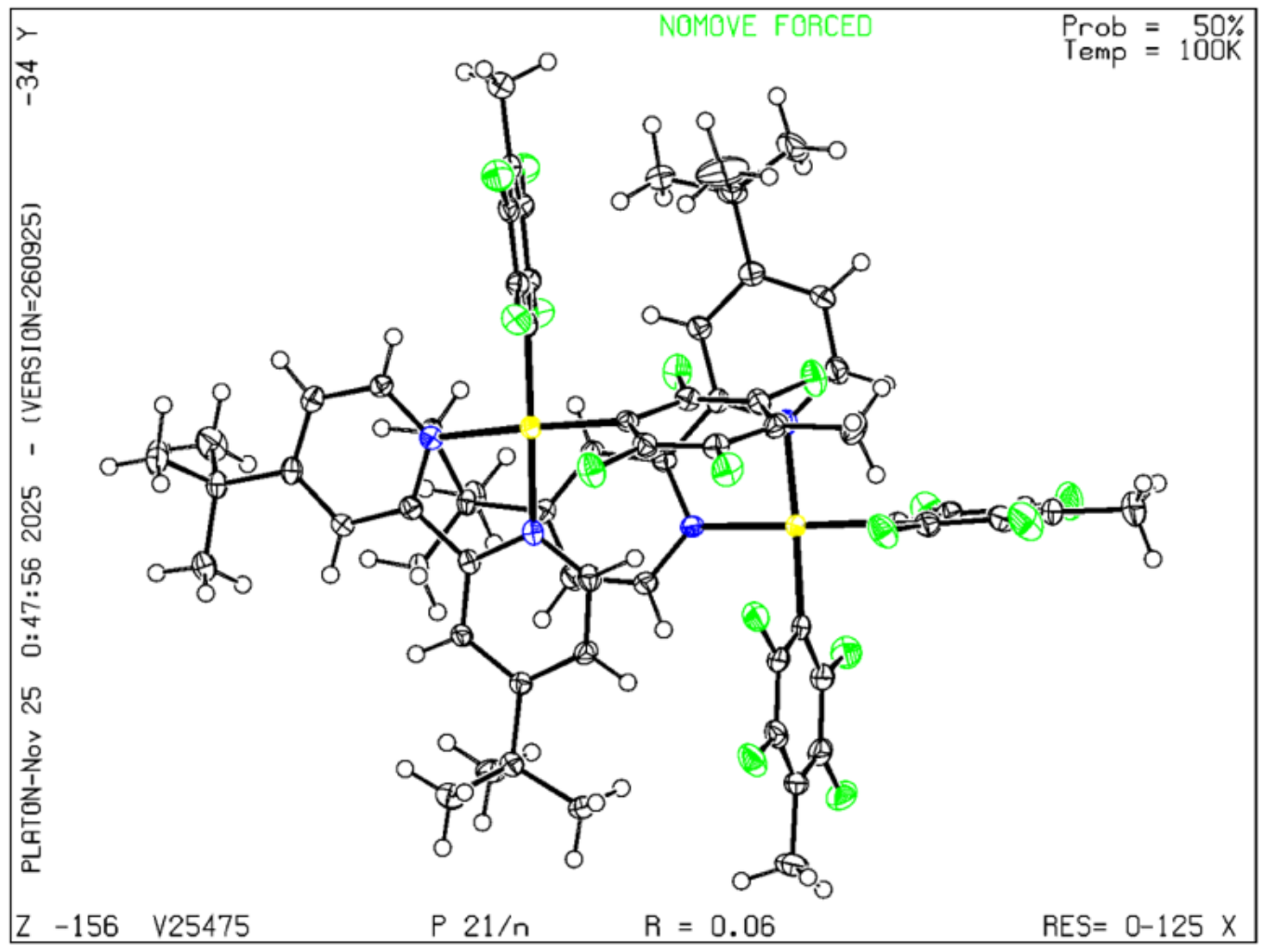

Table S16. Crystal data and structure refinement for 3f (CCDC 2552396)

Empirical formula	$C_{32}H_{30}N_2F_8Ni$
Formula weight	653.29
Temperature/K	100(2)
Crystal system	monoclinic
Space group	$P2_1/n$
a/Å	11.6158(4)
b/Å	35.5857(14)
c/Å	14.2273(6)
α/°	90
β/°	94.9298(18)
γ/°	90
Volume/Å^3	5859.2(4)
Z	8
ρ_{calc}g/cm^3	1.481
μ/mm^{-1}	1.637
F(000)	2688.0
Crystal size/mm^3	0.25 × 0.2 × 0.15
Radiation	CuKα (λ = 1.54178)
2Θ range for data collection/°	4.966 to 148.906
Index ranges	-14 ≤ h ≤ 14, -44 ≤ k ≤ 44, -17 ≤ l ≤ 17
Reflections collected	93269
Independent reflections	11950 [R_{int} = 0.0547, R_{sigma} = 0.0279]
Data/restraints/parameters	11950/0/791
Goodness-of-fit on F^2	1.240
Final R indexes [I>=2σ (I)]	R_1 = 0.0614, wR_2 = 0.1175
Final R indexes [all data]	R_1 = 0.0797, wR_2 = 0.1320
Largest diff. peak/hole / e Å^{-3}	0.53/-0.56

Suitable crystals of **3h** were grown from a slow evaporation of a DCM solution. Compound **3h** crystallizes in the monoclinic space group *P*2$_1$/*n* with one molecule in the asymmetric unit. One of the aryl groups was disordered over two positions.

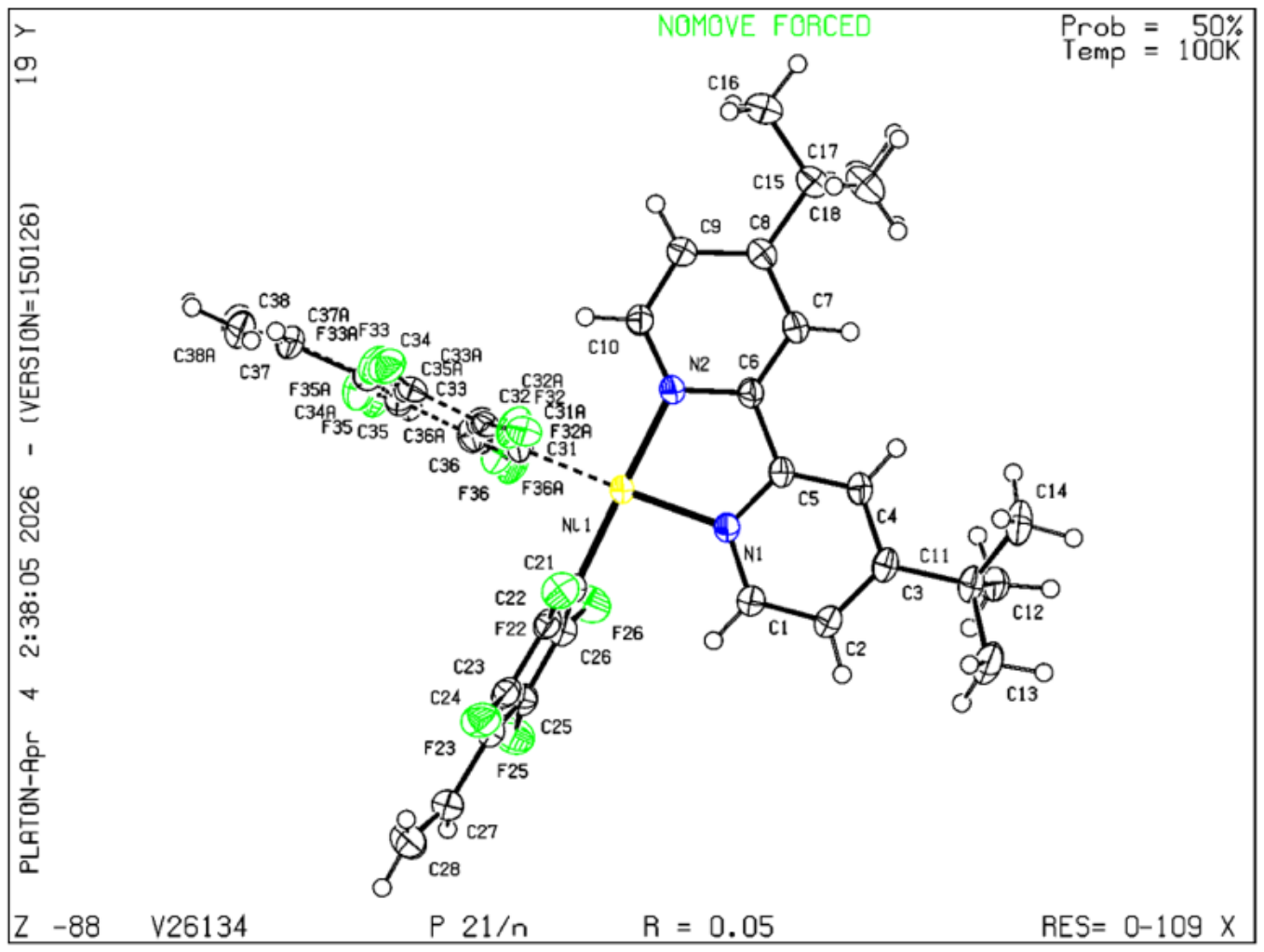

Table S17. Crystal data and structure refinement for 3h (CCDC 2552393)

Empirical formula	$C_{34}H_{30}N_2F_8Ni$
Formula weight	677.31
Temperature/K	100(2)
Crystal system	monoclinic
Space group	P2$_1$/n
a/Å	11.1213(9)
b/Å	23.0878(17)
c/Å	11.6675(10)
α/°	90
β/°	95.637(3)
γ/°	90
Volume/Å^3	2981.3(4)
Z	4
ρ_{calc}g/cm^3	1.509
μ/mm^{-1}	0.729
F(000)	1392.0
Crystal size/mm^3	0.2 × 0.2 × 0.15
Radiation	MoKα (λ = 0.71073)
2Θ range for data collection/°	4.828 to 61.07
Index ranges	-15 ≤ h ≤ 15, -32 ≤ k ≤ 33, -16 ≤ l ≤ 16
Reflections collected	81301
Independent reflections	9100 [R_{int} = 0.0326, R_{sigma} = 0.0182]
Data/restraints/parameters	9100/666/521
Goodness-of-fit on F^2	1.066
Final R indexes [I>=2σ (I)]	R_1 = 0.0465, wR_2 = 0.1150
Final R indexes [all data]	R_1 = 0.0609, wR_2 = 0.1307
Largest diff. peak/hole / e Å^{-3}	0.80/-0.71

Suitable crystals of **3i** were grown from a slow evaporation of a MeCN solution. Compound **3i** crystallizes in the monoclinic space group $P2_1/c$ with one molecule in the asymmetric unit along with one molecule of acetonitrile. One of the aromatic groups was modeled disordered mixture of ortho- and para-PhF_4CN.

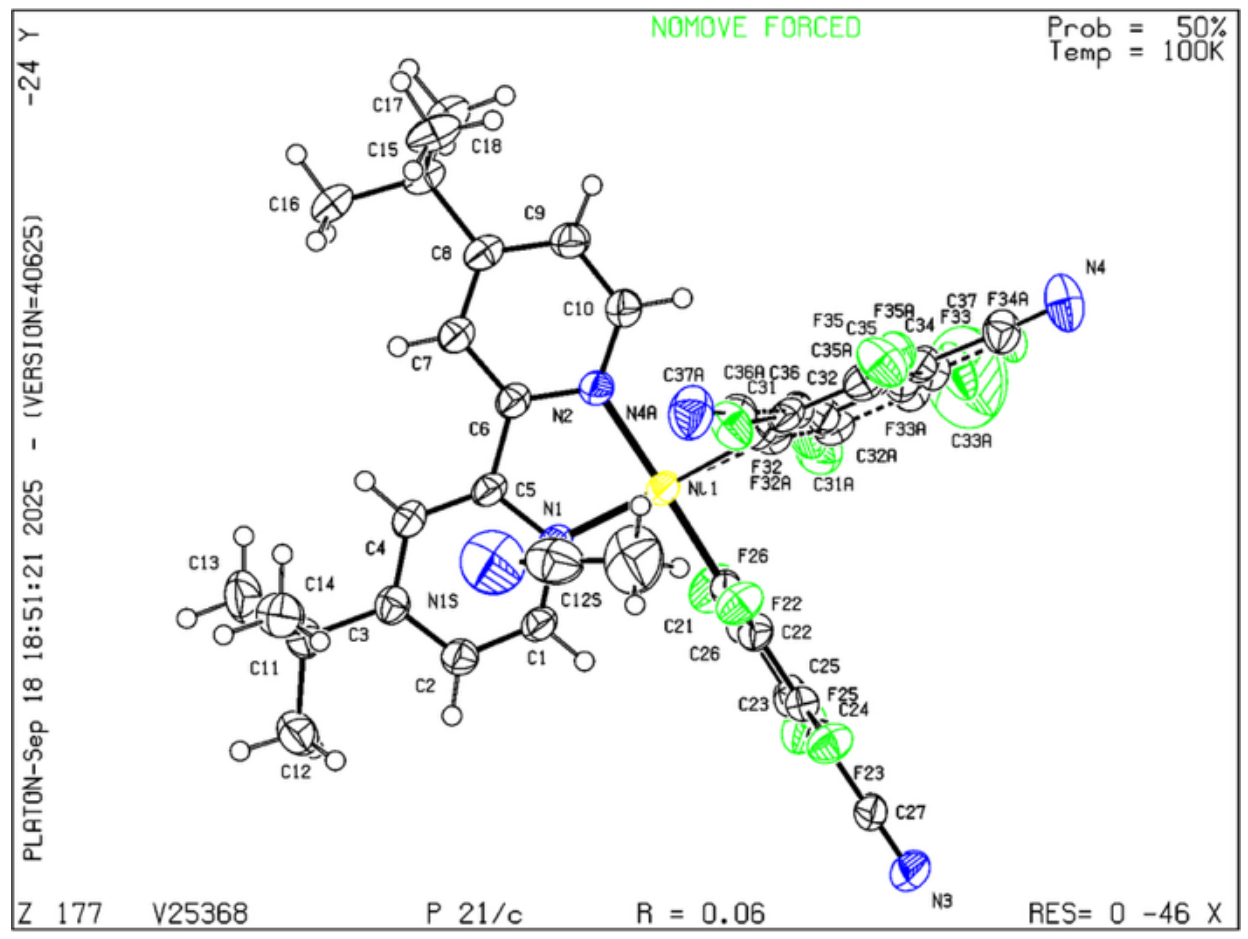

Table S18. Crystal data and structure refinement for 3i (CCDC 2552397)

Empirical formula	$C_{34}H_{27}N_5F_8Ni$
Formula weight	716.31
Temperature/K	100(2)
Crystal system	monoclinic
Space group	$P2_1/c$
a/Å	11.2470(5)
b/Å	16.3202(8)
c/Å	17.5852(8)
α/°	90
β/°	103.292(2)
γ/°	90
Volume/Å^3	3141.4(3)
Z	4
ρ_{calc}g/cm^3	1.515
μ/mm^{-1}	1.615
F(000)	1464.0
Crystal size/mm^3	0.3 × 0.2 × 0.1
Radiation	CuKα (λ = 1.54178)
2Θ range for data collection/°	7.484 to 149.46
Index ranges	-14 ≤ h ≤ 14, -20 ≤ k ≤ 20, -21 ≤ l ≤ 21
Reflections collected	67047
Independent reflections	6400 [R_{int} = 0.0493, R_{sigma} = 0.0237]
Data/restraints/parameters	6400/791/549
Goodness-of-fit on F^2	1.162
Final R indexes [I>=2σ (I)]	R_1 = 0.0643, wR_2 = 0.1359
Final R indexes [all data]	R_1 = 0.0765, wR_2 = 0.1458
Largest diff. peak/hole / e Å^{-3}	0.47/-0.55

Suitable crystals of **3k** were grown from a slow evaporation of a THF solution. Compound **3k** crystallizes in the monoclinic space group *C*2/*c* with half a molecule in the asymmetric unit along with one molecule of tetrahydrofuran.

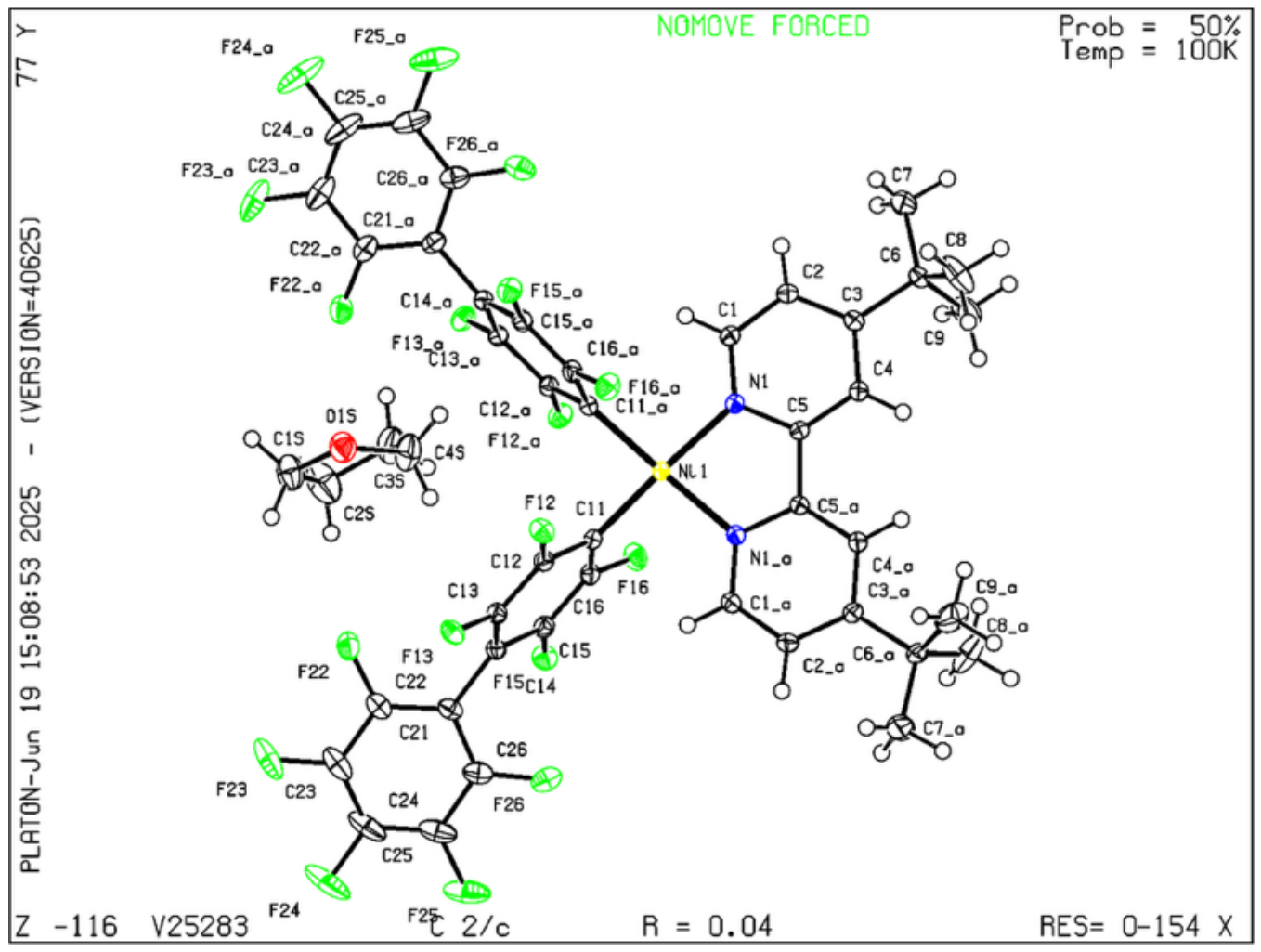

Table S19. Crystal data and structure refinement for 3k (CCDC 2552395)

Empirical formula	$C_{50}H_{40}N_2F_{18}NiO_2$
Formula weight	1101.55
Temperature/K	100(2)
Crystal system	monoclinic
Space group	C2/c
a/Å	12.809(2)
b/Å	22.781(5)
c/Å	16.738(4)
α/°	90
β/°	104.523(13)
γ/°	90
Volume/Å^3	4728.0(17)
Z	4
ρ_{calc}g/cm^3	1.548
μ/mm^{-1}	0.525
F(000)	2240.0
Crystal size/mm^3	0.3 × 0.15 × 0.15
Radiation	MoKα (λ = 0.71073)
2Θ range for data collection/°	4.372 to 61.094
Index ranges	-18 ≤ h ≤ 18, -32 ≤ k ≤ 32, -23 ≤ l ≤ 23
Reflections collected	73648
Independent reflections	7243 [R_{int} = 0.0441, R_{sigma} = 0.0211]
Data/restraints/parameters	7243/0/333
Goodness-of-fit on F^2	1.078
Final R indexes [I>=2σ (I)]	R_1 = 0.0371, wR_2 = 0.0960
Final R indexes [all data]	R_1 = 0.0407, wR_2 = 0.0981
Largest diff. peak/hole / e Å^{-3}	0.50/-0.32

Suitable crystals of **3l** were grown from a slow evaporation of a Et_2O solution. Compound **3l** crystallizes in the monoclinic space group *C*2/*c* with half a molecule in the asymmetric unit along with half a molecule of diethyl ether. The diethyl ether is located near a crystallographic rotation axis and was disordered over two positions. It was refined with the help of similarity restraints on the displacement parameters as well as enhanced rigid bond restraints for the anisotropic displacement parameters.

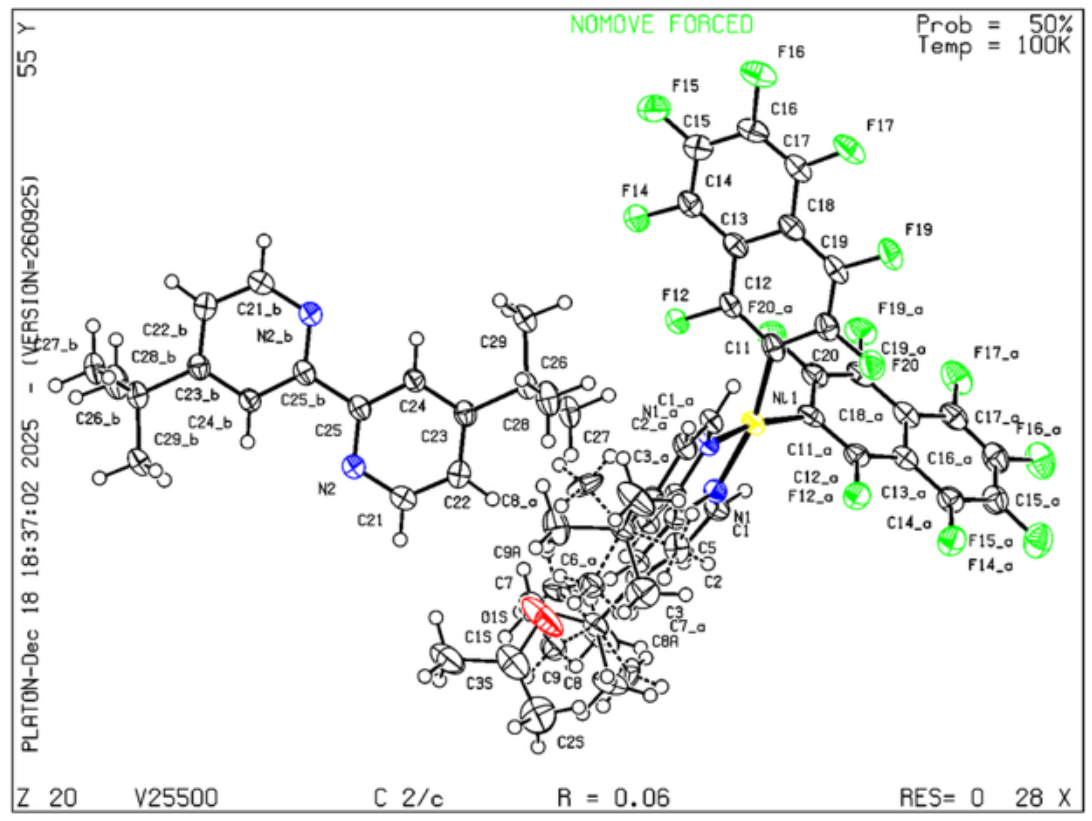

Table S20. Crystal data and structure refinement for 3l (CCDC 2552385)

Empirical formula	$C_{29.5}H_{27}N_2F_7Ni_{0.5}O_{0.5}$
Formula weight	579.88
Temperature/K	100(2)
Crystal system	monoclinic
Space group	C2/c
a/Å	18.0226(17)
b/Å	14.2638(14)
c/Å	21.513(2)
α/°	90
β/°	103.900(4)
γ/°	90
Volume/Å^3	5368.5(9)
Z	8
ρ_{calc}g/cm^3	1.435
μ/mm^{-1}	0.456
F(000)	2392.0
Crystal size/mm^3	0.2 × 0.1 × 0.05
Radiation	MoKα (λ = 0.71073)
2Θ range for data collection/°	3.898 to 50.048
Index ranges	-21 ≤ h ≤ 21, -16 ≤ k ≤ 16, -23 ≤ l ≤ 25
Reflections collected	17946
Independent reflections	4714 [R_{int} = 0.0496, R_{sigma} = 0.0531]
Data/restraints/parameters	4714/81/414
Goodness-of-fit on F^2	1.058
Final R indexes [I>=2σ (I)]	R_1 = 0.0609, wR_2 = 0.1445
Final R indexes [all data]	R_1 = 0.0928, wR_2 = 0.1678
Largest diff. peak/hole / e Å^{-3}	0.43/-0.52

Suitable crystals of **4a** were grown from a slow evaporation of a DCM solution. Compound **4a** crystallizes in the monoclinic space group *C*2/*c* with half a molecule in the asymmetric unit. There are two solvent accessible voids in the cell, A void analysis with the program PLATON[33] revealed the presence of two voids and the program SQUEEZE[34] was used to remove the contribution of the disordered electron density inside this void from the structure factors. SQUEEZE removed the contribution of 66 electrons from each of the two voids with volumes of 286 Å^3, respectively.

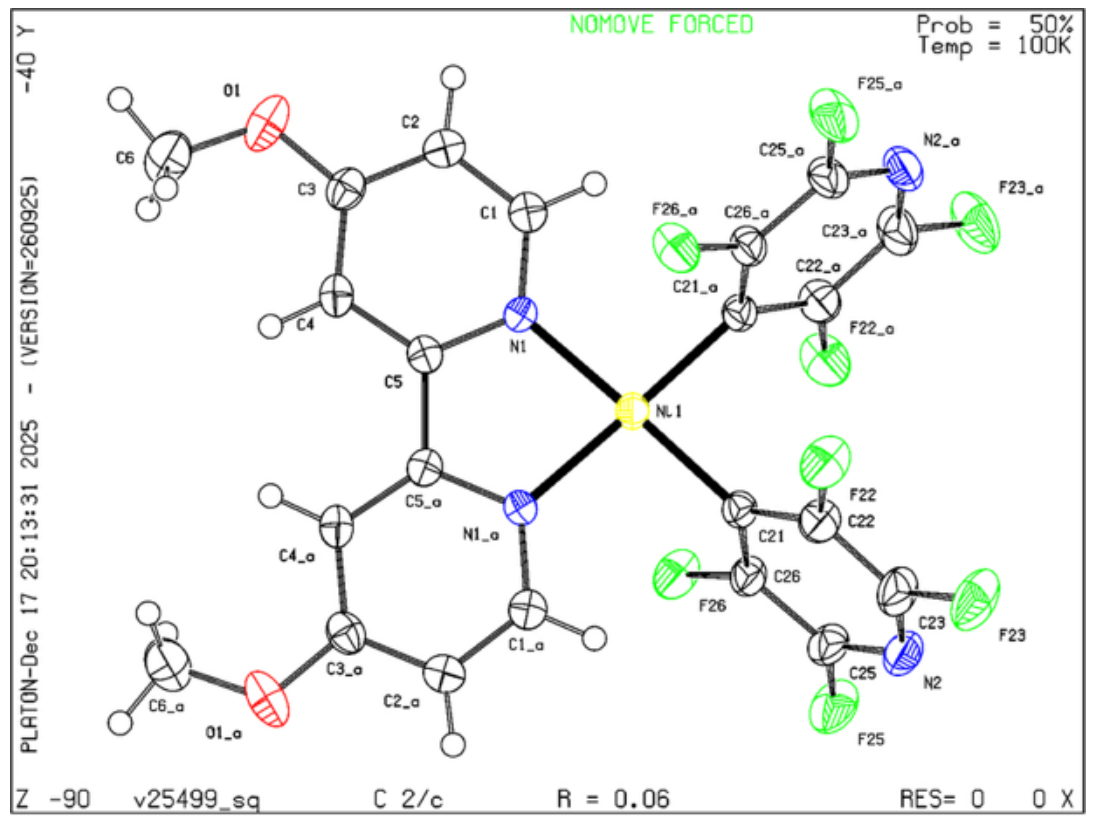

Table S21. Crystal data and structure refinement for 4a (CCDC 2552387)

Empirical formula	$C_{22}H_{12}N_4O_2F_8Ni$
Formula weight	575.07
Temperature/K	100(2)
Crystal system	monoclinic
Space group	C2/c
a/Å	14.7871(9)
b/Å	20.9336(14)
c/Å	8.2388(5)
α/°	90
β/°	96.050(2)
γ/°	90
Volume/Å^3	2536.1(3)
Z	4
ρ_{calc}g/cm^3	1.506
μ/mm^{-1}	0.849
F(000)	1152.0
Crystal size/mm^3	0.2 × 0.2 × 0.15
Radiation	MoKα (λ = 0.71073)
2Θ range for data collection/°	5.54 to 61.106
Index ranges	-19 ≤ h ≤ 21, -29 ≤ k ≤ 27, -11 ≤ l ≤ 11
Reflections collected	18539
Independent reflections	3864 [R_{int} = 0.0634, R_{sigma} = 0.0431]
Data/restraints/parameters	3864/0/169
Goodness-of-fit on F^2	1.054
Final R indexes [I>=2σ (I)]	R_1 = 0.0600, wR_2 = 0.1503
Final R indexes [all data]	R_1 = 0.0825, wR_2 = 0.1665
Largest diff. peak/hole / e Å^{-3}	1.80/-0.73

Suitable crystals of **4b** were grown from a slow evaporation of an acetone solution.

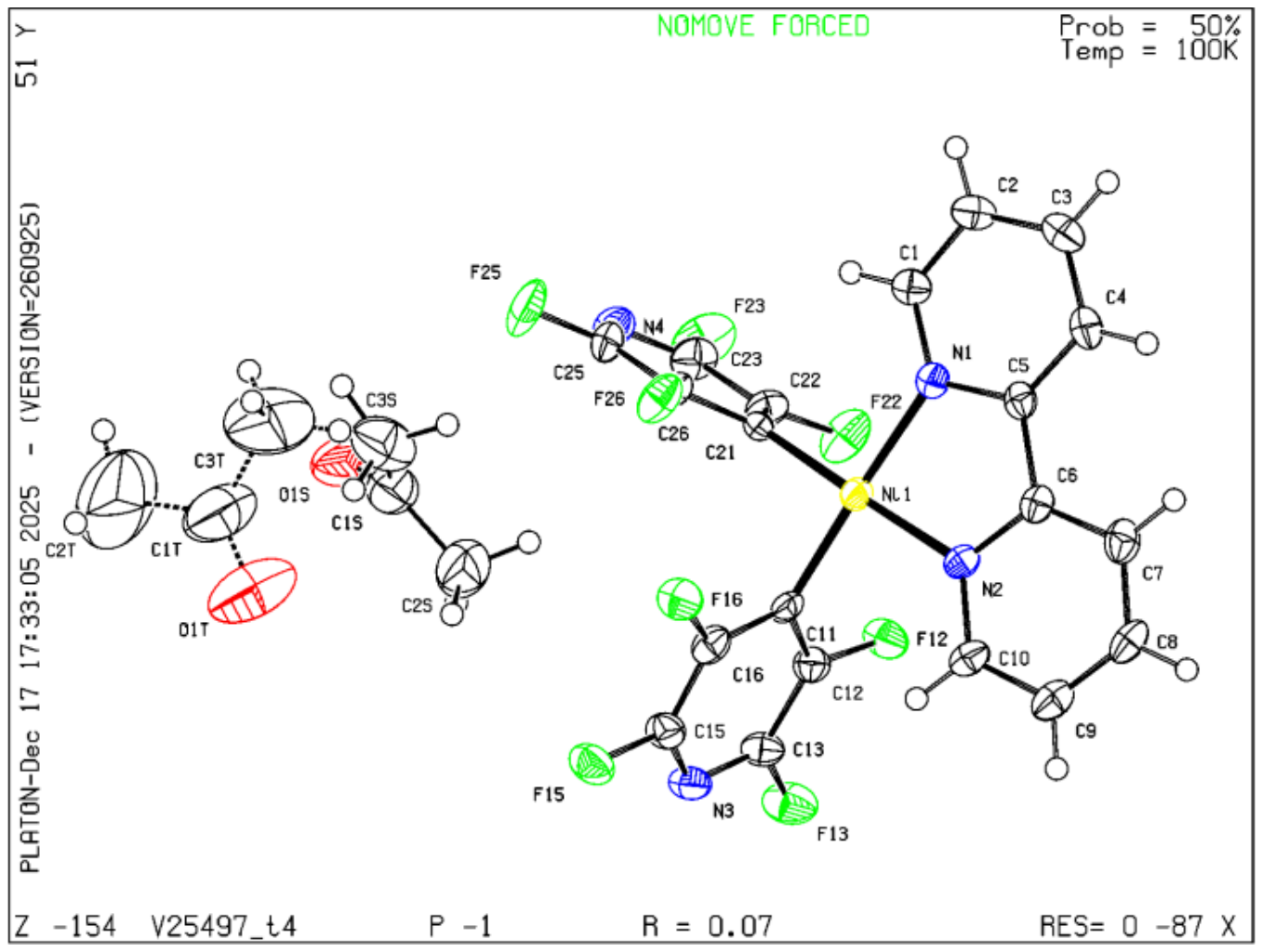

Table S22. Crystal data and structure refinement for 4b (CCDC 2552384)

Empirical formula	$C_{23}H_{14}N_4F_8NiO$
Formula weight	573.09
Temperature/K	100(2)
Crystal system	triclinic
Space group	P-1
a/Å	7.3674(5)
b/Å	12.2063(8)
c/Å	12.6992(8)
α/°	82.505(2)
β/°	89.797(2)
γ/°	81.301(2)
Volume/Å^3	1119.09(13)
Z	2
ρ_{calc}g/cm^3	1.701
μ/mm^{-1}	0.959
F(000)	576.0
Crystal size/mm^3	0.2 × 0.1 × 0.1
Radiation	MoKα (λ = 0.71073)
2Θ range for data collection/°	4.996 to 61.2
Index ranges	? ≤ h ≤ ?, ? ≤ k ≤ ?, ? ≤ l ≤ ?
Reflections collected	5535
Independent reflections	5535 [R_{int} = 0.1403, R_{sigma} = 0.3316]
Data/restraints/parameters	5535/92/375
Goodness-of-fit on F^2	1.072
Final R indexes [I>=2σ (I)]	R_1 = 0.0736, wR_2 = 0.1634
Final R indexes [all data]	R_1 = 0.2626, wR_2 = 0.2026
Largest diff. peak/hole / e Å^{-3}	1.08/-1.30

Suitable crystals of **4c** were grown from a slow evaporation of a DCM solution. Compound **4c** crystallizes in the triclinic space group *P*-1 with two molecules in the asymmetric unit.

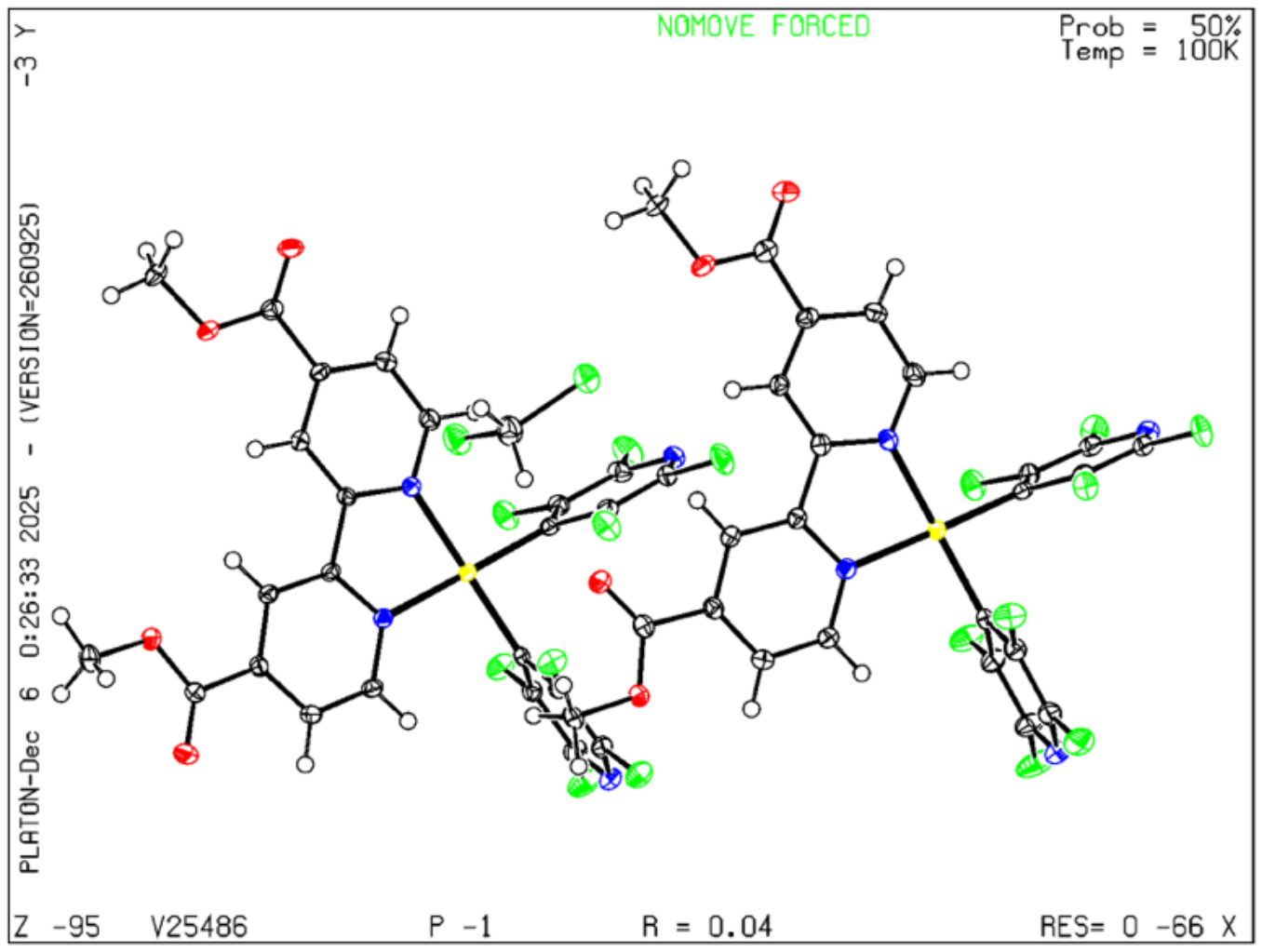

Table S23. Crystal data and structure refinement for 4c (CCDC 2552390)

Empirical formula	$C_{24.5}H_{13}N_4O_4F_8NiCl$
Formula weight	673.55
Temperature/K	100(2)
Crystal system	triclinic
Space group	P-1
a/Å	12.0607(5)
b/Å	12.3152(6)
c/Å	17.4740(8)
α/°	98.6211(17)
β/°	93.1669(16)
γ/°	101.5636(16)
Volume/Å^3	2504.2(2)
Z	4
ρ_{calc}g/cm^3	1.786
μ/mm^{-1}	3.051
F(000)	1348.0
Crystal size/mm^3	0.25 × 0.1 × 0.1
Radiation	CuKα (λ = 1.54178)
2Θ range for data collection/°	5.134 to 149.148
Index ranges	-14 ≤ h ≤ 15, -15 ≤ k ≤ 15, -21 ≤ l ≤ 21
Reflections collected	51801
Independent reflections	10184 [R_{int} = 0.0382, R_{sigma} = 0.0273]
Data/restraints/parameters	10184/0/770
Goodness-of-fit on F^2	1.216
Final R indexes [I>=2σ (I)]	R_1 = 0.0428, wR_2 = 0.0903
Final R indexes [all data]	R_1 = 0.0525, wR_2 = 0.0987
Largest diff. peak/hole / e Å^{-3}	0.45/-0.47

Suitable crystals of **4d** were grown from a slow evaporation of a DCM solution. Compound **4d** crystallizes in the triclinic space group *P*-1 with two molecules in the asymmetric unit.

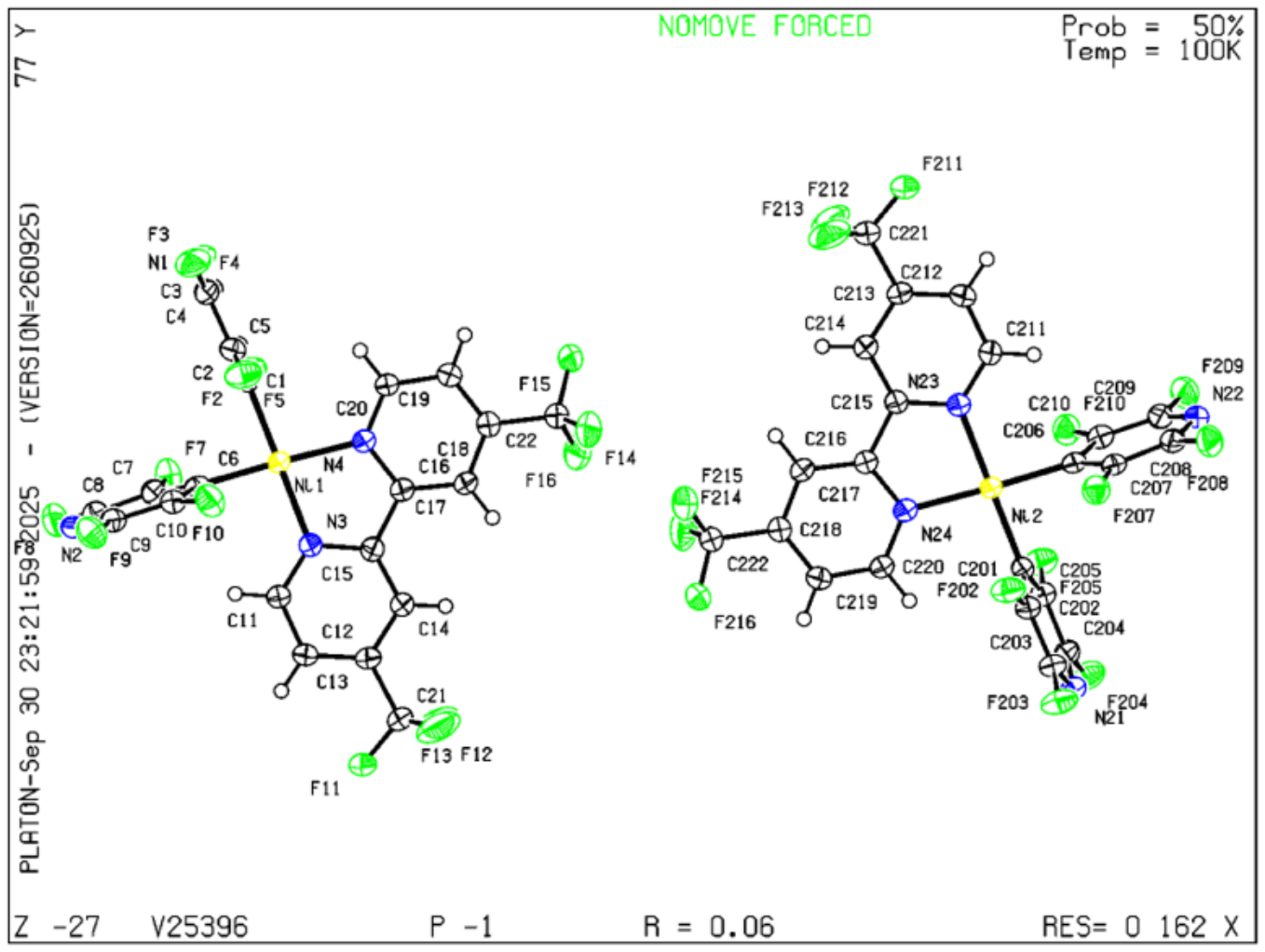

Table S24. Crystal data and structure refinement for 4d (CCDC 2552388)

Empirical formula	$C_{22}H_6N_4F_{14}Ni$
Formula weight	651.02
Temperature/K	100(2)
Crystal system	triclinic
Space group	P-1
a/Å	10.5615(7)
b/Å	11.1365(7)
c/Å	19.9065(12)
α/°	86.249(2)
β/°	74.687(3)
γ/°	88.032(2)
Volume/Å^3	2253.1(2)
Z	4
ρ_{calc}g/cm^3	1.919
μ/mm^{-1}	2.558
F(000)	1280.0
Crystal size/mm^3	0.1 × 0.1 × 0.1
Radiation	CuKα (λ = 1.54178)
2Θ range for data collection/°	4.61 to 150.31
Index ranges	-13 ≤ h ≤ 13, -13 ≤ k ≤ 13, -24 ≤ l ≤ 24
Reflections collected	41887
Independent reflections	9214 [R_{int} = 0.0593, R_{sigma} = 0.0472]
Data/restraints/parameters	9214/0/739
Goodness-of-fit on F^2	1.067
Final R indexes [I>=2σ (I)]	R_1 = 0.0606, wR_2 = 0.1505
Final R indexes [all data]	R_1 = 0.0763, wR_2 = 0.1671
Largest diff. peak/hole / e Å^{-3}	0.71/-0.68

Suitable crystals of **5** were grown from a slow evaporation of a DCM solution. Compound **5** crystallizes in the monoclinic space group *C*2/*c* with half a molecule in the asymmetric unit. The unit cell contains a tube-shaped solvent accessible. A void analysis with the program PLATON[33] revealed the presence of one large void and the program SQUEEZE[34] was used to remove the contribution of the disordered electron density inside this void from the structure factors. SQUEEZE removed the contribution of 56 electrons from a void with volume of 245 Å^3. This electron density corresponds to approximately 1.5 acetonitrile molecules.

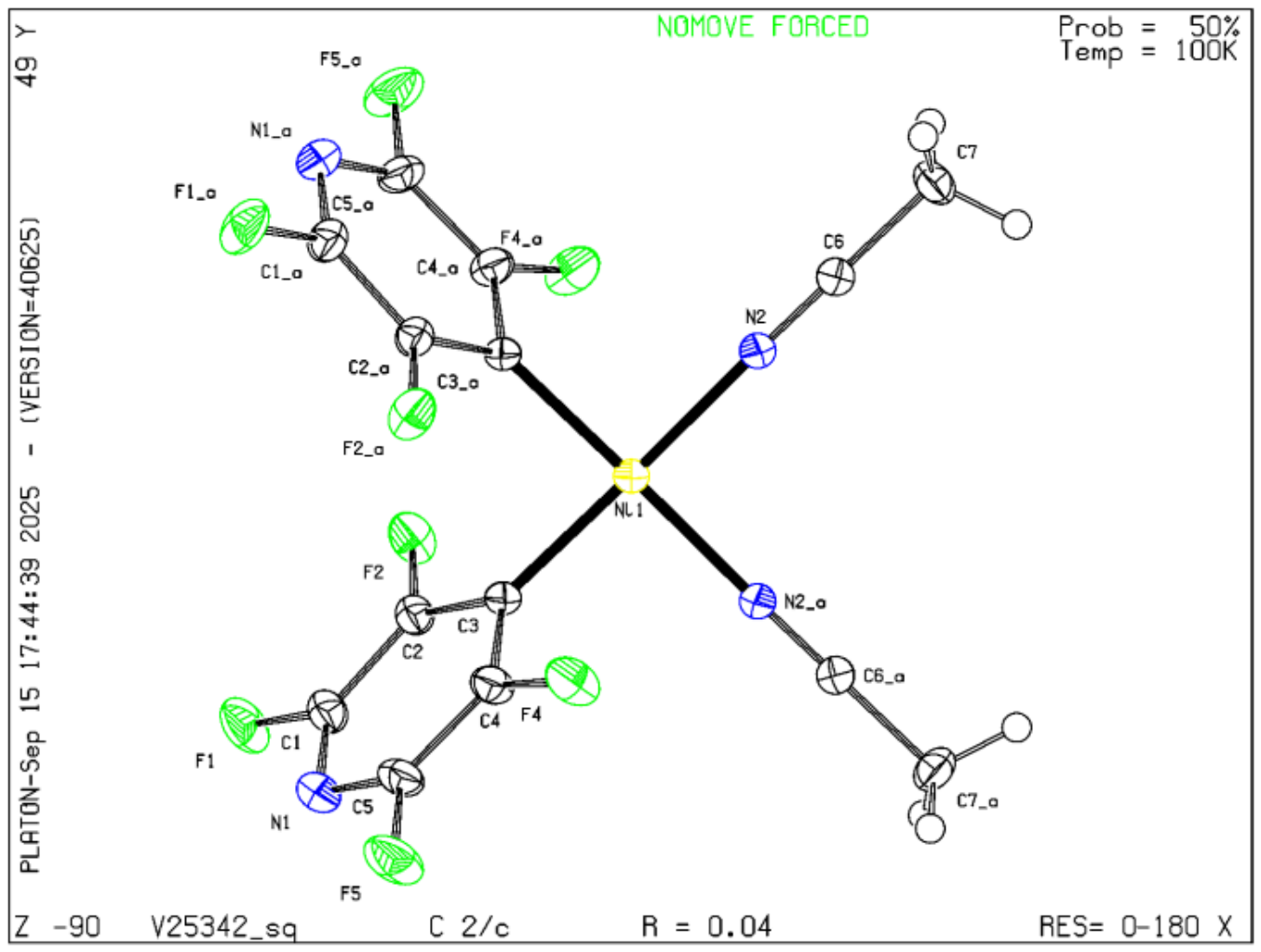

Table S25. Crystal data and structure refinement for 5 (CCDC 2552386)

Empirical formula	$C_{16}H_9N_3F_8Ni$
Formula weight	453.97
Temperature/K	100(2)
Crystal system	monoclinic
Space group	C2/c
a/Å	14.571(4)
b/Å	14.416(3)
c/Å	8.387(2)
α/°	90
β/°	101.330(14)
γ/°	90
Volume/Å^3	1727.5(7)
Z	4
ρ_{calc}g/cm^3	1.746
μ/mm^{-1}	1.211
F(000)	904.0
Crystal size/mm^3	0.2 × 0.15 × 0.15
Radiation	MoKα (λ = 0.71073)
2Θ range for data collection/°	5.652 to 72.624
Index ranges	-24 ≤ h ≤ 22, -23 ≤ k ≤ 23, -13 ≤ l ≤ 13
Reflections collected	16935
Independent reflections	4159 [R_{int} = 0.0546, R_{sigma} = 0.0385]
Data/restraints/parameters	4159/0/124

Goodness-of-fit on F^2	1.114
Final R indexes [I>=2σ (I)]	R_1 = 0.0438, wR_2 = 0.0878
Final R indexes [all data]	R_1 = 0.0642, wR_2 = 0.1003
Largest diff. peak/hole / e $Å^{-3}$	0.61/-0.91

8 References

1 L. Cronin, C. L. Higgitt, R. Karch and R. N. Perutz, *Organometallics*, 1997, **16**, 4920–4928.
2 S. Burling, P. I. P. Elliott, N. A. Jasim, R. J. Lindup, J. McKenna, R. N. Perutz, S. J. Archibald and A. C. Whitwood, *Dalton Trans*, 2005, 3686–3695.
3 G. Yin, I. Kalvet, U. Englert and F. Schoenebeck, *J. Am. Chem. Soc.*, 2015, **137**, 4164–4172.
4 L. Griego, J. B. Chae and L. M. Mirica, *Chem*, 2024, **10**, 867–881.
5 M. W. Kuntze-Fechner, H. Verplancke, L. Tendera, M. Diefenbach, I. Krummenacher, H. Braunschweig, T. B. Marder, M. C. Holthausen and U. Radius, *Chem Sci*, 2020, **11**, 11009–11023.
6 T. Yamamoto, A. Yamamoto and S. Ikeda, *J. Am. Chem. Soc.*, 1971, **93**, 3350–3359.
7 T. Yamamoto, M. Abla and Y. Murakami, *Bull. Chem. Soc. Jpn.*, 2002, **75**, 1997–2009.
8 L. Ju, C. T. Hu and T. Diao, *Organometallics*, 2022, **41**, 1748–1753.
9 B. Mirabi, A. D. Marchese and M. Lautens, *ACS Catal.*, 2021, **11**, 12785–12793.
10 R. S. Kim, L. O. Kgoadi, J. C. Hayes, D. P. Rainboth, C. M. Mudd, G. P. A. Yap and D. A. Watson, *J. Am. Chem. Soc.*, 2024, **146**, 17606–17612.
11 L. E. Ehehalt, O. M. Beleh, I. C. Priest, J. M. Mouat, A. K. Olszewski, B. N. Ahern, A. R. Cruz, B. K. Chi, A. J. Castro, K. Kang, J. Wang and D. J. Weix, *Chem. Rev.*, 2024, **124**, 13397–13569.
12 Z.-M. Su, J. Zhu, D. L. Poole, M. Rafiee, R. S. Paton, D. J. Weix and S. S. Stahl, *J. Am. Chem. Soc.*, 2025, **147**, 353–361.
13 D. A. Cagan, D. Bím, B. J. McNicholas, N. P. Kazmierczak, P. H. Oyala and R. G. Hadt, *Inorg. Chem.*, 2023, **62**, 9538–9551.
14 M. Mohadjer Beromi, G. W. Brudvig, N. Hazari, H. M. C. Lant and B. Q. Mercado, *Angew. Chem. Int. Ed.*, 2019, **58**, 6094–6098.
15 M. Tsednee, Y.-C. Huang, Y.-R. Chen and K.-C. Yeh, *Sci. Rep.*, 2016, **6**, 26785.
16 H. Atout, A. Bouguettoucha, D. Chebli, J. Crespo, J.-C. Dupin, J. M. López-de-Luzuriaga, H. Martínez, M. Monge, M. E. Olmos and M. Rodríguez-Castillo, *New J Chem*, 2021, **45**, 11727–11736.
17 J. Zhang, X. Zhao, J.-D. Yang and J.-P. Cheng, *J. Org. Chem.*, 2022, **87**, 294–300.
18 H. Nakai, K. Jeong, T. Matsumoto and S. Ogo, *Organometallics*, 2014, **33**, 4349–4352.
19 W.-C. Chang, H. Randel, T. Weyhermüller, A. A. Auer, C. Farès and C. Werlé, *Angew. Chem. Int. Ed.*, 2023, **62**, e202219127.
20 Z. Liu, C. Du, J. Han, X. Yang, S. Xia, K. Liu, C. Zhu, W. Li and J. Xie, *Nat. Chem.*, DOI:10.1038/s41557-025-01962-1.
21 F. Neese, *WIREs Comput. Mol. Sci.*, 2012, **2**, 73–78.
22 F. Neese, *WIREs Comput. Mol. Sci.*, 2025, **15**, e70019.
23 V. N. Staroverov, G. E. Scuseria, J. Tao and J. P. Perdew, *J. Chem. Phys.*, 2003, **119**, 12129–12137.
24 J. P. Perdew, J. Tao, V. N. Staroverov and G. E. Scuseria, *J. Chem. Phys.*, 2004, **120**, 6898–6911.
25 F. Weigend and R. Ahlrichs, *Phys. Chem. Chem. Phys. Inc. Faraday Trans.*, 2005, **7**, 3297.
26 S. Grimme, J. Antony, S. Ehrlich and H. Krieg, *J. Chem. Phys.*, 2010, **132**, 154104.
27 S. Grimme, S. Ehrlich and L. Goerigk, *J. Comput. Chem.*, 2011, **32**, 1456–1465.
28 F. Neese, F. Wennmohs, A. Hansen and U. Becker, *Mov. Front. Quantum Chem.*, 2009, **356**, 98–109.
29 K. B. Wiberg, *Tetrahedron*, 1968, **24**, 1083–1096.
30 G. M. Sheldrick, *Acta Crystallogr. Sect. A*, 1990, **46**, 467–473.
31 G. M. Sheldrick, *Acta Crystallogr. Sect. C Struct. Chem.*, 2015, **71**, 3–8.
32 P. Müller, *Crystallogr. Rev.*, 2009, **15**, 57–83.
33 A. L. Spek, *Acta Crystallogr. Sect. C*, 2015, **71**, 9–18.

34 P. van der Sluis and A. L. Spek, *Acta Crystallogr. Sect. A*, 1990, **46**, 194–201.
